# Developing digital tools for health surveys in low- and middle-income countries: Comparing findings of two mobile phone surveys with a nationally representative in-person survey in Bangladesh

**DOI:** 10.1371/journal.pgph.0002053

**Published:** 2023-07-27

**Authors:** Gulam Muhammaed Al Kibria, Saifuddin Ahmed, Iqbal Ansary Khan, Julián A. Fernández-Niño, Andres Vecino-Ortiz, Joseph Ali, George Pariyo, Michelle Kaufman, Aninda Sen, Sunada Basu, Dustin Gibson

**Affiliations:** 1 Department of International Health, Johns Hopkins Bloomberg School of Public Health, Baltimore, MD, United States of America; 2 Department of Population, Family and Reproductive Health, Johns Hopkins Bloomberg School of Public Health, Baltimore, MD, United States of America; 3 Institute of Epidemiology Disease Control and Research (IEDCR), Mohakhali, Dhaka, Bangladesh; 4 Johns Hopkins Berman Institute of Bioethics, Baltimore, MD, United States of America; Makerere University, UGANDA

## Abstract

Non-communicable disease (NCD) risk factor data from low- and middle-income countries (LMICs) are inadequate, mostly due to the cost and burden of collecting in-person population-level estimates. High-income countries regularly use phone-based surveys, and with increasing mobile phone subscription in developing countries, mobile phone surveys (MPS) could complement in-person surveys in LMICs. We compared the representativeness and prevalence estimates of two MPS (i.e., interactive voice response (IVR) and computer-assisted telephone interview (CATI)) with a nationally representative household survey in Bangladesh–the STEPwise approach to NCD risk factor surveillance (STEPs) 2018. This cross-sectional study included 18-69-year-old respondents. CATI and IVR recruitments were done by random digit dialing, while STEPs used multistage cluster sampling design. The prevalence of NCD risk factors related to tobacco, alcohol, diet, and hypertension was reported and compared by prevalence differences (PD) and prevalence ratios (PR). We included 2355 (57% males), 1942 (62% males), and 8185 (47% males) respondents in the CATI, IVR, and STEPs, respectively. CATI (28%) and IVR (52%) had a higher proportion of secondary/above-educated people than STEPs (13%). Most prevalence estimates differed by survey mode; however, CATI estimates were closer to STEPs than IVR. For instance, in CATI, IVR, and STEPs, respectively, the prevalence was 21.4%, 17.9%, and 23.5% for current smoking; and 1.6%, 2.2%, and 1.5% for alcohol drinking in past month. Compared to STEPs, the PD ranged from ‘-56.6% to 0.4%’ in CATI and ‘-41.0% to 8.4%’ in IVR; the PR ranged from ‘0.3 to 1.1’ in CATI and ‘0.3 to 1.6’ in IVR. There were some differences and some similarities in NCD indicators produced by MPS and STEPs with differences likely due to differences in socioeconomic characteristics between survey participants.

## Introduction

Non-communicable diseases (NCDs) are the leading causes of death globally [1, 2]. The burden of these diseases, including heart diseases, strokes, diabetes, obesity, and cancers, is increasing at an alarming rate in low- and middle-income countries (LMICs) [1, 3, 4]. Four modifiable risk factors mostly contribute to this high NCD burden: tobacco consumption, inadequate physical activity, excess alcohol, and unhealthy diets [5]. Many LMICs are also dealing with a ‘double disease burden,’ where there is a higher prevalence of NCDs along with infectious diseases [6, 7]. Bangladesh is an example of such a country, facing the need to simultaneously address significant burdens of NCDs (e.g., diabetes, hypertension, and obesity) alongside ongoing management of communicable diseases like pulmonary tuberculosis [8–10].

To decrease the future burden of NCDs, it is essential to develop effective programs and policies by regularly monitoring both prevalence and trends of these diseases using representative surveys [11]. Although high-income countries regularly conduct such surveys, in-person health surveys are expensive [12]. This high cost is one of the primary obstacles to implementing health surveys in LMICs. Currently, the STEPwise approach to NCD risk factor surveillance (STEPs), global adult tobacco surveys (GATS), demographic and health surveys (DHS), and multiple indicators cluster survey (MICS) are among the common health surveys utilized in LMICs [13–15]. Due to the required costs and efforts, these largely in-person-administered surveys are implemented every 3, 5, or even 10 years [12].

In addition to in-person surveys, public health agencies in high-income countries implement telephone-based surveys to collect data on diseases and risk factors [16]. In the past, lack of access to a telephone was an impediment to implementing such surveys in LMICs. However, recent decades have seen rapid development and adoption of mobile phone technology in LMICs [17, 18]. The mobile phone subscription rates in many LMICs are currently comparable to high-income countries [17, 18] and as such, large-scale mobile phone surveys (MPS) are now being implemented in several LMICs [19]. Furthermore, a large proportion of people in LMICs live in remote or hard-to-reach areas, making data collection from this population group more challenging [20]. Therefore, using mobile phones may allow for interviewing populations in a shorter timeframe at a lower cost. Similar to other LMICs, the mobile phone subscription rate is high in Bangladesh, with 107 subscriptions per 100 people in 2020 [21]. During the coronavirus disease 2019 pandemic (COVID-19) in Bangladesh, MPS has been used to collect data, in contact tracing, and even to provide services in many instances mainly to reduce the risk of virus transmission by interviewing participants remotely [22]. Therefore, MPS could serve as an alternate to in-person data collection method in Bangladesh and other similar LMICs [23].

Considering the promise of MPS to collect population-level data in LMICs, the knowledge regarding its feasibility, validity, and reliability is growing. Several MPS data collection methods are in use, including interactive voice response (IVR) and computer-assisted telephone interviews (CATI) [24, 25]. In IVR, an eligible participant uses their mobile phone’s keypad to provide answers to a prerecorded questionnaire (e.g., “If you are a male, press 1. If you are a female, press 2”). In CATI, live interviewers ask questions and record responses remotely over the phone [18, 26]. Although both MPS methods are in use, how participant demographics in these two survey methods differ from each other or other nationally representative surveys are not known in many LMICs. Furthermore, there has been limited investigation regarding the differences in prevalence estimates by survey type in many LMICs. The lack of IVR, CATI, and in-person survey (e.g., STEPs) data covering the same topics in similar age groups within a shorter time frame is another obstacle to understanding the comparability of these methods. In this study, we attempted to begin to fill the above-mentioned gaps in knowledge by comparing the representativeness and prevalence estimates of IVR, CATI, and STEPs in Bangladesh.

## Methods

### Study design and participants

This was a cross-sectional study. CATI and IVR participants were recruited by random digit dialing (RDD). Quota sampling was used to recruit participants from the following age-sex strata: 18–29, 30–44, 45–59, and ≥60-year-old males and females. STEPs 2018 was a large nationally representative in-person survey in Bangladesh. As STEPs included 18-69-year-old respondents, we limited our analysis to this age group for MPS as well.

### Procedures

Data collection for IVR took place in September 2020. Data collection for CATI took place from August to October 2020. The calls were administered in Bangla language to randomly selected numbers between 08:00 AM and 8:00 PM local time. While generating the numbers, the first three digits of the called phone numbers were the mobile network operator’s base digits and the remaining eight digits were randomly created to complete the eleven digits. After receiving the phone calls, potential participants were informed of the purpose of the survey, its probable duration, sponsoring agencies, and requirements. Participants were told they would be eligible to take the survey if they were at least 18 years old. As we employed age-sex quota (described above), participants were eligible to be enrolled in an age-sex stratum until the sample size for that stratum was met. Then, the participants were asked to provide consent to proceed with the survey. For IVR, participants had to press the button 1 on their mobile phone to indicate consent.

The IVR and CATI surveys had five major components in the following order: a) survey introduction, b) age-sex screening questions, c) consent, d) other demographic questions, and e) NCD modules. Four NCD modules were asked: a) tobacco use (i.e., smoked and smokeless forms), b) alcohol consumption, c) dietary habits (i.e., fruit, vegetable, and salt intake), and d) hypertension (i.e., diagnosis and treatment history). For IVR survey, the NCD modules were randomized to minimize attrition bias. Only participants who completed surveys received the incentive amount (about 1 USD).

Due to a lower proportion of older respondents (i.e., ages 60+), for CATI only, to enroll individuals within this category we used snowball sampling among the respondents whose respective quota was filled up. As those respondents were not eligible to be recruited in the study, we took the opportunity to ask them if they have an older person in their household who would be interested to participate in the study. If they consented, we scheduled an appointment to call the prospective older respondent at the appointed time.

Data collection for STEPs 2018 took place from March to May 2018. It was a population-based survey among 18-69-year-old non-institutionalized people in Bangladesh. It used a multistage cluster sampling design to obtain reliable estimates for men and women in four age groups: 18–24, 25–39, 40–54, and 55–69 [13]. This was based on the 2011 population and housing census conducted by the Bangladesh Bureau of Statistics [27]. A Primary Sampling Unit (PSU) was a geographic area with an average of 113 households. According to that census, Bangladesh had a total of 294,000 PSUs. During the first stage, 62 PSUs were selected in each of the 8 divisions. The PSUs were selected equally from rural and urban regions. After listing all households in all selected PSUs, a fixed number of 20 households were selected from each of the sampled PSUs. One individual was interviewed from each household [13].

### Outcomes

For CATI and IVR, we limited our analyses to people who completed the surveys. Only the prevalence of indicators that were available in all three surveys were compared: current tobacco smoke; current smokeless tobacco use; daily tobacco smoke; daily smokeless tobacco use; current (i.e., past 30 days) alcohol consumption; eating less than 5 servings of fruits-vegetables in a day; adding salt to food while eating; eating processed food high in salt; and diagnosis and treatment of hypertension. NCD indicators were created using STEPs standardized definitions [13].

We made all the variables binary (i.e., yes or no) based on the presence or absence of a risk factor indicator. The skip patterns of the MPS and STEPs questionnaires were considered to generate the indicator variables. For instance, the prevalence (%) of current smokers was calculated by dividing the number of ‘yes’ responses to the question by the number of total (i.e., yes + no) responses to that question. Next, the ‘daily smoking’ question was asked for participants who responded ‘yes’ to the ‘current smoking’ question. Therefore, the denominator of the ‘daily smoker’ variable included total ‘yes’ response to this variable plus the number of ‘no’ responses to the ‘current smoking’ variable.

### Statistical analysis

STEPs provided sample weights. As CATI and IVR did not have sample weights, we used an iterative proportional fitting algorithm (i.e., raking) to generate weights for them [28]. Based on the age, gender, education, and rural-urban location of residence distribution of STEPs, stepwise adjustment of survey sampling was performed for CATI and IVR. It repeated the adjustment process until the difference between the weighted sample distribution and the known population distribution (i.e., STEPs) became smaller based on a prespecified tolerance value and number of iterations.

To understand how these samples differed, we described basic unweighted and weighted sociodemographic characteristics of CATI, IVR, and STEPs participants. Then, we reported weighted prevalence (with 95% confidence interval [CI]) by survey mode. We also stratified the prevalence according to education level across the survey modes. Lastly, using the STEPs sample as the reference, we reported the prevalence differences (PD) and ratios (PR) (with 95% CI) of the studied indicators. We also compared prevalence estimates of CATI and IVR, using CATI as the reference. We used Stata 14.0 (College Station, TX, US) for all the analysis [29]. In Stata, we obtained the PD and PR using a generalized linear regression model with ‘link (identity)’ and ‘link (log)’ functions, respectively. The ‘svy:’ command was used to account for sample weights (S1 File: Do file).

## Results

The CATI, IVR, and STEPs had 56.5% (n = 1330/2355), 61.9% (n = 1203/1942), and 46.5% (n = 3804/8185) male respondents, respectively (Table 1). The proportion of 45-69-year-old respondents was higher in CATI and STEPs than in IVR. The proportion of people with more than secondary education was highest among IVR (51.9%) respondents, followed by CATI (27.5%) and STEPs (13.0%). Following the application of weighting, as expected, the distribution of CATI and IVR respondents was similar to that of STEPs.

**Table 1:** Unweighted Sociodemographic characteristics of participants in NCD-MPS (CATI and IVR) and WHO-STEPs in Bangladesh.

Characteristics	Unweighted, % (n)			Weighted, %		
	CATI	IVR	STEPs	CATI	IVR	STEPs
Sex						
Male	56.5 (1330)	61.9 (1203)	46.5 (3804)	49.3	49.3	49.3
Female	43.5 (1025)	38.1 (739)	53.5 (4381)	50.7	50.7	50.7
Age (in year)						
18–29	33.6 (792)	42.5 (825)	23.7 (1942)	45.1	45.1	42.4
30–44	33.5 (788)	35.4 (687)	42.8 (3505)	27.0	27.0	28.3
45–69	32.9 (775)	22.1 (430)	33.5 (2738)	27.9	27.9	29.3
Education level						
Up to primary	56.6 (1326)	21.1 (409)	76.1 (6211)	74.9	74.9	74.9
>Primary to secondary	15.9 (373)	27 (523)	10.9 (888)	12.6	12.6	12.6
>Secondary	27.5 (645)	51.9 (1007)	13 (1065)	12.5	12.5	12.5
Place of residence						
Urban	29.6 (696)	50.9 (987)	48.9 (4002)	22.5	22.5	22.5
Rural	70.4 (1659)	49.1 (954)	51.1 (4183)	77.5	77.5	77.5

Abbreviations: CATI: Computer Assisted Telephone Interview; IVR: Interactive Voice Response;: STEPs: STEPwise approach to NCD risk factor surveillance

Table 2 shows the weighted prevalence rates by survey mode. Overall, the prevalence for most indicators were higher among STEPs respondents than CATI and IVR respondents. Among CATI, IVR, and STEPs respondents, respectively, the prevalence (95% CI) was 21.4% (19.6 to 23.2), 17.9% (14.9 to 21.3), and 23.5% (22.1 to 24.9) for current smoking; and 20.4% (18.7 to 22.3), 13.0% (9.9 to 16.9), and 27.5% (26.1 to 29.0) for current smokeless tobacco use. The most noticeable higher prevalence was observed for eating <5 servings of fruits-vegetables in a day; although its prevalence (95% CI) was 89.6% (88.6 to 90.6) among STEPs respondents, it was 48.7% (44.1 to 53.3) among IVR, and 33.9% (31.8 to 36.1) among CATI participants. After stratifying the prevalence of the studied indicators by education, we observed that the prevalence rates were not closer to each other for most of the studied indicators (S1–S3 Tables).

**Table 2:** Prevalence (95% CI) of the studied indicators according to survey mode.

Indicator	Prevalence, % (95% CI)		
	CATI	IVR	STEPs
Current smoker	21.4 (19.6,23.2)	17.9 (14.9,21.3)	23.5 (22.1,24.9)
Current smokeless tobacco user	20.4 (18.7,22.3)	13.0 (9.9,16.9)	27.5 (26.1,29.0)
Daily smoker	16.7 (15.1,18.4)	15.4 (12.6,18.7)	22.3 (21.0,23.7)
Daily smokeless tobacco user	13.0 (11.6,14.6)	6.8 (4.8,9.7)	23.9 (22.5,25.3)
Alcohol past month	1.6 (1.1,2.3)	2.2 (1.3,3.8)	1.5 (1.1,2.0)
<5 servings of fruits-veg in a day	33.0 (30.9,35.2)	48.7 (44.1,53.3)	89.6 (88.6,90.6)
Add salt to food while eating	33.9 (31.8,36.1)	28.1 (24.2,32.4)	48.2 (46.5,50.0)
Processed food high in salt	11.9 (10.5,13.5)	21.9 (18.3,25.9)	13.5 (12.3,14.7)
Known raised BP/HTN	14.2 (12.7,15.7)	16.5 (13.1,20.6)	13.7 (12.6,14.9)
Take medication for BP/HTN	56.7 (50.9,62.3)	56.6 (44.5,68.1)	77.2 (73.6,80.5)

Abbreviations: CATI: Computer Assisted Telephone Interview; CI: Confidence interval; IVR: Interactive Voice Response: STEPs: STEPwise approach to NCD risk factor surveillance

As seen in Table 3, CATI and IVR surveys, produced estimates similar to STEPs for some indicators. For instance, compared to STEPs, CATI had similar prevalence for current smoking (PD: -2.1%; 95% CI: -4.4 to 0.2), current alcohol (PD: 0.1; 95% CI: -0.7 to 0.9), eating processed foods high in salt (-1.6; 95% CI: -3.5 to 0.3), and known raised blood pressure (PD: 0.4; 95% CI: -1.4 to 2.3). The remaining six indicators were different (PD> 5% or PR ≠1) between MPS and STEPs, although current smokeless tobacco use (PD: -7.1%; 95% CI: -7.8 to -3.5) and daily smoking (PD: -5.6%; 95% CI: -7.8 to -3.5) collected by CATI were within 7% from STEPs estimates.

**Table 3:** Prevalence difference and prevalence ratios of the studied indicators according to survey mode.

Indicator	CATI vs STEPs (Ref.)		IVR vs STEPs (Ref.)		IVR vs CATI (Ref.)	
	PD, % (95% CI)	PR (95% CI)	PD, % (95% CI)	PR (95% CI)	PD, % (95% CI)	PR (95% CI)
Current smoker	-2.1(-4.4,0.2)	0.9(0.8,1.0)	-5.6**(-9.1,-2.1)	0.8**(0.6,0.9)	-3.5(-7.1,0.2)	0.8(0.7,1.0)
Current smokeless tobacco user	-7.1***(-9.4,-4.8)	0.7***(0.7,0.8)	-14.5***(-18.3,-10.8)	0.5***(0.4,0.6)	-7.4***(-11.3,-3.5)	0.6**(0.5,0.8)
Daily smoker	-5.6***(-7.8,-3.5)	0.7***(0.7,0.8)	-6.9***(-10.2,-3.5)	0.7***(0.6,0.9)	-1.3(-4.7,2.2)	0.9(0.7,1.2)
Daily smokeless tobacco use	-10.8***(-12.9,-8.8)	0.5***(0.5,0.6)	-17.0***(-19.8,-14.3)	0.3***(0.2,0.4)	-6.2***(-9.0,-3.4)	0.5***(0.4,0.8)
Current alcohol	0.1(-0.7,0.9)	1.1(0.6,1.8)	0.8(-0.5,2.1)	1.5(0.8,2.9)	0.7(-0.7,2.0)	1.4(0.7,2.8)
<5 fruit-veg servings/day	-56.6***(-59.0,-54.3)	0.3***(0.3,0.3)	-41.0***(-45.7,-36.2)	0.5***(0.4,0.5)	15.7***(10.6,20.8)	1.5***(1.3,1.7)
Add salt to food in eating	-14.3***(-17.1,-11.5)	0.7***(0.7,0.8)	-20.1***(-24.6,-15.6)	0.6***(0.5,0.7)	-5.8*(-10.5,-1.1)	0.8*(0.7,1.0)
Processed food high in salt	-1.6(-3.5,0.3)	0.9(0.8,1.0)	8.4***(4.3,12.4)	1.6***(1.3,2.0)	9.9***(5.8,14.0)	1.8***(1.5,2.3)
Known raised BP/HTN	0.4(-1.4,2.3)	1.0(0.9,1.2)	2.8(-1.1,6.7)	1.2(0.9,1.5)	2.3(-1.7,6.3)	1.2(0.9,1.5)
Take BP/HTN medication	-20.6***(-27.2,-13.9)	0.7***(0.7,0.8)	-20.6**(-33.1,-8.1)	0.7**(0.6,0.9)	-0.1(-13.4,13.3)	1.0(0.8,1.3)

Abbreviations: CATI: Computer Assisted Telephone Interview; CI: Confidence interval; IVR: Interactive Voice Response; PD: Prevalence difference; PR: Prevalence ratio;: STEPs: STEPwise approach to NCD risk factor surveillance

*: p<0.05

**: p<0.01

***: p<0.001

## Discussion

In this analysis, we evaluated the representativeness and reliability of two MPS methods compared to STEPs. We observed that both MPS methods had a higher proportion of people with secondary/above education than that of STEPs participants. Although the prevalence estimates for CATI were a little closer to STEPs than IVR, the overall prevalence rates were higher in STEPs than in both MPS. To our knowledge, this is the first study comparing demographics and prevalence estimates of two MPS methods with each other and with a nationally representative household survey in an LMIC.

Although MPS and STEPs were conducted approximately 18 months apart, differences between NCD indicator estimates are not likely due to temporality. For instance, STEPs 2009 in Bangladesh yielded slightly higher, but similar estimates for many of the indicators: current smoker (26.2% in 2009 vs 23.5% in 2018), current drinker (0.8% in 2009 vs 1.5% in 2018), and less than 5 servings of fruits and/or vegetables (95.7% in 2009 vs 89.6% in 2018) [13].

The differences in prevalence estimates may result from differences in study samples as well as the survey modes. Overall, a larger proportion of STEPs respondents reported tobacco consumption than in the two MPS. The MPS represented a higher proportion of younger people and people with a higher education level, groups that historically have reported a lower prevalence of alcohol drinking, smoking, or smokeless tobacco use [30, 31]. Using inverse proportional weighting and quota sampling reduced some sample distortion towards the responses of over-represented people. In CATI, an interviewer can also explain questions to respondents, like in the administration of STEPs but not in IVR [32]. This may be the reason for smaller differences between CATI and STEPs compared to differences between IVR and STEPs.

Furthermore, two in-person surveys can generate different prevalence estimates, even differences in wording of questions can obtain different estimates. For instance, in the US, several nationally representative surveys are used to report NCD indicators, such as the National Health and Nutrition Examination Survey (NHANES), National Health Interview Survey (NHIS), and Behavioral Risk Factor Surveillance Survey (BRFSS). The NHANES and NHIS are in-person surveys, but the BRFSS is telephone-based [16, 33, 34]. A study by Keadle et al. compared the estimates for physical activity across these three surveys and found that 27%, 36%, and 44% of adults met the PA guideline’s recommendation as per NHANES, NHIS, and BRFSS, respectively. In Bangladesh and many other LMICs, studies comparing two or more in-person NCD risk factor surveys are sparse. The Bangladesh DHS (BDHS) 2017–18 reported the prevalence of hypertension/raised blood pressure and diabetes among people ages 18 and older [30]. Though the age group of STEPs 2018 was slightly different (i.e., 15-69-year-olds) and due to the lower age group, it was expected to see a relatively lower overall prevalence of hypertension (21% vs 27%) or diabetes (8% vs 10%) in STEPs than in the BDHS [30, 31]. The prevalence of hypertension and diabetes by age group in these two surveys were similar. We did not include the BDHS in our analysis as it covered only a few indicators reported by the CATI and IVR surveys and the age groups of these surveys were different [30]. Considering the similarities between BDHS and STEPs results, we expect the differences in estimates (e.g., PD or PR) between MPS and BDHS would be similar to the differences between MPS and STEPs (e.g., PD> 5% or PR ≠1). Further research is required to understand how MPS can generate representative samples and estimates like STEPs or BDHS.

Although differences in prevalence estimates were observed for most indicators, some indicators (e.g., current smoking) had similar prevalence with a lower PD (<5%) or PR (~1). Future studies should investigate the reasons for the similarities and dissimilarities as well as the reliability of MPS for some other indicators.

Our findings showed that CATI and IVR in Bangladesh may not obtain representative samples or estimates comparable to “gold standard” household surveys like STEPs; therefore, MPS may not replace STEPs in its current form. Moreover, the prevalence estimates of CATI and IVR were substantially different from one another for some indicators. These findings confirm several other studies from LMICs. For instance, Greenleaf et al. compared the contraceptive prevalence rate of a CATI survey with an in-person survey, Performance Monitoring and Accountability 2020, in Burkina Faso. They also found the odds of reporting contraceptive use to be twice as high among CATI respondents compared to IVR respondents [35]. Another study by L’Engle et al. compared their findings with the Ghana DHS; they reported large differences between studied indicators [36].

Despite multiple attempts and using quota sampling, we were unable to recruit a sufficient number of respondents in some age-sex strata (e.g., older people and females) of MPS. A lower proportion of females own mobile phones in LMICs compared to men or may not have sufficient access to mobile charging stations and/or airtime credit [37–39]. Furthermore, women tend to spend more time doing household chores and taking care of children compared to men and may not be as available to answer phone surveys [36].

We were able to recruit a larger proportion of older respondents (i.e., those ages 60+) in CATI by scheduling a time to call the older people of a household after talking to a younger household member. Similar scheduling can be done in IVR. Some other methods have also been shown to increase survey participation for some populations, such as using a female voice, motivational introductory messages, airtime incentives, and sending pre-survey text messages [24, 26, 32, 40, 41]. The usefulness of these methods should also be tested.

This study has several notable strengths. We adjusted for age, sex, place of residence, and education to generate sample weights for CATI and IVR; this reduces the bias related to sample selection. Then, as CATI and IVR included only randomly selected respondents, it reduced the selection bias. This study compared two MPS samples with a STEPs sample and used the STEPs sample’s weight as the reference, thereby increasing the authenticity of our results. The sample sizes of both MPS were large enough to compare the findings with STEPs (S2 and S3 Files).

This study has some limitations as well. The STEPs sample was obtained with a goal of collecting reliable samples from each administrative division in Bangladesh; however, the MPS samples did not have data on administrative divisions, and we were unable to add this variable in calculating the inverse proportional weight of the study sample. BDHS, STEPs, and other nationally representative surveys have shown large differences in health behaviors and outcomes by administrative divisions in Bangladesh [30]. The measurement (i.e., wording of questions) of variables may also cause some differences in responses. Despite obtaining two large MPS samples, by design, these study arms only included respondents who use personal mobile phones. Variables associated with mobile phone ownership are also associated with NCD risk factors; therefore, the responses of people without mobile phone use are not known [38, 42, 43]. It is also important to note that the prevalence of some indicators (e.g., hypertension, diabetes or dyslipidemia) may vary as a substantial proportion of people may be unaware of having the conditions, and MPS would not be able to obtain these indicators’ prevalence.

## Conclusions

Although CATI and IVR obtained large samples, there were some differences, and some similarities, in NCD indicator estimates between MPS and STEPs samples. Differences are likely due to sociodemographic differences between MPS and STEPs participants, including the design of the surveys. Considering the promise of MPS to monitor the prevalence and trends of NCDs, future studies should investigate how the representativeness can be increased.

## Supporting information

S1 File Do file of the study. (DOCX)

S2 File STROBE checklist. (DOCX)

S3 File Inclusivity statement. (DOCX)

S1 Table Prevalence (95% CI) of the studied indicators according to survey mode among people with ‘up to primary’ education level. (DOCX)

S2 Table Prevalence (95% CI) of the studied indicators according to survey mode among people with ‘more than primary’ to ‘up to secondary’ education level. (DOCX)

S3 Table Prevalence (95% CI) of the studied indicators according to survey mode among people with ‘more than secondary’ education level. (DOCX)

## Abbreviations

BRFSS: Survey and Behavioral Risk Factor Surveillance Survey
CATI: Computer-assisted telephone interview
DHS: Demographic and health survey
IVR: Interactive voice response
LMIC: Low- and middle-income country
MICS: Multiple indicator cluster survey
MPS: Mobile phone surveys
NCD: Non-communicable disease
NHANES: National Health and Nutrition Examination Survey
NHIS: National Health Interview
PD: Prevalence difference
PR: Prevalence ratios
STEPs: STEPwise approach to NCD risk factor surveillance
