# Supplementary material for: Developing digital tools for health surveys in low- and middle-income countries: Comparing findings of two mobile phone surveys with a nationally representative in-person survey in Bangladesh
Source: PLOS Glob Public Health. 2023 Jul 27;3(7):e0002053. doi: 10.1371/journal.pgph.0002053 (PMC10374008; doi:10.1371/journal.pgph.0002053)
Supplement: S1 File — (DOCX) [file pgph.0002053.s001.docx]

/*append using "analytic_file_STEPS_GMAK_19Jul2022.dta"

cap label drop file_name

recode file_name (2 = 1)

label define file_name 0"IVR" 1"STEPS"

label val file_name file_name

tab file_name

*/

/*

Male 0.032743426 0.076684293 0.070703859 0.063065213 0.054987049 0.047201703 0.041984828 0.034916566 0.028686175 0.023342933 0.016490457

Female 0.031892421 0.075354435 0.07047088 0.065819594 0.060343188 0.052582137 0.045195061 0.035494797 0.030106714 0.024849897 0.017082039

*/

clear

input sex_n age0 age1 age2 age3 age4 age5 age6 age7 age8 age9 age10

1 0.032743426 0.076684293 0.070703859 0.063065213 0.054987049 0.047201703 0.041984828 0.034916566 0.028686175 0.023342933 0.016490457

2 0.031892421 0.075354435 0.07047088 0.065819594 0.060343188 0.052582137 0.045195061 0.035494797 0.030106714 0.024849897 0.017082039

end

reshape long age, i(sex_n) j(agecat)

rename age popdist

sort agecat sex_n

save popdist, replace

********************************************************************************

use "analytic_file_IVR_GMAK_19Jul2022.dta", replace

*use "C:\study\D4H\Kibria\Final Tanzania IVR 2020.dta", clear

gen agecat=int((age_n-15)/5)

gen id=_n

svyset id

tab agecat

svy: ta agecat sex

matrix b=e(b)

*observed age-sex proportion

gen ivrprop=.

forvalues i=1(1)17 {

replace ivrprop=b[1,`i'*2-1] if agecat==`i'-1 & sex_n==1

replace ivrprop=b[1,`i'*2] if agecat==`i'-1 & sex_n==2

}

sort agecat sex_n

merge agecat sex_n using popdist

gen age_sex_wt=popdist/ivrprop

recode age_n (18 19 = .) (20/24 = 0) (25/29 = 1) (30/34 = 2) (35/39 = 3) ///

(40/44 = 4) (45/49 = 5) (50/54 = 6) (55/59 = 7) ///

(60/64 = 8) (65/69 = 9), gen(age_cat_w)

tab age_cat_w

tab age_n age_cat_w

append using "analytic_file_STEPS_GMAK_19Jul2022.dta"

gen age_cat = 0 if age_n >= 18 & age_n <= 29

replace age_cat = 1 if age_n >= 30 & age_n <= 44

replace age_cat = 2 if age_n >= 45 & age_n <= 69

label def age_cat 0 "18-29" 1 "30-44" 2 "45-69"

label val age_cat age_cat

label var age_cat "Age (in year)"

tab age_cat

tab sex_n

tab edu_n

tab edu_n, nol

recode edu_n (1 3 = 0) (5 7 = 1) (9 66 = 2), gen(school_n)

tab school_n

label define school_n 0 "Up to primary" 1 "More than primary to up to secondary" 2 "More than secondary"

label val school_n school_n

label var school_n "Education level"

tab school_n file_name

tab location_n

tab location_n, nol

recode location_n (3 = 1) (1 = 0), gen(por)

label def por 0"Urban" 1"Rural"

label val por por

label var por "Location of residence"

tab por

gen file1 = 0 if file_name == 0

replace file1 = 1 if file_name == 2

label def file1 0"IVR" 1"STEPS"

label val file1 file1

tab file1

tab file1

tab file1, nol

gen weight1 = 1 if file1 == 0

replace weight1 = wstep1 if file1 == 1

for var age_cat sex school por: prop X [iw=wstep1] if file1==1

for var age_cat sex school por: prop X if file1==0

tab1 file1 age_cat sex school_n por

tab1 file1 age_cat sex school_n por if file1 == 0

tab1 file1 age_cat sex school_n por if file1 == 1

logit file1 i.age_cat i.sex i.school_n i.por [iw = weight1]

predict p

gen weight_ipw = wstep1 if file1 == 1

replace weight_ipw = 1/(1-p) if file1 == 0

replace age_sex_w = wstep1 if age_sex_w == .

bys file1: sum weight_ipw

tab sex file1 [iweight = weight_ipw], col

******

label var current_smoke_n "Current smoker"

label var daily_smoke_n "Daily smoker"

label var past_smoke_n "Past smoker"

label var past_smoke_daily_n "past daily smoker"

label var current_smokeless_n "Current smokeless tobacco user"

label var daily_smokeless_n "Daily smokeless tobacco user"

label var past_smokeless_n "Past smokeless tobacco user"

label var current_tobacco_any "Any current tobacco user"

label var daily_tobacco_any "Any daily tobacco user"

label var alc_past_year "Alcohol past year"

label var alcohol_current "Alcohol past month"

label var no_fruit_veg "No fruit or vegetables"

label var no_fruit "No fruit"

label var no_veg "No veg"

label var atelessthan5 "<5 servings of fruits-veg in a day "

label var salt_eat_n "Add salt to food while eating"

label var salt_pack_n "Processed food high in salt"

label var htn_known "Known raised BP/HTN"

label var htn_med "Take medication for BP/HTN"

label var smoke_only "Only smoker"

label var smokeless_only "Only smokeless tobacco user"

label var both_smoke_smokeless "Both smoker and smokeless user"

*****

tab current_smoke_n file1, col

tab current_smoke_n file1 [iweight = weight_ipw], col

tab current_smoke_n file1 [iweight = age_sex_w], col

tab daily_smoke_n file1, col

tab daily_smoke_n file1 [iweight = weight_ipw], col

tab daily_smoke_n file1 [iweight = age_sex_w], col

tab past_smoke_n file1, col

tab past_smoke_n file1 [iweight = weight_ipw], col

tab past_smoke_n file1 [iweight = age_sex_w], col

tab past_smoke_daily_n file1, col

tab past_smoke_daily_n file1 [iweight = weight_ipw], col

tab past_smoke_daily_n file1 [iweight = age_sex_w], col

*******

tab current_smokeless_n file1, col

tab current_smokeless_n file1 [iweight = weight_ipw], col

tab current_smokeless_n file1 [iweight = age_sex_w], col

tab daily_smokeless_n file1, col

tab daily_smokeless_n file1 [iweight = weight_ipw], col

tab daily_smokeless_n file1 [iweight = age_sex_w], col

tab past_smokeless_n file1, col

tab past_smokeless_n file1 [iweight = weight_ipw], col

tab past_smokeless_n file1 [iweight = age_sex_w], col

*****

tab current_tobacco_any file1, col

tab current_tobacco_any file1 [iweight = weight_ipw], col

tab current_tobacco_any file1 [iweight = age_sex_w], col

*****

tab alc_past_year file1, col

tab alc_past_year file1 [iweight = weight_ipw], col

tab alc_past_year file1 [iweight = age_sex_w], col

tab alcohol_current file1, col

tab alcohol_current file1 [iweight = weight_ipw], col

tab alcohol_current file1 [iweight = age_sex_w], col

*******

tab atelessthan5 file1, col

tab atelessthan5 file1 [iweight = weight_ipw], col

tab atelessthan5 file1 [iweight = age_sex_w], col

tab salt_eat_n file1, col

tab salt_eat_n file1 [iweight = weight_ipw], col

tab salt_eat_n file1 [iweight = age_sex_w], col

tab salt_pack_n file1, col

tab salt_pack_n file1 [iweight = weight_ipw], col

tab salt_pack_n file1 [iweight = age_sex_w], col

save working_file, replace

*******

/* tab htn_known file1, col

tab htn_known file1 [iweight = weight_ipw], col

tab htn_known file1 [iweight = age_sex_w], col

tab htn_med file1, col

tab htn_med file1 [iweight = weight_ipw], col

tab htn_med file1 [iweight = age_sex_w], col

*Table 3: Prevalence of tobacco use indicators by survey mode

1.Both Sexes, 18-29

2.Both Sexes, 30-44

3.Both Sexes, 45-69

4.Both Sexes, 18-69

5.Males, 18-29

6.Males, 30-44

7.Males, 45-69

8.Males, 18-69

9.Females, 18-29

10.Females, 30-44

11.Females, 45-69

12.Females, 18-69

13.M, 18-29 years, Up to primary

14.M, 18-29 years, More than primary to up to secondary

15.M, 18-29 years, More than secondary

16.M, 30-44 years, Up to primary

17.M, 30-44 years, More than primary to up to secondary

18.M, 30-44 years, More than secondary

19.M, 45-69 years, Up to primary

20.M, 45-69 years, More than primary to up to secondary

21.M, 45-69 years, More than secondary

22.M, 18-69 years, Up to primary

23.M, 18-69 years, More than primary to up to secondary

24.M, 18-69 years, More than secondary

25.F, 18-29 years, Up to primary

26.F, 18-29 years, More than primary to up to secondary

27.F, 18-29 years, More than secondary

28.F, 30-44 years, Up to primary

29.F, 30-44 years, More than primary to up to secondary

30.F, 30-44 years, More than secondary

31.F, 45-69 years, Up to primary

32.F, 45-69 years, More than primary to up to secondary

33.F, 45-69 years, More than secondary

34.F, 18-69 years, Up to primary

35.F, 18-69 years, More than primary to up to secondary

36.F, 18-69 years, More than secondary

37.B, 18-29 years, Up to primary

38.B, 18-29 years, More than primary to up to secondary

39.B, 18-29 years, More than secondary

40.B, 30-44 years, Up to primary

41.B, 30-44 years, More than primary to up to secondary

42.B, 30-44 years, More than secondary

43.B, 45-69 years, Up to primary

44.B, 45-69 years, More than primary to up to secondary

45.B, 45-69 years, More than secondary

46.B, 18-69 years, Up to primary

47.B, 18-69 years, More than primary to up to secondary

48.B, 18-69 years, More than secondary */

save working_file, replace

use working_file, replace

**Table 1 Demo

*************************************************************************

*Table 1 Demo

preserve

tabout sex_n age_cat school_n file1 ///

using Table3_n.xls, ///

c(freq col) f(1c 1p) replace

import delimited using Table3_n.xls, clear

drop if v1 == "Total"

drop v6 v7

drop if v1 == ""

destring v2 v4, ignore(",") replace

destring v3 v5, ignore("%") replace

rename v2 IVR_n

rename v4 STEPS_n

rename v3 IVR_p

rename v5 STEPS_p

gen id = _n

save Table1_Dem_UW, replace

restore

*******

preserve

tabout sex_n age_cat school_n file1 [iweight = weight_ipw] ///

using Table3_n.xls, ///

c(freq col) f(1c 1p) replace

import delimited using Table3_n.xls, clear

drop if v1 == "Total"

drop v6 v7

drop if v1 == ""

destring v2 v4, ignore(",") replace

destring v3 v5, ignore("%") replace

rename v2 IVR_n_IPW

rename v4 STEPS_n_IPW

rename v3 IVR_p_IPW

rename v5 STEPS_p_IPW

gen id = _n

save Table1_Dem_IPW, replace

restore

*******

preserve

tabout sex_n age_cat school_n file1 [iweight = age_sex_w] ///

using Table3_n.xls, ///

c(freq col) f(1c 1p) replace

import delimited using Table3_n.xls, clear

drop if v1 == "Total"

drop v6 v7

drop if v1 == ""

destring v2 v4, ignore(",") replace

destring v3 v5, ignore("%") replace

rename v2 IVR_n_Age_Sex_W

rename v4 STEPS_n_Age_Sex_W

rename v3 IVR_p_Age_Sex_W

rename v5 STEPS_p_Age_Sex_W

gen id = _n

save Table1_Dem_Age_Sex_W, replace

restore

****************

preserve

svyset [pweight= weight_ipw]

tabout sex_n age_cat school_n file1 ///

using Table3.xls, ///

c(col ci) f(1 1 1) percent svy replace

import delimited using Table3.xls, clear

drop v6 v7

drop if v1 == "Total"

drop if v1 == ""

gen id = _n

drop v2 v4

rename v3 IVR_IPW_CI

rename v5 STEPS_IPW_CI

save Table1_Dem_IPW_CI, replace

restore

****************

preserve

svyset [pweight= age_sex_w]

tabout sex_n age_cat school_n file1 ///

using Table3.xls, ///

c(col ci) f(1 1 1) percent svy replace

import delimited using Table3.xls, clear

drop v6 v7

drop if v1 == "Total"

drop if v1 == ""

gen id = _n

drop v2 v4

rename v3 IVR_Age_Sex_W_CI

rename v5 STEPS_Age_Sex_W_CI

save Table1_Dem_Age_Sex_W_CI, replace

restore

********

preserve

use Table1_Dem_UW, replace

merge 1:1 id using Table1_Dem_IPW, nogen

merge 1:1 id using Table1_Dem_Age_Sex_W, nogen

merge 1:1 id using Table1_Dem_IPW_CI, nogen

merge 1:1 id using Table1_Dem_Age_Sex_W_CI, nogen

gen IVR_p1 = IVR_p

gen IVR_n1 = IVR_n

keep v1 IVR_p IVR_p_IPW IVR_IPW_CI IVR_n_IPW IVR_n ///

IVR_p1 IVR_p_Age_Sex_W IVR_Age_Sex_W_CI IVR_n_Age_Sex_W IVR_n1 ///

STEPS_p STEPS_p_Age_Sex_W STEPS_Age_Sex_W_CI STEPS_n_Age_Sex_W STEPS_n

order v1 IVR_p IVR_p_IPW IVR_IPW_CI IVR_n_IPW IVR_n ///

IVR_p1 IVR_p_Age_Sex_W IVR_Age_Sex_W_CI IVR_n_Age_Sex_W IVR_n1 ///

STEPS_p STEPS_p_Age_Sex_W STEPS_Age_Sex_W_CI STEPS_n_Age_Sex_W STEPS_n

export excel using Table1_Demo_IVRvsSTEPS.xls, replace

cap erase Table1_Dem_UW.dta

cap erase Table1_Dem_IPW.dta

cap erase Table1_Dem_Age_Sex_W.dta

cap erase Table1_Dem_IPW_CI.dta

cap erase Table1_Dem_Age_Sex_W_CI.dta

restore

****************************************************************************

preserve

tabout current_tobacco_any current_smokeless_n daily_smokeless_n ///

current_smoke_n daily_smoke_n ///

alcohol_current atelessthan5 ///

no_fruit_veg salt_eat_n salt_pack_n ///

htn_known htn_med file1 ///

using Table3_n.xls, ///

c(freq col) f(1c 1p) replace

import delimited using Table3_n.xls, clear

drop if v1 == "No"

drop if v1 == "Yes"

replace v1 = v1[_n - 1] if v1 == "Total"

drop v3 v5 v6 v7

drop if v1 == ""

drop if v2 == ""

destring v2 v4, ignore(",") replace

rename v2 IVR_n

rename v4 STEPS_n

gen id = _n

save Table3_n.dta, replace

restore

********

preserve

svyset [pweight= weight_ipw]

for var current_tobacco_any current_smokeless_n daily_smokeless_n ///

current_smoke_n daily_smoke_n ///

alcohol_current atelessthan5 ///

no_fruit_veg salt_eat_n salt_pack_n ///

htn_known htn_med: svy: proportion X, over(file1)

tabout current_tobacco_any current_smokeless_n daily_smokeless_n ///

current_smoke_n daily_smoke_n ///

alcohol_current atelessthan5 ///

no_fruit_veg salt_eat_n salt_pack_n ///

htn_known htn_med file1 ///

using Table3_Weight_IPW.xls, ///

c(col ci) f(1 1) percent svy replace

import delimited using Table3_Weight_IPW.xls, clear

drop v6 v7

drop if v1 == "No" | v1 == "Total"

replace v1 = v1[_n - 1] if v1 == "Yes"

drop if v2 == ""

drop if v1 == ""

rename v2 IVR_IPW

rename v4 STEPS_IPW

rename v3 IVR_IPW_CI

rename v5 STEPS_IPW_CI

gen id = _n

save Table3_Weighted_IPW.dta, replace

restore

********

preserve

svyset [pweight= age_sex_w]

for var current_tobacco_any current_smokeless_n daily_smokeless_n ///

current_smoke_n daily_smoke_n ///

alcohol_current atelessthan5 ///

no_fruit_veg salt_eat_n salt_pack_n ///

htn_known htn_med: svy: proportion X, over(file1)

tabout current_tobacco_any current_smokeless_n daily_smokeless_n ///

current_smoke_n daily_smoke_n ///

alcohol_current atelessthan5 ///

no_fruit_veg salt_eat_n salt_pack_n ///

htn_known htn_med file1 ///

using Table3_Weight_Age_Sex_W.xls, ///

c(col ci) f(1 1) percent svy replace

import delimited using Table3_Weight_Age_Sex_W.xls, clear

drop v6 v7

drop if v1 == "No" | v1 == "Total"

replace v1 = v1[_n - 1] if v1 == "Yes"

drop if v2 == ""

drop if v1 == ""

rename v2 IVR_Age_Sex

rename v4 STEPS_Age_Sex

rename v3 IVR_Age_Sex_CI

rename v5 STEPS_Age_Sex_CI

gen id = _n

save Table3_Weighted_Age_Sex_W.dta, replace

restore

********

preserve

recode file1 (1 = 0) (0 = 1)

svyset [pweight= weight_ipw]

gen file2 = file1

gen file3 = file1

gen file4 = file1

gen file5 = file1

gen file6 = file1

gen file7 = file1

gen file8 = file1

gen file9 = file1

gen file10 = file1

gen file11 = file1

gen file12 = file1

svy: glm current_tobacco_any file1, fam(bin) link(identity)

estimates store I1

svy: glm current_smokeless_n file2, fam(bin) link(identity)

estimates store I2

svy: glm daily_smokeless_n file3, fam(bin) link(identity)

estimates store I3

svy: glm current_smoke_n file4, fam(bin) link(identity)

estimates store I4

svy: glm daily_smoke_n file5, fam(bin) link(identity)

estimates store I5

svy: glm alcohol_current file6, fam(bin) link(identity)

estimates store I6

svy: glm atelessthan5 file7, fam(bin) link(identity)

estimates store I7

svy: glm no_fruit_veg file8, fam(bin) link(identity)

estimates store I8

svy: glm salt_eat_n file9, fam(bin) link(identity)

estimates store I9

svy: glm salt_pack_n file10, fam(bin) link(identity)

estimates store I10

svy: glm htn_known file11, fam(bin) link(identity)

estimates store I11

svy: glm htn_med file12, fam(bin) link(identity)

estimates store I12

estout I1 I2 I3 I4 I5 I6 I7 I8 I9 I10 I11 I12 using Table_RD.xls, ///

cells ("b(star fmt(1)) ci(par fmt(1) label(95% CI)) z(fmt(2)) p (fmt(4)) ") replace transform(@*100)

import delimited "Table_RD.xls", clear

replace v2 = v6 if v2 == ""

replace v2 = v10 if v2 == ""

replace v2 = v14 if v2 == ""

replace v2 = v18 if v2 == ""

replace v2 = v22 if v2 == ""

replace v2 = v26 if v2 == ""

replace v2 = v30 if v2 == ""

replace v2 = v34 if v2 == ""

replace v2 = v38 if v2 == ""

replace v2 = v42 if v2 == ""

replace v2 = v46 if v2 == ""

replace v3 = v7 if v3 == ""

replace v3 = v11 if v3 == ""

replace v3 = v15 if v3 == ""

replace v3 = v19 if v3 == ""

replace v3 = v23 if v3 == ""

replace v3 = v27 if v3 == ""

replace v3 = v31 if v3 == ""

replace v3 = v35 if v3 == ""

replace v3 = v39 if v3 == ""

replace v3 = v43 if v3 == ""

replace v3 = v47 if v3 == ""

replace v4 = v8 if v4 == ""

replace v4 = v12 if v4 == ""

replace v4 = v16 if v4 == ""

replace v4 = v20 if v4 == ""

replace v4 = v24 if v4 == ""

replace v4 = v28 if v4 == ""

replace v4 = v32 if v4 == ""

replace v4 = v36 if v4 == ""

replace v4 = v40 if v4 == ""

replace v4 = v44 if v4 == ""

replace v4 = v48 if v4 == ""

replace v5 = v9 if v5 == ""

replace v5 = v13 if v5 == ""

replace v5 = v17 if v5 == ""

replace v5 = v21 if v5 == ""

replace v5 = v25 if v5 == ""

replace v5 = v29 if v5 == ""

replace v5 = v33 if v5 == ""

replace v5 = v37 if v5 == ""

replace v5 = v41 if v5 == ""

replace v5 = v45 if v5 == ""

replace v5 = v49 if v5 == ""

keep v1 v2 v3 v4 v5

drop if v1 == ""

drop if v1 == "main"

drop if v1 == "_cons"

destring v1, replace ignore(file)

rename v2 Diff_IVR_IPW

rename v3 Diff_IVR_IPW_CI

rename v4 Diff_IVR_IPW_Z

rename v5 Diff_IVR_IPW_p

gen number = v1

tostring v1, replace

replace v1 = "Any current tobacco user" if v1 == "1"

replace v1 = "Current smokeless tobacco user" if v1 == "2"

replace v1 = "Daily smokeless tobacco user" if v1 == "3"

replace v1 = "Current smoker" if v1 == "4"

replace v1 = "Daily smoker" if v1 == "5"

replace v1 = "Alcohol past month" if v1 == "6"

replace v1 = "<5 servings of fruits-veg in a day " if v1 == "7"

replace v1 = "No fruit or vegetables" if v1 == "8"

replace v1 = "Add salt to food while eating" if v1 == "9"

replace v1 = "Processed food high in salt" if v1 == "10"

replace v1 = "Known raised BP/HTN" if v1 == "11"

replace v1 = "Take medication for BP/HTN" if v1 == "12"

save File_RD_IVR_IPW, replace

restore

********

preserve

recode file1 (1 = 0) (0 = 1)

svyset [pweight= age_sex_w]

gen file2 = file1

gen file3 = file1

gen file4 = file1

gen file5 = file1

gen file6 = file1

gen file7 = file1

gen file8 = file1

gen file9 = file1

gen file10 = file1

gen file11 = file1

gen file12 = file1

svy: glm current_tobacco_any file1, fam(bin) link(identity)

estimates store I1

svy: glm current_smokeless_n file2, fam(bin) link(identity)

estimates store I2

svy: glm daily_smokeless_n file3, fam(bin) link(identity)

estimates store I3

svy: glm current_smoke_n file4, fam(bin) link(identity)

estimates store I4

svy: glm daily_smoke_n file5, fam(bin) link(identity)

estimates store I5

svy: glm alcohol_current file6, fam(bin) link(identity)

estimates store I6

svy: glm atelessthan5 file7, fam(bin) link(identity)

estimates store I7

svy: glm no_fruit_veg file8, fam(bin) link(identity)

estimates store I8

svy: glm salt_eat_n file9, fam(bin) link(identity)

estimates store I9

svy: glm salt_pack_n file10, fam(bin) link(identity)

estimates store I10

svy: glm htn_known file11, fam(bin) link(identity)

estimates store I11

svy: glm htn_med file12, fam(bin) link(identity)

estimates store I12

estout I1 I2 I3 I4 I5 I6 I7 I8 I9 I10 I11 I12 using Table_RD.xls, ///

cells ("b(star fmt(1)) ci(par fmt(1) label(95% CI)) z(fmt(2)) p (fmt(4)) ") replace transform(@*100)

import delimited "Table_RD.xls", clear

replace v2 = v6 if v2 == ""

replace v2 = v10 if v2 == ""

replace v2 = v14 if v2 == ""

replace v2 = v18 if v2 == ""

replace v2 = v22 if v2 == ""

replace v2 = v26 if v2 == ""

replace v2 = v30 if v2 == ""

replace v2 = v34 if v2 == ""

replace v2 = v38 if v2 == ""

replace v2 = v42 if v2 == ""

replace v2 = v46 if v2 == ""

replace v3 = v7 if v3 == ""

replace v3 = v11 if v3 == ""

replace v3 = v15 if v3 == ""

replace v3 = v19 if v3 == ""

replace v3 = v23 if v3 == ""

replace v3 = v27 if v3 == ""

replace v3 = v31 if v3 == ""

replace v3 = v35 if v3 == ""

replace v3 = v39 if v3 == ""

replace v3 = v43 if v3 == ""

replace v3 = v47 if v3 == ""

replace v4 = v8 if v4 == ""

replace v4 = v12 if v4 == ""

replace v4 = v16 if v4 == ""

replace v4 = v20 if v4 == ""

replace v4 = v24 if v4 == ""

replace v4 = v28 if v4 == ""

replace v4 = v32 if v4 == ""

replace v4 = v36 if v4 == ""

replace v4 = v40 if v4 == ""

replace v4 = v44 if v4 == ""

replace v4 = v48 if v4 == ""

replace v5 = v9 if v5 == ""

replace v5 = v13 if v5 == ""

replace v5 = v17 if v5 == ""

replace v5 = v21 if v5 == ""

replace v5 = v25 if v5 == ""

replace v5 = v29 if v5 == ""

replace v5 = v33 if v5 == ""

replace v5 = v37 if v5 == ""

replace v5 = v41 if v5 == ""

replace v5 = v45 if v5 == ""

replace v5 = v49 if v5 == ""

keep v1 v2 v3 v4 v5

drop if v1 == ""

drop if v1 == "main"

drop if v1 == "_cons"

destring v1, replace ignore(file)

rename v2 Diff_IVR_Age_Sex

rename v3 Diff_IVR_Age_Sex_CI

rename v4 Diff_IVR_Age_Sex_Z

rename v5 Diff_IVR_Age_Sex_p

gen number = v1

tostring v1, replace

replace v1 = "Any current tobacco user" if v1 == "1"

replace v1 = "Current smokeless tobacco user" if v1 == "2"

replace v1 = "Daily smokeless tobacco user" if v1 == "3"

replace v1 = "Current smoker" if v1 == "4"

replace v1 = "Daily smoker" if v1 == "5"

replace v1 = "Alcohol past month" if v1 == "6"

replace v1 = "<5 servings of fruits-veg in a day " if v1 == "7"

replace v1 = "No fruit or vegetables" if v1 == "8"

replace v1 = "Add salt to food while eating" if v1 == "9"

replace v1 = "Processed food high in salt" if v1 == "10"

replace v1 = "Known raised BP/HTN" if v1 == "11"

replace v1 = "Take medication for BP/HTN" if v1 == "12"

save File_RD_IVR_Age_Sex, replace

restore

********

preserve

use Table3_n.dta, replace

merge 1:1 v1 using Table3_Weighted_IPW, nogen

merge 1:1 v1 using Table3_Weighted_Age_Sex_W.dta, nogen

sort id

drop id

label var v1 "Indicator"

drop STEPS_IPW

gen indicator = _n

gen pop_group = 12

merge 1:1 v1 using File_RD_IVR_IPW.dta, nogen

merge 1:1 v1 using File_RD_IVR_Age_Sex.dta, nogen

sort number

order v1 IVR_n IVR_IPW IVR_IPW_CI STEPS_n STEPS_Age_Sex STEPS_Age_Sex_CI Diff_IVR_IPW

export excel v1 IVR_n IVR_IPW IVR_IPW_CI STEPS_n STEPS_Age_Sex STEPS_Age_Sex_CI ///

Diff_IVR_IPW Diff_IVR_IPW_Z Diff_IVR_IPW_p Diff_IVR_IPW_CI using Table2_IVR_IPWvsSTEPS.xls, replace firstrow(variables)

order v1 IVR_n IVR_Age_Sex IVR_Age_Sex_CI STEPS_n STEPS_Age_Sex STEPS_Age_Sex_CI ///

Diff_IVR_Age_Sex

export excel v1 IVR_n IVR_Age_Sex IVR_Age_Sex_CI STEPS_n STEPS_Age_Sex STEPS_Age_Sex_CI ///

Diff_IVR_Age_Sex Diff_IVR_Age_Sex_Z Diff_IVR_Age_Sex_p Diff_IVR_Age_Sex_CI using Table2_IVR_AgeSexvsSTEPS.xls, replace firstrow(variables)

restore

x

********************************************************************************

*By Edu (IVR IPW vs STEPS)

*************

* IPW - Up to Primary

preserve

recode file1 (1 = 0) (0 = 1)

keep if school_n == 0

svyset [pweight= weight_ipw]

gen file2 = file1

gen file3 = file1

gen file4 = file1

gen file5 = file1

gen file6 = file1

gen file7 = file1

gen file8 = file1

gen file9 = file1

gen file10 = file1

gen file11 = file1

gen file12 = file1

svy: glm current_tobacco_any file1, fam(bin) link(identity)

estimates store I1

svy: glm current_smokeless_n file2, fam(bin) link(identity)

estimates store I2

svy: glm daily_smokeless_n file3, fam(bin) link(identity)

estimates store I3

svy: glm current_smoke_n file4, fam(bin) link(identity)

estimates store I4

svy: glm daily_smoke_n file5, fam(bin) link(identity)

estimates store I5

svy: glm alcohol_current file6, fam(bin) link(identity)

estimates store I6

svy: glm atelessthan5 file7, fam(bin) link(identity)

estimates store I7

svy: glm no_fruit_veg file8, fam(bin) link(identity)

estimates store I8

svy: glm salt_eat_n file9, fam(bin) link(identity)

estimates store I9

svy: glm salt_pack_n file10, fam(bin) link(identity)

estimates store I10

svy: glm htn_known file11, fam(bin) link(identity)

estimates store I11

svy: glm htn_med file12, fam(bin) link(identity)

estimates store I12

estout I1 I2 I3 I4 I5 I6 I7 I8 I9 I10 I11 I12 using Table_RD.xls, ///

cells ("b(star fmt(1)) ci(par fmt(1) label(95% CI)) z(fmt(2)) p (fmt(4)) ") replace transform(@*100)

import delimited "Table_RD.xls", clear

replace v2 = v6 if v2 == ""

replace v2 = v10 if v2 == ""

replace v2 = v14 if v2 == ""

replace v2 = v18 if v2 == ""

replace v2 = v22 if v2 == ""

replace v2 = v26 if v2 == ""

replace v2 = v30 if v2 == ""

replace v2 = v34 if v2 == ""

replace v2 = v38 if v2 == ""

replace v2 = v42 if v2 == ""

replace v2 = v46 if v2 == ""

replace v3 = v7 if v3 == ""

replace v3 = v11 if v3 == ""

replace v3 = v15 if v3 == ""

replace v3 = v19 if v3 == ""

replace v3 = v23 if v3 == ""

replace v3 = v27 if v3 == ""

replace v3 = v31 if v3 == ""

replace v3 = v35 if v3 == ""

replace v3 = v39 if v3 == ""

replace v3 = v43 if v3 == ""

replace v3 = v47 if v3 == ""

replace v4 = v8 if v4 == ""

replace v4 = v12 if v4 == ""

replace v4 = v16 if v4 == ""

replace v4 = v20 if v4 == ""

replace v4 = v24 if v4 == ""

replace v4 = v28 if v4 == ""

replace v4 = v32 if v4 == ""

replace v4 = v36 if v4 == ""

replace v4 = v40 if v4 == ""

replace v4 = v44 if v4 == ""

replace v4 = v48 if v4 == ""

replace v5 = v9 if v5 == ""

replace v5 = v13 if v5 == ""

replace v5 = v17 if v5 == ""

replace v5 = v21 if v5 == ""

replace v5 = v25 if v5 == ""

replace v5 = v29 if v5 == ""

replace v5 = v33 if v5 == ""

replace v5 = v37 if v5 == ""

replace v5 = v41 if v5 == ""

replace v5 = v45 if v5 == ""

replace v5 = v49 if v5 == ""

keep v1 v2 v3 v4 v5

drop if v1 == ""

drop if v1 == "main"

drop if v1 == "_cons"

destring v1, replace ignore(file)

rename v2 Diff_IVR_IPW

rename v3 Diff_IVR_IPW_CI

rename v4 Diff_IVR_IPW_Z

rename v5 Diff_IVR_IPW_p

gen number = v1

tostring v1, replace

replace v1 = "Any current tobacco user" if v1 == "1"

replace v1 = "Current smokeless tobacco user" if v1 == "2"

replace v1 = "Daily smokeless tobacco user" if v1 == "3"

replace v1 = "Current smoker" if v1 == "4"

replace v1 = "Daily smoker" if v1 == "5"

replace v1 = "Alcohol past month" if v1 == "6"

replace v1 = "<5 servings of fruits-veg in a day " if v1 == "7"

replace v1 = "No fruit or vegetables" if v1 == "8"

replace v1 = "Add salt to food while eating" if v1 == "9"

replace v1 = "Processed food high in salt" if v1 == "10"

replace v1 = "Known raised BP/HTN" if v1 == "11"

replace v1 = "Take medication for BP/HTN" if v1 == "12"

export excel File_RD_IVR_IPW_Edu0, replace firstrow(variables)

restore

********

* IPW - Primary to sec

preserve

recode file1 (1 = 0) (0 = 1)

keep if school_n == 1

svyset [pweight= weight_ipw]

gen file2 = file1

gen file3 = file1

gen file4 = file1

gen file5 = file1

gen file6 = file1

gen file7 = file1

gen file8 = file1

gen file9 = file1

gen file10 = file1

gen file11 = file1

gen file12 = file1

svy: glm current_tobacco_any file1, fam(bin) link(identity)

estimates store I1

svy: glm current_smokeless_n file2, fam(bin) link(identity)

estimates store I2

svy: glm daily_smokeless_n file3, fam(bin) link(identity)

estimates store I3

svy: glm current_smoke_n file4, fam(bin) link(identity)

estimates store I4

svy: glm daily_smoke_n file5, fam(bin) link(identity)

estimates store I5

svy: glm alcohol_current file6, fam(bin) link(identity)

estimates store I6

svy: glm atelessthan5 file7, fam(bin) link(identity)

estimates store I7

svy: glm no_fruit_veg file8, fam(bin) link(identity)

estimates store I8

svy: glm salt_eat_n file9, fam(bin) link(identity)

estimates store I9

svy: glm salt_pack_n file10, fam(bin) link(identity)

estimates store I10

svy: glm htn_known file11, fam(bin) link(identity)

estimates store I11

svy: glm htn_med file12, fam(bin) link(identity)

estimates store I12

estout I1 I2 I3 I4 I5 I6 I7 I8 I9 I10 I11 I12 using Table_RD.xls, ///

cells ("b(star fmt(1)) ci(par fmt(1) label(95% CI)) z(fmt(2)) p (fmt(4)) ") replace transform(@*100)

import delimited "Table_RD.xls", clear

replace v2 = v6 if v2 == ""

replace v2 = v10 if v2 == ""

replace v2 = v14 if v2 == ""

replace v2 = v18 if v2 == ""

replace v2 = v22 if v2 == ""

replace v2 = v26 if v2 == ""

replace v2 = v30 if v2 == ""

replace v2 = v34 if v2 == ""

replace v2 = v38 if v2 == ""

replace v2 = v42 if v2 == ""

replace v2 = v46 if v2 == ""

replace v3 = v7 if v3 == ""

replace v3 = v11 if v3 == ""

replace v3 = v15 if v3 == ""

replace v3 = v19 if v3 == ""

replace v3 = v23 if v3 == ""

replace v3 = v27 if v3 == ""

replace v3 = v31 if v3 == ""

replace v3 = v35 if v3 == ""

replace v3 = v39 if v3 == ""

replace v3 = v43 if v3 == ""

replace v3 = v47 if v3 == ""

replace v4 = v8 if v4 == ""

replace v4 = v12 if v4 == ""

replace v4 = v16 if v4 == ""

replace v4 = v20 if v4 == ""

replace v4 = v24 if v4 == ""

replace v4 = v28 if v4 == ""

replace v4 = v32 if v4 == ""

replace v4 = v36 if v4 == ""

replace v4 = v40 if v4 == ""

replace v4 = v44 if v4 == ""

replace v4 = v48 if v4 == ""

replace v5 = v9 if v5 == ""

replace v5 = v13 if v5 == ""

replace v5 = v17 if v5 == ""

replace v5 = v21 if v5 == ""

replace v5 = v25 if v5 == ""

replace v5 = v29 if v5 == ""

replace v5 = v33 if v5 == ""

replace v5 = v37 if v5 == ""

replace v5 = v41 if v5 == ""

replace v5 = v45 if v5 == ""

replace v5 = v49 if v5 == ""

keep v1 v2 v3 v4 v5

drop if v1 == ""

drop if v1 == "main"

drop if v1 == "_cons"

destring v1, replace ignore(file)

rename v2 Diff_IVR_IPW

rename v3 Diff_IVR_IPW_CI

rename v4 Diff_IVR_IPW_Z

rename v5 Diff_IVR_IPW_p

gen number = v1

tostring v1, replace

replace v1 = "Any current tobacco user" if v1 == "1"

replace v1 = "Current smokeless tobacco user" if v1 == "2"

replace v1 = "Daily smokeless tobacco user" if v1 == "3"

replace v1 = "Current smoker" if v1 == "4"

replace v1 = "Daily smoker" if v1 == "5"

replace v1 = "Alcohol past month" if v1 == "6"

replace v1 = "<5 servings of fruits-veg in a day " if v1 == "7"

replace v1 = "No fruit or vegetables" if v1 == "8"

replace v1 = "Add salt to food while eating" if v1 == "9"

replace v1 = "Processed food high in salt" if v1 == "10"

replace v1 = "Known raised BP/HTN" if v1 == "11"

replace v1 = "Take medication for BP/HTN" if v1 == "12"

export excel File_RD_IVR_IPW_Edu1, replace firstrow(variables)

restore

********

* IPW - More than sex

preserve

recode file1 (1 = 0) (0 = 1)

keep if school_n == 2

svyset [pweight= weight_ipw]

gen file2 = file1

gen file3 = file1

gen file4 = file1

gen file5 = file1

gen file6 = file1

gen file7 = file1

gen file8 = file1

gen file9 = file1

gen file10 = file1

gen file11 = file1

gen file12 = file1

svy: glm current_tobacco_any file1, fam(bin) link(identity)

estimates store I1

svy: glm current_smokeless_n file2, fam(bin) link(identity)

estimates store I2

svy: glm daily_smokeless_n file3, fam(bin) link(identity)

estimates store I3

svy: glm current_smoke_n file4, fam(bin) link(identity)

estimates store I4

svy: glm daily_smoke_n file5, fam(bin) link(identity)

estimates store I5

svy: glm alcohol_current file6, fam(bin) link(identity)

estimates store I6

svy: glm atelessthan5 file7, fam(bin) link(identity)

estimates store I7

svy: glm no_fruit_veg file8, fam(bin) link(identity)

estimates store I8

svy: glm salt_eat_n file9, fam(bin) link(identity)

estimates store I9

svy: glm salt_pack_n file10, fam(bin) link(identity)

estimates store I10

svy: glm htn_known file11, fam(bin) link(identity)

estimates store I11

svy: glm htn_med file12, fam(bin) link(identity)

estimates store I12

estout I1 I2 I3 I4 I5 I6 I7 I8 I9 I10 I11 I12 using Table_RD.xls, ///

cells ("b(star fmt(1)) ci(par fmt(1) label(95% CI)) z(fmt(2)) p (fmt(4)) ") replace transform(@*100)

import delimited "Table_RD.xls", clear

replace v2 = v6 if v2 == ""

replace v2 = v10 if v2 == ""

replace v2 = v14 if v2 == ""

replace v2 = v18 if v2 == ""

replace v2 = v22 if v2 == ""

replace v2 = v26 if v2 == ""

replace v2 = v30 if v2 == ""

replace v2 = v34 if v2 == ""

replace v2 = v38 if v2 == ""

replace v2 = v42 if v2 == ""

replace v2 = v46 if v2 == ""

replace v3 = v7 if v3 == ""

replace v3 = v11 if v3 == ""

replace v3 = v15 if v3 == ""

replace v3 = v19 if v3 == ""

replace v3 = v23 if v3 == ""

replace v3 = v27 if v3 == ""

replace v3 = v31 if v3 == ""

replace v3 = v35 if v3 == ""

replace v3 = v39 if v3 == ""

replace v3 = v43 if v3 == ""

replace v3 = v47 if v3 == ""

replace v4 = v8 if v4 == ""

replace v4 = v12 if v4 == ""

replace v4 = v16 if v4 == ""

replace v4 = v20 if v4 == ""

replace v4 = v24 if v4 == ""

replace v4 = v28 if v4 == ""

replace v4 = v32 if v4 == ""

replace v4 = v36 if v4 == ""

replace v4 = v40 if v4 == ""

replace v4 = v44 if v4 == ""

replace v4 = v48 if v4 == ""

replace v5 = v9 if v5 == ""

replace v5 = v13 if v5 == ""

replace v5 = v17 if v5 == ""

replace v5 = v21 if v5 == ""

replace v5 = v25 if v5 == ""

replace v5 = v29 if v5 == ""

replace v5 = v33 if v5 == ""

replace v5 = v37 if v5 == ""

replace v5 = v41 if v5 == ""

replace v5 = v45 if v5 == ""

replace v5 = v49 if v5 == ""

keep v1 v2 v3 v4 v5

drop if v1 == ""

drop if v1 == "main"

drop if v1 == "_cons"

destring v1, replace ignore(file)

rename v2 Diff_IVR_IPW

rename v3 Diff_IVR_IPW_CI

rename v4 Diff_IVR_IPW_Z

rename v5 Diff_IVR_IPW_p

gen number = v1

tostring v1, replace

replace v1 = "Any current tobacco user" if v1 == "1"

replace v1 = "Current smokeless tobacco user" if v1 == "2"

replace v1 = "Daily smokeless tobacco user" if v1 == "3"

replace v1 = "Current smoker" if v1 == "4"

replace v1 = "Daily smoker" if v1 == "5"

replace v1 = "Alcohol past month" if v1 == "6"

replace v1 = "<5 servings of fruits-veg in a day " if v1 == "7"

replace v1 = "No fruit or vegetables" if v1 == "8"

replace v1 = "Add salt to food while eating" if v1 == "9"

replace v1 = "Processed food high in salt" if v1 == "10"

replace v1 = "Known raised BP/HTN" if v1 == "11"

replace v1 = "Take medication for BP/HTN" if v1 == "12"

export excel File_RD_IVR_IPW_Edu2, replace firstrow(variables)

restore

**************************

* Age Sex - Up to Primary

preserve

recode file1 (1 = 0) (0 = 1)

keep if school_n == 0

svyset [pweight= age_sex_w]

gen file2 = file1

gen file3 = file1

gen file4 = file1

gen file5 = file1

gen file6 = file1

gen file7 = file1

gen file8 = file1

gen file9 = file1

gen file10 = file1

gen file11 = file1

gen file12 = file1

svy: glm current_tobacco_any file1, fam(bin) link(identity)

estimates store I1

svy: glm current_smokeless_n file2, fam(bin) link(identity)

estimates store I2

svy: glm daily_smokeless_n file3, fam(bin) link(identity)

estimates store I3

svy: glm current_smoke_n file4, fam(bin) link(identity)

estimates store I4

svy: glm daily_smoke_n file5, fam(bin) link(identity)

estimates store I5

svy: glm alcohol_current file6, fam(bin) link(identity)

estimates store I6

svy: glm atelessthan5 file7, fam(bin) link(identity)

estimates store I7

svy: glm no_fruit_veg file8, fam(bin) link(identity)

estimates store I8

svy: glm salt_eat_n file9, fam(bin) link(identity)

estimates store I9

svy: glm salt_pack_n file10, fam(bin) link(identity)

estimates store I10

svy: glm htn_known file11, fam(bin) link(identity)

estimates store I11

svy: glm htn_med file12, fam(bin) link(identity)

estimates store I12

estout I1 I2 I3 I4 I5 I6 I7 I8 I9 I10 I11 I12 using Table_RD.xls, ///

cells ("b(star fmt(1)) ci(par fmt(1) label(95% CI)) z(fmt(2)) p (fmt(4)) ") replace transform(@*100)

import delimited "Table_RD.xls", clear

replace v2 = v6 if v2 == ""

replace v2 = v10 if v2 == ""

replace v2 = v14 if v2 == ""

replace v2 = v18 if v2 == ""

replace v2 = v22 if v2 == ""

replace v2 = v26 if v2 == ""

replace v2 = v30 if v2 == ""

replace v2 = v34 if v2 == ""

replace v2 = v38 if v2 == ""

replace v2 = v42 if v2 == ""

replace v2 = v46 if v2 == ""

replace v3 = v7 if v3 == ""

replace v3 = v11 if v3 == ""

replace v3 = v15 if v3 == ""

replace v3 = v19 if v3 == ""

replace v3 = v23 if v3 == ""

replace v3 = v27 if v3 == ""

replace v3 = v31 if v3 == ""

replace v3 = v35 if v3 == ""

replace v3 = v39 if v3 == ""

replace v3 = v43 if v3 == ""

replace v3 = v47 if v3 == ""

replace v4 = v8 if v4 == ""

replace v4 = v12 if v4 == ""

replace v4 = v16 if v4 == ""

replace v4 = v20 if v4 == ""

replace v4 = v24 if v4 == ""

replace v4 = v28 if v4 == ""

replace v4 = v32 if v4 == ""

replace v4 = v36 if v4 == ""

replace v4 = v40 if v4 == ""

replace v4 = v44 if v4 == ""

replace v4 = v48 if v4 == ""

replace v5 = v9 if v5 == ""

replace v5 = v13 if v5 == ""

replace v5 = v17 if v5 == ""

replace v5 = v21 if v5 == ""

replace v5 = v25 if v5 == ""

replace v5 = v29 if v5 == ""

replace v5 = v33 if v5 == ""

replace v5 = v37 if v5 == ""

replace v5 = v41 if v5 == ""

replace v5 = v45 if v5 == ""

replace v5 = v49 if v5 == ""

keep v1 v2 v3 v4 v5

drop if v1 == ""

drop if v1 == "main"

drop if v1 == "_cons"

destring v1, replace ignore(file)

rename v2 Diff_IVR_Age_Sex

rename v3 Diff_IVR_Age_Sex_CI

rename v4 Diff_IVR_Age_Sex_Z

rename v5 Diff_IVR_Age_Sex_p

gen number = v1

tostring v1, replace

replace v1 = "Any current tobacco user" if v1 == "1"

replace v1 = "Current smokeless tobacco user" if v1 == "2"

replace v1 = "Daily smokeless tobacco user" if v1 == "3"

replace v1 = "Current smoker" if v1 == "4"

replace v1 = "Daily smoker" if v1 == "5"

replace v1 = "Alcohol past month" if v1 == "6"

replace v1 = "<5 servings of fruits-veg in a day " if v1 == "7"

replace v1 = "No fruit or vegetables" if v1 == "8"

replace v1 = "Add salt to food while eating" if v1 == "9"

replace v1 = "Processed food high in salt" if v1 == "10"

replace v1 = "Known raised BP/HTN" if v1 == "11"

replace v1 = "Take medication for BP/HTN" if v1 == "12"

export excel File_RD_IVR_Age_Sex_Edu0, replace firstrow(variables)

restore

********

* Age Sex - Primary to Sec

preserve

recode file1 (1 = 0) (0 = 1)

keep if school_n == 1

svyset [pweight= age_sex_w]

gen file2 = file1

gen file3 = file1

gen file4 = file1

gen file5 = file1

gen file6 = file1

gen file7 = file1

gen file8 = file1

gen file9 = file1

gen file10 = file1

gen file11 = file1

gen file12 = file1

svy: glm current_tobacco_any file1, fam(bin) link(identity)

estimates store I1

svy: glm current_smokeless_n file2, fam(bin) link(identity)

estimates store I2

svy: glm daily_smokeless_n file3, fam(bin) link(identity)

estimates store I3

svy: glm current_smoke_n file4, fam(bin) link(identity)

estimates store I4

svy: glm daily_smoke_n file5, fam(bin) link(identity)

estimates store I5

svy: glm alcohol_current file6, fam(bin) link(identity)

estimates store I6

svy: glm atelessthan5 file7, fam(bin) link(identity)

estimates store I7

svy: glm no_fruit_veg file8, fam(bin) link(identity)

estimates store I8

svy: glm salt_eat_n file9, fam(bin) link(identity)

estimates store I9

svy: glm salt_pack_n file10, fam(bin) link(identity)

estimates store I10

svy: glm htn_known file11, fam(bin) link(identity)

estimates store I11

svy: glm htn_med file12, fam(bin) link(identity)

estimates store I12

estout I1 I2 I3 I4 I5 I6 I7 I8 I9 I10 I11 I12 using Table_RD.xls, ///

cells ("b(star fmt(1)) ci(par fmt(1) label(95% CI)) z(fmt(2)) p (fmt(4)) ") replace transform(@*100)

import delimited "Table_RD.xls", clear

replace v2 = v6 if v2 == ""

replace v2 = v10 if v2 == ""

replace v2 = v14 if v2 == ""

replace v2 = v18 if v2 == ""

replace v2 = v22 if v2 == ""

replace v2 = v26 if v2 == ""

replace v2 = v30 if v2 == ""

replace v2 = v34 if v2 == ""

replace v2 = v38 if v2 == ""

replace v2 = v42 if v2 == ""

replace v2 = v46 if v2 == ""

replace v3 = v7 if v3 == ""

replace v3 = v11 if v3 == ""

replace v3 = v15 if v3 == ""

replace v3 = v19 if v3 == ""

replace v3 = v23 if v3 == ""

replace v3 = v27 if v3 == ""

replace v3 = v31 if v3 == ""

replace v3 = v35 if v3 == ""

replace v3 = v39 if v3 == ""

replace v3 = v43 if v3 == ""

replace v3 = v47 if v3 == ""

replace v4 = v8 if v4 == ""

replace v4 = v12 if v4 == ""

replace v4 = v16 if v4 == ""

replace v4 = v20 if v4 == ""

replace v4 = v24 if v4 == ""

replace v4 = v28 if v4 == ""

replace v4 = v32 if v4 == ""

replace v4 = v36 if v4 == ""

replace v4 = v40 if v4 == ""

replace v4 = v44 if v4 == ""

replace v4 = v48 if v4 == ""

replace v5 = v9 if v5 == ""

replace v5 = v13 if v5 == ""

replace v5 = v17 if v5 == ""

replace v5 = v21 if v5 == ""

replace v5 = v25 if v5 == ""

replace v5 = v29 if v5 == ""

replace v5 = v33 if v5 == ""

replace v5 = v37 if v5 == ""

replace v5 = v41 if v5 == ""

replace v5 = v45 if v5 == ""

replace v5 = v49 if v5 == ""

keep v1 v2 v3 v4 v5

drop if v1 == ""

drop if v1 == "main"

drop if v1 == "_cons"

destring v1, replace ignore(file)

rename v2 Diff_IVR_Age_Sex

rename v3 Diff_IVR_Age_Sex_CI

rename v4 Diff_IVR_Age_Sex_Z

rename v5 Diff_IVR_Age_Sex_p

gen number = v1

tostring v1, replace

replace v1 = "Any current tobacco user" if v1 == "1"

replace v1 = "Current smokeless tobacco user" if v1 == "2"

replace v1 = "Daily smokeless tobacco user" if v1 == "3"

replace v1 = "Current smoker" if v1 == "4"

replace v1 = "Daily smoker" if v1 == "5"

replace v1 = "Alcohol past month" if v1 == "6"

replace v1 = "<5 servings of fruits-veg in a day " if v1 == "7"

replace v1 = "No fruit or vegetables" if v1 == "8"

replace v1 = "Add salt to food while eating" if v1 == "9"

replace v1 = "Processed food high in salt" if v1 == "10"

replace v1 = "Known raised BP/HTN" if v1 == "11"

replace v1 = "Take medication for BP/HTN" if v1 == "12"

export excel File_RD_IVR_Age_Sex_Edu1, replace firstrow(variables)

restore

********

* Age Sex - More than Sec

preserve

recode file1 (1 = 0) (0 = 1)

keep if school_n == 2

svyset [pweight= age_sex_w]

gen file2 = file1

gen file3 = file1

gen file4 = file1

gen file5 = file1

gen file6 = file1

gen file7 = file1

gen file8 = file1

gen file9 = file1

gen file10 = file1

gen file11 = file1

gen file12 = file1

svy: glm current_tobacco_any file1, fam(bin) link(identity)

estimates store I1

svy: glm current_smokeless_n file2, fam(bin) link(identity)

estimates store I2

svy: glm daily_smokeless_n file3, fam(bin) link(identity)

estimates store I3

svy: glm current_smoke_n file4, fam(bin) link(identity)

estimates store I4

svy: glm daily_smoke_n file5, fam(bin) link(identity)

estimates store I5

svy: glm alcohol_current file6, fam(bin) link(identity)

estimates store I6

svy: glm atelessthan5 file7, fam(bin) link(identity)

estimates store I7

svy: glm no_fruit_veg file8, fam(bin) link(identity)

estimates store I8

svy: glm salt_eat_n file9, fam(bin) link(identity)

estimates store I9

svy: glm salt_pack_n file10, fam(bin) link(identity)

estimates store I10

svy: glm htn_known file11, fam(bin) link(identity)

estimates store I11

svy: glm htn_med file12, fam(bin) link(identity)

estimates store I12

estout I1 I2 I3 I4 I5 I6 I7 I8 I9 I10 I11 I12 using Table_RD.xls, ///

cells ("b(star fmt(1)) ci(par fmt(1) label(95% CI)) z(fmt(2)) p (fmt(4)) ") replace transform(@*100)

import delimited "Table_RD.xls", clear

replace v2 = v6 if v2 == ""

replace v2 = v10 if v2 == ""

replace v2 = v14 if v2 == ""

replace v2 = v18 if v2 == ""

replace v2 = v22 if v2 == ""

replace v2 = v26 if v2 == ""

replace v2 = v30 if v2 == ""

replace v2 = v34 if v2 == ""

replace v2 = v38 if v2 == ""

replace v2 = v42 if v2 == ""

replace v2 = v46 if v2 == ""

replace v3 = v7 if v3 == ""

replace v3 = v11 if v3 == ""

replace v3 = v15 if v3 == ""

replace v3 = v19 if v3 == ""

replace v3 = v23 if v3 == ""

replace v3 = v27 if v3 == ""

replace v3 = v31 if v3 == ""

replace v3 = v35 if v3 == ""

replace v3 = v39 if v3 == ""

replace v3 = v43 if v3 == ""

replace v3 = v47 if v3 == ""

replace v4 = v8 if v4 == ""

replace v4 = v12 if v4 == ""

replace v4 = v16 if v4 == ""

replace v4 = v20 if v4 == ""

replace v4 = v24 if v4 == ""

replace v4 = v28 if v4 == ""

replace v4 = v32 if v4 == ""

replace v4 = v36 if v4 == ""

replace v4 = v40 if v4 == ""

replace v4 = v44 if v4 == ""

replace v4 = v48 if v4 == ""

replace v5 = v9 if v5 == ""

replace v5 = v13 if v5 == ""

replace v5 = v17 if v5 == ""

replace v5 = v21 if v5 == ""

replace v5 = v25 if v5 == ""

replace v5 = v29 if v5 == ""

replace v5 = v33 if v5 == ""

replace v5 = v37 if v5 == ""

replace v5 = v41 if v5 == ""

replace v5 = v45 if v5 == ""

replace v5 = v49 if v5 == ""

keep v1 v2 v3 v4 v5

drop if v1 == ""

drop if v1 == "main"

drop if v1 == "_cons"

destring v1, replace ignore(file)

rename v2 Diff_IVR_Age_Sex

rename v3 Diff_IVR_Age_Sex_CI

rename v4 Diff_IVR_Age_Sex_Z

rename v5 Diff_IVR_Age_Sex_p

gen number = v1

tostring v1, replace

replace v1 = "Any current tobacco user" if v1 == "1"

replace v1 = "Current smokeless tobacco user" if v1 == "2"

replace v1 = "Daily smokeless tobacco user" if v1 == "3"

replace v1 = "Current smoker" if v1 == "4"

replace v1 = "Daily smoker" if v1 == "5"

replace v1 = "Alcohol past month" if v1 == "6"

replace v1 = "<5 servings of fruits-veg in a day " if v1 == "7"

replace v1 = "No fruit or vegetables" if v1 == "8"

replace v1 = "Add salt to food while eating" if v1 == "9"

replace v1 = "Processed food high in salt" if v1 == "10"

replace v1 = "Known raised BP/HTN" if v1 == "11"

replace v1 = "Take medication for BP/HTN" if v1 == "12"

export excel File_RD_IVR_Age_Sex_Edu2, replace firstrow(variables)

restore

********************************************************************************

*SummaryTable_LSVC

********

preserve

svyset [pweight= weight_ipw]

tabout current_tobacco_any current_smoke_n current_smokeless_n ///

daily_tobacco_any daily_smoke_n daily_smokeless_n current_tobacco_any ///

smoke_only smokeless_only both_smoke_smokeless ///

alcohol_current no_fruit_veg no_fruit no_veg ///

atelessthan5 salt_eat_n salt_pack_n ///

htn_known htn_med file1 ///

using Table3_Weight_IPW.xls, ///

c(col ci) f(1 1) percent svy replace

import delimited using Table3_Weight_IPW.xls, clear

drop v6 v7

drop if v1 == "No" | v1 == "Total"

replace v1 = v1[_n - 1] if v1 == "Yes"

drop if v2 == ""

drop if v1 == ""

rename v2 IVR_IPW

rename v4 STEPS_IPW

rename v3 IVR_IPW_CI

rename v5 STEPS_IPW_CI

gen id = _n

save Table_Weighted_LSVC_IPW.dta, replace

restore

********

preserve

svyset [pweight= age_sex_w]

tabout current_tobacco_any current_smoke_n current_smokeless_n ///

daily_tobacco_any daily_smoke_n daily_smokeless_n current_tobacco_any ///

smoke_only smokeless_only both_smoke_smokeless ///

alcohol_current no_fruit_veg no_fruit no_veg ///

atelessthan5 salt_eat_n salt_pack_n ///

htn_known htn_med file1 ///

using Table3_Weight_Age_Sex.xls, ///

c(col ci) f(1 1) percent svy replace

import delimited using Table3_Weight_Age_Sex.xls, clear

drop v6 v7

drop if v1 == "No" | v1 == "Total"

replace v1 = v1[_n - 1] if v1 == "Yes"

drop if v2 == ""

drop if v1 == ""

rename v2 IVR_Age_Sex

rename v4 STEPS_Age_Sex

rename v3 IVR_Age_Sex_CI

rename v5 STEPS_Age_Sex_CI

gen id = _n

save Table_Weighted_LSVC_Age_Sex.dta, replace

restore

*Finding Prev Differences - IPW

preserve

recode file1 (1 = 0) (0 = 1)

svyset [pweight= weight_ipw]

gen file2 = file1

gen file3 = file1

gen file4 = file1

gen file5 = file1

gen file6 = file1

gen file7 = file1

gen file8 = file1

gen file9 = file1

gen file10 = file1

gen file11 = file1

gen file12 = file1

gen file13 = file1

gen file14 = file1

gen file15 = file1

gen file16 = file1

gen file17 = file1

gen file18 = file1

gen file19 = file1

svy: glm current_tobacco_any file1, fam(bin) link(identity)

estimates store I1

svy: glm current_smoke_n file2, fam(bin) link(identity)

estimates store I2

svy: glm current_smokeless_n file3, fam(bin) link(identity)

estimates store I3

svy: glm daily_tobacco_any file4, fam(bin) link(identity)

estimates store I4

svy: glm daily_smoke_n file5, fam(bin) link(identity)

estimates store I5

svy: glm daily_smokeless_n file6, fam(bin) link(identity)

estimates store I6

svy: glm current_tobacco_any file7, fam(bin) link(identity)

estimates store I7

svy: glm smoke_only file8, fam(bin) link(identity)

estimates store I8

svy: glm smokeless_only file9, fam(bin) link(identity)

estimates store I9

svy: glm both_smoke_smokeless file10, fam(bin) link(identity)

estimates store I10

svy: glm alcohol_current file11, fam(bin) link(identity)

estimates store I11

svy: glm atelessthan5 file12, fam(bin) link(identity)

estimates store I12

svy: glm no_fruit_veg file13, fam(bin) link(identity)

estimates store I13

svy: glm no_fruit file14, fam(bin) link(identity)

estimates store I14

svy: glm no_veg file15, fam(bin) link(identity)

estimates store I15

svy: glm salt_eat_n file16, fam(bin) link(identity)

estimates store I16

svy: glm salt_pack_n file17, fam(bin) link(identity)

estimates store I17

svy: glm htn_known file18, fam(bin) link(identity)

estimates store I18

svy: glm htn_med file19, fam(bin) link(identity)

estimates store I19

estout I1 I2 I3 I4 I5 I6 I7 I8 I9 I10 I11 I12 I13 I14 I15 I16 I17 I18 I19 using Table_RD.xls, ///

cells ("b(star fmt(1)) ci(par fmt(1) label(95% CI)) p (fmt(4)) ") replace transform(@*100)

import delimited "Table_RD.xls", clear

replace v2 = v5 if v2 == ""

replace v2 = v8 if v2 == ""

replace v2 = v11 if v2 == ""

replace v2 = v14 if v2 == ""

replace v2 = v17 if v2 == ""

replace v2 = v20 if v2 == ""

replace v2 = v23 if v2 == ""

replace v2 = v26 if v2 == ""

replace v2 = v29 if v2 == ""

replace v2 = v32 if v2 == ""

replace v2 = v35 if v2 == ""

replace v2 = v38 if v2 == ""

replace v2 = v41 if v2 == ""

replace v2 = v44 if v2 == ""

replace v2 = v47 if v2 == ""

replace v2 = v50 if v2 == ""

replace v2 = v53 if v2 == ""

replace v2 = v56 if v2 == ""

replace v3 = v6 if v3 == ""

replace v3 = v9 if v3 == ""

replace v3 = v12 if v3 == ""

replace v3 = v15 if v3 == ""

replace v3 = v18 if v3 == ""

replace v3 = v21 if v3 == ""

replace v3 = v24 if v3 == ""

replace v3 = v27 if v3 == ""

replace v3 = v30 if v3 == ""

replace v3 = v33 if v3 == ""

replace v3 = v36 if v3 == ""

replace v3 = v39 if v3 == ""

replace v3 = v42 if v3 == ""

replace v3 = v45 if v3 == ""

replace v3 = v48 if v3 == ""

replace v3 = v51 if v3 == ""

replace v3 = v54 if v3 == ""

replace v3 = v57 if v3 == ""

replace v4 = v7 if v4 == ""

replace v4 = v10 if v4 == ""

replace v4 = v13 if v4 == ""

replace v4 = v16 if v4 == ""

replace v4 = v19 if v4 == ""

replace v4 = v22 if v4 == ""

replace v4 = v25 if v4 == ""

replace v4 = v28 if v4 == ""

replace v4 = v31 if v4 == ""

replace v4 = v34 if v4 == ""

replace v4 = v37 if v4 == ""

replace v4 = v40 if v4 == ""

replace v4 = v43 if v4 == ""

replace v4 = v46 if v4 == ""

replace v4 = v49 if v4 == ""

replace v4 = v52 if v4 == ""

replace v4 = v55 if v4 == ""

replace v4 = v58 if v4 == ""

keep v1 v2 v3 v4

drop if v1 == ""

drop if v1 == "main"

drop if v1 == "_cons"

destring v1, ignore(file) gen(id)

*rename v1 indicator

rename v2 Diff_IVR_IPW

rename v3 Diff_IVR_IPW_CI

rename v4 Diff_IVR_IPW_P

save File_RD_IVR_LSVC_IPW, replace

restore

*Finding Prev Differences - Age Sex

preserve

recode file1 (1 = 0) (0 = 1)

svyset [pweight= age_sex_w]

gen file2 = file1

gen file3 = file1

gen file4 = file1

gen file5 = file1

gen file6 = file1

gen file7 = file1

gen file8 = file1

gen file9 = file1

gen file10 = file1

gen file11 = file1

gen file12 = file1

gen file13 = file1

gen file14 = file1

gen file15 = file1

gen file16 = file1

gen file17 = file1

gen file18 = file1

gen file19 = file1

svy: glm current_tobacco_any file1, fam(bin) link(identity)

estimates store I1

svy: glm current_smoke_n file2, fam(bin) link(identity)

estimates store I2

svy: glm current_smokeless_n file3, fam(bin) link(identity)

estimates store I3

svy: glm daily_tobacco_any file4, fam(bin) link(identity)

estimates store I4

svy: glm daily_smoke_n file5, fam(bin) link(identity)

estimates store I5

svy: glm daily_smokeless_n file6, fam(bin) link(identity)

estimates store I6

svy: glm current_tobacco_any file7, fam(bin) link(identity)

estimates store I7

svy: glm smoke_only file8, fam(bin) link(identity)

estimates store I8

svy: glm smokeless_only file9, fam(bin) link(identity)

estimates store I9

svy: glm both_smoke_smokeless file10, fam(bin) link(identity)

estimates store I10

svy: glm alcohol_current file11, fam(bin) link(identity)

estimates store I11

svy: glm atelessthan5 file12, fam(bin) link(identity)

estimates store I12

svy: glm no_fruit_veg file13, fam(bin) link(identity)

estimates store I13

svy: glm no_fruit file14, fam(bin) link(identity)

estimates store I14

svy: glm no_veg file15, fam(bin) link(identity)

estimates store I15

svy: glm salt_eat_n file16, fam(bin) link(identity)

estimates store I16

svy: glm salt_pack_n file17, fam(bin) link(identity)

estimates store I17

svy: glm htn_known file18, fam(bin) link(identity)

estimates store I18

svy: glm htn_med file19, fam(bin) link(identity)

estimates store I19

estout I1 I2 I3 I4 I5 I6 I7 I8 I9 I10 I11 I12 I13 I14 I15 I16 I17 I18 I19 using Table_RD.xls, ///

cells ("b(star fmt(1)) ci(par fmt(1) label(95% CI)) p (fmt(4)) ") replace transform(@*100)

import delimited "Table_RD.xls", clear

replace v2 = v5 if v2 == ""

replace v2 = v8 if v2 == ""

replace v2 = v11 if v2 == ""

replace v2 = v14 if v2 == ""

replace v2 = v17 if v2 == ""

replace v2 = v20 if v2 == ""

replace v2 = v23 if v2 == ""

replace v2 = v26 if v2 == ""

replace v2 = v29 if v2 == ""

replace v2 = v32 if v2 == ""

replace v2 = v35 if v2 == ""

replace v2 = v38 if v2 == ""

replace v2 = v41 if v2 == ""

replace v2 = v44 if v2 == ""

replace v2 = v47 if v2 == ""

replace v2 = v50 if v2 == ""

replace v2 = v53 if v2 == ""

replace v2 = v56 if v2 == ""

replace v3 = v6 if v3 == ""

replace v3 = v9 if v3 == ""

replace v3 = v12 if v3 == ""

replace v3 = v15 if v3 == ""

replace v3 = v18 if v3 == ""

replace v3 = v21 if v3 == ""

replace v3 = v24 if v3 == ""

replace v3 = v27 if v3 == ""

replace v3 = v30 if v3 == ""

replace v3 = v33 if v3 == ""

replace v3 = v36 if v3 == ""

replace v3 = v39 if v3 == ""

replace v3 = v42 if v3 == ""

replace v3 = v45 if v3 == ""

replace v3 = v48 if v3 == ""

replace v3 = v51 if v3 == ""

replace v3 = v54 if v3 == ""

replace v3 = v57 if v3 == ""

replace v4 = v7 if v4 == ""

replace v4 = v10 if v4 == ""

replace v4 = v13 if v4 == ""

replace v4 = v16 if v4 == ""

replace v4 = v19 if v4 == ""

replace v4 = v22 if v4 == ""

replace v4 = v25 if v4 == ""

replace v4 = v28 if v4 == ""

replace v4 = v31 if v4 == ""

replace v4 = v34 if v4 == ""

replace v4 = v37 if v4 == ""

replace v4 = v40 if v4 == ""

replace v4 = v43 if v4 == ""

replace v4 = v46 if v4 == ""

replace v4 = v49 if v4 == ""

replace v4 = v52 if v4 == ""

replace v4 = v55 if v4 == ""

replace v4 = v58 if v4 == ""

keep v1 v2 v3 v4

drop if v1 == ""

drop if v1 == "main"

drop if v1 == "_cons"

destring v1, ignore(file) gen(id)

*rename v1 indicator

rename v2 Diff_IVR_Age_Sex

rename v3 Diff_IVR_Age_Sex_CI

rename v4 Diff_IVR_Age_Sex_P

save File_RD_IVR_LSVC_Age_Sex, replace

restore

preserve

use Table_Weighted_LSVC_IPW.dta

merge 1:1 id using File_RD_IVR_LSVC_IPW, nogen

merge 1:1 id using Table_Weighted_LSVC_Age_Sex.dta, nogen

merge 1:1 id using File_RD_IVR_LSVC_Age_Sex, nogen

export excel FIle_LSVC_IVR.xls, replace firstrow(variables)

restore

********************************************************************************

preserve

keep if age_cat == 0

tabout current_tobacco_any current_smoke_n current_smokeless_n ///

daily_tobacco_any daily_smoke_n daily_smokeless_n ///

smoke_only smokeless_only both_smoke_smokeless ///

alcohol_current no_fruit_veg no_fruit no_veg ///

atelessthan5 salt_eat_n salt_pack_n ///

htn_known htn_med file1 ///

using Table3_n.xls, ///

c(freq col) f(1c 1p) replace

import delimited using Table3_n.xls, clear

drop if v1 == "No"

drop if v1 == "Yes"

replace v1 = v1[_n - 1] if v1 == "Total"

drop v3 v5 v6 v7

drop if v1 == ""

drop if v2 == ""

destring v2 v4, ignore(",") replace

rename v2 IVR_n

rename v4 STEPS_n

gen id = _n

save Table3_n.dta, replace

restore

preserve

keep if age_cat == 0

gen wt = 1

svyset [pweight= wt]

tabout current_tobacco_any current_smoke_n current_smokeless_n ///

daily_tobacco_any daily_smoke_n daily_smokeless_n ///

smoke_only smokeless_only both_smoke_smokeless ///

alcohol_current no_fruit_veg no_fruit no_veg ///

atelessthan5 salt_eat_n salt_pack_n ///

htn_known htn_med file1 ///

using Table3_Unweight.xls, ///

c(col ci) f(1 1) percent svy replace

import delimited using Table3_Unweight.xls, clear

drop v6 v7

drop if v1 == "No" | v1 == "Total"

replace v1 = v1[_n - 1] if v1 == "Yes"

drop if v2 == ""

drop if v1 == ""

rename v2 IVR_Unweight

rename v4 STEPS_Unweight

rename v3 IVR_Unweight_CI

rename v5 STEPS_Unweight_CI

gen id = _n

save Table3_Unweighted.dta, replace

restore

********

preserve

keep if age_cat == 0

svyset [pweight= weight_ipw]

tabout current_tobacco_any current_smoke_n current_smokeless_n ///

daily_tobacco_any daily_smoke_n daily_smokeless_n ///

smoke_only smokeless_only both_smoke_smokeless ///

alcohol_current no_fruit_veg no_fruit no_veg ///

atelessthan5 salt_eat_n salt_pack_n ///

htn_known htn_med file1 ///

using Table3_Weight_IPW.xls, ///

c(col ci) f(1 1) percent svy replace

import delimited using Table3_Weight_IPW.xls, clear

drop v6 v7

drop if v1 == "No" | v1 == "Total"

replace v1 = v1[_n - 1] if v1 == "Yes"

drop if v2 == ""

drop if v1 == ""

rename v2 IVR_IPW

rename v4 STEPS_IPW

rename v3 IVR_IPW_CI

rename v5 STEPS_IPW_CI

gen id = _n

save Table3_Weighted_IPW.dta, replace

restore

********

preserve

keep if age_cat == 0

svyset [pweight= age_sex_w]

tabout current_tobacco_any current_smoke_n current_smokeless_n ///

daily_tobacco_any daily_smoke_n daily_smokeless_n ///

smoke_only smokeless_only both_smoke_smokeless ///

alcohol_current no_fruit_veg no_fruit no_veg ///

atelessthan5 salt_eat_n salt_pack_n ///

htn_known htn_med file1 ///

using Table3_Weight_Age_Sex_W.xls, ///

c(col ci) f(1 1) percent svy replace

import delimited using Table3_Weight_Age_Sex_W.xls, clear

drop v6 v7

drop if v1 == "No" | v1 == "Total"

replace v1 = v1[_n - 1] if v1 == "Yes"

drop if v2 == ""

drop if v1 == ""

rename v2 IVR_Age_Sex

rename v4 STEPS_Age_Sex

rename v3 IVR_Age_Sex_CI

rename v5 STEPS_Age_Sex_CI

gen id = _n

save Table3_Weighted_Age_Sex_W.dta, replace

restore

***

preserve

recode file1 (1 = 0) (0 = 1)

keep if age_cat == 0

svyset [pweight= weight_ipw]

gen file2 = file1

gen file3 = file1

gen file4 = file1

gen file5 = file1

gen file6 = file1

gen file7 = file1

gen file8 = file1

gen file9 = file1

gen file10 = file1

gen file11 = file1

gen file12 = file1

gen file13 = file1

gen file14 = file1

gen file15 = file1

gen file16 = file1

gen file17 = file1

gen file18 = file1

svy: glm current_tobacco_any file1, fam(bin) link(identity)

estimates store I1

svy: glm current_smoke_n file2, fam(bin) link(identity)

estimates store I2

svy: glm current_smokeless_n file3, fam(bin) link(identity)

estimates store I3

svy: glm daily_tobacco_any file4, fam(bin) link(identity)

estimates store I4

svy: glm daily_smoke_n file5, fam(bin) link(identity)

estimates store I5

svy: glm daily_smokeless_n file6, fam(bin) link(identity)

estimates store I6

svy: glm smoke_only file7, fam(bin) link(identity)

estimates store I7

svy: glm smokeless_only file8, fam(bin) link(identity)

estimates store I8

svy: glm both_smoke_smokeless file9, fam(bin) link(identity)

estimates store I9

svy: glm alcohol_current file10, fam(bin) link(identity)

estimates store I10

svy: glm no_fruit_veg file11, fam(bin) link(identity)

estimates store I11

svy: glm no_fruit file12, fam(bin) link(identity)

estimates store I12

svy: glm no_veg file13, fam(bin) link(identity)

estimates store I13

svy: glm atelessthan5 file14, fam(bin) link(identity)

estimates store I14

svy: glm salt_eat_n file15, fam(bin) link(identity)

estimates store I15

svy: glm salt_pack_n file16, fam(bin) link(identity)

estimates store I16

svy: glm htn_known file17, fam(bin) link(identity)

estimates store I17

svy: glm htn_med file18, fam(bin) link(identity)

estimates store I18

estout I1 I2 I3 I4 I5 I6 I7 I8 I9 I10 I11 I12 I13 I14 I15 I16 I17 I18 using Table_RD.xls, ///

cells ("b(star fmt(1)) ci(par fmt(1) label(95% CI)) p (fmt(4)) ") replace transform(@*100)

import delimited "Table_RD.xls", clear

replace v2 = v5 if v2 == ""

replace v2 = v8 if v2 == ""

replace v2 = v11 if v2 == ""

replace v2 = v14 if v2 == ""

replace v2 = v17 if v2 == ""

replace v2 = v20 if v2 == ""

replace v2 = v23 if v2 == ""

replace v2 = v26 if v2 == ""

replace v2 = v29 if v2 == ""

replace v2 = v32 if v2 == ""

replace v2 = v35 if v2 == ""

replace v2 = v38 if v2 == ""

replace v2 = v41 if v2 == ""

replace v2 = v44 if v2 == ""

replace v2 = v47 if v2 == ""

replace v2 = v50 if v2 == ""

replace v2 = v53 if v2 == ""

replace v3 = v6 if v3 == ""

replace v3 = v9 if v3 == ""

replace v3 = v12 if v3 == ""

replace v3 = v15 if v3 == ""

replace v3 = v18 if v3 == ""

replace v3 = v21 if v3 == ""

replace v3 = v24 if v3 == ""

replace v3 = v27 if v3 == ""

replace v3 = v30 if v3 == ""

replace v3 = v33 if v3 == ""

replace v3 = v36 if v3 == ""

replace v3 = v39 if v3 == ""

replace v3 = v42 if v3 == ""

replace v3 = v45 if v3 == ""

replace v3 = v48 if v3 == ""

replace v3 = v51 if v3 == ""

replace v3 = v54 if v3 == ""

replace v4 = v7 if v4 == ""

replace v4 = v10 if v4 == ""

replace v4 = v13 if v4 == ""

replace v4 = v16 if v4 == ""

replace v4 = v19 if v4 == ""

replace v4 = v22 if v4 == ""

replace v4 = v25 if v4 == ""

replace v4 = v28 if v4 == ""

replace v4 = v31 if v4 == ""

replace v4 = v34 if v4 == ""

replace v4 = v37 if v4 == ""

replace v4 = v40 if v4 == ""

replace v4 = v43 if v4 == ""

replace v4 = v46 if v4 == ""

replace v4 = v49 if v4 == ""

replace v4 = v52 if v4 == ""

replace v4 = v55 if v4 == ""

keep v1 v2 v3 v4

drop if v1 == ""

drop if v1 == "main"

drop if v1 == "_cons"

destring v1, replace ignore(file)

rename v2 Diff_IVR_IPW

rename v3 Diff_IVR_IPW_CI

rename v4 Diff_IVR_IPW_P

gen pop_group = 1

tostring v1, replace

replace v1 = "Any current tobacco user" if v1 == "1"

replace v1 = "Current smoker" if v1 == "2"

replace v1 = "Current smokeless tobacco user" if v1 == "3"

replace v1 = "Any daily tobacco user" if v1 == "4"

replace v1 = "Daily smoker" if v1 == "5"

replace v1 = "Daily smokeless tobacco user" if v1 == "6"

replace v1 = "Only smoker" if v1 == "7"

replace v1 = "Only smokeless tobacco user" if v1 == "8"

replace v1 = "Both smoker and smokeless user" if v1 == "9"

replace v1 = "Alcohol past month" if v1 == "10"

replace v1 = "No fruit or vegetables" if v1 == "11"

replace v1 = "No fruit" if v1 == "12"

replace v1 = "No veg" if v1 == "13"

replace v1 = "<5 servings of fruits-veg in a day " if v1 == "14"

replace v1 = "Add salt to food while eating" if v1 == "15"

replace v1 = "Processed food high in salt" if v1 == "16"

replace v1 = "Known raised BP/HTN" if v1 == "17"

replace v1 = "Take medication for BP/HTN" if v1 == "18"

save File_RD_IVR_IPW, replace

restore

********

preserve

recode file1 (1 = 0) (0 = 1)

keep if age_cat == 0

svyset [pweight= age_sex_w]

gen file2 = file1

gen file3 = file1

gen file4 = file1

gen file5 = file1

gen file6 = file1

gen file7 = file1

gen file8 = file1

gen file9 = file1

gen file10 = file1

gen file11 = file1

gen file12 = file1

gen file13 = file1

gen file14 = file1

gen file15 = file1

gen file16 = file1

gen file17 = file1

gen file18 = file1

svy: glm current_tobacco_any file1, fam(bin) link(identity)

estimates store I1

svy: glm current_smoke_n file2, fam(bin) link(identity)

estimates store I2

svy: glm current_smokeless_n file3, fam(bin) link(identity)

estimates store I3

svy: glm daily_tobacco_any file4, fam(bin) link(identity)

estimates store I4

svy: glm daily_smoke_n file5, fam(bin) link(identity)

estimates store I5

svy: glm daily_smokeless_n file6, fam(bin) link(identity)

estimates store I6

svy: glm smoke_only file7, fam(bin) link(identity)

estimates store I7

svy: glm smokeless_only file8, fam(bin) link(identity)

estimates store I8

svy: glm both_smoke_smokeless file9, fam(bin) link(identity)

estimates store I9

svy: glm alcohol_current file10, fam(bin) link(identity)

estimates store I10

svy: glm no_fruit_veg file11, fam(bin) link(identity)

estimates store I11

svy: glm no_fruit file12, fam(bin) link(identity)

estimates store I12

svy: glm no_veg file13, fam(bin) link(identity)

estimates store I13

svy: glm atelessthan5 file14, fam(bin) link(identity)

estimates store I14

svy: glm salt_eat_n file15, fam(bin) link(identity)

estimates store I15

svy: glm salt_pack_n file16, fam(bin) link(identity)

estimates store I16

svy: glm htn_known file17, fam(bin) link(identity)

estimates store I17

svy: glm htn_med file18, fam(bin) link(identity)

estimates store I18

estout I1 I2 I3 I4 I5 I6 I7 I8 I9 I10 I11 I12 I13 I14 I15 I16 I17 I18 using Table_RD.xls, ///

cells ("b(star fmt(1)) ci(par fmt(1) label(95% CI)) p (fmt(4)) ") replace transform(@*100)

import delimited "Table_RD.xls", clear

replace v2 = v5 if v2 == ""

replace v2 = v8 if v2 == ""

replace v2 = v11 if v2 == ""

replace v2 = v14 if v2 == ""

replace v2 = v17 if v2 == ""

replace v2 = v20 if v2 == ""

replace v2 = v23 if v2 == ""

replace v2 = v26 if v2 == ""

replace v2 = v29 if v2 == ""

replace v2 = v32 if v2 == ""

replace v2 = v35 if v2 == ""

replace v2 = v38 if v2 == ""

replace v2 = v41 if v2 == ""

replace v2 = v44 if v2 == ""

replace v2 = v47 if v2 == ""

replace v2 = v50 if v2 == ""

replace v2 = v53 if v2 == ""

replace v3 = v6 if v3 == ""

replace v3 = v9 if v3 == ""

replace v3 = v12 if v3 == ""

replace v3 = v15 if v3 == ""

replace v3 = v18 if v3 == ""

replace v3 = v21 if v3 == ""

replace v3 = v24 if v3 == ""

replace v3 = v27 if v3 == ""

replace v3 = v30 if v3 == ""

replace v3 = v33 if v3 == ""

replace v3 = v36 if v3 == ""

replace v3 = v39 if v3 == ""

replace v3 = v42 if v3 == ""

replace v3 = v45 if v3 == ""

replace v3 = v48 if v3 == ""

replace v3 = v51 if v3 == ""

replace v3 = v54 if v3 == ""

replace v4 = v7 if v4 == ""

replace v4 = v10 if v4 == ""

replace v4 = v13 if v4 == ""

replace v4 = v16 if v4 == ""

replace v4 = v19 if v4 == ""

replace v4 = v22 if v4 == ""

replace v4 = v25 if v4 == ""

replace v4 = v28 if v4 == ""

replace v4 = v31 if v4 == ""

replace v4 = v34 if v4 == ""

replace v4 = v37 if v4 == ""

replace v4 = v40 if v4 == ""

replace v4 = v43 if v4 == ""

replace v4 = v46 if v4 == ""

replace v4 = v49 if v4 == ""

replace v4 = v52 if v4 == ""

replace v4 = v55 if v4 == ""

keep v1 v2 v3 v4

drop if v1 == ""

drop if v1 == "main"

drop if v1 == "_cons"

destring v1, replace ignore(file)

rename v2 Diff_IVR_Age_Sex

rename v3 Diff_IVR_Age_Sex_CI

rename v4 Diff_IVR_Age_Sex_P

gen pop_group = 1

gen number = v1

tostring v1, replace

replace v1 = "Any current tobacco user" if v1 == "1"

replace v1 = "Current smoker" if v1 == "2"

replace v1 = "Current smokeless tobacco user" if v1 == "3"

replace v1 = "Any daily tobacco user" if v1 == "4"

replace v1 = "Daily smoker" if v1 == "5"

replace v1 = "Daily smokeless tobacco user" if v1 == "6"

replace v1 = "Only smoker" if v1 == "7"

replace v1 = "Only smokeless tobacco user" if v1 == "8"

replace v1 = "Both smoker and smokeless user" if v1 == "9"

replace v1 = "Alcohol past month" if v1 == "10"

replace v1 = "No fruit or vegetables" if v1 == "11"

replace v1 = "No fruit" if v1 == "12"

replace v1 = "No veg" if v1 == "13"

replace v1 = "<5 servings of fruits-veg in a day " if v1 == "14"

replace v1 = "Add salt to food while eating" if v1 == "15"

replace v1 = "Processed food high in salt" if v1 == "16"

replace v1 = "Known raised BP/HTN" if v1 == "17"

replace v1 = "Take medication for BP/HTN" if v1 == "18"

save File_RD_IVR_Age_Sex, replace

restore

*********

preserve

use Table3_Unweighted.dta, replace

merge 1:1 v1 using Table3_Weighted_IPW, nogen

merge 1:1 v1 using Table3_Weighted_Age_Sex_W.dta, nogen

merge 1:1 v1 using Table3_n.dta, nogen

sort id

drop id

label var v1 "Indicator"

drop STEPS_IPW

gen indicator = _n

gen pop_group = 1

save Table3_01_Prevalence_GMAK_20Jul2022.dta, replace

merge 1:1 v1 using File_RD_IVR_IPW.dta, nogen

merge 1:1 v1 using File_RD_IVR_Age_Sex.dta, nogen

sort number

order v1 IVR_n IVR_IPW IVR_IPW_CI STEPS_n STEPS_Age_Sex STEPS_Age_Sex_CI Diff_IVR_IPW

export excel v1 IVR_n IVR_IPW IVR_IPW_CI STEPS_n STEPS_Age_Sex STEPS_Age_Sex_CI ///

Diff_IVR_IPW using SumTab_IVR_IPWvsSTEPS.xls, replace

order v1 IVR_n IVR_Age_Sex IVR_Age_Sex_CI STEPS_n STEPS_Age_Sex STEPS_Age_Sex_CI ///

Diff_IVR_Age_Sex

export excel v1 IVR_n IVR_Age_Sex IVR_Age_Sex_CI STEPS_n STEPS_Age_Sex STEPS_Age_Sex_CI ///

Diff_IVR_Age_Sex using SumTab_IVR_AgeSexvsSTEPS.xls, replace

restore

********************************************************************************

preserve

keep if age_cat == 1

tabout current_tobacco_any current_smoke_n current_smokeless_n ///

daily_tobacco_any daily_smoke_n daily_smokeless_n ///

smoke_only smokeless_only both_smoke_smokeless ///

alcohol_current no_fruit_veg no_fruit no_veg ///

atelessthan5 salt_eat_n salt_pack_n ///

htn_known htn_med file1 ///

using Table3_n.xls, ///

c(freq col) f(1c 1p) replace

import delimited using Table3_n.xls, clear

drop if v1 == "No"

drop if v1 == "Yes"

replace v1 = v1[_n - 1] if v1 == "Total"

drop v3 v5 v6 v7

drop if v1 == ""

drop if v2 == ""

destring v2 v4, ignore(",") replace

rename v2 IVR_n

rename v4 STEPS_n

gen id = _n

save Table3_n.dta, replace

restore

preserve

keep if age_cat == 1

gen wt = 1

svyset [pweight= wt]

tabout current_tobacco_any current_smoke_n current_smokeless_n ///

daily_tobacco_any daily_smoke_n daily_smokeless_n ///

smoke_only smokeless_only both_smoke_smokeless ///

alcohol_current no_fruit_veg no_fruit no_veg ///

atelessthan5 salt_eat_n salt_pack_n ///

htn_known htn_med file1 ///

using Table3_Unweight.xls, ///

c(col ci) f(1 1) percent svy replace

import delimited using Table3_Unweight.xls, clear

drop v6 v7

drop if v1 == "No" | v1 == "Total"

replace v1 = v1[_n - 1] if v1 == "Yes"

drop if v2 == ""

drop if v1 == ""

rename v2 IVR_Unweight

rename v4 STEPS_Unweight

rename v3 IVR_Unweight_CI

rename v5 STEPS_Unweight_CI

gen id = _n

save Table3_Unweighted.dta, replace

restore

********

preserve

keep if age_cat == 1

svyset [pweight= weight_ipw]

tabout current_tobacco_any current_smoke_n current_smokeless_n ///

daily_tobacco_any daily_smoke_n daily_smokeless_n ///

smoke_only smokeless_only both_smoke_smokeless ///

alcohol_current no_fruit_veg no_fruit no_veg ///

atelessthan5 salt_eat_n salt_pack_n ///

htn_known htn_med file1 ///

using Table3_Weight_IPW.xls, ///

c(col ci) f(1 1) percent svy replace

import delimited using Table3_Weight_IPW.xls, clear

drop v6 v7

drop if v1 == "No" | v1 == "Total"

replace v1 = v1[_n - 1] if v1 == "Yes"

drop if v2 == ""

drop if v1 == ""

rename v2 IVR_IPW

rename v4 STEPS_IPW

rename v3 IVR_IPW_CI

rename v5 STEPS_IPW_CI

gen id = _n

save Table3_Weighted_IPW.dta, replace

restore

********

preserve

keep if age_cat == 1

svyset [pweight= age_sex_w]

tabout current_tobacco_any current_smoke_n current_smokeless_n ///

daily_tobacco_any daily_smoke_n daily_smokeless_n ///

smoke_only smokeless_only both_smoke_smokeless ///

alcohol_current no_fruit_veg no_fruit no_veg ///

atelessthan5 salt_eat_n salt_pack_n ///

htn_known htn_med file1 ///

using Table3_Weight_Age_Sex_W.xls, ///

c(col ci) f(1 1) percent svy replace

import delimited using Table3_Weight_Age_Sex_W.xls, clear

drop v6 v7

drop if v1 == "No" | v1 == "Total"

replace v1 = v1[_n - 1] if v1 == "Yes"

drop if v2 == ""

drop if v1 == ""

rename v2 IVR_Age_Sex

rename v4 STEPS_Age_Sex

rename v3 IVR_Age_Sex_CI

rename v5 STEPS_Age_Sex_CI

gen id = _n

save Table3_Weighted_Age_Sex_W.dta, replace

restore

*********

******

*

preserve

recode file1 (1 = 0) (0 = 1)

keep if age_cat == 1

svyset [pweight= weight_ipw]

gen file2 = file1

gen file3 = file1

gen file4 = file1

gen file5 = file1

gen file6 = file1

gen file7 = file1

gen file8 = file1

gen file9 = file1

gen file10 = file1

gen file11 = file1

gen file12 = file1

gen file13 = file1

gen file14 = file1

gen file15 = file1

gen file16 = file1

gen file17 = file1

gen file18 = file1

svy: glm current_tobacco_any file1, fam(bin) link(identity)

estimates store I1

svy: glm current_smoke_n file2, fam(bin) link(identity)

estimates store I2

svy: glm current_smokeless_n file3, fam(bin) link(identity)

estimates store I3

svy: glm daily_tobacco_any file4, fam(bin) link(identity)

estimates store I4

svy: glm daily_smoke_n file5, fam(bin) link(identity)

estimates store I5

svy: glm daily_smokeless_n file6, fam(bin) link(identity)

estimates store I6

svy: glm smoke_only file7, fam(bin) link(identity)

estimates store I7

svy: glm smokeless_only file8, fam(bin) link(identity)

estimates store I8

svy: glm both_smoke_smokeless file9, fam(bin) link(identity)

estimates store I9

svy: glm alcohol_current file10, fam(bin) link(identity)

estimates store I10

svy: glm no_fruit_veg file11, fam(bin) link(identity)

estimates store I11

svy: glm no_fruit file12, fam(bin) link(identity)

estimates store I12

svy: glm no_veg file13, fam(bin) link(identity)

estimates store I13

svy: glm atelessthan5 file14, fam(bin) link(identity)

estimates store I14

svy: glm salt_eat_n file15, fam(bin) link(identity)

estimates store I15

svy: glm salt_pack_n file16, fam(bin) link(identity)

estimates store I16

svy: glm htn_known file17, fam(bin) link(identity)

estimates store I17

svy: glm htn_med file18, fam(bin) link(identity)

estimates store I18

estout I1 I2 I3 I4 I5 I6 I7 I8 I9 I10 I11 I12 I13 I14 I15 I16 I17 I18 using Table_RD.xls, ///

cells ("b(star fmt(1)) ci(par fmt(1) label(95% CI)) p (fmt(4)) ") replace transform(@*100)

import delimited "Table_RD.xls", clear

replace v2 = v5 if v2 == ""

replace v2 = v8 if v2 == ""

replace v2 = v11 if v2 == ""

replace v2 = v14 if v2 == ""

replace v2 = v17 if v2 == ""

replace v2 = v20 if v2 == ""

replace v2 = v23 if v2 == ""

replace v2 = v26 if v2 == ""

replace v2 = v29 if v2 == ""

replace v2 = v32 if v2 == ""

replace v2 = v35 if v2 == ""

replace v2 = v38 if v2 == ""

replace v2 = v41 if v2 == ""

replace v2 = v44 if v2 == ""

replace v2 = v47 if v2 == ""

replace v2 = v50 if v2 == ""

replace v2 = v53 if v2 == ""

replace v3 = v6 if v3 == ""

replace v3 = v9 if v3 == ""

replace v3 = v12 if v3 == ""

replace v3 = v15 if v3 == ""

replace v3 = v18 if v3 == ""

replace v3 = v21 if v3 == ""

replace v3 = v24 if v3 == ""

replace v3 = v27 if v3 == ""

replace v3 = v30 if v3 == ""

replace v3 = v33 if v3 == ""

replace v3 = v36 if v3 == ""

replace v3 = v39 if v3 == ""

replace v3 = v42 if v3 == ""

replace v3 = v45 if v3 == ""

replace v3 = v48 if v3 == ""

replace v3 = v51 if v3 == ""

replace v3 = v54 if v3 == ""

replace v4 = v7 if v4 == ""

replace v4 = v10 if v4 == ""

replace v4 = v13 if v4 == ""

replace v4 = v16 if v4 == ""

replace v4 = v19 if v4 == ""

replace v4 = v22 if v4 == ""

replace v4 = v25 if v4 == ""

replace v4 = v28 if v4 == ""

replace v4 = v31 if v4 == ""

replace v4 = v34 if v4 == ""

replace v4 = v37 if v4 == ""

replace v4 = v40 if v4 == ""

replace v4 = v43 if v4 == ""

replace v4 = v46 if v4 == ""

replace v4 = v49 if v4 == ""

replace v4 = v52 if v4 == ""

replace v4 = v55 if v4 == ""

keep v1 v2 v3 v4

drop if v1 == ""

drop if v1 == "main"

drop if v1 == "_cons"

destring v1, replace ignore(file)

rename v2 Diff_IVR_IPW

rename v3 Diff_IVR_IPW_CI

rename v4 Diff_IVR_IPW_P

gen pop_group = 1

tostring v1, replace

replace v1 = "Any current tobacco user" if v1 == "1"

replace v1 = "Current smoker" if v1 == "2"

replace v1 = "Current smokeless tobacco user" if v1 == "3"

replace v1 = "Any daily tobacco user" if v1 == "4"

replace v1 = "Daily smoker" if v1 == "5"

replace v1 = "Daily smokeless tobacco user" if v1 == "6"

replace v1 = "Only smoker" if v1 == "7"

replace v1 = "Only smokeless tobacco user" if v1 == "8"

replace v1 = "Both smoker and smokeless user" if v1 == "9"

replace v1 = "Alcohol past month" if v1 == "10"

replace v1 = "No fruit or vegetables" if v1 == "11"

replace v1 = "No fruit" if v1 == "12"

replace v1 = "No veg" if v1 == "13"

replace v1 = "<5 servings of fruits-veg in a day " if v1 == "14"

replace v1 = "Add salt to food while eating" if v1 == "15"

replace v1 = "Processed food high in salt" if v1 == "16"

replace v1 = "Known raised BP/HTN" if v1 == "17"

replace v1 = "Take medication for BP/HTN" if v1 == "18"

save File_RD_IVR_IPW, replace

restore

********

preserve

recode file1 (1 = 0) (0 = 1)

keep if age_cat == 1

svyset [pweight= age_sex_w]

gen file2 = file1

gen file3 = file1

gen file4 = file1

gen file5 = file1

gen file6 = file1

gen file7 = file1

gen file8 = file1

gen file9 = file1

gen file10 = file1

gen file11 = file1

gen file12 = file1

gen file13 = file1

gen file14 = file1

gen file15 = file1

gen file16 = file1

gen file17 = file1

gen file18 = file1

svy: glm current_tobacco_any file1, fam(bin) link(identity)

estimates store I1

svy: glm current_smoke_n file2, fam(bin) link(identity)

estimates store I2

svy: glm current_smokeless_n file3, fam(bin) link(identity)

estimates store I3

svy: glm daily_tobacco_any file4, fam(bin) link(identity)

estimates store I4

svy: glm daily_smoke_n file5, fam(bin) link(identity)

estimates store I5

svy: glm daily_smokeless_n file6, fam(bin) link(identity)

estimates store I6

svy: glm smoke_only file7, fam(bin) link(identity)

estimates store I7

svy: glm smokeless_only file8, fam(bin) link(identity)

estimates store I8

svy: glm both_smoke_smokeless file9, fam(bin) link(identity)

estimates store I9

svy: glm alcohol_current file10, fam(bin) link(identity)

estimates store I10

svy: glm no_fruit_veg file11, fam(bin) link(identity)

estimates store I11

svy: glm no_fruit file12, fam(bin) link(identity)

estimates store I12

svy: glm no_veg file13, fam(bin) link(identity)

estimates store I13

svy: glm atelessthan5 file14, fam(bin) link(identity)

estimates store I14

svy: glm salt_eat_n file15, fam(bin) link(identity)

estimates store I15

svy: glm salt_pack_n file16, fam(bin) link(identity)

estimates store I16

svy: glm htn_known file17, fam(bin) link(identity)

estimates store I17

svy: glm htn_med file18, fam(bin) link(identity)

estimates store I18

estout I1 I2 I3 I4 I5 I6 I7 I8 I9 I10 I11 I12 I13 I14 I15 I16 I17 I18 using Table_RD.xls, ///

cells ("b(star fmt(1)) ci(par fmt(1) label(95% CI)) p (fmt(4)) ") replace transform(@*100)

import delimited "Table_RD.xls", clear

replace v2 = v5 if v2 == ""

replace v2 = v8 if v2 == ""

replace v2 = v11 if v2 == ""

replace v2 = v14 if v2 == ""

replace v2 = v17 if v2 == ""

replace v2 = v20 if v2 == ""

replace v2 = v23 if v2 == ""

replace v2 = v26 if v2 == ""

replace v2 = v29 if v2 == ""

replace v2 = v32 if v2 == ""

replace v2 = v35 if v2 == ""

replace v2 = v38 if v2 == ""

replace v2 = v41 if v2 == ""

replace v2 = v44 if v2 == ""

replace v2 = v47 if v2 == ""

replace v2 = v50 if v2 == ""

replace v2 = v53 if v2 == ""

replace v3 = v6 if v3 == ""

replace v3 = v9 if v3 == ""

replace v3 = v12 if v3 == ""

replace v3 = v15 if v3 == ""

replace v3 = v18 if v3 == ""

replace v3 = v21 if v3 == ""

replace v3 = v24 if v3 == ""

replace v3 = v27 if v3 == ""

replace v3 = v30 if v3 == ""

replace v3 = v33 if v3 == ""

replace v3 = v36 if v3 == ""

replace v3 = v39 if v3 == ""

replace v3 = v42 if v3 == ""

replace v3 = v45 if v3 == ""

replace v3 = v48 if v3 == ""

replace v3 = v51 if v3 == ""

replace v3 = v54 if v3 == ""

replace v4 = v7 if v4 == ""

replace v4 = v10 if v4 == ""

replace v4 = v13 if v4 == ""

replace v4 = v16 if v4 == ""

replace v4 = v19 if v4 == ""

replace v4 = v22 if v4 == ""

replace v4 = v25 if v4 == ""

replace v4 = v28 if v4 == ""

replace v4 = v31 if v4 == ""

replace v4 = v34 if v4 == ""

replace v4 = v37 if v4 == ""

replace v4 = v40 if v4 == ""

replace v4 = v43 if v4 == ""

replace v4 = v46 if v4 == ""

replace v4 = v49 if v4 == ""

replace v4 = v52 if v4 == ""

replace v4 = v55 if v4 == ""

keep v1 v2 v3 v4

drop if v1 == ""

drop if v1 == "main"

drop if v1 == "_cons"

destring v1, replace ignore(file)

rename v2 Diff_IVR_Age_Sex

rename v3 Diff_IVR_Age_Sex_CI

rename v4 Diff_IVR_Age_Sex_P

gen pop_group = 1

gen number = v1

tostring v1, replace

replace v1 = "Any current tobacco user" if v1 == "1"

replace v1 = "Current smoker" if v1 == "2"

replace v1 = "Current smokeless tobacco user" if v1 == "3"

replace v1 = "Any daily tobacco user" if v1 == "4"

replace v1 = "Daily smoker" if v1 == "5"

replace v1 = "Daily smokeless tobacco user" if v1 == "6"

replace v1 = "Only smoker" if v1 == "7"

replace v1 = "Only smokeless tobacco user" if v1 == "8"

replace v1 = "Both smoker and smokeless user" if v1 == "9"

replace v1 = "Alcohol past month" if v1 == "10"

replace v1 = "No fruit or vegetables" if v1 == "11"

replace v1 = "No fruit" if v1 == "12"

replace v1 = "No veg" if v1 == "13"

replace v1 = "<5 servings of fruits-veg in a day " if v1 == "14"

replace v1 = "Add salt to food while eating" if v1 == "15"

replace v1 = "Processed food high in salt" if v1 == "16"

replace v1 = "Known raised BP/HTN" if v1 == "17"

replace v1 = "Take medication for BP/HTN" if v1 == "18"

save File_RD_IVR_Age_Sex, replace

restore

*********

preserve

use Table3_Unweighted.dta, replace

merge 1:1 v1 using Table3_Weighted_IPW, nogen

merge 1:1 v1 using Table3_Weighted_Age_Sex_W.dta, nogen

merge 1:1 v1 using Table3_n.dta, nogen

sort id

drop id

label var v1 "Indicator"

drop STEPS_IPW

gen indicator = _n

gen pop_group = 2

save Table3_02_Prevalence_GMAK_20Jul2022.dta, replace

merge 1:1 v1 using File_RD_IVR_IPW.dta, nogen

merge 1:1 v1 using File_RD_IVR_Age_Sex.dta, nogen

sort number

order v1 IVR_n IVR_IPW IVR_IPW_CI STEPS_n STEPS_Age_Sex STEPS_Age_Sex_CI Diff_IVR_IPW

export excel v1 IVR_n IVR_IPW IVR_IPW_CI STEPS_n STEPS_Age_Sex STEPS_Age_Sex_CI ///

Diff_IVR_IPW using SumTab_IVR_IPWvsSTEPS.xls, replace

order v1 IVR_n IVR_Age_Sex IVR_Age_Sex_CI STEPS_n STEPS_Age_Sex STEPS_Age_Sex_CI ///

Diff_IVR_Age_Sex

export excel v1 IVR_n IVR_Age_Sex IVR_Age_Sex_CI STEPS_n STEPS_Age_Sex STEPS_Age_Sex_CI ///

Diff_IVR_Age_Sex using SumTab_IVR_AgeSexvsSTEPS.xls, replace

restore

********************************************************************************

preserve

keep if age_cat == 2

tabout current_tobacco_any current_smoke_n current_smokeless_n ///

daily_tobacco_any daily_smoke_n daily_smokeless_n ///

smoke_only smokeless_only both_smoke_smokeless ///

alcohol_current no_fruit_veg no_fruit no_veg ///

atelessthan5 salt_eat_n salt_pack_n ///

htn_known htn_med file1 ///

using Table3_n.xls, ///

c(freq col) f(1c 1p) replace

import delimited using Table3_n.xls, clear

drop if v1 == "No"

drop if v1 == "Yes"

replace v1 = v1[_n - 1] if v1 == "Total"

drop v3 v5 v6 v7

drop if v1 == ""

drop if v2 == ""

destring v2 v4, ignore(",") replace

rename v2 IVR_n

rename v4 STEPS_n

gen id = _n

save Table3_n.dta, replace

restore

preserve

keep if age_cat == 2

gen wt = 1

svyset [pweight= wt]

tabout current_tobacco_any current_smoke_n current_smokeless_n ///

daily_tobacco_any daily_smoke_n daily_smokeless_n ///

smoke_only smokeless_only both_smoke_smokeless ///

alcohol_current no_fruit_veg no_fruit no_veg ///

atelessthan5 salt_eat_n salt_pack_n ///

htn_known htn_med file1 ///

using Table3_Unweight.xls, ///

c(col ci) f(1 1) percent svy replace

import delimited using Table3_Unweight.xls, clear

drop v6 v7

drop if v1 == "No" | v1 == "Total"

replace v1 = v1[_n - 1] if v1 == "Yes"

drop if v2 == ""

drop if v1 == ""

rename v2 IVR_Unweight

rename v4 STEPS_Unweight

rename v3 IVR_Unweight_CI

rename v5 STEPS_Unweight_CI

gen id = _n

save Table3_Unweighted.dta, replace

restore

********

preserve

keep if age_cat == 2

svyset [pweight= weight_ipw]

tabout current_tobacco_any current_smoke_n current_smokeless_n ///

daily_tobacco_any daily_smoke_n daily_smokeless_n ///

smoke_only smokeless_only both_smoke_smokeless ///

alcohol_current no_fruit_veg no_fruit no_veg ///

atelessthan5 salt_eat_n salt_pack_n ///

htn_known htn_med file1 ///

using Table3_Weight_IPW.xls, ///

c(col ci) f(1 1) percent svy replace

import delimited using Table3_Weight_IPW.xls, clear

drop v6 v7

drop if v1 == "No" | v1 == "Total"

replace v1 = v1[_n - 1] if v1 == "Yes"

drop if v2 == ""

drop if v1 == ""

rename v2 IVR_IPW

rename v4 STEPS_IPW

rename v3 IVR_IPW_CI

rename v5 STEPS_IPW_CI

gen id = _n

save Table3_Weighted_IPW.dta, replace

restore

********

preserve

keep if age_cat == 2

svyset [pweight= age_sex_w]

tabout current_tobacco_any current_smoke_n current_smokeless_n ///

daily_tobacco_any daily_smoke_n daily_smokeless_n ///

smoke_only smokeless_only both_smoke_smokeless ///

alcohol_current no_fruit_veg no_fruit no_veg ///

atelessthan5 salt_eat_n salt_pack_n ///

htn_known htn_med file1 ///

using Table3_Weight_Age_Sex_W.xls, ///

c(col ci) f(1 1) percent svy replace

import delimited using Table3_Weight_Age_Sex_W.xls, clear

drop v6 v7

drop if v1 == "No" | v1 == "Total"

replace v1 = v1[_n - 1] if v1 == "Yes"

drop if v2 == ""

drop if v1 == ""

rename v2 IVR_Age_Sex

rename v4 STEPS_Age_Sex

rename v3 IVR_Age_Sex_CI

rename v5 STEPS_Age_Sex_CI

gen id = _n

save Table3_Weighted_Age_Sex_W.dta, replace

restore

********

******

*

preserve

recode file1 (1 = 0) (0 = 1)

keep if age_cat == 2

svyset [pweight= weight_ipw]

gen file2 = file1

gen file3 = file1

gen file4 = file1

gen file5 = file1

gen file6 = file1

gen file7 = file1

gen file8 = file1

gen file9 = file1

gen file10 = file1

gen file11 = file1

gen file12 = file1

gen file13 = file1

gen file14 = file1

gen file15 = file1

gen file16 = file1

gen file17 = file1

gen file18 = file1

svy: glm current_tobacco_any file1, fam(bin) link(identity)

estimates store I1

svy: glm current_smoke_n file2, fam(bin) link(identity)

estimates store I2

svy: glm current_smokeless_n file3, fam(bin) link(identity)

estimates store I3

svy: glm daily_tobacco_any file4, fam(bin) link(identity)

estimates store I4

svy: glm daily_smoke_n file5, fam(bin) link(identity)

estimates store I5

svy: glm daily_smokeless_n file6, fam(bin) link(identity)

estimates store I6

svy: glm smoke_only file7, fam(bin) link(identity)

estimates store I7

svy: glm smokeless_only file8, fam(bin) link(identity)

estimates store I8

svy: glm both_smoke_smokeless file9, fam(bin) link(identity)

estimates store I9

svy: glm alcohol_current file10, fam(bin) link(identity)

estimates store I10

svy: glm no_fruit_veg file11, fam(bin) link(identity)

estimates store I11

svy: glm no_fruit file12, fam(bin) link(identity)

estimates store I12

svy: glm no_veg file13, fam(bin) link(identity)

estimates store I13

svy: glm atelessthan5 file14, fam(bin) link(identity)

estimates store I14

svy: glm salt_eat_n file15, fam(bin) link(identity)

estimates store I15

svy: glm salt_pack_n file16, fam(bin) link(identity)

estimates store I16

svy: glm htn_known file17, fam(bin) link(identity)

estimates store I17

svy: glm htn_med file18, fam(bin) link(identity)

estimates store I18

estout I1 I2 I3 I4 I5 I6 I7 I8 I9 I10 I11 I12 I13 I14 I15 I16 I17 I18 using Table_RD.xls, ///

cells ("b(star fmt(1)) ci(par fmt(1) label(95% CI)) p (fmt(4)) ") replace transform(@*100)

import delimited "Table_RD.xls", clear

replace v2 = v5 if v2 == ""

replace v2 = v8 if v2 == ""

replace v2 = v11 if v2 == ""

replace v2 = v14 if v2 == ""

replace v2 = v17 if v2 == ""

replace v2 = v20 if v2 == ""

replace v2 = v23 if v2 == ""

replace v2 = v26 if v2 == ""

replace v2 = v29 if v2 == ""

replace v2 = v32 if v2 == ""

replace v2 = v35 if v2 == ""

replace v2 = v38 if v2 == ""

replace v2 = v41 if v2 == ""

replace v2 = v44 if v2 == ""

replace v2 = v47 if v2 == ""

replace v2 = v50 if v2 == ""

replace v2 = v53 if v2 == ""

replace v3 = v6 if v3 == ""

replace v3 = v9 if v3 == ""

replace v3 = v12 if v3 == ""

replace v3 = v15 if v3 == ""

replace v3 = v18 if v3 == ""

replace v3 = v21 if v3 == ""

replace v3 = v24 if v3 == ""

replace v3 = v27 if v3 == ""

replace v3 = v30 if v3 == ""

replace v3 = v33 if v3 == ""

replace v3 = v36 if v3 == ""

replace v3 = v39 if v3 == ""

replace v3 = v42 if v3 == ""

replace v3 = v45 if v3 == ""

replace v3 = v48 if v3 == ""

replace v3 = v51 if v3 == ""

replace v3 = v54 if v3 == ""

replace v4 = v7 if v4 == ""

replace v4 = v10 if v4 == ""

replace v4 = v13 if v4 == ""

replace v4 = v16 if v4 == ""

replace v4 = v19 if v4 == ""

replace v4 = v22 if v4 == ""

replace v4 = v25 if v4 == ""

replace v4 = v28 if v4 == ""

replace v4 = v31 if v4 == ""

replace v4 = v34 if v4 == ""

replace v4 = v37 if v4 == ""

replace v4 = v40 if v4 == ""

replace v4 = v43 if v4 == ""

replace v4 = v46 if v4 == ""

replace v4 = v49 if v4 == ""

replace v4 = v52 if v4 == ""

replace v4 = v55 if v4 == ""

keep v1 v2 v3 v4

drop if v1 == ""

drop if v1 == "main"

drop if v1 == "_cons"

destring v1, replace ignore(file)

rename v2 Diff_IVR_IPW

rename v3 Diff_IVR_IPW_CI

rename v4 Diff_IVR_IPW_P

gen pop_group = 1

tostring v1, replace

replace v1 = "Any current tobacco user" if v1 == "1"

replace v1 = "Current smoker" if v1 == "2"

replace v1 = "Current smokeless tobacco user" if v1 == "3"

replace v1 = "Any daily tobacco user" if v1 == "4"

replace v1 = "Daily smoker" if v1 == "5"

replace v1 = "Daily smokeless tobacco user" if v1 == "6"

replace v1 = "Only smoker" if v1 == "7"

replace v1 = "Only smokeless tobacco user" if v1 == "8"

replace v1 = "Both smoker and smokeless user" if v1 == "9"

replace v1 = "Alcohol past month" if v1 == "10"

replace v1 = "No fruit or vegetables" if v1 == "11"

replace v1 = "No fruit" if v1 == "12"

replace v1 = "No veg" if v1 == "13"

replace v1 = "<5 servings of fruits-veg in a day " if v1 == "14"

replace v1 = "Add salt to food while eating" if v1 == "15"

replace v1 = "Processed food high in salt" if v1 == "16"

replace v1 = "Known raised BP/HTN" if v1 == "17"

replace v1 = "Take medication for BP/HTN" if v1 == "18"

save File_RD_IVR_IPW, replace

restore

********

preserve

recode file1 (1 = 0) (0 = 1)

keep if age_cat == 2

svyset [pweight= age_sex_w]

gen file2 = file1

gen file3 = file1

gen file4 = file1

gen file5 = file1

gen file6 = file1

gen file7 = file1

gen file8 = file1

gen file9 = file1

gen file10 = file1

gen file11 = file1

gen file12 = file1

gen file13 = file1

gen file14 = file1

gen file15 = file1

gen file16 = file1

gen file17 = file1

gen file18 = file1

svy: glm current_tobacco_any file1, fam(bin) link(identity)

estimates store I1

svy: glm current_smoke_n file2, fam(bin) link(identity)

estimates store I2

svy: glm current_smokeless_n file3, fam(bin) link(identity)

estimates store I3

svy: glm daily_tobacco_any file4, fam(bin) link(identity)

estimates store I4

svy: glm daily_smoke_n file5, fam(bin) link(identity)

estimates store I5

svy: glm daily_smokeless_n file6, fam(bin) link(identity)

estimates store I6

svy: glm smoke_only file7, fam(bin) link(identity)

estimates store I7

svy: glm smokeless_only file8, fam(bin) link(identity)

estimates store I8

svy: glm both_smoke_smokeless file9, fam(bin) link(identity)

estimates store I9

svy: glm alcohol_current file10, fam(bin) link(identity)

estimates store I10

svy: glm no_fruit_veg file11, fam(bin) link(identity)

estimates store I11

svy: glm no_fruit file12, fam(bin) link(identity)

estimates store I12

svy: glm no_veg file13, fam(bin) link(identity)

estimates store I13

svy: glm atelessthan5 file14, fam(bin) link(identity)

estimates store I14

svy: glm salt_eat_n file15, fam(bin) link(identity)

estimates store I15

svy: glm salt_pack_n file16, fam(bin) link(identity)

estimates store I16

svy: glm htn_known file17, fam(bin) link(identity)

estimates store I17

svy: glm htn_med file18, fam(bin) link(identity)

estimates store I18

estout I1 I2 I3 I4 I5 I6 I7 I8 I9 I10 I11 I12 I13 I14 I15 I16 I17 I18 using Table_RD.xls, ///

cells ("b(star fmt(1)) ci(par fmt(1) label(95% CI)) p (fmt(4)) ") replace transform(@*100)

import delimited "Table_RD.xls", clear

replace v2 = v5 if v2 == ""

replace v2 = v8 if v2 == ""

replace v2 = v11 if v2 == ""

replace v2 = v14 if v2 == ""

replace v2 = v17 if v2 == ""

replace v2 = v20 if v2 == ""

replace v2 = v23 if v2 == ""

replace v2 = v26 if v2 == ""

replace v2 = v29 if v2 == ""

replace v2 = v32 if v2 == ""

replace v2 = v35 if v2 == ""

replace v2 = v38 if v2 == ""

replace v2 = v41 if v2 == ""

replace v2 = v44 if v2 == ""

replace v2 = v47 if v2 == ""

replace v2 = v50 if v2 == ""

replace v2 = v53 if v2 == ""

replace v3 = v6 if v3 == ""

replace v3 = v9 if v3 == ""

replace v3 = v12 if v3 == ""

replace v3 = v15 if v3 == ""

replace v3 = v18 if v3 == ""

replace v3 = v21 if v3 == ""

replace v3 = v24 if v3 == ""

replace v3 = v27 if v3 == ""

replace v3 = v30 if v3 == ""

replace v3 = v33 if v3 == ""

replace v3 = v36 if v3 == ""

replace v3 = v39 if v3 == ""

replace v3 = v42 if v3 == ""

replace v3 = v45 if v3 == ""

replace v3 = v48 if v3 == ""

replace v3 = v51 if v3 == ""

replace v3 = v54 if v3 == ""

replace v4 = v7 if v4 == ""

replace v4 = v10 if v4 == ""

replace v4 = v13 if v4 == ""

replace v4 = v16 if v4 == ""

replace v4 = v19 if v4 == ""

replace v4 = v22 if v4 == ""

replace v4 = v25 if v4 == ""

replace v4 = v28 if v4 == ""

replace v4 = v31 if v4 == ""

replace v4 = v34 if v4 == ""

replace v4 = v37 if v4 == ""

replace v4 = v40 if v4 == ""

replace v4 = v43 if v4 == ""

replace v4 = v46 if v4 == ""

replace v4 = v49 if v4 == ""

replace v4 = v52 if v4 == ""

replace v4 = v55 if v4 == ""

keep v1 v2 v3 v4

drop if v1 == ""

drop if v1 == "main"

drop if v1 == "_cons"

destring v1, replace ignore(file)

rename v2 Diff_IVR_Age_Sex

rename v3 Diff_IVR_Age_Sex_CI

rename v4 Diff_IVR_Age_Sex_P

gen pop_group = 1

gen number = v1

tostring v1, replace

replace v1 = "Any current tobacco user" if v1 == "1"

replace v1 = "Current smoker" if v1 == "2"

replace v1 = "Current smokeless tobacco user" if v1 == "3"

replace v1 = "Any daily tobacco user" if v1 == "4"

replace v1 = "Daily smoker" if v1 == "5"

replace v1 = "Daily smokeless tobacco user" if v1 == "6"

replace v1 = "Only smoker" if v1 == "7"

replace v1 = "Only smokeless tobacco user" if v1 == "8"

replace v1 = "Both smoker and smokeless user" if v1 == "9"

replace v1 = "Alcohol past month" if v1 == "10"

replace v1 = "No fruit or vegetables" if v1 == "11"

replace v1 = "No fruit" if v1 == "12"

replace v1 = "No veg" if v1 == "13"

replace v1 = "<5 servings of fruits-veg in a day " if v1 == "14"

replace v1 = "Add salt to food while eating" if v1 == "15"

replace v1 = "Processed food high in salt" if v1 == "16"

replace v1 = "Known raised BP/HTN" if v1 == "17"

replace v1 = "Take medication for BP/HTN" if v1 == "18"

save File_RD_IVR_Age_Sex, replace

restore

*******

preserve

use Table3_Unweighted.dta, replace

merge 1:1 v1 using Table3_Weighted_IPW, nogen

merge 1:1 v1 using Table3_Weighted_Age_Sex_W.dta, nogen

merge 1:1 v1 using Table3_n.dta, nogen

sort id

drop id

label var v1 "Indicator"

drop STEPS_IPW

gen indicator = _n

gen pop_group = 3

save Table3_03_Prevalence_GMAK_20Jul2022.dta, replace

merge 1:1 v1 using File_RD_IVR_IPW.dta, nogen

merge 1:1 v1 using File_RD_IVR_Age_Sex.dta, nogen

sort number

order v1 IVR_n IVR_IPW IVR_IPW_CI STEPS_n STEPS_Age_Sex STEPS_Age_Sex_CI Diff_IVR_IPW

export excel v1 IVR_n IVR_IPW IVR_IPW_CI STEPS_n STEPS_Age_Sex STEPS_Age_Sex_CI ///

Diff_IVR_IPW using SumTab_IVR_IPWvsSTEPS.xls, replace

order v1 IVR_n IVR_Age_Sex IVR_Age_Sex_CI STEPS_n STEPS_Age_Sex STEPS_Age_Sex_CI ///

Diff_IVR_Age_Sex

export excel v1 IVR_n IVR_Age_Sex IVR_Age_Sex_CI STEPS_n STEPS_Age_Sex STEPS_Age_Sex_CI ///

Diff_IVR_Age_Sex using SumTab_IVR_AgeSexvsSTEPS.xls, replace

restore

********************************************************************************

preserve

tabout current_tobacco_any current_smoke_n current_smokeless_n ///

daily_tobacco_any daily_smoke_n daily_smokeless_n ///

smoke_only smokeless_only both_smoke_smokeless ///

alcohol_current no_fruit_veg no_fruit no_veg ///

atelessthan5 salt_eat_n salt_pack_n ///

htn_known htn_med file1 ///

using Table3_n.xls, ///

c(freq col) f(1c 1p) replace

import delimited using Table3_n.xls, clear

drop if v1 == "No"

drop if v1 == "Yes"

replace v1 = v1[_n - 1] if v1 == "Total"

drop v3 v5 v6 v7

drop if v1 == ""

drop if v2 == ""

destring v2 v4, ignore(",") replace

rename v2 IVR_n

rename v4 STEPS_n

gen id = _n

save Table3_n.dta, replace

restore

preserve

gen wt = 1

svyset [pweight= wt]

tabout current_tobacco_any current_smoke_n current_smokeless_n ///

daily_tobacco_any daily_smoke_n daily_smokeless_n ///

smoke_only smokeless_only both_smoke_smokeless ///

alcohol_current no_fruit_veg no_fruit no_veg ///

atelessthan5 salt_eat_n salt_pack_n ///

htn_known htn_med file1 ///

using Table3_Unweight.xls, ///

c(col ci) f(1 1) percent svy replace

import delimited using Table3_Unweight.xls, clear

drop v6 v7

drop if v1 == "No" | v1 == "Total"

replace v1 = v1[_n - 1] if v1 == "Yes"

drop if v2 == ""

drop if v1 == ""

rename v2 IVR_Unweight

rename v4 STEPS_Unweight

rename v3 IVR_Unweight_CI

rename v5 STEPS_Unweight_CI

gen id = _n

save Table3_Unweighted.dta, replace

restore

********

preserve

svyset [pweight= weight_ipw]

tabout current_tobacco_any current_smoke_n current_smokeless_n ///

daily_tobacco_any daily_smoke_n daily_smokeless_n ///

smoke_only smokeless_only both_smoke_smokeless ///

alcohol_current no_fruit_veg no_fruit no_veg ///

atelessthan5 salt_eat_n salt_pack_n ///

htn_known htn_med file1 ///

using Table3_Weight_IPW.xls, ///

c(col ci) f(1 1) percent svy replace

import delimited using Table3_Weight_IPW.xls, clear

drop v6 v7

drop if v1 == "No" | v1 == "Total"

replace v1 = v1[_n - 1] if v1 == "Yes"

drop if v2 == ""

drop if v1 == ""

rename v2 IVR_IPW

rename v4 STEPS_IPW

rename v3 IVR_IPW_CI

rename v5 STEPS_IPW_CI

gen id = _n

save Table3_Weighted_IPW.dta, replace

restore

********

preserve

svyset [pweight= age_sex_w]

tabout current_tobacco_any current_smoke_n current_smokeless_n ///

daily_tobacco_any daily_smoke_n daily_smokeless_n ///

smoke_only smokeless_only both_smoke_smokeless ///

alcohol_current no_fruit_veg no_fruit no_veg ///

atelessthan5 salt_eat_n salt_pack_n ///

htn_known htn_med file1 ///

using Table3_Weight_Age_Sex_W.xls, ///

c(col ci) f(1 1) percent svy replace

import delimited using Table3_Weight_Age_Sex_W.xls, clear

drop v6 v7

drop if v1 == "No" | v1 == "Total"

replace v1 = v1[_n - 1] if v1 == "Yes"

drop if v2 == ""

drop if v1 == ""

rename v2 IVR_Age_Sex

rename v4 STEPS_Age_Sex

rename v3 IVR_Age_Sex_CI

rename v5 STEPS_Age_Sex_CI

gen id = _n

save Table3_Weighted_Age_Sex_W.dta, replace

restore

*****

preserve

recode file1 (1 = 0) (0 = 1)

svyset [pweight= weight_ipw]

gen file2 = file1

gen file3 = file1

gen file4 = file1

gen file5 = file1

gen file6 = file1

gen file7 = file1

gen file8 = file1

gen file9 = file1

gen file10 = file1

gen file11 = file1

gen file12 = file1

gen file13 = file1

gen file14 = file1

gen file15 = file1

gen file16 = file1

gen file17 = file1

gen file18 = file1

svy: glm current_tobacco_any file1, fam(bin) link(identity)

estimates store I1

svy: glm current_smoke_n file2, fam(bin) link(identity)

estimates store I2

svy: glm current_smokeless_n file3, fam(bin) link(identity)

estimates store I3

svy: glm daily_tobacco_any file4, fam(bin) link(identity)

estimates store I4

svy: glm daily_smoke_n file5, fam(bin) link(identity)

estimates store I5

svy: glm daily_smokeless_n file6, fam(bin) link(identity)

estimates store I6

svy: glm smoke_only file7, fam(bin) link(identity)

estimates store I7

svy: glm smokeless_only file8, fam(bin) link(identity)

estimates store I8

svy: glm both_smoke_smokeless file9, fam(bin) link(identity)

estimates store I9

svy: glm alcohol_current file10, fam(bin) link(identity)

estimates store I10

svy: glm no_fruit_veg file11, fam(bin) link(identity)

estimates store I11

svy: glm no_fruit file12, fam(bin) link(identity)

estimates store I12

svy: glm no_veg file13, fam(bin) link(identity)

estimates store I13

svy: glm atelessthan5 file14, fam(bin) link(identity)

estimates store I14

svy: glm salt_eat_n file15, fam(bin) link(identity)

estimates store I15

svy: glm salt_pack_n file16, fam(bin) link(identity)

estimates store I16

svy: glm htn_known file17, fam(bin) link(identity)

estimates store I17

svy: glm htn_med file18, fam(bin) link(identity)

estimates store I18

estout I1 I2 I3 I4 I5 I6 I7 I8 I9 I10 I11 I12 I13 I14 I15 I16 I17 I18 using Table_RD.xls, ///

cells ("b(star fmt(1)) ci(par fmt(1) label(95% CI)) p (fmt(4)) ") replace transform(@*100)

import delimited "Table_RD.xls", clear

replace v2 = v5 if v2 == ""

replace v2 = v8 if v2 == ""

replace v2 = v11 if v2 == ""

replace v2 = v14 if v2 == ""

replace v2 = v17 if v2 == ""

replace v2 = v20 if v2 == ""

replace v2 = v23 if v2 == ""

replace v2 = v26 if v2 == ""

replace v2 = v29 if v2 == ""

replace v2 = v32 if v2 == ""

replace v2 = v35 if v2 == ""

replace v2 = v38 if v2 == ""

replace v2 = v41 if v2 == ""

replace v2 = v44 if v2 == ""

replace v2 = v47 if v2 == ""

replace v2 = v50 if v2 == ""

replace v2 = v53 if v2 == ""

replace v3 = v6 if v3 == ""

replace v3 = v9 if v3 == ""

replace v3 = v12 if v3 == ""

replace v3 = v15 if v3 == ""

replace v3 = v18 if v3 == ""

replace v3 = v21 if v3 == ""

replace v3 = v24 if v3 == ""

replace v3 = v27 if v3 == ""

replace v3 = v30 if v3 == ""

replace v3 = v33 if v3 == ""

replace v3 = v36 if v3 == ""

replace v3 = v39 if v3 == ""

replace v3 = v42 if v3 == ""

replace v3 = v45 if v3 == ""

replace v3 = v48 if v3 == ""

replace v3 = v51 if v3 == ""

replace v3 = v54 if v3 == ""

replace v4 = v7 if v4 == ""

replace v4 = v10 if v4 == ""

replace v4 = v13 if v4 == ""

replace v4 = v16 if v4 == ""

replace v4 = v19 if v4 == ""

replace v4 = v22 if v4 == ""

replace v4 = v25 if v4 == ""

replace v4 = v28 if v4 == ""

replace v4 = v31 if v4 == ""

replace v4 = v34 if v4 == ""

replace v4 = v37 if v4 == ""

replace v4 = v40 if v4 == ""

replace v4 = v43 if v4 == ""

replace v4 = v46 if v4 == ""

replace v4 = v49 if v4 == ""

replace v4 = v52 if v4 == ""

replace v4 = v55 if v4 == ""

keep v1 v2 v3 v4

drop if v1 == ""

drop if v1 == "main"

drop if v1 == "_cons"

destring v1, replace ignore(file)

rename v2 Diff_IVR_IPW

rename v3 Diff_IVR_IPW_CI

rename v4 Diff_IVR_IPW_P

gen pop_group = 1

tostring v1, replace

replace v1 = "Any current tobacco user" if v1 == "1"

replace v1 = "Current smoker" if v1 == "2"

replace v1 = "Current smokeless tobacco user" if v1 == "3"

replace v1 = "Any daily tobacco user" if v1 == "4"

replace v1 = "Daily smoker" if v1 == "5"

replace v1 = "Daily smokeless tobacco user" if v1 == "6"

replace v1 = "Only smoker" if v1 == "7"

replace v1 = "Only smokeless tobacco user" if v1 == "8"

replace v1 = "Both smoker and smokeless user" if v1 == "9"

replace v1 = "Alcohol past month" if v1 == "10"

replace v1 = "No fruit or vegetables" if v1 == "11"

replace v1 = "No fruit" if v1 == "12"

replace v1 = "No veg" if v1 == "13"

replace v1 = "<5 servings of fruits-veg in a day " if v1 == "14"

replace v1 = "Add salt to food while eating" if v1 == "15"

replace v1 = "Processed food high in salt" if v1 == "16"

replace v1 = "Known raised BP/HTN" if v1 == "17"

replace v1 = "Take medication for BP/HTN" if v1 == "18"

save File_RD_IVR_IPW, replace

restore

********

preserve

recode file1 (1 = 0) (0 = 1)

svyset [pweight= age_sex_w]

gen file2 = file1

gen file3 = file1

gen file4 = file1

gen file5 = file1

gen file6 = file1

gen file7 = file1

gen file8 = file1

gen file9 = file1

gen file10 = file1

gen file11 = file1

gen file12 = file1

gen file13 = file1

gen file14 = file1

gen file15 = file1

gen file16 = file1

gen file17 = file1

gen file18 = file1

svy: glm current_tobacco_any file1, fam(bin) link(identity)

estimates store I1

svy: glm current_smoke_n file2, fam(bin) link(identity)

estimates store I2

svy: glm current_smokeless_n file3, fam(bin) link(identity)

estimates store I3

svy: glm daily_tobacco_any file4, fam(bin) link(identity)

estimates store I4

svy: glm daily_smoke_n file5, fam(bin) link(identity)

estimates store I5

svy: glm daily_smokeless_n file6, fam(bin) link(identity)

estimates store I6

svy: glm smoke_only file7, fam(bin) link(identity)

estimates store I7

svy: glm smokeless_only file8, fam(bin) link(identity)

estimates store I8

svy: glm both_smoke_smokeless file9, fam(bin) link(identity)

estimates store I9

svy: glm alcohol_current file10, fam(bin) link(identity)

estimates store I10

svy: glm no_fruit_veg file11, fam(bin) link(identity)

estimates store I11

svy: glm no_fruit file12, fam(bin) link(identity)

estimates store I12

svy: glm no_veg file13, fam(bin) link(identity)

estimates store I13

svy: glm atelessthan5 file14, fam(bin) link(identity)

estimates store I14

svy: glm salt_eat_n file15, fam(bin) link(identity)

estimates store I15

svy: glm salt_pack_n file16, fam(bin) link(identity)

estimates store I16

svy: glm htn_known file17, fam(bin) link(identity)

estimates store I17

svy: glm htn_med file18, fam(bin) link(identity)

estimates store I18

estout I1 I2 I3 I4 I5 I6 I7 I8 I9 I10 I11 I12 I13 I14 I15 I16 I17 I18 using Table_RD.xls, ///

cells ("b(star fmt(1)) ci(par fmt(1) label(95% CI)) p (fmt(4)) ") replace transform(@*100)

import delimited "Table_RD.xls", clear

replace v2 = v5 if v2 == ""

replace v2 = v8 if v2 == ""

replace v2 = v11 if v2 == ""

replace v2 = v14 if v2 == ""

replace v2 = v17 if v2 == ""

replace v2 = v20 if v2 == ""

replace v2 = v23 if v2 == ""

replace v2 = v26 if v2 == ""

replace v2 = v29 if v2 == ""

replace v2 = v32 if v2 == ""

replace v2 = v35 if v2 == ""

replace v2 = v38 if v2 == ""

replace v2 = v41 if v2 == ""

replace v2 = v44 if v2 == ""

replace v2 = v47 if v2 == ""

replace v2 = v50 if v2 == ""

replace v2 = v53 if v2 == ""

replace v3 = v6 if v3 == ""

replace v3 = v9 if v3 == ""

replace v3 = v12 if v3 == ""

replace v3 = v15 if v3 == ""

replace v3 = v18 if v3 == ""

replace v3 = v21 if v3 == ""

replace v3 = v24 if v3 == ""

replace v3 = v27 if v3 == ""

replace v3 = v30 if v3 == ""

replace v3 = v33 if v3 == ""

replace v3 = v36 if v3 == ""

replace v3 = v39 if v3 == ""

replace v3 = v42 if v3 == ""

replace v3 = v45 if v3 == ""

replace v3 = v48 if v3 == ""

replace v3 = v51 if v3 == ""

replace v3 = v54 if v3 == ""

replace v4 = v7 if v4 == ""

replace v4 = v10 if v4 == ""

replace v4 = v13 if v4 == ""

replace v4 = v16 if v4 == ""

replace v4 = v19 if v4 == ""

replace v4 = v22 if v4 == ""

replace v4 = v25 if v4 == ""

replace v4 = v28 if v4 == ""

replace v4 = v31 if v4 == ""

replace v4 = v34 if v4 == ""

replace v4 = v37 if v4 == ""

replace v4 = v40 if v4 == ""

replace v4 = v43 if v4 == ""

replace v4 = v46 if v4 == ""

replace v4 = v49 if v4 == ""

replace v4 = v52 if v4 == ""

replace v4 = v55 if v4 == ""

keep v1 v2 v3 v4

drop if v1 == ""

drop if v1 == "main"

drop if v1 == "_cons"

destring v1, replace ignore(file)

rename v2 Diff_IVR_Age_Sex

rename v3 Diff_IVR_Age_Sex_CI

rename v4 Diff_IVR_Age_Sex_P

gen pop_group = 1

gen number = v1

tostring v1, replace

replace v1 = "Any current tobacco user" if v1 == "1"

replace v1 = "Current smoker" if v1 == "2"

replace v1 = "Current smokeless tobacco user" if v1 == "3"

replace v1 = "Any daily tobacco user" if v1 == "4"

replace v1 = "Daily smoker" if v1 == "5"

replace v1 = "Daily smokeless tobacco user" if v1 == "6"

replace v1 = "Only smoker" if v1 == "7"

replace v1 = "Only smokeless tobacco user" if v1 == "8"

replace v1 = "Both smoker and smokeless user" if v1 == "9"

replace v1 = "Alcohol past month" if v1 == "10"

replace v1 = "No fruit or vegetables" if v1 == "11"

replace v1 = "No fruit" if v1 == "12"

replace v1 = "No veg" if v1 == "13"

replace v1 = "<5 servings of fruits-veg in a day " if v1 == "14"

replace v1 = "Add salt to food while eating" if v1 == "15"

replace v1 = "Processed food high in salt" if v1 == "16"

replace v1 = "Known raised BP/HTN" if v1 == "17"

replace v1 = "Take medication for BP/HTN" if v1 == "18"

save File_RD_IVR_Age_Sex, replace

restore

preserve

use Table3_Unweighted.dta, replace

merge 1:1 v1 using Table3_Weighted_IPW, nogen

merge 1:1 v1 using Table3_Weighted_Age_Sex_W.dta, nogen

merge 1:1 v1 using Table3_n.dta, nogen

sort id

drop id

label var v1 "Indicator"

drop STEPS_IPW

gen indicator = _n

gen pop_group = 4

save Table3_04_Prevalence_GMAK_20Jul2022.dta, replace

merge 1:1 v1 using File_RD_IVR_IPW.dta, nogen

merge 1:1 v1 using File_RD_IVR_Age_Sex.dta, nogen

sort number

order v1 IVR_n IVR_IPW IVR_IPW_CI STEPS_n STEPS_Age_Sex STEPS_Age_Sex_CI Diff_IVR_IPW

export excel v1 IVR_n IVR_IPW IVR_IPW_CI STEPS_n STEPS_Age_Sex STEPS_Age_Sex_CI ///

Diff_IVR_IPW using SumTab_IVR_IPWvsSTEPS.xls, replace

order v1 IVR_n IVR_Age_Sex IVR_Age_Sex_CI STEPS_n STEPS_Age_Sex STEPS_Age_Sex_CI ///

Diff_IVR_Age_Sex

export excel v1 IVR_n IVR_Age_Sex IVR_Age_Sex_CI STEPS_n STEPS_Age_Sex STEPS_Age_Sex_CI ///

Diff_IVR_Age_Sex using SumTab_IVR_AgeSexvsSTEPS.xls, replace

restore

********************************************************************************

preserve

keep if sex_n == 1

keep if age_cat == 0

tabout current_tobacco_any current_smoke_n current_smokeless_n ///

daily_tobacco_any daily_smoke_n daily_smokeless_n ///

smoke_only smokeless_only both_smoke_smokeless ///

alcohol_current no_fruit_veg no_fruit no_veg ///

atelessthan5 salt_eat_n salt_pack_n ///

htn_known htn_med file1 ///

using Table3_n.xls, ///

c(freq col) f(1c 1p) replace

import delimited using Table3_n.xls, clear

drop if v1 == "No"

drop if v1 == "Yes"

replace v1 = v1[_n - 1] if v1 == "Total"

drop v3 v5 v6 v7

drop if v1 == ""

drop if v2 == ""

destring v2 v4, ignore(",") replace

rename v2 IVR_n

rename v4 STEPS_n

gen id = _n

save Table3_n.dta, replace

restore

preserve

keep if sex_n == 1

keep if age_cat == 0

gen wt = 1

svyset [pweight= wt]

tabout current_tobacco_any current_smoke_n current_smokeless_n ///

daily_tobacco_any daily_smoke_n daily_smokeless_n ///

smoke_only smokeless_only both_smoke_smokeless ///

alcohol_current no_fruit_veg no_fruit no_veg ///

atelessthan5 salt_eat_n salt_pack_n ///

htn_known htn_med file1 ///

using Table3_Unweight.xls, ///

c(col ci) f(1 1) percent svy replace

import delimited using Table3_Unweight.xls, clear

drop v6 v7

drop if v1 == "No" | v1 == "Total"

replace v1 = v1[_n - 1] if v1 == "Yes"

drop if v2 == ""

drop if v1 == ""

rename v2 IVR_Unweight

rename v4 STEPS_Unweight

rename v3 IVR_Unweight_CI

rename v5 STEPS_Unweight_CI

gen id = _n

save Table3_Unweighted.dta, replace

restore

********

preserve

keep if sex_n == 1

keep if age_cat == 0

svyset [pweight= weight_ipw]

tabout current_tobacco_any current_smoke_n current_smokeless_n ///

daily_tobacco_any daily_smoke_n daily_smokeless_n ///

smoke_only smokeless_only both_smoke_smokeless ///

alcohol_current no_fruit_veg no_fruit no_veg ///

atelessthan5 salt_eat_n salt_pack_n ///

htn_known htn_med file1 ///

using Table3_Weight_IPW.xls, ///

c(col ci) f(1 1) percent svy replace

import delimited using Table3_Weight_IPW.xls, clear

drop v6 v7

drop if v1 == "No" | v1 == "Total"

replace v1 = v1[_n - 1] if v1 == "Yes"

drop if v2 == ""

drop if v1 == ""

rename v2 IVR_IPW

rename v4 STEPS_IPW

rename v3 IVR_IPW_CI

rename v5 STEPS_IPW_CI

gen id = _n

save Table3_Weighted_IPW.dta, replace

restore

********

preserve

keep if sex_n == 1

keep if age_cat == 0

svyset [pweight= age_sex_w]

tabout current_tobacco_any current_smoke_n current_smokeless_n ///

daily_tobacco_any daily_smoke_n daily_smokeless_n ///

smoke_only smokeless_only both_smoke_smokeless ///

alcohol_current no_fruit_veg no_fruit no_veg ///

atelessthan5 salt_eat_n salt_pack_n ///

htn_known htn_med file1 ///

using Table3_Weight_Age_Sex_W.xls, ///

c(col ci) f(1 1) percent svy replace

import delimited using Table3_Weight_Age_Sex_W.xls, clear

drop v6 v7

drop if v1 == "No" | v1 == "Total"

replace v1 = v1[_n - 1] if v1 == "Yes"

drop if v2 == ""

drop if v1 == ""

rename v2 IVR_Age_Sex

rename v4 STEPS_Age_Sex

rename v3 IVR_Age_Sex_CI

rename v5 STEPS_Age_Sex_CI

gen id = _n

save Table3_Weighted_Age_Sex_W.dta, replace

restore

********

*****

preserve

recode file1 (1 = 0) (0 = 1)

keep if sex_n == 1

keep if age_cat == 0

svyset [pweight= weight_ipw]

gen file2 = file1

gen file3 = file1

gen file4 = file1

gen file5 = file1

gen file6 = file1

gen file7 = file1

gen file8 = file1

gen file9 = file1

gen file10 = file1

gen file11 = file1

gen file12 = file1

gen file13 = file1

gen file14 = file1

gen file15 = file1

gen file16 = file1

gen file17 = file1

gen file18 = file1

svy: glm current_tobacco_any file1, fam(bin) link(identity)

estimates store I1

svy: glm current_smoke_n file2, fam(bin) link(identity)

estimates store I2

svy: glm current_smokeless_n file3, fam(bin) link(identity)

estimates store I3

svy: glm daily_tobacco_any file4, fam(bin) link(identity)

estimates store I4

svy: glm daily_smoke_n file5, fam(bin) link(identity)

estimates store I5

svy: glm daily_smokeless_n file6, fam(bin) link(identity)

estimates store I6

svy: glm smoke_only file7, fam(bin) link(identity)

estimates store I7

svy: glm smokeless_only file8, fam(bin) link(identity)

estimates store I8

svy: glm both_smoke_smokeless file9, fam(bin) link(identity)

estimates store I9

svy: glm alcohol_current file10, fam(bin) link(identity)

estimates store I10

svy: glm no_fruit_veg file11, fam(bin) link(identity)

estimates store I11

svy: glm no_fruit file12, fam(bin) link(identity)

estimates store I12

svy: glm no_veg file13, fam(bin) link(identity)

estimates store I13

svy: glm atelessthan5 file14, fam(bin) link(identity)

estimates store I14

svy: glm salt_eat_n file15, fam(bin) link(identity)

estimates store I15

svy: glm salt_pack_n file16, fam(bin) link(identity)

estimates store I16

svy: glm htn_known file17, fam(bin) link(identity)

estimates store I17

svy: glm htn_med file18, fam(bin) link(identity)

estimates store I18

estout I1 I2 I3 I4 I5 I6 I7 I8 I9 I10 I11 I12 I13 I14 I15 I16 I17 I18 using Table_RD.xls, ///

cells ("b(star fmt(1)) ci(par fmt(1) label(95% CI)) p (fmt(4)) ") replace transform(@*100)

import delimited "Table_RD.xls", clear

replace v2 = v5 if v2 == ""

replace v2 = v8 if v2 == ""

replace v2 = v11 if v2 == ""

replace v2 = v14 if v2 == ""

replace v2 = v17 if v2 == ""

replace v2 = v20 if v2 == ""

replace v2 = v23 if v2 == ""

replace v2 = v26 if v2 == ""

replace v2 = v29 if v2 == ""

replace v2 = v32 if v2 == ""

replace v2 = v35 if v2 == ""

replace v2 = v38 if v2 == ""

replace v2 = v41 if v2 == ""

replace v2 = v44 if v2 == ""

replace v2 = v47 if v2 == ""

replace v2 = v50 if v2 == ""

replace v2 = v53 if v2 == ""

replace v3 = v6 if v3 == ""

replace v3 = v9 if v3 == ""

replace v3 = v12 if v3 == ""

replace v3 = v15 if v3 == ""

replace v3 = v18 if v3 == ""

replace v3 = v21 if v3 == ""

replace v3 = v24 if v3 == ""

replace v3 = v27 if v3 == ""

replace v3 = v30 if v3 == ""

replace v3 = v33 if v3 == ""

replace v3 = v36 if v3 == ""

replace v3 = v39 if v3 == ""

replace v3 = v42 if v3 == ""

replace v3 = v45 if v3 == ""

replace v3 = v48 if v3 == ""

replace v3 = v51 if v3 == ""

replace v3 = v54 if v3 == ""

replace v4 = v7 if v4 == ""

replace v4 = v10 if v4 == ""

replace v4 = v13 if v4 == ""

replace v4 = v16 if v4 == ""

replace v4 = v19 if v4 == ""

replace v4 = v22 if v4 == ""

replace v4 = v25 if v4 == ""

replace v4 = v28 if v4 == ""

replace v4 = v31 if v4 == ""

replace v4 = v34 if v4 == ""

replace v4 = v37 if v4 == ""

replace v4 = v40 if v4 == ""

replace v4 = v43 if v4 == ""

replace v4 = v46 if v4 == ""

replace v4 = v49 if v4 == ""

replace v4 = v52 if v4 == ""

replace v4 = v55 if v4 == ""

keep v1 v2 v3 v4

drop if v1 == ""

drop if v1 == "main"

drop if v1 == "_cons"

destring v1, replace ignore(file)

rename v2 Diff_IVR_IPW

rename v3 Diff_IVR_IPW_CI

rename v4 Diff_IVR_IPW_P

gen pop_group = 1

tostring v1, replace

replace v1 = "Any current tobacco user" if v1 == "1"

replace v1 = "Current smoker" if v1 == "2"

replace v1 = "Current smokeless tobacco user" if v1 == "3"

replace v1 = "Any daily tobacco user" if v1 == "4"

replace v1 = "Daily smoker" if v1 == "5"

replace v1 = "Daily smokeless tobacco user" if v1 == "6"

replace v1 = "Only smoker" if v1 == "7"

replace v1 = "Only smokeless tobacco user" if v1 == "8"

replace v1 = "Both smoker and smokeless user" if v1 == "9"

replace v1 = "Alcohol past month" if v1 == "10"

replace v1 = "No fruit or vegetables" if v1 == "11"

replace v1 = "No fruit" if v1 == "12"

replace v1 = "No veg" if v1 == "13"

replace v1 = "<5 servings of fruits-veg in a day " if v1 == "14"

replace v1 = "Add salt to food while eating" if v1 == "15"

replace v1 = "Processed food high in salt" if v1 == "16"

replace v1 = "Known raised BP/HTN" if v1 == "17"

replace v1 = "Take medication for BP/HTN" if v1 == "18"

save File_RD_IVR_IPW, replace

restore

********

preserve

recode file1 (1 = 0) (0 = 1)

keep if sex_n == 1

keep if age_cat == 0

svyset [pweight= age_sex_w]

gen file2 = file1

gen file3 = file1

gen file4 = file1

gen file5 = file1

gen file6 = file1

gen file7 = file1

gen file8 = file1

gen file9 = file1

gen file10 = file1

gen file11 = file1

gen file12 = file1

gen file13 = file1

gen file14 = file1

gen file15 = file1

gen file16 = file1

gen file17 = file1

gen file18 = file1

svy: glm current_tobacco_any file1, fam(bin) link(identity)

estimates store I1

svy: glm current_smoke_n file2, fam(bin) link(identity)

estimates store I2

svy: glm current_smokeless_n file3, fam(bin) link(identity)

estimates store I3

svy: glm daily_tobacco_any file4, fam(bin) link(identity)

estimates store I4

svy: glm daily_smoke_n file5, fam(bin) link(identity)

estimates store I5

svy: glm daily_smokeless_n file6, fam(bin) link(identity)

estimates store I6

svy: glm smoke_only file7, fam(bin) link(identity)

estimates store I7

svy: glm smokeless_only file8, fam(bin) link(identity)

estimates store I8

svy: glm both_smoke_smokeless file9, fam(bin) link(identity)

estimates store I9

svy: glm alcohol_current file10, fam(bin) link(identity)

estimates store I10

svy: glm no_fruit_veg file11, fam(bin) link(identity)

estimates store I11

svy: glm no_fruit file12, fam(bin) link(identity)

estimates store I12

svy: glm no_veg file13, fam(bin) link(identity)

estimates store I13

svy: glm atelessthan5 file14, fam(bin) link(identity)

estimates store I14

svy: glm salt_eat_n file15, fam(bin) link(identity)

estimates store I15

svy: glm salt_pack_n file16, fam(bin) link(identity)

estimates store I16

svy: glm htn_known file17, fam(bin) link(identity)

estimates store I17

svy: glm htn_med file18, fam(bin) link(identity)

estimates store I18

estout I1 I2 I3 I4 I5 I6 I7 I8 I9 I10 I11 I12 I13 I14 I15 I16 I17 I18 using Table_RD.xls, ///

cells ("b(star fmt(1)) ci(par fmt(1) label(95% CI)) p (fmt(4)) ") replace transform(@*100)

import delimited "Table_RD.xls", clear

replace v2 = v5 if v2 == ""

replace v2 = v8 if v2 == ""

replace v2 = v11 if v2 == ""

replace v2 = v14 if v2 == ""

replace v2 = v17 if v2 == ""

replace v2 = v20 if v2 == ""

replace v2 = v23 if v2 == ""

replace v2 = v26 if v2 == ""

replace v2 = v29 if v2 == ""

replace v2 = v32 if v2 == ""

replace v2 = v35 if v2 == ""

replace v2 = v38 if v2 == ""

replace v2 = v41 if v2 == ""

replace v2 = v44 if v2 == ""

replace v2 = v47 if v2 == ""

replace v2 = v50 if v2 == ""

replace v2 = v53 if v2 == ""

replace v3 = v6 if v3 == ""

replace v3 = v9 if v3 == ""

replace v3 = v12 if v3 == ""

replace v3 = v15 if v3 == ""

replace v3 = v18 if v3 == ""

replace v3 = v21 if v3 == ""

replace v3 = v24 if v3 == ""

replace v3 = v27 if v3 == ""

replace v3 = v30 if v3 == ""

replace v3 = v33 if v3 == ""

replace v3 = v36 if v3 == ""

replace v3 = v39 if v3 == ""

replace v3 = v42 if v3 == ""

replace v3 = v45 if v3 == ""

replace v3 = v48 if v3 == ""

replace v3 = v51 if v3 == ""

replace v3 = v54 if v3 == ""

replace v4 = v7 if v4 == ""

replace v4 = v10 if v4 == ""

replace v4 = v13 if v4 == ""

replace v4 = v16 if v4 == ""

replace v4 = v19 if v4 == ""

replace v4 = v22 if v4 == ""

replace v4 = v25 if v4 == ""

replace v4 = v28 if v4 == ""

replace v4 = v31 if v4 == ""

replace v4 = v34 if v4 == ""

replace v4 = v37 if v4 == ""

replace v4 = v40 if v4 == ""

replace v4 = v43 if v4 == ""

replace v4 = v46 if v4 == ""

replace v4 = v49 if v4 == ""

replace v4 = v52 if v4 == ""

replace v4 = v55 if v4 == ""

keep v1 v2 v3 v4

drop if v1 == ""

drop if v1 == "main"

drop if v1 == "_cons"

destring v1, replace ignore(file)

rename v2 Diff_IVR_Age_Sex

rename v3 Diff_IVR_Age_Sex_CI

rename v4 Diff_IVR_Age_Sex_P

gen pop_group = 1

gen number = v1

tostring v1, replace

replace v1 = "Any current tobacco user" if v1 == "1"

replace v1 = "Current smoker" if v1 == "2"

replace v1 = "Current smokeless tobacco user" if v1 == "3"

replace v1 = "Any daily tobacco user" if v1 == "4"

replace v1 = "Daily smoker" if v1 == "5"

replace v1 = "Daily smokeless tobacco user" if v1 == "6"

replace v1 = "Only smoker" if v1 == "7"

replace v1 = "Only smokeless tobacco user" if v1 == "8"

replace v1 = "Both smoker and smokeless user" if v1 == "9"

replace v1 = "Alcohol past month" if v1 == "10"

replace v1 = "No fruit or vegetables" if v1 == "11"

replace v1 = "No fruit" if v1 == "12"

replace v1 = "No veg" if v1 == "13"

replace v1 = "<5 servings of fruits-veg in a day " if v1 == "14"

replace v1 = "Add salt to food while eating" if v1 == "15"

replace v1 = "Processed food high in salt" if v1 == "16"

replace v1 = "Known raised BP/HTN" if v1 == "17"

replace v1 = "Take medication for BP/HTN" if v1 == "18"

save File_RD_IVR_Age_Sex, replace

restore

********

preserve

use Table3_Unweighted.dta, replace

merge 1:1 v1 using Table3_Weighted_IPW, nogen

merge 1:1 v1 using Table3_Weighted_Age_Sex_W.dta, nogen

merge 1:1 v1 using Table3_n.dta, nogen

sort id

drop id

label var v1 "Indicator"

drop STEPS_IPW

gen indicator = _n

gen pop_group = 5

save Table3_05_Prevalence_GMAK_20Jul2022.dta, replace

merge 1:1 v1 using File_RD_IVR_IPW.dta, nogen

merge 1:1 v1 using File_RD_IVR_Age_Sex.dta, nogen

sort number

order v1 IVR_n IVR_IPW IVR_IPW_CI STEPS_n STEPS_Age_Sex STEPS_Age_Sex_CI Diff_IVR_IPW

export excel v1 IVR_n IVR_IPW IVR_IPW_CI STEPS_n STEPS_Age_Sex STEPS_Age_Sex_CI ///

Diff_IVR_IPW using SumTab_IVR_IPWvsSTEPS.xls, replace

order v1 IVR_n IVR_Age_Sex IVR_Age_Sex_CI STEPS_n STEPS_Age_Sex STEPS_Age_Sex_CI ///

Diff_IVR_Age_Sex

export excel v1 IVR_n IVR_Age_Sex IVR_Age_Sex_CI STEPS_n STEPS_Age_Sex STEPS_Age_Sex_CI ///

Diff_IVR_Age_Sex using SumTab_IVR_AgeSexvsSTEPS.xls, replace

restore

********************************************************************************

preserve

keep if sex_n == 1

keep if age_cat == 1

tabout current_tobacco_any current_smoke_n current_smokeless_n ///

daily_tobacco_any daily_smoke_n daily_smokeless_n ///

smoke_only smokeless_only both_smoke_smokeless ///

alcohol_current no_fruit_veg no_fruit no_veg ///

atelessthan5 salt_eat_n salt_pack_n ///

htn_known htn_med file1 ///

using Table3_n.xls, ///

c(freq col) f(1c 1p) replace

import delimited using Table3_n.xls, clear

drop if v1 == "No"

drop if v1 == "Yes"

replace v1 = v1[_n - 1] if v1 == "Total"

drop v3 v5 v6 v7

drop if v1 == ""

drop if v2 == ""

destring v2 v4, ignore(",") replace

rename v2 IVR_n

rename v4 STEPS_n

gen id = _n

save Table3_n.dta, replace

restore

********

preserve

keep if sex_n == 1

keep if age_cat == 1

gen wt = 1

svyset [pweight= wt]

tabout current_tobacco_any current_smoke_n current_smokeless_n ///

daily_tobacco_any daily_smoke_n daily_smokeless_n ///

smoke_only smokeless_only both_smoke_smokeless ///

alcohol_current no_fruit_veg no_fruit no_veg ///

atelessthan5 salt_eat_n salt_pack_n ///

htn_known htn_med file1 ///

using Table3_Unweight.xls, ///

c(col ci) f(1 1) percent svy replace

import delimited using Table3_Unweight.xls, clear

drop v6 v7

drop if v1 == "No" | v1 == "Total"

replace v1 = v1[_n - 1] if v1 == "Yes"

drop if v2 == ""

drop if v1 == ""

rename v2 IVR_Unweight

rename v4 STEPS_Unweight

rename v3 IVR_Unweight_CI

rename v5 STEPS_Unweight_CI

gen id = _n

save Table3_Unweighted.dta, replace

restore

********

preserve

keep if sex_n == 1

keep if age_cat == 1

svyset [pweight= weight_ipw]

tabout current_tobacco_any current_smoke_n current_smokeless_n ///

daily_tobacco_any daily_smoke_n daily_smokeless_n ///

smoke_only smokeless_only both_smoke_smokeless ///

alcohol_current no_fruit_veg no_fruit no_veg ///

atelessthan5 salt_eat_n salt_pack_n ///

htn_known htn_med file1 ///

using Table3_Weight_IPW.xls, ///

c(col ci) f(1 1) percent svy replace

import delimited using Table3_Weight_IPW.xls, clear

drop v6 v7

drop if v1 == "No" | v1 == "Total"

replace v1 = v1[_n - 1] if v1 == "Yes"

drop if v2 == ""

drop if v1 == ""

rename v2 IVR_IPW

rename v4 STEPS_IPW

rename v3 IVR_IPW_CI

rename v5 STEPS_IPW_CI

gen id = _n

save Table3_Weighted_IPW.dta, replace

restore

********

preserve

keep if sex_n == 1

keep if age_cat == 1

svyset [pweight= age_sex_w]

tabout current_tobacco_any current_smoke_n current_smokeless_n ///

daily_tobacco_any daily_smoke_n daily_smokeless_n ///

smoke_only smokeless_only both_smoke_smokeless ///

alcohol_current no_fruit_veg no_fruit no_veg ///

atelessthan5 salt_eat_n salt_pack_n ///

htn_known htn_med file1 ///

using Table3_Weight_Age_Sex_W.xls, ///

c(col ci) f(1 1) percent svy replace

import delimited using Table3_Weight_Age_Sex_W.xls, clear

drop v6 v7

drop if v1 == "No" | v1 == "Total"

replace v1 = v1[_n - 1] if v1 == "Yes"

drop if v2 == ""

drop if v1 == ""

rename v2 IVR_Age_Sex

rename v4 STEPS_Age_Sex

rename v3 IVR_Age_Sex_CI

rename v5 STEPS_Age_Sex_CI

gen id = _n

save Table3_Weighted_Age_Sex_W.dta, replace

restore

*****

preserve

recode file1 (1 = 0) (0 = 1)

keep if sex_n == 1

keep if age_cat == 1

svyset [pweight= weight_ipw]

gen file2 = file1

gen file3 = file1

gen file4 = file1

gen file5 = file1

gen file6 = file1

gen file7 = file1

gen file8 = file1

gen file9 = file1

gen file10 = file1

gen file11 = file1

gen file12 = file1

gen file13 = file1

gen file14 = file1

gen file15 = file1

gen file16 = file1

gen file17 = file1

gen file18 = file1

svy: glm current_tobacco_any file1, fam(bin) link(identity)

estimates store I1

svy: glm current_smoke_n file2, fam(bin) link(identity)

estimates store I2

svy: glm current_smokeless_n file3, fam(bin) link(identity)

estimates store I3

svy: glm daily_tobacco_any file4, fam(bin) link(identity)

estimates store I4

svy: glm daily_smoke_n file5, fam(bin) link(identity)

estimates store I5

svy: glm daily_smokeless_n file6, fam(bin) link(identity)

estimates store I6

svy: glm smoke_only file7, fam(bin) link(identity)

estimates store I7

svy: glm smokeless_only file8, fam(bin) link(identity)

estimates store I8

svy: glm both_smoke_smokeless file9, fam(bin) link(identity)

estimates store I9

svy: glm alcohol_current file10, fam(bin) link(identity)

estimates store I10

svy: glm no_fruit_veg file11, fam(bin) link(identity)

estimates store I11

svy: glm no_fruit file12, fam(bin) link(identity)

estimates store I12

svy: glm no_veg file13, fam(bin) link(identity)

estimates store I13

svy: glm atelessthan5 file14, fam(bin) link(identity)

estimates store I14

svy: glm salt_eat_n file15, fam(bin) link(identity)

estimates store I15

svy: glm salt_pack_n file16, fam(bin) link(identity)

estimates store I16

svy: glm htn_known file17, fam(bin) link(identity)

estimates store I17

svy: glm htn_med file18, fam(bin) link(identity)

estimates store I18

estout I1 I2 I3 I4 I5 I6 I7 I8 I9 I10 I11 I12 I13 I14 I15 I16 I17 I18 using Table_RD.xls, ///

cells ("b(star fmt(1)) ci(par fmt(1) label(95% CI)) p (fmt(4)) ") replace transform(@*100)

import delimited "Table_RD.xls", clear

replace v2 = v5 if v2 == ""

replace v2 = v8 if v2 == ""

replace v2 = v11 if v2 == ""

replace v2 = v14 if v2 == ""

replace v2 = v17 if v2 == ""

replace v2 = v20 if v2 == ""

replace v2 = v23 if v2 == ""

replace v2 = v26 if v2 == ""

replace v2 = v29 if v2 == ""

replace v2 = v32 if v2 == ""

replace v2 = v35 if v2 == ""

replace v2 = v38 if v2 == ""

replace v2 = v41 if v2 == ""

replace v2 = v44 if v2 == ""

replace v2 = v47 if v2 == ""

replace v2 = v50 if v2 == ""

replace v2 = v53 if v2 == ""

replace v3 = v6 if v3 == ""

replace v3 = v9 if v3 == ""

replace v3 = v12 if v3 == ""

replace v3 = v15 if v3 == ""

replace v3 = v18 if v3 == ""

replace v3 = v21 if v3 == ""

replace v3 = v24 if v3 == ""

replace v3 = v27 if v3 == ""

replace v3 = v30 if v3 == ""

replace v3 = v33 if v3 == ""

replace v3 = v36 if v3 == ""

replace v3 = v39 if v3 == ""

replace v3 = v42 if v3 == ""

replace v3 = v45 if v3 == ""

replace v3 = v48 if v3 == ""

replace v3 = v51 if v3 == ""

replace v3 = v54 if v3 == ""

replace v4 = v7 if v4 == ""

replace v4 = v10 if v4 == ""

replace v4 = v13 if v4 == ""

replace v4 = v16 if v4 == ""

replace v4 = v19 if v4 == ""

replace v4 = v22 if v4 == ""

replace v4 = v25 if v4 == ""

replace v4 = v28 if v4 == ""

replace v4 = v31 if v4 == ""

replace v4 = v34 if v4 == ""

replace v4 = v37 if v4 == ""

replace v4 = v40 if v4 == ""

replace v4 = v43 if v4 == ""

replace v4 = v46 if v4 == ""

replace v4 = v49 if v4 == ""

replace v4 = v52 if v4 == ""

replace v4 = v55 if v4 == ""

keep v1 v2 v3 v4

drop if v1 == ""

drop if v1 == "main"

drop if v1 == "_cons"

destring v1, replace ignore(file)

rename v2 Diff_IVR_IPW

rename v3 Diff_IVR_IPW_CI

rename v4 Diff_IVR_IPW_P

gen pop_group = 1

tostring v1, replace

replace v1 = "Any current tobacco user" if v1 == "1"

replace v1 = "Current smoker" if v1 == "2"

replace v1 = "Current smokeless tobacco user" if v1 == "3"

replace v1 = "Any daily tobacco user" if v1 == "4"

replace v1 = "Daily smoker" if v1 == "5"

replace v1 = "Daily smokeless tobacco user" if v1 == "6"

replace v1 = "Only smoker" if v1 == "7"

replace v1 = "Only smokeless tobacco user" if v1 == "8"

replace v1 = "Both smoker and smokeless user" if v1 == "9"

replace v1 = "Alcohol past month" if v1 == "10"

replace v1 = "No fruit or vegetables" if v1 == "11"

replace v1 = "No fruit" if v1 == "12"

replace v1 = "No veg" if v1 == "13"

replace v1 = "<5 servings of fruits-veg in a day " if v1 == "14"

replace v1 = "Add salt to food while eating" if v1 == "15"

replace v1 = "Processed food high in salt" if v1 == "16"

replace v1 = "Known raised BP/HTN" if v1 == "17"

replace v1 = "Take medication for BP/HTN" if v1 == "18"

save File_RD_IVR_IPW, replace

restore

********

preserve

recode file1 (1 = 0) (0 = 1)

keep if sex_n == 1

keep if age_cat == 1

svyset [pweight= age_sex_w]

gen file2 = file1

gen file3 = file1

gen file4 = file1

gen file5 = file1

gen file6 = file1

gen file7 = file1

gen file8 = file1

gen file9 = file1

gen file10 = file1

gen file11 = file1

gen file12 = file1

gen file13 = file1

gen file14 = file1

gen file15 = file1

gen file16 = file1

gen file17 = file1

gen file18 = file1

svy: glm current_tobacco_any file1, fam(bin) link(identity)

estimates store I1

svy: glm current_smoke_n file2, fam(bin) link(identity)

estimates store I2

svy: glm current_smokeless_n file3, fam(bin) link(identity)

estimates store I3

svy: glm daily_tobacco_any file4, fam(bin) link(identity)

estimates store I4

svy: glm daily_smoke_n file5, fam(bin) link(identity)

estimates store I5

svy: glm daily_smokeless_n file6, fam(bin) link(identity)

estimates store I6

svy: glm smoke_only file7, fam(bin) link(identity)

estimates store I7

svy: glm smokeless_only file8, fam(bin) link(identity)

estimates store I8

svy: glm both_smoke_smokeless file9, fam(bin) link(identity)

estimates store I9

svy: glm alcohol_current file10, fam(bin) link(identity)

estimates store I10

svy: glm no_fruit_veg file11, fam(bin) link(identity)

estimates store I11

svy: glm no_fruit file12, fam(bin) link(identity)

estimates store I12

svy: glm no_veg file13, fam(bin) link(identity)

estimates store I13

svy: glm atelessthan5 file14, fam(bin) link(identity)

estimates store I14

svy: glm salt_eat_n file15, fam(bin) link(identity)

estimates store I15

svy: glm salt_pack_n file16, fam(bin) link(identity)

estimates store I16

svy: glm htn_known file17, fam(bin) link(identity)

estimates store I17

svy: glm htn_med file18, fam(bin) link(identity)

estimates store I18

estout I1 I2 I3 I4 I5 I6 I7 I8 I9 I10 I11 I12 I13 I14 I15 I16 I17 I18 using Table_RD.xls, ///

cells ("b(star fmt(1)) ci(par fmt(1) label(95% CI)) p (fmt(4)) ") replace transform(@*100)

import delimited "Table_RD.xls", clear

replace v2 = v5 if v2 == ""

replace v2 = v8 if v2 == ""

replace v2 = v11 if v2 == ""

replace v2 = v14 if v2 == ""

replace v2 = v17 if v2 == ""

replace v2 = v20 if v2 == ""

replace v2 = v23 if v2 == ""

replace v2 = v26 if v2 == ""

replace v2 = v29 if v2 == ""

replace v2 = v32 if v2 == ""

replace v2 = v35 if v2 == ""

replace v2 = v38 if v2 == ""

replace v2 = v41 if v2 == ""

replace v2 = v44 if v2 == ""

replace v2 = v47 if v2 == ""

replace v2 = v50 if v2 == ""

replace v2 = v53 if v2 == ""

replace v3 = v6 if v3 == ""

replace v3 = v9 if v3 == ""

replace v3 = v12 if v3 == ""

replace v3 = v15 if v3 == ""

replace v3 = v18 if v3 == ""

replace v3 = v21 if v3 == ""

replace v3 = v24 if v3 == ""

replace v3 = v27 if v3 == ""

replace v3 = v30 if v3 == ""

replace v3 = v33 if v3 == ""

replace v3 = v36 if v3 == ""

replace v3 = v39 if v3 == ""

replace v3 = v42 if v3 == ""

replace v3 = v45 if v3 == ""

replace v3 = v48 if v3 == ""

replace v3 = v51 if v3 == ""

replace v3 = v54 if v3 == ""

replace v4 = v7 if v4 == ""

replace v4 = v10 if v4 == ""

replace v4 = v13 if v4 == ""

replace v4 = v16 if v4 == ""

replace v4 = v19 if v4 == ""

replace v4 = v22 if v4 == ""

replace v4 = v25 if v4 == ""

replace v4 = v28 if v4 == ""

replace v4 = v31 if v4 == ""

replace v4 = v34 if v4 == ""

replace v4 = v37 if v4 == ""

replace v4 = v40 if v4 == ""

replace v4 = v43 if v4 == ""

replace v4 = v46 if v4 == ""

replace v4 = v49 if v4 == ""

replace v4 = v52 if v4 == ""

replace v4 = v55 if v4 == ""

keep v1 v2 v3 v4

drop if v1 == ""

drop if v1 == "main"

drop if v1 == "_cons"

destring v1, replace ignore(file)

rename v2 Diff_IVR_Age_Sex

rename v3 Diff_IVR_Age_Sex_CI

rename v4 Diff_IVR_Age_Sex_P

gen pop_group = 1

gen number = v1

tostring v1, replace

replace v1 = "Any current tobacco user" if v1 == "1"

replace v1 = "Current smoker" if v1 == "2"

replace v1 = "Current smokeless tobacco user" if v1 == "3"

replace v1 = "Any daily tobacco user" if v1 == "4"

replace v1 = "Daily smoker" if v1 == "5"

replace v1 = "Daily smokeless tobacco user" if v1 == "6"

replace v1 = "Only smoker" if v1 == "7"

replace v1 = "Only smokeless tobacco user" if v1 == "8"

replace v1 = "Both smoker and smokeless user" if v1 == "9"

replace v1 = "Alcohol past month" if v1 == "10"

replace v1 = "No fruit or vegetables" if v1 == "11"

replace v1 = "No fruit" if v1 == "12"

replace v1 = "No veg" if v1 == "13"

replace v1 = "<5 servings of fruits-veg in a day " if v1 == "14"

replace v1 = "Add salt to food while eating" if v1 == "15"

replace v1 = "Processed food high in salt" if v1 == "16"

replace v1 = "Known raised BP/HTN" if v1 == "17"

replace v1 = "Take medication for BP/HTN" if v1 == "18"

save File_RD_IVR_Age_Sex, replace

restore

********

preserve

use Table3_Unweighted.dta, replace

merge 1:1 v1 using Table3_Weighted_IPW, nogen

merge 1:1 v1 using Table3_Weighted_Age_Sex_W.dta, nogen

merge 1:1 v1 using Table3_n.dta, nogen

sort id

drop id

label var v1 "Indicator"

drop STEPS_IPW

gen indicator = _n

gen pop_group = 6

save Table3_06_Prevalence_GMAK_20Jul2022.dta, replace

merge 1:1 v1 using File_RD_IVR_IPW.dta, nogen

merge 1:1 v1 using File_RD_IVR_Age_Sex.dta, nogen

sort number

order v1 IVR_n IVR_IPW IVR_IPW_CI STEPS_n STEPS_Age_Sex STEPS_Age_Sex_CI Diff_IVR_IPW

export excel v1 IVR_n IVR_IPW IVR_IPW_CI STEPS_n STEPS_Age_Sex STEPS_Age_Sex_CI ///

Diff_IVR_IPW using SumTab_IVR_IPWvsSTEPS.xls, replace

order v1 IVR_n IVR_Age_Sex IVR_Age_Sex_CI STEPS_n STEPS_Age_Sex STEPS_Age_Sex_CI ///

Diff_IVR_Age_Sex

export excel v1 IVR_n IVR_Age_Sex IVR_Age_Sex_CI STEPS_n STEPS_Age_Sex STEPS_Age_Sex_CI ///

Diff_IVR_Age_Sex using SumTab_IVR_AgeSexvsSTEPS.xls, replace

restore

********************************************************************************

preserve

keep if sex_n == 1

keep if age_cat == 2

tabout current_tobacco_any current_smoke_n current_smokeless_n ///

daily_tobacco_any daily_smoke_n daily_smokeless_n ///

smoke_only smokeless_only both_smoke_smokeless ///

alcohol_current no_fruit_veg no_fruit no_veg ///

atelessthan5 salt_eat_n salt_pack_n ///

htn_known htn_med file1 ///

using Table3_n.xls, ///

c(freq col) f(1c 1p) replace

import delimited using Table3_n.xls, clear

drop if v1 == "No"

drop if v1 == "Yes"

replace v1 = v1[_n - 1] if v1 == "Total"

drop v3 v5 v6 v7

drop if v1 == ""

drop if v2 == ""

destring v2 v4, ignore(",") replace

rename v2 IVR_n

rename v4 STEPS_n

gen id = _n

save Table3_n.dta, replace

restore

preserve

keep if sex_n == 1

keep if age_cat == 2

gen wt = 1

svyset [pweight= wt]

tabout current_tobacco_any current_smoke_n current_smokeless_n ///

daily_tobacco_any daily_smoke_n daily_smokeless_n ///

smoke_only smokeless_only both_smoke_smokeless ///

alcohol_current no_fruit_veg no_fruit no_veg ///

atelessthan5 salt_eat_n salt_pack_n ///

htn_known htn_med file1 ///

using Table3_Unweight.xls, ///

c(col ci) f(1 1) percent svy replace

import delimited using Table3_Unweight.xls, clear

drop v6 v7

drop if v1 == "No" | v1 == "Total"

replace v1 = v1[_n - 1] if v1 == "Yes"

drop if v2 == ""

drop if v1 == ""

rename v2 IVR_Unweight

rename v4 STEPS_Unweight

rename v3 IVR_Unweight_CI

rename v5 STEPS_Unweight_CI

gen id = _n

save Table3_Unweighted.dta, replace

restore

********

preserve

keep if sex_n == 1

keep if age_cat == 2

svyset [pweight= weight_ipw]

tabout current_tobacco_any current_smoke_n current_smokeless_n ///

daily_tobacco_any daily_smoke_n daily_smokeless_n ///

smoke_only smokeless_only both_smoke_smokeless ///

alcohol_current no_fruit_veg no_fruit no_veg ///

atelessthan5 salt_eat_n salt_pack_n ///

htn_known htn_med file1 ///

using Table3_Weight_IPW.xls, ///

c(col ci) f(1 1) percent svy replace

import delimited using Table3_Weight_IPW.xls, clear

drop v6 v7

drop if v1 == "No" | v1 == "Total"

replace v1 = v1[_n - 1] if v1 == "Yes"

drop if v2 == ""

drop if v1 == ""

rename v2 IVR_IPW

rename v4 STEPS_IPW

rename v3 IVR_IPW_CI

rename v5 STEPS_IPW_CI

gen id = _n

save Table3_Weighted_IPW.dta, replace

restore

********

preserve

keep if sex_n == 1

keep if age_cat == 2

svyset [pweight= age_sex_w]

tabout current_tobacco_any current_smoke_n current_smokeless_n ///

daily_tobacco_any daily_smoke_n daily_smokeless_n ///

smoke_only smokeless_only both_smoke_smokeless ///

alcohol_current no_fruit_veg no_fruit no_veg ///

atelessthan5 salt_eat_n salt_pack_n ///

htn_known htn_med file1 ///

using Table3_Weight_Age_Sex_W.xls, ///

c(col ci) f(1 1) percent svy replace

import delimited using Table3_Weight_Age_Sex_W.xls, clear

drop v6 v7

drop if v1 == "No" | v1 == "Total"

replace v1 = v1[_n - 1] if v1 == "Yes"

drop if v2 == ""

drop if v1 == ""

rename v2 IVR_Age_Sex

rename v4 STEPS_Age_Sex

rename v3 IVR_Age_Sex_CI

rename v5 STEPS_Age_Sex_CI

gen id = _n

save Table3_Weighted_Age_Sex_W.dta, replace

restore

*****

preserve

recode file1 (1 = 0) (0 = 1)

keep if sex_n == 1

keep if age_cat == 2

svyset [pweight= weight_ipw]

gen file2 = file1

gen file3 = file1

gen file4 = file1

gen file5 = file1

gen file6 = file1

gen file7 = file1

gen file8 = file1

gen file9 = file1

gen file10 = file1

gen file11 = file1

gen file12 = file1

gen file13 = file1

gen file14 = file1

gen file15 = file1

gen file16 = file1

gen file17 = file1

gen file18 = file1

svy: glm current_tobacco_any file1, fam(bin) link(identity)

estimates store I1

svy: glm current_smoke_n file2, fam(bin) link(identity)

estimates store I2

svy: glm current_smokeless_n file3, fam(bin) link(identity)

estimates store I3

svy: glm daily_tobacco_any file4, fam(bin) link(identity)

estimates store I4

svy: glm daily_smoke_n file5, fam(bin) link(identity)

estimates store I5

svy: glm daily_smokeless_n file6, fam(bin) link(identity)

estimates store I6

svy: glm smoke_only file7, fam(bin) link(identity)

estimates store I7

svy: glm smokeless_only file8, fam(bin) link(identity)

estimates store I8

svy: glm both_smoke_smokeless file9, fam(bin) link(identity)

estimates store I9

svy: glm alcohol_current file10, fam(bin) link(identity)

estimates store I10

svy: glm no_fruit_veg file11, fam(bin) link(identity)

estimates store I11

svy: glm no_fruit file12, fam(bin) link(identity)

estimates store I12

svy: glm no_veg file13, fam(bin) link(identity)

estimates store I13

svy: glm atelessthan5 file14, fam(bin) link(identity)

estimates store I14

svy: glm salt_eat_n file15, fam(bin) link(identity)

estimates store I15

svy: glm salt_pack_n file16, fam(bin) link(identity)

estimates store I16

svy: glm htn_known file17, fam(bin) link(identity)

estimates store I17

svy: glm htn_med file18, fam(bin) link(identity)

estimates store I18

estout I1 I2 I3 I4 I5 I6 I7 I8 I9 I10 I11 I12 I13 I14 I15 I16 I17 I18 using Table_RD.xls, ///

cells ("b(star fmt(1)) ci(par fmt(1) label(95% CI)) p (fmt(4)) ") replace transform(@*100)

import delimited "Table_RD.xls", clear

replace v2 = v5 if v2 == ""

replace v2 = v8 if v2 == ""

replace v2 = v11 if v2 == ""

replace v2 = v14 if v2 == ""

replace v2 = v17 if v2 == ""

replace v2 = v20 if v2 == ""

replace v2 = v23 if v2 == ""

replace v2 = v26 if v2 == ""

replace v2 = v29 if v2 == ""

replace v2 = v32 if v2 == ""

replace v2 = v35 if v2 == ""

replace v2 = v38 if v2 == ""

replace v2 = v41 if v2 == ""

replace v2 = v44 if v2 == ""

replace v2 = v47 if v2 == ""

replace v2 = v50 if v2 == ""

replace v2 = v53 if v2 == ""

replace v3 = v6 if v3 == ""

replace v3 = v9 if v3 == ""

replace v3 = v12 if v3 == ""

replace v3 = v15 if v3 == ""

replace v3 = v18 if v3 == ""

replace v3 = v21 if v3 == ""

replace v3 = v24 if v3 == ""

replace v3 = v27 if v3 == ""

replace v3 = v30 if v3 == ""

replace v3 = v33 if v3 == ""

replace v3 = v36 if v3 == ""

replace v3 = v39 if v3 == ""

replace v3 = v42 if v3 == ""

replace v3 = v45 if v3 == ""

replace v3 = v48 if v3 == ""

replace v3 = v51 if v3 == ""

replace v3 = v54 if v3 == ""

replace v4 = v7 if v4 == ""

replace v4 = v10 if v4 == ""

replace v4 = v13 if v4 == ""

replace v4 = v16 if v4 == ""

replace v4 = v19 if v4 == ""

replace v4 = v22 if v4 == ""

replace v4 = v25 if v4 == ""

replace v4 = v28 if v4 == ""

replace v4 = v31 if v4 == ""

replace v4 = v34 if v4 == ""

replace v4 = v37 if v4 == ""

replace v4 = v40 if v4 == ""

replace v4 = v43 if v4 == ""

replace v4 = v46 if v4 == ""

replace v4 = v49 if v4 == ""

replace v4 = v52 if v4 == ""

replace v4 = v55 if v4 == ""

keep v1 v2 v3 v4

drop if v1 == ""

drop if v1 == "main"

drop if v1 == "_cons"

destring v1, replace ignore(file)

rename v2 Diff_IVR_IPW

rename v3 Diff_IVR_IPW_CI

rename v4 Diff_IVR_IPW_P

gen pop_group = 1

tostring v1, replace

replace v1 = "Any current tobacco user" if v1 == "1"

replace v1 = "Current smoker" if v1 == "2"

replace v1 = "Current smokeless tobacco user" if v1 == "3"

replace v1 = "Any daily tobacco user" if v1 == "4"

replace v1 = "Daily smoker" if v1 == "5"

replace v1 = "Daily smokeless tobacco user" if v1 == "6"

replace v1 = "Only smoker" if v1 == "7"

replace v1 = "Only smokeless tobacco user" if v1 == "8"

replace v1 = "Both smoker and smokeless user" if v1 == "9"

replace v1 = "Alcohol past month" if v1 == "10"

replace v1 = "No fruit or vegetables" if v1 == "11"

replace v1 = "No fruit" if v1 == "12"

replace v1 = "No veg" if v1 == "13"

replace v1 = "<5 servings of fruits-veg in a day " if v1 == "14"

replace v1 = "Add salt to food while eating" if v1 == "15"

replace v1 = "Processed food high in salt" if v1 == "16"

replace v1 = "Known raised BP/HTN" if v1 == "17"

replace v1 = "Take medication for BP/HTN" if v1 == "18"

save File_RD_IVR_IPW, replace

restore

********

preserve

recode file1 (1 = 0) (0 = 1)

keep if sex_n == 1

keep if age_cat == 2

svyset [pweight= age_sex_w]

gen file2 = file1

gen file3 = file1

gen file4 = file1

gen file5 = file1

gen file6 = file1

gen file7 = file1

gen file8 = file1

gen file9 = file1

gen file10 = file1

gen file11 = file1

gen file12 = file1

gen file13 = file1

gen file14 = file1

gen file15 = file1

gen file16 = file1

gen file17 = file1

gen file18 = file1

svy: glm current_tobacco_any file1, fam(bin) link(identity)

estimates store I1

svy: glm current_smoke_n file2, fam(bin) link(identity)

estimates store I2

svy: glm current_smokeless_n file3, fam(bin) link(identity)

estimates store I3

svy: glm daily_tobacco_any file4, fam(bin) link(identity)

estimates store I4

svy: glm daily_smoke_n file5, fam(bin) link(identity)

estimates store I5

svy: glm daily_smokeless_n file6, fam(bin) link(identity)

estimates store I6

svy: glm smoke_only file7, fam(bin) link(identity)

estimates store I7

svy: glm smokeless_only file8, fam(bin) link(identity)

estimates store I8

svy: glm both_smoke_smokeless file9, fam(bin) link(identity)

estimates store I9

svy: glm alcohol_current file10, fam(bin) link(identity)

estimates store I10

svy: glm no_fruit_veg file11, fam(bin) link(identity)

estimates store I11

svy: glm no_fruit file12, fam(bin) link(identity)

estimates store I12

svy: glm no_veg file13, fam(bin) link(identity)

estimates store I13

svy: glm atelessthan5 file14, fam(bin) link(identity)

estimates store I14

svy: glm salt_eat_n file15, fam(bin) link(identity)

estimates store I15

svy: glm salt_pack_n file16, fam(bin) link(identity)

estimates store I16

svy: glm htn_known file17, fam(bin) link(identity)

estimates store I17

svy: glm htn_med file18, fam(bin) link(identity)

estimates store I18

estout I1 I2 I3 I4 I5 I6 I7 I8 I9 I10 I11 I12 I13 I14 I15 I16 I17 I18 using Table_RD.xls, ///

cells ("b(star fmt(1)) ci(par fmt(1) label(95% CI)) p (fmt(4)) ") replace transform(@*100)

import delimited "Table_RD.xls", clear

replace v2 = v5 if v2 == ""

replace v2 = v8 if v2 == ""

replace v2 = v11 if v2 == ""

replace v2 = v14 if v2 == ""

replace v2 = v17 if v2 == ""

replace v2 = v20 if v2 == ""

replace v2 = v23 if v2 == ""

replace v2 = v26 if v2 == ""

replace v2 = v29 if v2 == ""

replace v2 = v32 if v2 == ""

replace v2 = v35 if v2 == ""

replace v2 = v38 if v2 == ""

replace v2 = v41 if v2 == ""

replace v2 = v44 if v2 == ""

replace v2 = v47 if v2 == ""

replace v2 = v50 if v2 == ""

replace v2 = v53 if v2 == ""

replace v3 = v6 if v3 == ""

replace v3 = v9 if v3 == ""

replace v3 = v12 if v3 == ""

replace v3 = v15 if v3 == ""

replace v3 = v18 if v3 == ""

replace v3 = v21 if v3 == ""

replace v3 = v24 if v3 == ""

replace v3 = v27 if v3 == ""

replace v3 = v30 if v3 == ""

replace v3 = v33 if v3 == ""

replace v3 = v36 if v3 == ""

replace v3 = v39 if v3 == ""

replace v3 = v42 if v3 == ""

replace v3 = v45 if v3 == ""

replace v3 = v48 if v3 == ""

replace v3 = v51 if v3 == ""

replace v3 = v54 if v3 == ""

replace v4 = v7 if v4 == ""

replace v4 = v10 if v4 == ""

replace v4 = v13 if v4 == ""

replace v4 = v16 if v4 == ""

replace v4 = v19 if v4 == ""

replace v4 = v22 if v4 == ""

replace v4 = v25 if v4 == ""

replace v4 = v28 if v4 == ""

replace v4 = v31 if v4 == ""

replace v4 = v34 if v4 == ""

replace v4 = v37 if v4 == ""

replace v4 = v40 if v4 == ""

replace v4 = v43 if v4 == ""

replace v4 = v46 if v4 == ""

replace v4 = v49 if v4 == ""

replace v4 = v52 if v4 == ""

replace v4 = v55 if v4 == ""

keep v1 v2 v3 v4

drop if v1 == ""

drop if v1 == "main"

drop if v1 == "_cons"

destring v1, replace ignore(file)

rename v2 Diff_IVR_Age_Sex

rename v3 Diff_IVR_Age_Sex_CI

rename v4 Diff_IVR_Age_Sex_P

gen pop_group = 1

gen number = v1

tostring v1, replace

replace v1 = "Any current tobacco user" if v1 == "1"

replace v1 = "Current smoker" if v1 == "2"

replace v1 = "Current smokeless tobacco user" if v1 == "3"

replace v1 = "Any daily tobacco user" if v1 == "4"

replace v1 = "Daily smoker" if v1 == "5"

replace v1 = "Daily smokeless tobacco user" if v1 == "6"

replace v1 = "Only smoker" if v1 == "7"

replace v1 = "Only smokeless tobacco user" if v1 == "8"

replace v1 = "Both smoker and smokeless user" if v1 == "9"

replace v1 = "Alcohol past month" if v1 == "10"

replace v1 = "No fruit or vegetables" if v1 == "11"

replace v1 = "No fruit" if v1 == "12"

replace v1 = "No veg" if v1 == "13"

replace v1 = "<5 servings of fruits-veg in a day " if v1 == "14"

replace v1 = "Add salt to food while eating" if v1 == "15"

replace v1 = "Processed food high in salt" if v1 == "16"

replace v1 = "Known raised BP/HTN" if v1 == "17"

replace v1 = "Take medication for BP/HTN" if v1 == "18"

save File_RD_IVR_Age_Sex, replace

restore

********

preserve

use Table3_Unweighted.dta, replace

merge 1:1 v1 using Table3_Weighted_IPW, nogen

merge 1:1 v1 using Table3_Weighted_Age_Sex_W.dta, nogen

merge 1:1 v1 using Table3_n.dta, nogen

sort id

drop id

label var v1 "Indicator"

drop STEPS_IPW

gen indicator = _n

gen pop_group = 7

save Table3_07_Prevalence_GMAK_20Jul2022.dta, replace

merge 1:1 v1 using File_RD_IVR_IPW.dta, nogen

merge 1:1 v1 using File_RD_IVR_Age_Sex.dta, nogen

sort number

order v1 IVR_n IVR_IPW IVR_IPW_CI STEPS_n STEPS_Age_Sex STEPS_Age_Sex_CI Diff_IVR_IPW

export excel v1 IVR_n IVR_IPW IVR_IPW_CI STEPS_n STEPS_Age_Sex STEPS_Age_Sex_CI ///

Diff_IVR_IPW using SumTab_IVR_IPWvsSTEPS.xls, replace

order v1 IVR_n IVR_Age_Sex IVR_Age_Sex_CI STEPS_n STEPS_Age_Sex STEPS_Age_Sex_CI ///

Diff_IVR_Age_Sex

export excel v1 IVR_n IVR_Age_Sex IVR_Age_Sex_CI STEPS_n STEPS_Age_Sex STEPS_Age_Sex_CI ///

Diff_IVR_Age_Sex using SumTab_IVR_AgeSexvsSTEPS.xls, replace

restore

********************************************************************************

preserve

keep if sex_n == 1

tabout current_tobacco_any current_smoke_n current_smokeless_n ///

daily_tobacco_any daily_smoke_n daily_smokeless_n ///

smoke_only smokeless_only both_smoke_smokeless ///

alcohol_current no_fruit_veg no_fruit no_veg ///

atelessthan5 salt_eat_n salt_pack_n ///

htn_known htn_med file1 ///

using Table3_n.xls, ///

c(freq col) f(1c 1p) replace

import delimited using Table3_n.xls, clear

drop if v1 == "No"

drop if v1 == "Yes"

replace v1 = v1[_n - 1] if v1 == "Total"

drop v3 v5 v6 v7

drop if v1 == ""

drop if v2 == ""

destring v2 v4, ignore(",") replace

rename v2 IVR_n

rename v4 STEPS_n

gen id = _n

save Table3_n.dta, replace

restore

preserve

keep if sex_n == 1

gen wt = 1

svyset [pweight= wt]

tabout current_tobacco_any current_smoke_n current_smokeless_n ///

daily_tobacco_any daily_smoke_n daily_smokeless_n ///

smoke_only smokeless_only both_smoke_smokeless ///

alcohol_current no_fruit_veg no_fruit no_veg ///

atelessthan5 salt_eat_n salt_pack_n ///

htn_known htn_med file1 ///

using Table3_Unweight.xls, ///

c(col ci) f(1 1) percent svy replace

import delimited using Table3_Unweight.xls, clear

drop v6 v7

drop if v1 == "No" | v1 == "Total"

replace v1 = v1[_n - 1] if v1 == "Yes"

drop if v2 == ""

drop if v1 == ""

rename v2 IVR_Unweight

rename v4 STEPS_Unweight

rename v3 IVR_Unweight_CI

rename v5 STEPS_Unweight_CI

gen id = _n

save Table3_Unweighted.dta, replace

restore

********

preserve

keep if sex_n == 1

svyset [pweight= weight_ipw]

tabout current_tobacco_any current_smoke_n current_smokeless_n ///

daily_tobacco_any daily_smoke_n daily_smokeless_n ///

smoke_only smokeless_only both_smoke_smokeless ///

alcohol_current no_fruit_veg no_fruit no_veg ///

atelessthan5 salt_eat_n salt_pack_n ///

htn_known htn_med file1 ///

using Table3_Weight_IPW.xls, ///

c(col ci) f(1 1) percent svy replace

import delimited using Table3_Weight_IPW.xls, clear

drop v6 v7

drop if v1 == "No" | v1 == "Total"

replace v1 = v1[_n - 1] if v1 == "Yes"

drop if v2 == ""

drop if v1 == ""

rename v2 IVR_IPW

rename v4 STEPS_IPW

rename v3 IVR_IPW_CI

rename v5 STEPS_IPW_CI

gen id = _n

save Table3_Weighted_IPW.dta, replace

restore

********

preserve

keep if sex_n == 1

svyset [pweight= age_sex_w]

tabout current_tobacco_any current_smoke_n current_smokeless_n ///

daily_tobacco_any daily_smoke_n daily_smokeless_n ///

smoke_only smokeless_only both_smoke_smokeless ///

alcohol_current no_fruit_veg no_fruit no_veg ///

atelessthan5 salt_eat_n salt_pack_n ///

htn_known htn_med file1 ///

using Table3_Weight_Age_Sex_W.xls, ///

c(col ci) f(1 1) percent svy replace

import delimited using Table3_Weight_Age_Sex_W.xls, clear

drop v6 v7

drop if v1 == "No" | v1 == "Total"

replace v1 = v1[_n - 1] if v1 == "Yes"

drop if v2 == ""

drop if v1 == ""

rename v2 IVR_Age_Sex

rename v4 STEPS_Age_Sex

rename v3 IVR_Age_Sex_CI

rename v5 STEPS_Age_Sex_CI

gen id = _n

save Table3_Weighted_Age_Sex_W.dta, replace

restore

********

preserve

recode file1 (1 = 0) (0 = 1)

keep if sex_n == 1

svyset [pweight= weight_ipw]

gen file2 = file1

gen file3 = file1

gen file4 = file1

gen file5 = file1

gen file6 = file1

gen file7 = file1

gen file8 = file1

gen file9 = file1

gen file10 = file1

gen file11 = file1

gen file12 = file1

gen file13 = file1

gen file14 = file1

gen file15 = file1

gen file16 = file1

gen file17 = file1

gen file18 = file1

svy: glm current_tobacco_any file1, fam(bin) link(identity)

estimates store I1

svy: glm current_smoke_n file2, fam(bin) link(identity)

estimates store I2

svy: glm current_smokeless_n file3, fam(bin) link(identity)

estimates store I3

svy: glm daily_tobacco_any file4, fam(bin) link(identity)

estimates store I4

svy: glm daily_smoke_n file5, fam(bin) link(identity)

estimates store I5

svy: glm daily_smokeless_n file6, fam(bin) link(identity)

estimates store I6

svy: glm smoke_only file7, fam(bin) link(identity)

estimates store I7

svy: glm smokeless_only file8, fam(bin) link(identity)

estimates store I8

svy: glm both_smoke_smokeless file9, fam(bin) link(identity)

estimates store I9

svy: glm alcohol_current file10, fam(bin) link(identity)

estimates store I10

svy: glm no_fruit_veg file11, fam(bin) link(identity)

estimates store I11

svy: glm no_fruit file12, fam(bin) link(identity)

estimates store I12

svy: glm no_veg file13, fam(bin) link(identity)

estimates store I13

svy: glm atelessthan5 file14, fam(bin) link(identity)

estimates store I14

svy: glm salt_eat_n file15, fam(bin) link(identity)

estimates store I15

svy: glm salt_pack_n file16, fam(bin) link(identity)

estimates store I16

svy: glm htn_known file17, fam(bin) link(identity)

estimates store I17

svy: glm htn_med file18, fam(bin) link(identity)

estimates store I18

estout I1 I2 I3 I4 I5 I6 I7 I8 I9 I10 I11 I12 I13 I14 I15 I16 I17 I18 using Table_RD.xls, ///

cells ("b(star fmt(1)) ci(par fmt(1) label(95% CI)) p (fmt(4)) ") replace transform(@*100)

import delimited "Table_RD.xls", clear

replace v2 = v5 if v2 == ""

replace v2 = v8 if v2 == ""

replace v2 = v11 if v2 == ""

replace v2 = v14 if v2 == ""

replace v2 = v17 if v2 == ""

replace v2 = v20 if v2 == ""

replace v2 = v23 if v2 == ""

replace v2 = v26 if v2 == ""

replace v2 = v29 if v2 == ""

replace v2 = v32 if v2 == ""

replace v2 = v35 if v2 == ""

replace v2 = v38 if v2 == ""

replace v2 = v41 if v2 == ""

replace v2 = v44 if v2 == ""

replace v2 = v47 if v2 == ""

replace v2 = v50 if v2 == ""

replace v2 = v53 if v2 == ""

replace v3 = v6 if v3 == ""

replace v3 = v9 if v3 == ""

replace v3 = v12 if v3 == ""

replace v3 = v15 if v3 == ""

replace v3 = v18 if v3 == ""

replace v3 = v21 if v3 == ""

replace v3 = v24 if v3 == ""

replace v3 = v27 if v3 == ""

replace v3 = v30 if v3 == ""

replace v3 = v33 if v3 == ""

replace v3 = v36 if v3 == ""

replace v3 = v39 if v3 == ""

replace v3 = v42 if v3 == ""

replace v3 = v45 if v3 == ""

replace v3 = v48 if v3 == ""

replace v3 = v51 if v3 == ""

replace v3 = v54 if v3 == ""

replace v4 = v7 if v4 == ""

replace v4 = v10 if v4 == ""

replace v4 = v13 if v4 == ""

replace v4 = v16 if v4 == ""

replace v4 = v19 if v4 == ""

replace v4 = v22 if v4 == ""

replace v4 = v25 if v4 == ""

replace v4 = v28 if v4 == ""

replace v4 = v31 if v4 == ""

replace v4 = v34 if v4 == ""

replace v4 = v37 if v4 == ""

replace v4 = v40 if v4 == ""

replace v4 = v43 if v4 == ""

replace v4 = v46 if v4 == ""

replace v4 = v49 if v4 == ""

replace v4 = v52 if v4 == ""

replace v4 = v55 if v4 == ""

keep v1 v2 v3 v4

drop if v1 == ""

drop if v1 == "main"

drop if v1 == "_cons"

destring v1, replace ignore(file)

rename v2 Diff_IVR_IPW

rename v3 Diff_IVR_IPW_CI

rename v4 Diff_IVR_IPW_P

gen pop_group = 1

tostring v1, replace

replace v1 = "Any current tobacco user" if v1 == "1"

replace v1 = "Current smoker" if v1 == "2"

replace v1 = "Current smokeless tobacco user" if v1 == "3"

replace v1 = "Any daily tobacco user" if v1 == "4"

replace v1 = "Daily smoker" if v1 == "5"

replace v1 = "Daily smokeless tobacco user" if v1 == "6"

replace v1 = "Only smoker" if v1 == "7"

replace v1 = "Only smokeless tobacco user" if v1 == "8"

replace v1 = "Both smoker and smokeless user" if v1 == "9"

replace v1 = "Alcohol past month" if v1 == "10"

replace v1 = "No fruit or vegetables" if v1 == "11"

replace v1 = "No fruit" if v1 == "12"

replace v1 = "No veg" if v1 == "13"

replace v1 = "<5 servings of fruits-veg in a day " if v1 == "14"

replace v1 = "Add salt to food while eating" if v1 == "15"

replace v1 = "Processed food high in salt" if v1 == "16"

replace v1 = "Known raised BP/HTN" if v1 == "17"

replace v1 = "Take medication for BP/HTN" if v1 == "18"

save File_RD_IVR_IPW, replace

restore

********

preserve

recode file1 (1 = 0) (0 = 1)

keep if sex_n == 1

svyset [pweight= age_sex_w]

gen file2 = file1

gen file3 = file1

gen file4 = file1

gen file5 = file1

gen file6 = file1

gen file7 = file1

gen file8 = file1

gen file9 = file1

gen file10 = file1

gen file11 = file1

gen file12 = file1

gen file13 = file1

gen file14 = file1

gen file15 = file1

gen file16 = file1

gen file17 = file1

gen file18 = file1

svy: glm current_tobacco_any file1, fam(bin) link(identity)

estimates store I1

svy: glm current_smoke_n file2, fam(bin) link(identity)

estimates store I2

svy: glm current_smokeless_n file3, fam(bin) link(identity)

estimates store I3

svy: glm daily_tobacco_any file4, fam(bin) link(identity)

estimates store I4

svy: glm daily_smoke_n file5, fam(bin) link(identity)

estimates store I5

svy: glm daily_smokeless_n file6, fam(bin) link(identity)

estimates store I6

svy: glm smoke_only file7, fam(bin) link(identity)

estimates store I7

svy: glm smokeless_only file8, fam(bin) link(identity)

estimates store I8

svy: glm both_smoke_smokeless file9, fam(bin) link(identity)

estimates store I9

svy: glm alcohol_current file10, fam(bin) link(identity)

estimates store I10

svy: glm no_fruit_veg file11, fam(bin) link(identity)

estimates store I11

svy: glm no_fruit file12, fam(bin) link(identity)

estimates store I12

svy: glm no_veg file13, fam(bin) link(identity)

estimates store I13

svy: glm atelessthan5 file14, fam(bin) link(identity)

estimates store I14

svy: glm salt_eat_n file15, fam(bin) link(identity)

estimates store I15

svy: glm salt_pack_n file16, fam(bin) link(identity)

estimates store I16

svy: glm htn_known file17, fam(bin) link(identity)

estimates store I17

svy: glm htn_med file18, fam(bin) link(identity)

estimates store I18

estout I1 I2 I3 I4 I5 I6 I7 I8 I9 I10 I11 I12 I13 I14 I15 I16 I17 I18 using Table_RD.xls, ///

cells ("b(star fmt(1)) ci(par fmt(1) label(95% CI)) p (fmt(4)) ") replace transform(@*100)

import delimited "Table_RD.xls", clear

replace v2 = v5 if v2 == ""

replace v2 = v8 if v2 == ""

replace v2 = v11 if v2 == ""

replace v2 = v14 if v2 == ""

replace v2 = v17 if v2 == ""

replace v2 = v20 if v2 == ""

replace v2 = v23 if v2 == ""

replace v2 = v26 if v2 == ""

replace v2 = v29 if v2 == ""

replace v2 = v32 if v2 == ""

replace v2 = v35 if v2 == ""

replace v2 = v38 if v2 == ""

replace v2 = v41 if v2 == ""

replace v2 = v44 if v2 == ""

replace v2 = v47 if v2 == ""

replace v2 = v50 if v2 == ""

replace v2 = v53 if v2 == ""

replace v3 = v6 if v3 == ""

replace v3 = v9 if v3 == ""

replace v3 = v12 if v3 == ""

replace v3 = v15 if v3 == ""

replace v3 = v18 if v3 == ""

replace v3 = v21 if v3 == ""

replace v3 = v24 if v3 == ""

replace v3 = v27 if v3 == ""

replace v3 = v30 if v3 == ""

replace v3 = v33 if v3 == ""

replace v3 = v36 if v3 == ""

replace v3 = v39 if v3 == ""

replace v3 = v42 if v3 == ""

replace v3 = v45 if v3 == ""

replace v3 = v48 if v3 == ""

replace v3 = v51 if v3 == ""

replace v3 = v54 if v3 == ""

replace v4 = v7 if v4 == ""

replace v4 = v10 if v4 == ""

replace v4 = v13 if v4 == ""

replace v4 = v16 if v4 == ""

replace v4 = v19 if v4 == ""

replace v4 = v22 if v4 == ""

replace v4 = v25 if v4 == ""

replace v4 = v28 if v4 == ""

replace v4 = v31 if v4 == ""

replace v4 = v34 if v4 == ""

replace v4 = v37 if v4 == ""

replace v4 = v40 if v4 == ""

replace v4 = v43 if v4 == ""

replace v4 = v46 if v4 == ""

replace v4 = v49 if v4 == ""

replace v4 = v52 if v4 == ""

replace v4 = v55 if v4 == ""

keep v1 v2 v3 v4

drop if v1 == ""

drop if v1 == "main"

drop if v1 == "_cons"

destring v1, replace ignore(file)

rename v2 Diff_IVR_Age_Sex

rename v3 Diff_IVR_Age_Sex_CI

rename v4 Diff_IVR_Age_Sex_P

gen pop_group = 1

gen number = v1

tostring v1, replace

replace v1 = "Any current tobacco user" if v1 == "1"

replace v1 = "Current smoker" if v1 == "2"

replace v1 = "Current smokeless tobacco user" if v1 == "3"

replace v1 = "Any daily tobacco user" if v1 == "4"

replace v1 = "Daily smoker" if v1 == "5"

replace v1 = "Daily smokeless tobacco user" if v1 == "6"

replace v1 = "Only smoker" if v1 == "7"

replace v1 = "Only smokeless tobacco user" if v1 == "8"

replace v1 = "Both smoker and smokeless user" if v1 == "9"

replace v1 = "Alcohol past month" if v1 == "10"

replace v1 = "No fruit or vegetables" if v1 == "11"

replace v1 = "No fruit" if v1 == "12"

replace v1 = "No veg" if v1 == "13"

replace v1 = "<5 servings of fruits-veg in a day " if v1 == "14"

replace v1 = "Add salt to food while eating" if v1 == "15"

replace v1 = "Processed food high in salt" if v1 == "16"

replace v1 = "Known raised BP/HTN" if v1 == "17"

replace v1 = "Take medication for BP/HTN" if v1 == "18"

save File_RD_IVR_Age_Sex, replace

restore

********

preserve

use Table3_Unweighted.dta, replace

merge 1:1 v1 using Table3_Weighted_IPW, nogen

merge 1:1 v1 using Table3_Weighted_Age_Sex_W.dta, nogen

merge 1:1 v1 using Table3_n.dta, nogen

sort id

drop id

label var v1 "Indicator"

drop STEPS_IPW

gen indicator = _n

gen pop_group = 8

save Table3_08_Prevalence_GMAK_20Jul2022.dta, replace

merge 1:1 v1 using File_RD_IVR_IPW.dta, nogen

merge 1:1 v1 using File_RD_IVR_Age_Sex.dta, nogen

sort number

order v1 IVR_n IVR_IPW IVR_IPW_CI STEPS_n STEPS_Age_Sex STEPS_Age_Sex_CI Diff_IVR_IPW

export excel v1 IVR_n IVR_IPW IVR_IPW_CI STEPS_n STEPS_Age_Sex STEPS_Age_Sex_CI ///

Diff_IVR_IPW using SumTab_IVR_IPWvsSTEPS.xls, replace

order v1 IVR_n IVR_Age_Sex IVR_Age_Sex_CI STEPS_n STEPS_Age_Sex STEPS_Age_Sex_CI ///

Diff_IVR_Age_Sex

export excel v1 IVR_n IVR_Age_Sex IVR_Age_Sex_CI STEPS_n STEPS_Age_Sex STEPS_Age_Sex_CI ///

Diff_IVR_Age_Sex using SumTab_IVR_AgeSexvsSTEPS.xls, replace

restore

********************************************************************************

preserve

keep if sex_n == 2

keep if age_cat == 0

tabout current_tobacco_any current_smoke_n current_smokeless_n ///

daily_tobacco_any daily_smoke_n daily_smokeless_n ///

smoke_only smokeless_only both_smoke_smokeless ///

alcohol_current no_fruit_veg no_fruit no_veg ///

atelessthan5 salt_eat_n salt_pack_n ///

htn_known htn_med file1 ///

using Table3_n.xls, ///

c(freq col) f(1c 1p) replace

import delimited using Table3_n.xls, clear

drop if v1 == "No"

drop if v1 == "Yes"

replace v1 = v1[_n - 1] if v1 == "Total"

drop v3 v5 v6 v7

drop if v1 == ""

drop if v2 == ""

destring v2 v4, ignore(",") replace

rename v2 IVR_n

rename v4 STEPS_n

gen id = _n

save Table3_n.dta, replace

restore

preserve

keep if sex_n == 2

keep if age_cat == 0

gen wt = 1

svyset [pweight= wt]

tabout current_tobacco_any current_smoke_n current_smokeless_n ///

daily_tobacco_any daily_smoke_n daily_smokeless_n ///

smoke_only smokeless_only both_smoke_smokeless ///

alcohol_current no_fruit_veg no_fruit no_veg ///

atelessthan5 salt_eat_n salt_pack_n ///

htn_known htn_med file1 ///

using Table3_Unweight.xls, ///

c(col ci) f(1 1) percent svy replace

import delimited using Table3_Unweight.xls, clear

drop v6 v7

drop if v1 == "No" | v1 == "Total"

replace v1 = v1[_n - 1] if v1 == "Yes"

drop if v2 == ""

drop if v1 == ""

rename v2 IVR_Unweight

rename v4 STEPS_Unweight

rename v3 IVR_Unweight_CI

rename v5 STEPS_Unweight_CI

gen id = _n

save Table3_Unweighted.dta, replace

restore

********

preserve

keep if sex_n == 2

keep if age_cat == 0

svyset [pweight= weight_ipw]

tabout current_tobacco_any current_smoke_n current_smokeless_n ///

daily_tobacco_any daily_smoke_n daily_smokeless_n ///

smoke_only smokeless_only both_smoke_smokeless ///

alcohol_current no_fruit_veg no_fruit no_veg ///

atelessthan5 salt_eat_n salt_pack_n ///

htn_known htn_med file1 ///

using Table3_Weight_IPW.xls, ///

c(col ci) f(1 1) percent svy replace

import delimited using Table3_Weight_IPW.xls, clear

drop v6 v7

drop if v1 == "No" | v1 == "Total"

replace v1 = v1[_n - 1] if v1 == "Yes"

drop if v2 == ""

drop if v1 == ""

rename v2 IVR_IPW

rename v4 STEPS_IPW

rename v3 IVR_IPW_CI

rename v5 STEPS_IPW_CI

gen id = _n

save Table3_Weighted_IPW.dta, replace

restore

********

preserve

keep if sex_n == 2

keep if age_cat == 0

svyset [pweight= age_sex_w]

tabout current_tobacco_any current_smoke_n current_smokeless_n ///

daily_tobacco_any daily_smoke_n daily_smokeless_n ///

smoke_only smokeless_only both_smoke_smokeless ///

alcohol_current no_fruit_veg no_fruit no_veg ///

atelessthan5 salt_eat_n salt_pack_n ///

htn_known htn_med file1 ///

using Table3_Weight_Age_Sex_W.xls, ///

c(col ci) f(1 1) percent svy replace

import delimited using Table3_Weight_Age_Sex_W.xls, clear

drop v6 v7

drop if v1 == "No" | v1 == "Total"

replace v1 = v1[_n - 1] if v1 == "Yes"

drop if v2 == ""

drop if v1 == ""

rename v2 IVR_Age_Sex

rename v4 STEPS_Age_Sex

rename v3 IVR_Age_Sex_CI

rename v5 STEPS_Age_Sex_CI

gen id = _n

save Table3_Weighted_Age_Sex_W.dta, replace

restore

*********

*****

preserve

recode file1 (1 = 0) (0 = 1)

keep if sex_n == 2

keep if age_cat == 0

svyset [pweight= weight_ipw]

gen file2 = file1

gen file3 = file1

gen file4 = file1

gen file5 = file1

gen file6 = file1

gen file7 = file1

gen file8 = file1

gen file9 = file1

gen file10 = file1

gen file11 = file1

gen file12 = file1

gen file13 = file1

gen file14 = file1

gen file15 = file1

gen file16 = file1

gen file17 = file1

gen file18 = file1

svy: glm current_tobacco_any file1, fam(bin) link(identity)

estimates store I1

svy: glm current_smoke_n file2, fam(bin) link(identity)

estimates store I2

svy: glm current_smokeless_n file3, fam(bin) link(identity)

estimates store I3

svy: glm daily_tobacco_any file4, fam(bin) link(identity)

estimates store I4

svy: glm daily_smoke_n file5, fam(bin) link(identity)

estimates store I5

svy: glm daily_smokeless_n file6, fam(bin) link(identity)

estimates store I6

svy: glm smoke_only file7, fam(bin) link(identity)

estimates store I7

svy: glm smokeless_only file8, fam(bin) link(identity)

estimates store I8

svy: glm both_smoke_smokeless file9, fam(bin) link(identity)

estimates store I9

svy: glm alcohol_current file10, fam(bin) link(identity)

estimates store I10

svy: glm no_fruit_veg file11, fam(bin) link(identity)

estimates store I11

svy: glm no_fruit file12, fam(bin) link(identity)

estimates store I12

svy: glm no_veg file13, fam(bin) link(identity)

estimates store I13

svy: glm atelessthan5 file14, fam(bin) link(identity)

estimates store I14

svy: glm salt_eat_n file15, fam(bin) link(identity)

estimates store I15

svy: glm salt_pack_n file16, fam(bin) link(identity)

estimates store I16

svy: glm htn_known file17, fam(bin) link(identity)

estimates store I17

svy: glm htn_med file18, fam(bin) link(identity)

estimates store I18

estout I1 I2 I3 I4 I5 I6 I7 I8 I9 I10 I11 I12 I13 I14 I15 I16 I17 I18 using Table_RD.xls, ///

cells ("b(star fmt(1)) ci(par fmt(1) label(95% CI)) p (fmt(4)) ") replace transform(@*100)

import delimited "Table_RD.xls", clear

replace v2 = v5 if v2 == ""

replace v2 = v8 if v2 == ""

replace v2 = v11 if v2 == ""

replace v2 = v14 if v2 == ""

replace v2 = v17 if v2 == ""

replace v2 = v20 if v2 == ""

replace v2 = v23 if v2 == ""

replace v2 = v26 if v2 == ""

replace v2 = v29 if v2 == ""

replace v2 = v32 if v2 == ""

replace v2 = v35 if v2 == ""

replace v2 = v38 if v2 == ""

replace v2 = v41 if v2 == ""

replace v2 = v44 if v2 == ""

replace v2 = v47 if v2 == ""

replace v2 = v50 if v2 == ""

replace v2 = v53 if v2 == ""

replace v3 = v6 if v3 == ""

replace v3 = v9 if v3 == ""

replace v3 = v12 if v3 == ""

replace v3 = v15 if v3 == ""

replace v3 = v18 if v3 == ""

replace v3 = v21 if v3 == ""

replace v3 = v24 if v3 == ""

replace v3 = v27 if v3 == ""

replace v3 = v30 if v3 == ""

replace v3 = v33 if v3 == ""

replace v3 = v36 if v3 == ""

replace v3 = v39 if v3 == ""

replace v3 = v42 if v3 == ""

replace v3 = v45 if v3 == ""

replace v3 = v48 if v3 == ""

replace v3 = v51 if v3 == ""

replace v3 = v54 if v3 == ""

replace v4 = v7 if v4 == ""

replace v4 = v10 if v4 == ""

replace v4 = v13 if v4 == ""

replace v4 = v16 if v4 == ""

replace v4 = v19 if v4 == ""

replace v4 = v22 if v4 == ""

replace v4 = v25 if v4 == ""

replace v4 = v28 if v4 == ""

replace v4 = v31 if v4 == ""

replace v4 = v34 if v4 == ""

replace v4 = v37 if v4 == ""

replace v4 = v40 if v4 == ""

replace v4 = v43 if v4 == ""

replace v4 = v46 if v4 == ""

replace v4 = v49 if v4 == ""

replace v4 = v52 if v4 == ""

replace v4 = v55 if v4 == ""

keep v1 v2 v3 v4

drop if v1 == ""

drop if v1 == "main"

drop if v1 == "_cons"

destring v1, replace ignore(file)

rename v2 Diff_IVR_IPW

rename v3 Diff_IVR_IPW_CI

rename v4 Diff_IVR_IPW_P

gen pop_group = 1

tostring v1, replace

replace v1 = "Any current tobacco user" if v1 == "1"

replace v1 = "Current smoker" if v1 == "2"

replace v1 = "Current smokeless tobacco user" if v1 == "3"

replace v1 = "Any daily tobacco user" if v1 == "4"

replace v1 = "Daily smoker" if v1 == "5"

replace v1 = "Daily smokeless tobacco user" if v1 == "6"

replace v1 = "Only smoker" if v1 == "7"

replace v1 = "Only smokeless tobacco user" if v1 == "8"

replace v1 = "Both smoker and smokeless user" if v1 == "9"

replace v1 = "Alcohol past month" if v1 == "10"

replace v1 = "No fruit or vegetables" if v1 == "11"

replace v1 = "No fruit" if v1 == "12"

replace v1 = "No veg" if v1 == "13"

replace v1 = "<5 servings of fruits-veg in a day " if v1 == "14"

replace v1 = "Add salt to food while eating" if v1 == "15"

replace v1 = "Processed food high in salt" if v1 == "16"

replace v1 = "Known raised BP/HTN" if v1 == "17"

replace v1 = "Take medication for BP/HTN" if v1 == "18"

save File_RD_IVR_IPW, replace

restore

********

preserve

recode file1 (1 = 0) (0 = 1)

keep if sex_n == 2

keep if age_cat == 0

svyset [pweight= age_sex_w]

gen file2 = file1

gen file3 = file1

gen file4 = file1

gen file5 = file1

gen file6 = file1

gen file7 = file1

gen file8 = file1

gen file9 = file1

gen file10 = file1

gen file11 = file1

gen file12 = file1

gen file13 = file1

gen file14 = file1

gen file15 = file1

gen file16 = file1

gen file17 = file1

gen file18 = file1

svy: glm current_tobacco_any file1, fam(bin) link(identity)

estimates store I1

svy: glm current_smoke_n file2, fam(bin) link(identity)

estimates store I2

svy: glm current_smokeless_n file3, fam(bin) link(identity)

estimates store I3

svy: glm daily_tobacco_any file4, fam(bin) link(identity)

estimates store I4

svy: glm daily_smoke_n file5, fam(bin) link(identity)

estimates store I5

svy: glm daily_smokeless_n file6, fam(bin) link(identity)

estimates store I6

svy: glm smoke_only file7, fam(bin) link(identity)

estimates store I7

svy: glm smokeless_only file8, fam(bin) link(identity)

estimates store I8

svy: glm both_smoke_smokeless file9, fam(bin) link(identity)

estimates store I9

svy: glm alcohol_current file10, fam(bin) link(identity)

estimates store I10

svy: glm no_fruit_veg file11, fam(bin) link(identity)

estimates store I11

svy: glm no_fruit file12, fam(bin) link(identity)

estimates store I12

svy: glm no_veg file13, fam(bin) link(identity)

estimates store I13

svy: glm atelessthan5 file14, fam(bin) link(identity)

estimates store I14

svy: glm salt_eat_n file15, fam(bin) link(identity)

estimates store I15

svy: glm salt_pack_n file16, fam(bin) link(identity)

estimates store I16

svy: glm htn_known file17, fam(bin) link(identity)

estimates store I17

svy: glm htn_med file18, fam(bin) link(identity)

estimates store I18

estout I1 I2 I3 I4 I5 I6 I7 I8 I9 I10 I11 I12 I13 I14 I15 I16 I17 I18 using Table_RD.xls, ///

cells ("b(star fmt(1)) ci(par fmt(1) label(95% CI)) p (fmt(4)) ") replace transform(@*100)

import delimited "Table_RD.xls", clear

replace v2 = v5 if v2 == ""

replace v2 = v8 if v2 == ""

replace v2 = v11 if v2 == ""

replace v2 = v14 if v2 == ""

replace v2 = v17 if v2 == ""

replace v2 = v20 if v2 == ""

replace v2 = v23 if v2 == ""

replace v2 = v26 if v2 == ""

replace v2 = v29 if v2 == ""

replace v2 = v32 if v2 == ""

replace v2 = v35 if v2 == ""

replace v2 = v38 if v2 == ""

replace v2 = v41 if v2 == ""

replace v2 = v44 if v2 == ""

replace v2 = v47 if v2 == ""

replace v2 = v50 if v2 == ""

replace v2 = v53 if v2 == ""

replace v3 = v6 if v3 == ""

replace v3 = v9 if v3 == ""

replace v3 = v12 if v3 == ""

replace v3 = v15 if v3 == ""

replace v3 = v18 if v3 == ""

replace v3 = v21 if v3 == ""

replace v3 = v24 if v3 == ""

replace v3 = v27 if v3 == ""

replace v3 = v30 if v3 == ""

replace v3 = v33 if v3 == ""

replace v3 = v36 if v3 == ""

replace v3 = v39 if v3 == ""

replace v3 = v42 if v3 == ""

replace v3 = v45 if v3 == ""

replace v3 = v48 if v3 == ""

replace v3 = v51 if v3 == ""

replace v3 = v54 if v3 == ""

replace v4 = v7 if v4 == ""

replace v4 = v10 if v4 == ""

replace v4 = v13 if v4 == ""

replace v4 = v16 if v4 == ""

replace v4 = v19 if v4 == ""

replace v4 = v22 if v4 == ""

replace v4 = v25 if v4 == ""

replace v4 = v28 if v4 == ""

replace v4 = v31 if v4 == ""

replace v4 = v34 if v4 == ""

replace v4 = v37 if v4 == ""

replace v4 = v40 if v4 == ""

replace v4 = v43 if v4 == ""

replace v4 = v46 if v4 == ""

replace v4 = v49 if v4 == ""

replace v4 = v52 if v4 == ""

replace v4 = v55 if v4 == ""

keep v1 v2 v3 v4

drop if v1 == ""

drop if v1 == "main"

drop if v1 == "_cons"

destring v1, replace ignore(file)

rename v2 Diff_IVR_Age_Sex

rename v3 Diff_IVR_Age_Sex_CI

rename v4 Diff_IVR_Age_Sex_P

gen pop_group = 1

gen number = v1

tostring v1, replace

replace v1 = "Any current tobacco user" if v1 == "1"

replace v1 = "Current smoker" if v1 == "2"

replace v1 = "Current smokeless tobacco user" if v1 == "3"

replace v1 = "Any daily tobacco user" if v1 == "4"

replace v1 = "Daily smoker" if v1 == "5"

replace v1 = "Daily smokeless tobacco user" if v1 == "6"

replace v1 = "Only smoker" if v1 == "7"

replace v1 = "Only smokeless tobacco user" if v1 == "8"

replace v1 = "Both smoker and smokeless user" if v1 == "9"

replace v1 = "Alcohol past month" if v1 == "10"

replace v1 = "No fruit or vegetables" if v1 == "11"

replace v1 = "No fruit" if v1 == "12"

replace v1 = "No veg" if v1 == "13"

replace v1 = "<5 servings of fruits-veg in a day " if v1 == "14"

replace v1 = "Add salt to food while eating" if v1 == "15"

replace v1 = "Processed food high in salt" if v1 == "16"

replace v1 = "Known raised BP/HTN" if v1 == "17"

replace v1 = "Take medication for BP/HTN" if v1 == "18"

save File_RD_IVR_Age_Sex, replace

restore

*********

preserve

use Table3_Unweighted.dta, replace

merge 1:1 v1 using Table3_Weighted_IPW, nogen

merge 1:1 v1 using Table3_Weighted_Age_Sex_W.dta, nogen

merge 1:1 v1 using Table3_n.dta, nogen

sort id

drop id

label var v1 "Indicator"

drop STEPS_IPW

gen indicator = _n

gen pop_group = 9

save Table3_09_Prevalence_GMAK_20Jul2022.dta, replace

merge 1:1 v1 using File_RD_IVR_IPW.dta, nogen

merge 1:1 v1 using File_RD_IVR_Age_Sex.dta, nogen

sort number

order v1 IVR_n IVR_IPW IVR_IPW_CI STEPS_n STEPS_Age_Sex STEPS_Age_Sex_CI Diff_IVR_IPW

export excel v1 IVR_n IVR_IPW IVR_IPW_CI STEPS_n STEPS_Age_Sex STEPS_Age_Sex_CI ///

Diff_IVR_IPW using SumTab_IVR_IPWvsSTEPS.xls, replace

order v1 IVR_n IVR_Age_Sex IVR_Age_Sex_CI STEPS_n STEPS_Age_Sex STEPS_Age_Sex_CI ///

Diff_IVR_Age_Sex

export excel v1 IVR_n IVR_Age_Sex IVR_Age_Sex_CI STEPS_n STEPS_Age_Sex STEPS_Age_Sex_CI ///

Diff_IVR_Age_Sex using SumTab_IVR_AgeSexvsSTEPS.xls, replace

restore

********************************************************************************

preserve

keep if sex_n == 2

keep if age_cat == 1

tabout current_tobacco_any current_smoke_n current_smokeless_n ///

daily_tobacco_any daily_smoke_n daily_smokeless_n ///

smoke_only smokeless_only both_smoke_smokeless ///

alcohol_current no_fruit_veg no_fruit no_veg ///

atelessthan5 salt_eat_n salt_pack_n ///

htn_known htn_med file1 ///

using Table3_n.xls, ///

c(freq col) f(1c 1p) replace

import delimited using Table3_n.xls, clear

drop if v1 == "No"

drop if v1 == "Yes"

replace v1 = v1[_n - 1] if v1 == "Total"

drop v3 v5 v6 v7

drop if v1 == ""

drop if v2 == ""

destring v2 v4, ignore(",") replace

rename v2 IVR_n

rename v4 STEPS_n

gen id = _n

save Table3_n.dta, replace

restore

preserve

keep if sex_n == 2

keep if age_cat == 1

gen wt = 1

svyset [pweight= wt]

tabout current_tobacco_any current_smoke_n current_smokeless_n ///

daily_tobacco_any daily_smoke_n daily_smokeless_n ///

smoke_only smokeless_only both_smoke_smokeless ///

alcohol_current no_fruit_veg no_fruit no_veg ///

atelessthan5 salt_eat_n salt_pack_n ///

htn_known htn_med file1 ///

using Table3_Unweight.xls, ///

c(col ci) f(1 1) percent svy replace

import delimited using Table3_Unweight.xls, clear

drop v6 v7

drop if v1 == "No" | v1 == "Total"

replace v1 = v1[_n - 1] if v1 == "Yes"

drop if v2 == ""

drop if v1 == ""

rename v2 IVR_Unweight

rename v4 STEPS_Unweight

rename v3 IVR_Unweight_CI

rename v5 STEPS_Unweight_CI

gen id = _n

save Table3_Unweighted.dta, replace

restore

********

preserve

keep if sex_n == 2

keep if age_cat == 1

svyset [pweight= weight_ipw]

tabout current_tobacco_any current_smoke_n current_smokeless_n ///

daily_tobacco_any daily_smoke_n daily_smokeless_n ///

smoke_only smokeless_only both_smoke_smokeless ///

alcohol_current no_fruit_veg no_fruit no_veg ///

atelessthan5 salt_eat_n salt_pack_n ///

htn_known htn_med file1 ///

using Table3_Weight_IPW.xls, ///

c(col ci) f(1 1) percent svy replace

import delimited using Table3_Weight_IPW.xls, clear

drop v6 v7

drop if v1 == "No" | v1 == "Total"

replace v1 = v1[_n - 1] if v1 == "Yes"

drop if v2 == ""

drop if v1 == ""

rename v2 IVR_IPW

rename v4 STEPS_IPW

rename v3 IVR_IPW_CI

rename v5 STEPS_IPW_CI

gen id = _n

save Table3_Weighted_IPW.dta, replace

restore

********

preserve

keep if sex_n == 2

keep if age_cat == 1

svyset [pweight= age_sex_w]

tabout current_tobacco_any current_smoke_n current_smokeless_n ///

daily_tobacco_any daily_smoke_n daily_smokeless_n ///

smoke_only smokeless_only both_smoke_smokeless ///

alcohol_current no_fruit_veg no_fruit no_veg ///

atelessthan5 salt_eat_n salt_pack_n ///

htn_known htn_med file1 ///

using Table3_Weight_Age_Sex_W.xls, ///

c(col ci) f(1 1) percent svy replace

import delimited using Table3_Weight_Age_Sex_W.xls, clear

drop v6 v7

drop if v1 == "No" | v1 == "Total"

replace v1 = v1[_n - 1] if v1 == "Yes"

drop if v2 == ""

drop if v1 == ""

rename v2 IVR_Age_Sex

rename v4 STEPS_Age_Sex

rename v3 IVR_Age_Sex_CI

rename v5 STEPS_Age_Sex_CI

gen id = _n

save Table3_Weighted_Age_Sex_W.dta, replace

restore

*******

*****

preserve

recode file1 (1 = 0) (0 = 1)

keep if sex_n == 2

keep if age_cat == 1

svyset [pweight= weight_ipw]

gen file2 = file1

gen file3 = file1

gen file4 = file1

gen file5 = file1

gen file6 = file1

gen file7 = file1

gen file8 = file1

gen file9 = file1

gen file10 = file1

gen file11 = file1

gen file12 = file1

gen file13 = file1

gen file14 = file1

gen file15 = file1

gen file16 = file1

gen file17 = file1

gen file18 = file1

svy: glm current_tobacco_any file1, fam(bin) link(identity)

estimates store I1

svy: glm current_smoke_n file2, fam(bin) link(identity)

estimates store I2

svy: glm current_smokeless_n file3, fam(bin) link(identity)

estimates store I3

svy: glm daily_tobacco_any file4, fam(bin) link(identity)

estimates store I4

svy: glm daily_smoke_n file5, fam(bin) link(identity)

estimates store I5

svy: glm daily_smokeless_n file6, fam(bin) link(identity)

estimates store I6

svy: glm smoke_only file7, fam(bin) link(identity)

estimates store I7

svy: glm smokeless_only file8, fam(bin) link(identity)

estimates store I8

svy: glm both_smoke_smokeless file9, fam(bin) link(identity)

estimates store I9

svy: glm alcohol_current file10, fam(bin) link(identity)

estimates store I10

svy: glm no_fruit_veg file11, fam(bin) link(identity)

estimates store I11

svy: glm no_fruit file12, fam(bin) link(identity)

estimates store I12

svy: glm no_veg file13, fam(bin) link(identity)

estimates store I13

svy: glm atelessthan5 file14, fam(bin) link(identity)

estimates store I14

svy: glm salt_eat_n file15, fam(bin) link(identity)

estimates store I15

svy: glm salt_pack_n file16, fam(bin) link(identity)

estimates store I16

svy: glm htn_known file17, fam(bin) link(identity)

estimates store I17

svy: glm htn_med file18, fam(bin) link(identity)

estimates store I18

estout I1 I2 I3 I4 I5 I6 I7 I8 I9 I10 I11 I12 I13 I14 I15 I16 I17 I18 using Table_RD.xls, ///

cells ("b(star fmt(1)) ci(par fmt(1) label(95% CI)) p (fmt(4)) ") replace transform(@*100)

import delimited "Table_RD.xls", clear

replace v2 = v5 if v2 == ""

replace v2 = v8 if v2 == ""

replace v2 = v11 if v2 == ""

replace v2 = v14 if v2 == ""

replace v2 = v17 if v2 == ""

replace v2 = v20 if v2 == ""

replace v2 = v23 if v2 == ""

replace v2 = v26 if v2 == ""

replace v2 = v29 if v2 == ""

replace v2 = v32 if v2 == ""

replace v2 = v35 if v2 == ""

replace v2 = v38 if v2 == ""

replace v2 = v41 if v2 == ""

replace v2 = v44 if v2 == ""

replace v2 = v47 if v2 == ""

replace v2 = v50 if v2 == ""

replace v2 = v53 if v2 == ""

replace v3 = v6 if v3 == ""

replace v3 = v9 if v3 == ""

replace v3 = v12 if v3 == ""

replace v3 = v15 if v3 == ""

replace v3 = v18 if v3 == ""

replace v3 = v21 if v3 == ""

replace v3 = v24 if v3 == ""

replace v3 = v27 if v3 == ""

replace v3 = v30 if v3 == ""

replace v3 = v33 if v3 == ""

replace v3 = v36 if v3 == ""

replace v3 = v39 if v3 == ""

replace v3 = v42 if v3 == ""

replace v3 = v45 if v3 == ""

replace v3 = v48 if v3 == ""

replace v3 = v51 if v3 == ""

replace v3 = v54 if v3 == ""

replace v4 = v7 if v4 == ""

replace v4 = v10 if v4 == ""

replace v4 = v13 if v4 == ""

replace v4 = v16 if v4 == ""

replace v4 = v19 if v4 == ""

replace v4 = v22 if v4 == ""

replace v4 = v25 if v4 == ""

replace v4 = v28 if v4 == ""

replace v4 = v31 if v4 == ""

replace v4 = v34 if v4 == ""

replace v4 = v37 if v4 == ""

replace v4 = v40 if v4 == ""

replace v4 = v43 if v4 == ""

replace v4 = v46 if v4 == ""

replace v4 = v49 if v4 == ""

replace v4 = v52 if v4 == ""

replace v4 = v55 if v4 == ""

keep v1 v2 v3 v4

drop if v1 == ""

drop if v1 == "main"

drop if v1 == "_cons"

destring v1, replace ignore(file)

rename v2 Diff_IVR_IPW

rename v3 Diff_IVR_IPW_CI

rename v4 Diff_IVR_IPW_P

gen pop_group = 1

tostring v1, replace

replace v1 = "Any current tobacco user" if v1 == "1"

replace v1 = "Current smoker" if v1 == "2"

replace v1 = "Current smokeless tobacco user" if v1 == "3"

replace v1 = "Any daily tobacco user" if v1 == "4"

replace v1 = "Daily smoker" if v1 == "5"

replace v1 = "Daily smokeless tobacco user" if v1 == "6"

replace v1 = "Only smoker" if v1 == "7"

replace v1 = "Only smokeless tobacco user" if v1 == "8"

replace v1 = "Both smoker and smokeless user" if v1 == "9"

replace v1 = "Alcohol past month" if v1 == "10"

replace v1 = "No fruit or vegetables" if v1 == "11"

replace v1 = "No fruit" if v1 == "12"

replace v1 = "No veg" if v1 == "13"

replace v1 = "<5 servings of fruits-veg in a day " if v1 == "14"

replace v1 = "Add salt to food while eating" if v1 == "15"

replace v1 = "Processed food high in salt" if v1 == "16"

replace v1 = "Known raised BP/HTN" if v1 == "17"

replace v1 = "Take medication for BP/HTN" if v1 == "18"

save File_RD_IVR_IPW, replace

restore

********

preserve

recode file1 (1 = 0) (0 = 1)

keep if sex_n == 2

keep if age_cat == 1

svyset [pweight= age_sex_w]

gen file2 = file1

gen file3 = file1

gen file4 = file1

gen file5 = file1

gen file6 = file1

gen file7 = file1

gen file8 = file1

gen file9 = file1

gen file10 = file1

gen file11 = file1

gen file12 = file1

gen file13 = file1

gen file14 = file1

gen file15 = file1

gen file16 = file1

gen file17 = file1

gen file18 = file1

svy: glm current_tobacco_any file1, fam(bin) link(identity)

estimates store I1

svy: glm current_smoke_n file2, fam(bin) link(identity)

estimates store I2

svy: glm current_smokeless_n file3, fam(bin) link(identity)

estimates store I3

svy: glm daily_tobacco_any file4, fam(bin) link(identity)

estimates store I4

svy: glm daily_smoke_n file5, fam(bin) link(identity)

estimates store I5

svy: glm daily_smokeless_n file6, fam(bin) link(identity)

estimates store I6

svy: glm smoke_only file7, fam(bin) link(identity)

estimates store I7

svy: glm smokeless_only file8, fam(bin) link(identity)

estimates store I8

svy: glm both_smoke_smokeless file9, fam(bin) link(identity)

estimates store I9

svy: glm alcohol_current file10, fam(bin) link(identity)

estimates store I10

svy: glm no_fruit_veg file11, fam(bin) link(identity)

estimates store I11

svy: glm no_fruit file12, fam(bin) link(identity)

estimates store I12

svy: glm no_veg file13, fam(bin) link(identity)

estimates store I13

svy: glm atelessthan5 file14, fam(bin) link(identity)

estimates store I14

svy: glm salt_eat_n file15, fam(bin) link(identity)

estimates store I15

svy: glm salt_pack_n file16, fam(bin) link(identity)

estimates store I16

svy: glm htn_known file17, fam(bin) link(identity)

estimates store I17

svy: glm htn_med file18, fam(bin) link(identity)

estimates store I18

estout I1 I2 I3 I4 I5 I6 I7 I8 I9 I10 I11 I12 I13 I14 I15 I16 I17 I18 using Table_RD.xls, ///

cells ("b(star fmt(1)) ci(par fmt(1) label(95% CI)) p (fmt(4)) ") replace transform(@*100)

import delimited "Table_RD.xls", clear

replace v2 = v5 if v2 == ""

replace v2 = v8 if v2 == ""

replace v2 = v11 if v2 == ""

replace v2 = v14 if v2 == ""

replace v2 = v17 if v2 == ""

replace v2 = v20 if v2 == ""

replace v2 = v23 if v2 == ""

replace v2 = v26 if v2 == ""

replace v2 = v29 if v2 == ""

replace v2 = v32 if v2 == ""

replace v2 = v35 if v2 == ""

replace v2 = v38 if v2 == ""

replace v2 = v41 if v2 == ""

replace v2 = v44 if v2 == ""

replace v2 = v47 if v2 == ""

replace v2 = v50 if v2 == ""

replace v2 = v53 if v2 == ""

replace v3 = v6 if v3 == ""

replace v3 = v9 if v3 == ""

replace v3 = v12 if v3 == ""

replace v3 = v15 if v3 == ""

replace v3 = v18 if v3 == ""

replace v3 = v21 if v3 == ""

replace v3 = v24 if v3 == ""

replace v3 = v27 if v3 == ""

replace v3 = v30 if v3 == ""

replace v3 = v33 if v3 == ""

replace v3 = v36 if v3 == ""

replace v3 = v39 if v3 == ""

replace v3 = v42 if v3 == ""

replace v3 = v45 if v3 == ""

replace v3 = v48 if v3 == ""

replace v3 = v51 if v3 == ""

replace v3 = v54 if v3 == ""

replace v4 = v7 if v4 == ""

replace v4 = v10 if v4 == ""

replace v4 = v13 if v4 == ""

replace v4 = v16 if v4 == ""

replace v4 = v19 if v4 == ""

replace v4 = v22 if v4 == ""

replace v4 = v25 if v4 == ""

replace v4 = v28 if v4 == ""

replace v4 = v31 if v4 == ""

replace v4 = v34 if v4 == ""

replace v4 = v37 if v4 == ""

replace v4 = v40 if v4 == ""

replace v4 = v43 if v4 == ""

replace v4 = v46 if v4 == ""

replace v4 = v49 if v4 == ""

replace v4 = v52 if v4 == ""

replace v4 = v55 if v4 == ""

keep v1 v2 v3 v4

drop if v1 == ""

drop if v1 == "main"

drop if v1 == "_cons"

destring v1, replace ignore(file)

rename v2 Diff_IVR_Age_Sex

rename v3 Diff_IVR_Age_Sex_CI

rename v4 Diff_IVR_Age_Sex_P

gen pop_group = 1

gen number = v1

tostring v1, replace

replace v1 = "Any current tobacco user" if v1 == "1"

replace v1 = "Current smoker" if v1 == "2"

replace v1 = "Current smokeless tobacco user" if v1 == "3"

replace v1 = "Any daily tobacco user" if v1 == "4"

replace v1 = "Daily smoker" if v1 == "5"

replace v1 = "Daily smokeless tobacco user" if v1 == "6"

replace v1 = "Only smoker" if v1 == "7"

replace v1 = "Only smokeless tobacco user" if v1 == "8"

replace v1 = "Both smoker and smokeless user" if v1 == "9"

replace v1 = "Alcohol past month" if v1 == "10"

replace v1 = "No fruit or vegetables" if v1 == "11"

replace v1 = "No fruit" if v1 == "12"

replace v1 = "No veg" if v1 == "13"

replace v1 = "<5 servings of fruits-veg in a day " if v1 == "14"

replace v1 = "Add salt to food while eating" if v1 == "15"

replace v1 = "Processed food high in salt" if v1 == "16"

replace v1 = "Known raised BP/HTN" if v1 == "17"

replace v1 = "Take medication for BP/HTN" if v1 == "18"

save File_RD_IVR_Age_Sex, replace

restore

*******

preserve

use Table3_Unweighted.dta, replace

merge 1:1 v1 using Table3_Weighted_IPW, nogen

merge 1:1 v1 using Table3_Weighted_Age_Sex_W.dta, nogen

merge 1:1 v1 using Table3_n.dta, nogen

sort id

drop id

label var v1 "Indicator"

drop STEPS_IPW

gen indicator = _n

gen pop_group = 10

save Table3_10_Prevalence_GMAK_20Jul2022.dta, replace

merge 1:1 v1 using File_RD_IVR_IPW.dta, nogen

merge 1:1 v1 using File_RD_IVR_Age_Sex.dta, nogen

sort number

order v1 IVR_n IVR_IPW IVR_IPW_CI STEPS_n STEPS_Age_Sex STEPS_Age_Sex_CI Diff_IVR_IPW

export excel v1 IVR_n IVR_IPW IVR_IPW_CI STEPS_n STEPS_Age_Sex STEPS_Age_Sex_CI ///

Diff_IVR_IPW using SumTab_IVR_IPWvsSTEPS.xls, replace

order v1 IVR_n IVR_Age_Sex IVR_Age_Sex_CI STEPS_n STEPS_Age_Sex STEPS_Age_Sex_CI ///

Diff_IVR_Age_Sex

export excel v1 IVR_n IVR_Age_Sex IVR_Age_Sex_CI STEPS_n STEPS_Age_Sex STEPS_Age_Sex_CI ///

Diff_IVR_Age_Sex using SumTab_IVR_AgeSexvsSTEPS.xls, replace

restore

********************************************************************************

preserve

keep if sex_n == 2

keep if age_cat == 2

tabout current_tobacco_any current_smoke_n current_smokeless_n ///

daily_tobacco_any daily_smoke_n daily_smokeless_n ///

smoke_only smokeless_only both_smoke_smokeless ///

alcohol_current no_fruit_veg no_fruit no_veg ///

atelessthan5 salt_eat_n salt_pack_n ///

htn_known htn_med file1 ///

using Table3_n.xls, ///

c(freq col) f(1c 1p) replace

import delimited using Table3_n.xls, clear

drop if v1 == "No"

drop if v1 == "Yes"

replace v1 = v1[_n - 1] if v1 == "Total"

drop v3 v5 v6 v7

drop if v1 == ""

drop if v2 == ""

destring v2 v4, ignore(",") replace

rename v2 IVR_n

rename v4 STEPS_n

gen id = _n

save Table3_n.dta, replace

restore

preserve

keep if sex_n == 2

keep if age_cat == 2

gen wt = 1

svyset [pweight= wt]

tabout current_tobacco_any current_smoke_n current_smokeless_n ///

daily_tobacco_any daily_smoke_n daily_smokeless_n ///

smoke_only smokeless_only both_smoke_smokeless ///

alcohol_current no_fruit_veg no_fruit no_veg ///

atelessthan5 salt_eat_n salt_pack_n ///

htn_known htn_med file1 ///

using Table3_Unweight.xls, ///

c(col ci) f(1 1) percent svy replace

import delimited using Table3_Unweight.xls, clear

drop v6 v7

drop if v1 == "No" | v1 == "Total"

replace v1 = v1[_n - 1] if v1 == "Yes"

drop if v2 == ""

drop if v1 == ""

rename v2 IVR_Unweight

rename v4 STEPS_Unweight

rename v3 IVR_Unweight_CI

rename v5 STEPS_Unweight_CI

gen id = _n

save Table3_Unweighted.dta, replace

restore

********

preserve

keep if sex_n == 2

keep if age_cat == 2

svyset [pweight= weight_ipw]

tabout current_tobacco_any current_smoke_n current_smokeless_n ///

daily_tobacco_any daily_smoke_n daily_smokeless_n ///

smoke_only smokeless_only both_smoke_smokeless ///

alcohol_current no_fruit_veg no_fruit no_veg ///

atelessthan5 salt_eat_n salt_pack_n ///

htn_known htn_med file1 ///

using Table3_Weight_IPW.xls, ///

c(col ci) f(1 1) percent svy replace

import delimited using Table3_Weight_IPW.xls, clear

drop v6 v7

drop if v1 == "No" | v1 == "Total"

replace v1 = v1[_n - 1] if v1 == "Yes"

drop if v2 == ""

drop if v1 == ""

rename v2 IVR_IPW

rename v4 STEPS_IPW

rename v3 IVR_IPW_CI

rename v5 STEPS_IPW_CI

gen id = _n

save Table3_Weighted_IPW.dta, replace

restore

********

preserve

keep if sex_n == 2

keep if age_cat == 2

svyset [pweight= age_sex_w]

tabout current_tobacco_any current_smoke_n current_smokeless_n ///

daily_tobacco_any daily_smoke_n daily_smokeless_n ///

smoke_only smokeless_only both_smoke_smokeless ///

alcohol_current no_fruit_veg no_fruit no_veg ///

atelessthan5 salt_eat_n salt_pack_n ///

htn_known htn_med file1 ///

using Table3_Weight_Age_Sex_W.xls, ///

c(col ci) f(1 1) percent svy replace

import delimited using Table3_Weight_Age_Sex_W.xls, clear

drop v6 v7

drop if v1 == "No" | v1 == "Total"

replace v1 = v1[_n - 1] if v1 == "Yes"

drop if v2 == ""

drop if v1 == ""

rename v2 IVR_Age_Sex

rename v4 STEPS_Age_Sex

rename v3 IVR_Age_Sex_CI

rename v5 STEPS_Age_Sex_CI

gen id = _n

save Table3_Weighted_Age_Sex_W.dta, replace

restore

********

****

preserve

recode file1 (1 = 0) (0 = 1)

keep if sex_n == 2

keep if age_cat == 2

svyset [pweight= weight_ipw]

gen file2 = file1

gen file3 = file1

gen file4 = file1

gen file5 = file1

gen file6 = file1

gen file7 = file1

gen file8 = file1

gen file9 = file1

gen file10 = file1

gen file11 = file1

gen file12 = file1

gen file13 = file1

gen file14 = file1

gen file15 = file1

gen file16 = file1

gen file17 = file1

gen file18 = file1

svy: glm current_tobacco_any file1, fam(bin) link(identity)

estimates store I1

svy: glm current_smoke_n file2, fam(bin) link(identity)

estimates store I2

svy: glm current_smokeless_n file3, fam(bin) link(identity)

estimates store I3

svy: glm daily_tobacco_any file4, fam(bin) link(identity)

estimates store I4

svy: glm daily_smoke_n file5, fam(bin) link(identity)

estimates store I5

svy: glm daily_smokeless_n file6, fam(bin) link(identity)

estimates store I6

svy: glm smoke_only file7, fam(bin) link(identity)

estimates store I7

svy: glm smokeless_only file8, fam(bin) link(identity)

estimates store I8

svy: glm both_smoke_smokeless file9, fam(bin) link(identity)

estimates store I9

svy: glm alcohol_current file10, fam(bin) link(identity)

estimates store I10

svy: glm no_fruit_veg file11, fam(bin) link(identity)

estimates store I11

svy: glm no_fruit file12, fam(bin) link(identity)

estimates store I12

svy: glm no_veg file13, fam(bin) link(identity)

estimates store I13

svy: glm atelessthan5 file14, fam(bin) link(identity)

estimates store I14

svy: glm salt_eat_n file15, fam(bin) link(identity)

estimates store I15

svy: glm salt_pack_n file16, fam(bin) link(identity)

estimates store I16

svy: glm htn_known file17, fam(bin) link(identity)

estimates store I17

svy: glm htn_med file18, fam(bin) link(identity)

estimates store I18

estout I1 I2 I3 I4 I5 I6 I7 I8 I9 I10 I11 I12 I13 I14 I15 I16 I17 I18 using Table_RD.xls, ///

cells ("b(star fmt(1)) ci(par fmt(1) label(95% CI)) p (fmt(4)) ") replace transform(@*100)

import delimited "Table_RD.xls", clear

replace v2 = v5 if v2 == ""

replace v2 = v8 if v2 == ""

replace v2 = v11 if v2 == ""

replace v2 = v14 if v2 == ""

replace v2 = v17 if v2 == ""

replace v2 = v20 if v2 == ""

replace v2 = v23 if v2 == ""

replace v2 = v26 if v2 == ""

replace v2 = v29 if v2 == ""

replace v2 = v32 if v2 == ""

replace v2 = v35 if v2 == ""

replace v2 = v38 if v2 == ""

replace v2 = v41 if v2 == ""

replace v2 = v44 if v2 == ""

replace v2 = v47 if v2 == ""

replace v2 = v50 if v2 == ""

replace v2 = v53 if v2 == ""

replace v3 = v6 if v3 == ""

replace v3 = v9 if v3 == ""

replace v3 = v12 if v3 == ""

replace v3 = v15 if v3 == ""

replace v3 = v18 if v3 == ""

replace v3 = v21 if v3 == ""

replace v3 = v24 if v3 == ""

replace v3 = v27 if v3 == ""

replace v3 = v30 if v3 == ""

replace v3 = v33 if v3 == ""

replace v3 = v36 if v3 == ""

replace v3 = v39 if v3 == ""

replace v3 = v42 if v3 == ""

replace v3 = v45 if v3 == ""

replace v3 = v48 if v3 == ""

replace v3 = v51 if v3 == ""

replace v3 = v54 if v3 == ""

replace v4 = v7 if v4 == ""

replace v4 = v10 if v4 == ""

replace v4 = v13 if v4 == ""

replace v4 = v16 if v4 == ""

replace v4 = v19 if v4 == ""

replace v4 = v22 if v4 == ""

replace v4 = v25 if v4 == ""

replace v4 = v28 if v4 == ""

replace v4 = v31 if v4 == ""

replace v4 = v34 if v4 == ""

replace v4 = v37 if v4 == ""

replace v4 = v40 if v4 == ""

replace v4 = v43 if v4 == ""

replace v4 = v46 if v4 == ""

replace v4 = v49 if v4 == ""

replace v4 = v52 if v4 == ""

replace v4 = v55 if v4 == ""

keep v1 v2 v3 v4

drop if v1 == ""

drop if v1 == "main"

drop if v1 == "_cons"

destring v1, replace ignore(file)

rename v2 Diff_IVR_IPW

rename v3 Diff_IVR_IPW_CI

rename v4 Diff_IVR_IPW_P

gen pop_group = 1

tostring v1, replace

replace v1 = "Any current tobacco user" if v1 == "1"

replace v1 = "Current smoker" if v1 == "2"

replace v1 = "Current smokeless tobacco user" if v1 == "3"

replace v1 = "Any daily tobacco user" if v1 == "4"

replace v1 = "Daily smoker" if v1 == "5"

replace v1 = "Daily smokeless tobacco user" if v1 == "6"

replace v1 = "Only smoker" if v1 == "7"

replace v1 = "Only smokeless tobacco user" if v1 == "8"

replace v1 = "Both smoker and smokeless user" if v1 == "9"

replace v1 = "Alcohol past month" if v1 == "10"

replace v1 = "No fruit or vegetables" if v1 == "11"

replace v1 = "No fruit" if v1 == "12"

replace v1 = "No veg" if v1 == "13"

replace v1 = "<5 servings of fruits-veg in a day " if v1 == "14"

replace v1 = "Add salt to food while eating" if v1 == "15"

replace v1 = "Processed food high in salt" if v1 == "16"

replace v1 = "Known raised BP/HTN" if v1 == "17"

replace v1 = "Take medication for BP/HTN" if v1 == "18"

save File_RD_IVR_IPW, replace

restore

********

preserve

recode file1 (1 = 0) (0 = 1)

keep if sex_n == 2

keep if age_cat == 2

svyset [pweight= age_sex_w]

gen file2 = file1

gen file3 = file1

gen file4 = file1

gen file5 = file1

gen file6 = file1

gen file7 = file1

gen file8 = file1

gen file9 = file1

gen file10 = file1

gen file11 = file1

gen file12 = file1

gen file13 = file1

gen file14 = file1

gen file15 = file1

gen file16 = file1

gen file17 = file1

gen file18 = file1

svy: glm current_tobacco_any file1, fam(bin) link(identity)

estimates store I1

svy: glm current_smoke_n file2, fam(bin) link(identity)

estimates store I2

svy: glm current_smokeless_n file3, fam(bin) link(identity)

estimates store I3

svy: glm daily_tobacco_any file4, fam(bin) link(identity)

estimates store I4

svy: glm daily_smoke_n file5, fam(bin) link(identity)

estimates store I5

svy: glm daily_smokeless_n file6, fam(bin) link(identity)

estimates store I6

svy: glm smoke_only file7, fam(bin) link(identity)

estimates store I7

svy: glm smokeless_only file8, fam(bin) link(identity)

estimates store I8

svy: glm both_smoke_smokeless file9, fam(bin) link(identity)

estimates store I9

svy: glm alcohol_current file10, fam(bin) link(identity)

estimates store I10

svy: glm no_fruit_veg file11, fam(bin) link(identity)

estimates store I11

svy: glm no_fruit file12, fam(bin) link(identity)

estimates store I12

svy: glm no_veg file13, fam(bin) link(identity)

estimates store I13

svy: glm atelessthan5 file14, fam(bin) link(identity)

estimates store I14

svy: glm salt_eat_n file15, fam(bin) link(identity)

estimates store I15

svy: glm salt_pack_n file16, fam(bin) link(identity)

estimates store I16

svy: glm htn_known file17, fam(bin) link(identity)

estimates store I17

svy: glm htn_med file18, fam(bin) link(identity)

estimates store I18

estout I1 I2 I3 I4 I5 I6 I7 I8 I9 I10 I11 I12 I13 I14 I15 I16 I17 I18 using Table_RD.xls, ///

cells ("b(star fmt(1)) ci(par fmt(1) label(95% CI)) p (fmt(4)) ") replace transform(@*100)

import delimited "Table_RD.xls", clear

replace v2 = v5 if v2 == ""

replace v2 = v8 if v2 == ""

replace v2 = v11 if v2 == ""

replace v2 = v14 if v2 == ""

replace v2 = v17 if v2 == ""

replace v2 = v20 if v2 == ""

replace v2 = v23 if v2 == ""

replace v2 = v26 if v2 == ""

replace v2 = v29 if v2 == ""

replace v2 = v32 if v2 == ""

replace v2 = v35 if v2 == ""

replace v2 = v38 if v2 == ""

replace v2 = v41 if v2 == ""

replace v2 = v44 if v2 == ""

replace v2 = v47 if v2 == ""

replace v2 = v50 if v2 == ""

replace v2 = v53 if v2 == ""

replace v3 = v6 if v3 == ""

replace v3 = v9 if v3 == ""

replace v3 = v12 if v3 == ""

replace v3 = v15 if v3 == ""

replace v3 = v18 if v3 == ""

replace v3 = v21 if v3 == ""

replace v3 = v24 if v3 == ""

replace v3 = v27 if v3 == ""

replace v3 = v30 if v3 == ""

replace v3 = v33 if v3 == ""

replace v3 = v36 if v3 == ""

replace v3 = v39 if v3 == ""

replace v3 = v42 if v3 == ""

replace v3 = v45 if v3 == ""

replace v3 = v48 if v3 == ""

replace v3 = v51 if v3 == ""

replace v3 = v54 if v3 == ""

replace v4 = v7 if v4 == ""

replace v4 = v10 if v4 == ""

replace v4 = v13 if v4 == ""

replace v4 = v16 if v4 == ""

replace v4 = v19 if v4 == ""

replace v4 = v22 if v4 == ""

replace v4 = v25 if v4 == ""

replace v4 = v28 if v4 == ""

replace v4 = v31 if v4 == ""

replace v4 = v34 if v4 == ""

replace v4 = v37 if v4 == ""

replace v4 = v40 if v4 == ""

replace v4 = v43 if v4 == ""

replace v4 = v46 if v4 == ""

replace v4 = v49 if v4 == ""

replace v4 = v52 if v4 == ""

replace v4 = v55 if v4 == ""

keep v1 v2 v3 v4

drop if v1 == ""

drop if v1 == "main"

drop if v1 == "_cons"

destring v1, replace ignore(file)

rename v2 Diff_IVR_Age_Sex

rename v3 Diff_IVR_Age_Sex_CI

rename v4 Diff_IVR_Age_Sex_P

gen pop_group = 1

gen number = v1

tostring v1, replace

replace v1 = "Any current tobacco user" if v1 == "1"

replace v1 = "Current smoker" if v1 == "2"

replace v1 = "Current smokeless tobacco user" if v1 == "3"

replace v1 = "Any daily tobacco user" if v1 == "4"

replace v1 = "Daily smoker" if v1 == "5"

replace v1 = "Daily smokeless tobacco user" if v1 == "6"

replace v1 = "Only smoker" if v1 == "7"

replace v1 = "Only smokeless tobacco user" if v1 == "8"

replace v1 = "Both smoker and smokeless user" if v1 == "9"

replace v1 = "Alcohol past month" if v1 == "10"

replace v1 = "No fruit or vegetables" if v1 == "11"

replace v1 = "No fruit" if v1 == "12"

replace v1 = "No veg" if v1 == "13"

replace v1 = "<5 servings of fruits-veg in a day " if v1 == "14"

replace v1 = "Add salt to food while eating" if v1 == "15"

replace v1 = "Processed food high in salt" if v1 == "16"

replace v1 = "Known raised BP/HTN" if v1 == "17"

replace v1 = "Take medication for BP/HTN" if v1 == "18"

save File_RD_IVR_Age_Sex, replace

restore

*******

preserve

use Table3_Unweighted.dta, replace

merge 1:1 v1 using Table3_Weighted_IPW, nogen

merge 1:1 v1 using Table3_Weighted_Age_Sex_W.dta, nogen

merge 1:1 v1 using Table3_n.dta, nogen

sort id

drop id

label var v1 "Indicator"

drop STEPS_IPW

gen indicator = _n

gen pop_group = 11

save Table3_11_Prevalence_GMAK_20Jul2022.dta, replace

merge 1:1 v1 using File_RD_IVR_IPW.dta, nogen

merge 1:1 v1 using File_RD_IVR_Age_Sex.dta, nogen

sort number

order v1 IVR_n IVR_IPW IVR_IPW_CI STEPS_n STEPS_Age_Sex STEPS_Age_Sex_CI Diff_IVR_IPW

export excel v1 IVR_n IVR_IPW IVR_IPW_CI STEPS_n STEPS_Age_Sex STEPS_Age_Sex_CI ///

Diff_IVR_IPW using SumTab_IVR_IPWvsSTEPS.xls, replace

order v1 IVR_n IVR_Age_Sex IVR_Age_Sex_CI STEPS_n STEPS_Age_Sex STEPS_Age_Sex_CI ///

Diff_IVR_Age_Sex

export excel v1 IVR_n IVR_Age_Sex IVR_Age_Sex_CI STEPS_n STEPS_Age_Sex STEPS_Age_Sex_CI ///

Diff_IVR_Age_Sex using SumTab_IVR_AgeSexvsSTEPS.xls, replace

restore

cap erase Table3_Unweighted.dta

cap erase Table3_Weighted_IPW.dta

cap erase Table3_Weighted_Age_Sex_W.dta

cap erase Table3_Weight_Age_Sex_W.xls

cap erase Table3_Weight_IPW.xls

cap erase Table3_Unweight.xls

********************************************************************************

preserve

keep if sex_n == 2

tabout current_tobacco_any current_smoke_n current_smokeless_n ///

daily_tobacco_any daily_smoke_n daily_smokeless_n ///

smoke_only smokeless_only both_smoke_smokeless ///

alcohol_current no_fruit_veg no_fruit no_veg ///

atelessthan5 salt_eat_n salt_pack_n ///

htn_known htn_med file1 ///

using Table3_n.xls, ///

c(freq col) f(1c 1p) replace

import delimited using Table3_n.xls, clear

drop if v1 == "No"

drop if v1 == "Yes"

replace v1 = v1[_n - 1] if v1 == "Total"

drop v3 v5 v6 v7

drop if v1 == ""

drop if v2 == ""

destring v2 v4, ignore(",") replace

rename v2 IVR_n

rename v4 STEPS_n

gen id = _n

save Table3_n.dta, replace

restore

preserve

keep if sex_n == 2

gen wt = 1

svyset [pweight= wt]

tabout current_tobacco_any current_smoke_n current_smokeless_n ///

daily_tobacco_any daily_smoke_n daily_smokeless_n ///

smoke_only smokeless_only both_smoke_smokeless ///

alcohol_current no_fruit_veg no_fruit no_veg ///

atelessthan5 salt_eat_n salt_pack_n ///

htn_known htn_med file1 ///

using Table3_Unweight.xls, ///

c(col ci) f(1 1) percent svy replace

import delimited using Table3_Unweight.xls, clear

drop v6 v7

drop if v1 == "No" | v1 == "Total"

replace v1 = v1[_n - 1] if v1 == "Yes"

drop if v2 == ""

drop if v1 == ""

rename v2 IVR_Unweight

rename v4 STEPS_Unweight

rename v3 IVR_Unweight_CI

rename v5 STEPS_Unweight_CI

gen id = _n

save Table3_Unweighted.dta, replace

restore

********

preserve

keep if sex_n == 2

svyset [pweight= weight_ipw]

tabout current_tobacco_any current_smoke_n current_smokeless_n ///

daily_tobacco_any daily_smoke_n daily_smokeless_n ///

smoke_only smokeless_only both_smoke_smokeless ///

alcohol_current no_fruit_veg no_fruit no_veg ///

atelessthan5 salt_eat_n salt_pack_n ///

htn_known htn_med file1 ///

using Table3_Weight_IPW.xls, ///

c(col ci) f(1 1) percent svy replace

import delimited using Table3_Weight_IPW.xls, clear

drop v6 v7

drop if v1 == "No" | v1 == "Total"

replace v1 = v1[_n - 1] if v1 == "Yes"

drop if v2 == ""

drop if v1 == ""

rename v2 IVR_IPW

rename v4 STEPS_IPW

rename v3 IVR_IPW_CI

rename v5 STEPS_IPW_CI

gen id = _n

save Table3_Weighted_IPW.dta, replace

restore

********

preserve

keep if sex_n == 2

svyset [pweight= age_sex_w]

tabout current_tobacco_any current_smoke_n current_smokeless_n ///

daily_tobacco_any daily_smoke_n daily_smokeless_n ///

smoke_only smokeless_only both_smoke_smokeless ///

alcohol_current no_fruit_veg no_fruit no_veg ///

atelessthan5 salt_eat_n salt_pack_n ///

htn_known htn_med file1 ///

using Table3_Weight_Age_Sex_W.xls, ///

c(col ci) f(1 1) percent svy replace

import delimited using Table3_Weight_Age_Sex_W.xls, clear

drop v6 v7

drop if v1 == "No" | v1 == "Total"

replace v1 = v1[_n - 1] if v1 == "Yes"

drop if v2 == ""

drop if v1 == ""

rename v2 IVR_Age_Sex

rename v4 STEPS_Age_Sex

rename v3 IVR_Age_Sex_CI

rename v5 STEPS_Age_Sex_CI

gen id = _n

save Table3_Weighted_Age_Sex_W.dta, replace

restore

****

preserve

recode file1 (1 = 0) (0 = 1)

keep if sex_n == 2

svyset [pweight= weight_ipw]

gen file2 = file1

gen file3 = file1

gen file4 = file1

gen file5 = file1

gen file6 = file1

gen file7 = file1

gen file8 = file1

gen file9 = file1

gen file10 = file1

gen file11 = file1

gen file12 = file1

gen file13 = file1

gen file14 = file1

gen file15 = file1

gen file16 = file1

gen file17 = file1

gen file18 = file1

svy: glm current_tobacco_any file1, fam(bin) link(identity)

estimates store I1

svy: glm current_smoke_n file2, fam(bin) link(identity)

estimates store I2

svy: glm current_smokeless_n file3, fam(bin) link(identity)

estimates store I3

svy: glm daily_tobacco_any file4, fam(bin) link(identity)

estimates store I4

svy: glm daily_smoke_n file5, fam(bin) link(identity)

estimates store I5

svy: glm daily_smokeless_n file6, fam(bin) link(identity)

estimates store I6

svy: glm smoke_only file7, fam(bin) link(identity)

estimates store I7

svy: glm smokeless_only file8, fam(bin) link(identity)

estimates store I8

svy: glm both_smoke_smokeless file9, fam(bin) link(identity)

estimates store I9

svy: glm alcohol_current file10, fam(bin) link(identity)

estimates store I10

svy: glm no_fruit_veg file11, fam(bin) link(identity)

estimates store I11

svy: glm no_fruit file12, fam(bin) link(identity)

estimates store I12

svy: glm no_veg file13, fam(bin) link(identity)

estimates store I13

svy: glm atelessthan5 file14, fam(bin) link(identity)

estimates store I14

svy: glm salt_eat_n file15, fam(bin) link(identity)

estimates store I15

svy: glm salt_pack_n file16, fam(bin) link(identity)

estimates store I16

svy: glm htn_known file17, fam(bin) link(identity)

estimates store I17

svy: glm htn_med file18, fam(bin) link(identity)

estimates store I18

estout I1 I2 I3 I4 I5 I6 I7 I8 I9 I10 I11 I12 I13 I14 I15 I16 I17 I18 using Table_RD.xls, ///

cells ("b(star fmt(1)) ci(par fmt(1) label(95% CI)) p (fmt(4)) ") replace transform(@*100)

import delimited "Table_RD.xls", clear

replace v2 = v5 if v2 == ""

replace v2 = v8 if v2 == ""

replace v2 = v11 if v2 == ""

replace v2 = v14 if v2 == ""

replace v2 = v17 if v2 == ""

replace v2 = v20 if v2 == ""

replace v2 = v23 if v2 == ""

replace v2 = v26 if v2 == ""

replace v2 = v29 if v2 == ""

replace v2 = v32 if v2 == ""

replace v2 = v35 if v2 == ""

replace v2 = v38 if v2 == ""

replace v2 = v41 if v2 == ""

replace v2 = v44 if v2 == ""

replace v2 = v47 if v2 == ""

replace v2 = v50 if v2 == ""

replace v2 = v53 if v2 == ""

replace v3 = v6 if v3 == ""

replace v3 = v9 if v3 == ""

replace v3 = v12 if v3 == ""

replace v3 = v15 if v3 == ""

replace v3 = v18 if v3 == ""

replace v3 = v21 if v3 == ""

replace v3 = v24 if v3 == ""

replace v3 = v27 if v3 == ""

replace v3 = v30 if v3 == ""

replace v3 = v33 if v3 == ""

replace v3 = v36 if v3 == ""

replace v3 = v39 if v3 == ""

replace v3 = v42 if v3 == ""

replace v3 = v45 if v3 == ""

replace v3 = v48 if v3 == ""

replace v3 = v51 if v3 == ""

replace v3 = v54 if v3 == ""

replace v4 = v7 if v4 == ""

replace v4 = v10 if v4 == ""

replace v4 = v13 if v4 == ""

replace v4 = v16 if v4 == ""

replace v4 = v19 if v4 == ""

replace v4 = v22 if v4 == ""

replace v4 = v25 if v4 == ""

replace v4 = v28 if v4 == ""

replace v4 = v31 if v4 == ""

replace v4 = v34 if v4 == ""

replace v4 = v37 if v4 == ""

replace v4 = v40 if v4 == ""

replace v4 = v43 if v4 == ""

replace v4 = v46 if v4 == ""

replace v4 = v49 if v4 == ""

replace v4 = v52 if v4 == ""

replace v4 = v55 if v4 == ""

keep v1 v2 v3 v4

drop if v1 == ""

drop if v1 == "main"

drop if v1 == "_cons"

destring v1, replace ignore(file)

rename v2 Diff_IVR_IPW

rename v3 Diff_IVR_IPW_CI

rename v4 Diff_IVR_IPW_P

gen pop_group = 1

tostring v1, replace

replace v1 = "Any current tobacco user" if v1 == "1"

replace v1 = "Current smoker" if v1 == "2"

replace v1 = "Current smokeless tobacco user" if v1 == "3"

replace v1 = "Any daily tobacco user" if v1 == "4"

replace v1 = "Daily smoker" if v1 == "5"

replace v1 = "Daily smokeless tobacco user" if v1 == "6"

replace v1 = "Only smoker" if v1 == "7"

replace v1 = "Only smokeless tobacco user" if v1 == "8"

replace v1 = "Both smoker and smokeless user" if v1 == "9"

replace v1 = "Alcohol past month" if v1 == "10"

replace v1 = "No fruit or vegetables" if v1 == "11"

replace v1 = "No fruit" if v1 == "12"

replace v1 = "No veg" if v1 == "13"

replace v1 = "<5 servings of fruits-veg in a day " if v1 == "14"

replace v1 = "Add salt to food while eating" if v1 == "15"

replace v1 = "Processed food high in salt" if v1 == "16"

replace v1 = "Known raised BP/HTN" if v1 == "17"

replace v1 = "Take medication for BP/HTN" if v1 == "18"

save File_RD_IVR_IPW, replace

restore

********

preserve

recode file1 (1 = 0) (0 = 1)

keep if sex_n == 2

svyset [pweight= age_sex_w]

gen file2 = file1

gen file3 = file1

gen file4 = file1

gen file5 = file1

gen file6 = file1

gen file7 = file1

gen file8 = file1

gen file9 = file1

gen file10 = file1

gen file11 = file1

gen file12 = file1

gen file13 = file1

gen file14 = file1

gen file15 = file1

gen file16 = file1

gen file17 = file1

gen file18 = file1

svy: glm current_tobacco_any file1, fam(bin) link(identity)

estimates store I1

svy: glm current_smoke_n file2, fam(bin) link(identity)

estimates store I2

svy: glm current_smokeless_n file3, fam(bin) link(identity)

estimates store I3

svy: glm daily_tobacco_any file4, fam(bin) link(identity)

estimates store I4

svy: glm daily_smoke_n file5, fam(bin) link(identity)

estimates store I5

svy: glm daily_smokeless_n file6, fam(bin) link(identity)

estimates store I6

svy: glm smoke_only file7, fam(bin) link(identity)

estimates store I7

svy: glm smokeless_only file8, fam(bin) link(identity)

estimates store I8

svy: glm both_smoke_smokeless file9, fam(bin) link(identity)

estimates store I9

svy: glm alcohol_current file10, fam(bin) link(identity)

estimates store I10

svy: glm no_fruit_veg file11, fam(bin) link(identity)

estimates store I11

svy: glm no_fruit file12, fam(bin) link(identity)

estimates store I12

svy: glm no_veg file13, fam(bin) link(identity)

estimates store I13

svy: glm atelessthan5 file14, fam(bin) link(identity)

estimates store I14

svy: glm salt_eat_n file15, fam(bin) link(identity)

estimates store I15

svy: glm salt_pack_n file16, fam(bin) link(identity)

estimates store I16

svy: glm htn_known file17, fam(bin) link(identity)

estimates store I17

svy: glm htn_med file18, fam(bin) link(identity)

estimates store I18

estout I1 I2 I3 I4 I5 I6 I7 I8 I9 I10 I11 I12 I13 I14 I15 I16 I17 I18 using Table_RD.xls, ///

cells ("b(star fmt(1)) ci(par fmt(1) label(95% CI)) p (fmt(4)) ") replace transform(@*100)

import delimited "Table_RD.xls", clear

replace v2 = v5 if v2 == ""

replace v2 = v8 if v2 == ""

replace v2 = v11 if v2 == ""

replace v2 = v14 if v2 == ""

replace v2 = v17 if v2 == ""

replace v2 = v20 if v2 == ""

replace v2 = v23 if v2 == ""

replace v2 = v26 if v2 == ""

replace v2 = v29 if v2 == ""

replace v2 = v32 if v2 == ""

replace v2 = v35 if v2 == ""

replace v2 = v38 if v2 == ""

replace v2 = v41 if v2 == ""

replace v2 = v44 if v2 == ""

replace v2 = v47 if v2 == ""

replace v2 = v50 if v2 == ""

replace v2 = v53 if v2 == ""

replace v3 = v6 if v3 == ""

replace v3 = v9 if v3 == ""

replace v3 = v12 if v3 == ""

replace v3 = v15 if v3 == ""

replace v3 = v18 if v3 == ""

replace v3 = v21 if v3 == ""

replace v3 = v24 if v3 == ""

replace v3 = v27 if v3 == ""

replace v3 = v30 if v3 == ""

replace v3 = v33 if v3 == ""

replace v3 = v36 if v3 == ""

replace v3 = v39 if v3 == ""

replace v3 = v42 if v3 == ""

replace v3 = v45 if v3 == ""

replace v3 = v48 if v3 == ""

replace v3 = v51 if v3 == ""

replace v3 = v54 if v3 == ""

replace v4 = v7 if v4 == ""

replace v4 = v10 if v4 == ""

replace v4 = v13 if v4 == ""

replace v4 = v16 if v4 == ""

replace v4 = v19 if v4 == ""

replace v4 = v22 if v4 == ""

replace v4 = v25 if v4 == ""

replace v4 = v28 if v4 == ""

replace v4 = v31 if v4 == ""

replace v4 = v34 if v4 == ""

replace v4 = v37 if v4 == ""

replace v4 = v40 if v4 == ""

replace v4 = v43 if v4 == ""

replace v4 = v46 if v4 == ""

replace v4 = v49 if v4 == ""

replace v4 = v52 if v4 == ""

replace v4 = v55 if v4 == ""

keep v1 v2 v3 v4

drop if v1 == ""

drop if v1 == "main"

drop if v1 == "_cons"

destring v1, replace ignore(file)

rename v2 Diff_IVR_Age_Sex

rename v3 Diff_IVR_Age_Sex_CI

rename v4 Diff_IVR_Age_Sex_P

gen pop_group = 1

gen number = v1

tostring v1, replace

replace v1 = "Any current tobacco user" if v1 == "1"

replace v1 = "Current smoker" if v1 == "2"

replace v1 = "Current smokeless tobacco user" if v1 == "3"

replace v1 = "Any daily tobacco user" if v1 == "4"

replace v1 = "Daily smoker" if v1 == "5"

replace v1 = "Daily smokeless tobacco user" if v1 == "6"

replace v1 = "Only smoker" if v1 == "7"

replace v1 = "Only smokeless tobacco user" if v1 == "8"

replace v1 = "Both smoker and smokeless user" if v1 == "9"

replace v1 = "Alcohol past month" if v1 == "10"

replace v1 = "No fruit or vegetables" if v1 == "11"

replace v1 = "No fruit" if v1 == "12"

replace v1 = "No veg" if v1 == "13"

replace v1 = "<5 servings of fruits-veg in a day " if v1 == "14"

replace v1 = "Add salt to food while eating" if v1 == "15"

replace v1 = "Processed food high in salt" if v1 == "16"

replace v1 = "Known raised BP/HTN" if v1 == "17"

replace v1 = "Take medication for BP/HTN" if v1 == "18"

save File_RD_IVR_Age_Sex, replace

restore

*******

preserve

use Table3_Unweighted.dta, replace

merge 1:1 v1 using Table3_Weighted_IPW, nogen

merge 1:1 v1 using Table3_Weighted_Age_Sex_W.dta, nogen

merge 1:1 v1 using Table3_n.dta, nogen

sort id

drop id

label var v1 "Indicator"

drop STEPS_IPW

gen indicator = _n

gen pop_group = 12

save Table3_12_Prevalence_GMAK_20Jul2022.dta, replace

merge 1:1 v1 using File_RD_IVR_IPW.dta, nogen

merge 1:1 v1 using File_RD_IVR_Age_Sex.dta, nogen

sort number

order v1 IVR_n IVR_IPW IVR_IPW_CI STEPS_n STEPS_Age_Sex STEPS_Age_Sex_CI Diff_IVR_IPW

export excel v1 IVR_n IVR_IPW IVR_IPW_CI STEPS_n STEPS_Age_Sex STEPS_Age_Sex_CI ///

Diff_IVR_IPW using SumTab_IVR_IPWvsSTEPS.xls, replace

order v1 IVR_n IVR_Age_Sex IVR_Age_Sex_CI STEPS_n STEPS_Age_Sex STEPS_Age_Sex_CI ///

Diff_IVR_Age_Sex

export excel v1 IVR_n IVR_Age_Sex IVR_Age_Sex_CI STEPS_n STEPS_Age_Sex STEPS_Age_Sex_CI ///

Diff_IVR_Age_Sex using SumTab_IVR_AgeSexvsSTEPS.xls, replace

restore

********************************************************************************

preserve

keep if sex_n == 1

keep if age_cat == 0

keep if school_n == 0

tabout current_tobacco_any current_smoke_n current_smokeless_n ///

daily_tobacco_any daily_smoke_n daily_smokeless_n ///

smoke_only smokeless_only both_smoke_smokeless ///

alcohol_current no_fruit_veg no_fruit no_veg ///

atelessthan5 salt_eat_n salt_pack_n ///

htn_known htn_med file1 ///

using Table3_n.xls, ///

c(freq col) f(1c 1p) replace

import delimited using Table3_n.xls, clear

drop if v1 == "No"

drop if v1 == "Yes"

replace v1 = v1[_n - 1] if v1 == "Total"

drop v3 v5 v6 v7

drop if v1 == ""

drop if v2 == ""

destring v2 v4, ignore(",") replace

rename v2 IVR_n

rename v4 STEPS_n

gen id = _n

save Table3_n.dta, replace

restore

preserve

keep if sex_n == 1

keep if age_cat == 0

keep if school_n == 0

gen wt = 1

svyset [pweight= wt]

tabout current_tobacco_any current_smoke_n current_smokeless_n ///

daily_tobacco_any daily_smoke_n daily_smokeless_n ///

smoke_only smokeless_only both_smoke_smokeless ///

alcohol_current no_fruit_veg no_fruit no_veg ///

atelessthan5 salt_eat_n salt_pack_n ///

htn_known htn_med file1 ///

using Table3_Unweight.xls, ///

c(col ci) f(1 1) percent svy replace

import delimited using Table3_Unweight.xls, clear

drop v6 v7

drop if v1 == "No" | v1 == "Total"

replace v1 = v1[_n - 1] if v1 == "Yes"

drop if v2 == ""

drop if v1 == ""

rename v2 IVR_Unweight

rename v4 STEPS_Unweight

rename v3 IVR_Unweight_CI

rename v5 STEPS_Unweight_CI

gen id = _n

save Table3_Unweighted.dta, replace

restore

********

preserve

keep if sex_n == 1

keep if age_cat == 0

keep if school_n == 0

svyset [pweight= weight_ipw]

tabout current_tobacco_any current_smoke_n current_smokeless_n ///

daily_tobacco_any daily_smoke_n daily_smokeless_n ///

smoke_only smokeless_only both_smoke_smokeless ///

alcohol_current no_fruit_veg no_fruit no_veg ///

atelessthan5 salt_eat_n salt_pack_n ///

htn_known htn_med file1 ///

using Table3_Weight_IPW.xls, ///

c(col ci) f(1 1) percent svy replace

import delimited using Table3_Weight_IPW.xls, clear

drop v6 v7

drop if v1 == "No" | v1 == "Total"

replace v1 = v1[_n - 1] if v1 == "Yes"

drop if v2 == ""

drop if v1 == ""

rename v2 IVR_IPW

rename v4 STEPS_IPW

rename v3 IVR_IPW_CI

rename v5 STEPS_IPW_CI

gen id = _n

save Table3_Weighted_IPW.dta, replace

restore

********

preserve

keep if sex_n == 1

keep if age_cat == 0

keep if school_n == 0

svyset [pweight= age_sex_w]

tabout current_tobacco_any current_smoke_n current_smokeless_n ///

daily_tobacco_any daily_smoke_n daily_smokeless_n ///

smoke_only smokeless_only both_smoke_smokeless ///

alcohol_current no_fruit_veg no_fruit no_veg ///

atelessthan5 salt_eat_n salt_pack_n ///

htn_known htn_med file1 ///

using Table3_Weight_Age_Sex_W.xls, ///

c(col ci) f(1 1) percent svy replace

import delimited using Table3_Weight_Age_Sex_W.xls, clear

drop v6 v7

drop if v1 == "No" | v1 == "Total"

replace v1 = v1[_n - 1] if v1 == "Yes"

drop if v2 == ""

drop if v1 == ""

rename v2 IVR_Age_Sex

rename v4 STEPS_Age_Sex

rename v3 IVR_Age_Sex_CI

rename v5 STEPS_Age_Sex_CI

gen id = _n

save Table3_Weighted_Age_Sex_W.dta, replace

restore

preserve

use Table3_Unweighted.dta, replace

merge 1:1 v1 using Table3_Weighted_IPW, nogen

merge 1:1 v1 using Table3_Weighted_Age_Sex_W.dta, nogen

merge 1:1 v1 using Table3_n.dta, nogen

sort id

drop id

label var v1 "Indicator"

drop STEPS_IPW

gen indicator = _n

gen pop_group = 13

save Table3_13_Prevalence_GMAK_20Jul2022.dta, replace

restore

cap erase Table3_Unweighted.dta

cap erase Table3_Weighted_IPW.dta

cap erase Table3_Weighted_Age_Sex_W.dta

cap erase Table3_Weight_Age_Sex_W.xls

cap erase Table3_Weight_IPW.xls

cap erase Table3_Unweight.xls

********************************************************************************

preserve

keep if sex_n == 1

keep if age_cat == 0

keep if school_n == 1

tabout current_tobacco_any current_smoke_n current_smokeless_n ///

daily_tobacco_any daily_smoke_n daily_smokeless_n ///

smoke_only smokeless_only both_smoke_smokeless ///

alcohol_current no_fruit_veg no_fruit no_veg ///

atelessthan5 salt_eat_n salt_pack_n ///

htn_known htn_med file1 ///

using Table3_n.xls, ///

c(freq col) f(1c 1p) replace

import delimited using Table3_n.xls, clear

drop if v1 == "No"

drop if v1 == "Yes"

replace v1 = v1[_n - 1] if v1 == "Total"

drop v3 v5 v6 v7

drop if v1 == ""

drop if v2 == ""

destring v2 v4, ignore(",") replace

rename v2 IVR_n

rename v4 STEPS_n

gen id = _n

save Table3_n.dta, replace

restore

preserve

keep if sex_n == 1

keep if age_cat == 0

keep if school_n == 1

gen wt = 1

svyset [pweight= wt]

tabout current_tobacco_any current_smoke_n current_smokeless_n ///

daily_tobacco_any daily_smoke_n daily_smokeless_n ///

smoke_only smokeless_only both_smoke_smokeless ///

alcohol_current no_fruit_veg no_fruit no_veg ///

atelessthan5 salt_eat_n salt_pack_n ///

htn_known htn_med file1 ///

using Table3_Unweight.xls, ///

c(col ci) f(1 1) percent svy replace

import delimited using Table3_Unweight.xls, clear

drop v6 v7

drop if v1 == "No" | v1 == "Total"

replace v1 = v1[_n - 1] if v1 == "Yes"

drop if v2 == ""

drop if v1 == ""

rename v2 IVR_Unweight

rename v4 STEPS_Unweight

rename v3 IVR_Unweight_CI

rename v5 STEPS_Unweight_CI

gen id = _n

save Table3_Unweighted.dta, replace

restore

********

preserve

keep if sex_n == 1

keep if age_cat == 0

keep if school_n == 1

svyset [pweight= weight_ipw]

tabout current_tobacco_any current_smoke_n current_smokeless_n ///

daily_tobacco_any daily_smoke_n daily_smokeless_n ///

smoke_only smokeless_only both_smoke_smokeless ///

alcohol_current no_fruit_veg no_fruit no_veg ///

atelessthan5 salt_eat_n salt_pack_n ///

htn_known htn_med file1 ///

using Table3_Weight_IPW.xls, ///

c(col ci) f(1 1) percent svy replace

import delimited using Table3_Weight_IPW.xls, clear

drop v6 v7

drop if v1 == "No" | v1 == "Total"

replace v1 = v1[_n - 1] if v1 == "Yes"

drop if v2 == ""

drop if v1 == ""

rename v2 IVR_IPW

rename v4 STEPS_IPW

rename v3 IVR_IPW_CI

rename v5 STEPS_IPW_CI

gen id = _n

save Table3_Weighted_IPW.dta, replace

restore

********

preserve

keep if sex_n == 1

keep if age_cat == 0

keep if school_n == 1

svyset [pweight= age_sex_w]

tabout current_tobacco_any current_smoke_n current_smokeless_n ///

daily_tobacco_any daily_smoke_n daily_smokeless_n ///

smoke_only smokeless_only both_smoke_smokeless ///

alcohol_current no_fruit_veg no_fruit no_veg ///

atelessthan5 salt_eat_n salt_pack_n ///

htn_known htn_med file1 ///

using Table3_Weight_Age_Sex_W.xls, ///

c(col ci) f(1 1) percent svy replace

import delimited using Table3_Weight_Age_Sex_W.xls, clear

drop v6 v7

drop if v1 == "No" | v1 == "Total"

replace v1 = v1[_n - 1] if v1 == "Yes"

drop if v2 == ""

drop if v1 == ""

rename v2 IVR_Age_Sex

rename v4 STEPS_Age_Sex

rename v3 IVR_Age_Sex_CI

rename v5 STEPS_Age_Sex_CI

gen id = _n

save Table3_Weighted_Age_Sex_W.dta, replace

restore

preserve

use Table3_Unweighted.dta, replace

merge 1:1 v1 using Table3_Weighted_IPW, nogen

merge 1:1 v1 using Table3_Weighted_Age_Sex_W.dta, nogen

merge 1:1 v1 using Table3_n.dta, nogen

sort id

drop id

label var v1 "Indicator"

drop STEPS_IPW

gen indicator = _n

gen pop_group = 14

save Table3_14_Prevalence_GMAK_20Jul2022.dta, replace

restore

cap erase Table3_Unweighted.dta

cap erase Table3_Weighted_IPW.dta

cap erase Table3_Weighted_Age_Sex_W.dta

cap erase Table3_Weight_Age_Sex_W.xls

cap erase Table3_Weight_IPW.xls

cap erase Table3_Unweight.xls

********************************************************************************

use working_file, replace

preserve

keep if sex_n == 1

keep if age_cat == 0

keep if school_n == 2

tabout current_tobacco_any current_smoke_n current_smokeless_n ///

daily_tobacco_any daily_smoke_n daily_smokeless_n ///

smoke_only smokeless_only both_smoke_smokeless ///

alcohol_current no_fruit_veg no_fruit no_veg ///

atelessthan5 salt_eat_n salt_pack_n ///

htn_known htn_med file1 ///

using Table3_n.xls, ///

c(freq col) f(1c 1p) replace

import delimited using Table3_n.xls, clear

drop if v1 == "No"

drop if v1 == "Yes"

replace v1 = v1[_n - 1] if v1 == "Total"

drop v3 v5 v6 v7

drop if v1 == ""

drop if v2 == ""

destring v2 v4, ignore(",") replace

rename v2 IVR_n

rename v4 STEPS_n

gen id = _n

save Table3_n.dta, replace

restore

preserve

keep if sex_n == 1

keep if age_cat == 0

keep if school_n == 2

gen wt = 1

svyset [pweight= wt]

gen file2 = 0 if file1 == 1

replace file2 = 1 if file1 == 0

for var current_tobacco_any current_smoke_n current_smokeless_n ///

daily_tobacco_any daily_smoke_n daily_smokeless_n ///

smoke_only smokeless_only both_smoke_smokeless ///

alcohol_current no_fruit_veg no_fruit no_veg ///

atelessthan5 salt_eat_n salt_pack_n ///

htn_known htn_med: tab X file1

tabout current_tobacco_any current_smoke_n current_smokeless_n ///

daily_tobacco_any daily_smoke_n ///

smoke_only smokeless_only ///

no_fruit_veg no_fruit no_veg ///

atelessthan5 salt_eat_n salt_pack_n ///

htn_known htn_med file1 ///

using Table3_Unweight.xls, ///

c(col ci) f(1 1) percent svy replace

import delimited using Table3_Unweight.xls, clear

drop v6 v7

drop if v1 == "No" | v1 == "Total"

replace v1 = v1[_n - 1] if v1 == "Yes"

drop if v2 == ""

drop if v1 == ""

rename v2 IVR_Unweight

rename v4 STEPS_Unweight

rename v3 IVR_Unweight_CI

rename v5 STEPS_Unweight_CI

gen id = _n

save Table3_Unweighted.dta, replace

restore

********

preserve

keep if sex_n == 1

keep if age_cat == 0

keep if school_n == 2

svyset [pweight= weight_ipw]

tabout current_tobacco_any current_smoke_n current_smokeless_n ///

daily_tobacco_any daily_smoke_n ///

smoke_only smokeless_only ///

no_fruit_veg no_fruit no_veg ///

atelessthan5 salt_eat_n salt_pack_n ///

htn_known htn_med file1 ///

using Table3_Weight_IPW.xls, ///

c(col ci) f(1 1) percent svy replace chkwtnone

import delimited using Table3_Weight_IPW.xls, clear

drop v6 v7

drop if v1 == "No" | v1 == "Total"

replace v1 = v1[_n - 1] if v1 == "Yes"

drop if v2 == ""

drop if v1 == ""

rename v2 IVR_IPW

rename v4 STEPS_IPW

rename v3 IVR_IPW_CI

rename v5 STEPS_IPW_CI

gen id = _n

save Table3_Weighted_IPW.dta, replace

restore

********

preserve

keep if sex_n == 1

keep if age_cat == 0

keep if school_n == 2

svyset [pweight= age_sex_w]

tabout current_tobacco_any current_smoke_n current_smokeless_n ///

daily_tobacco_any daily_smoke_n ///

smoke_only smokeless_only ///

no_fruit_veg no_fruit no_veg ///

atelessthan5 salt_eat_n salt_pack_n ///

htn_known htn_med file1 ///

using Table3_Weight_Age_Sex_W.xls, ///

c(col ci) f(1 1) percent svy replace chkwtnone

import delimited using Table3_Weight_Age_Sex_W.xls, clear

drop v6 v7

drop if v1 == "No" | v1 == "Total"

replace v1 = v1[_n - 1] if v1 == "Yes"

drop if v2 == ""

drop if v1 == ""

rename v2 IVR_Age_Sex

rename v4 STEPS_Age_Sex

rename v3 IVR_Age_Sex_CI

rename v5 STEPS_Age_Sex_CI

gen id = _n

save Table3_Weighted_Age_Sex_W.dta, replace

restore

preserve

use Table3_Unweighted.dta, replace

merge 1:1 v1 using Table3_Weighted_IPW, nogen

merge 1:1 v1 using Table3_Weighted_Age_Sex_W.dta, nogen

merge 1:1 v1 using Table3_n.dta, nogen

sort id

drop id

label var v1 "Indicator"

drop STEPS_IPW

gen indicator = _n

gen pop_group = 15

save Table3_15_Prevalence_GMAK_20Jul2022.dta, replace

restore

cap erase Table3_Unweighted.dta

cap erase Table3_Weighted_IPW.dta

cap erase Table3_Weighted_Age_Sex_W.dta

cap erase Table3_Weight_Age_Sex_W.xls

cap erase Table3_Weight_IPW.xls

cap erase Table3_Unweight.xls

********************************************************************************

preserve

keep if sex_n == 1

keep if age_cat == 1

keep if school_n == 0

tabout current_tobacco_any current_smoke_n current_smokeless_n ///

daily_tobacco_any daily_smoke_n daily_smokeless_n ///

smoke_only smokeless_only both_smoke_smokeless ///

alcohol_current no_fruit_veg no_fruit no_veg ///

atelessthan5 salt_eat_n salt_pack_n ///

htn_known htn_med file1 ///

using Table3_n.xls, ///

c(freq col) f(1c 1p) replace

import delimited using Table3_n.xls, clear

drop if v1 == "No"

drop if v1 == "Yes"

replace v1 = v1[_n - 1] if v1 == "Total"

drop v3 v5 v6 v7

drop if v1 == ""

drop if v2 == ""

destring v2 v4, ignore(",") replace

rename v2 IVR_n

rename v4 STEPS_n

gen id = _n

save Table3_n.dta, replace

restore

preserve

keep if sex_n == 1

keep if age_cat == 1

keep if school_n == 0

gen wt = 1

svyset [pweight= wt]

tabout current_tobacco_any current_smoke_n current_smokeless_n ///

daily_tobacco_any daily_smoke_n daily_smokeless_n ///

smoke_only smokeless_only both_smoke_smokeless ///

alcohol_current no_fruit_veg no_fruit no_veg ///

atelessthan5 salt_eat_n salt_pack_n ///

htn_known htn_med file1 ///

using Table3_Unweight.xls, ///

c(col ci) f(1 1) percent svy replace

import delimited using Table3_Unweight.xls, clear

drop v6 v7

drop if v1 == "No" | v1 == "Total"

replace v1 = v1[_n - 1] if v1 == "Yes"

drop if v2 == ""

drop if v1 == ""

rename v2 IVR_Unweight

rename v4 STEPS_Unweight

rename v3 IVR_Unweight_CI

rename v5 STEPS_Unweight_CI

gen id = _n

save Table3_Unweighted.dta, replace

restore

********

preserve

keep if sex_n == 1

keep if age_cat == 1

keep if school_n == 0

svyset [pweight= weight_ipw]

tabout current_tobacco_any current_smoke_n current_smokeless_n ///

daily_tobacco_any daily_smoke_n daily_smokeless_n ///

smoke_only smokeless_only both_smoke_smokeless ///

alcohol_current no_fruit_veg no_fruit no_veg ///

atelessthan5 salt_eat_n salt_pack_n ///

htn_known htn_med file1 ///

using Table3_Weight_IPW.xls, ///

c(col ci) f(1 1) percent svy replace

import delimited using Table3_Weight_IPW.xls, clear

drop v6 v7

drop if v1 == "No" | v1 == "Total"

replace v1 = v1[_n - 1] if v1 == "Yes"

drop if v2 == ""

drop if v1 == ""

rename v2 IVR_IPW

rename v4 STEPS_IPW

rename v3 IVR_IPW_CI

rename v5 STEPS_IPW_CI

gen id = _n

save Table3_Weighted_IPW.dta, replace

restore

********

preserve

keep if sex_n == 1

keep if age_cat == 1

keep if school_n == 0

svyset [pweight= age_sex_w]

tabout current_tobacco_any current_smoke_n current_smokeless_n ///

daily_tobacco_any daily_smoke_n daily_smokeless_n ///

smoke_only smokeless_only both_smoke_smokeless ///

alcohol_current no_fruit_veg no_fruit no_veg ///

atelessthan5 salt_eat_n salt_pack_n ///

htn_known htn_med file1 ///

using Table3_Weight_Age_Sex_W.xls, ///

c(col ci) f(1 1) percent svy replace

import delimited using Table3_Weight_Age_Sex_W.xls, clear

drop v6 v7

drop if v1 == "No" | v1 == "Total"

replace v1 = v1[_n - 1] if v1 == "Yes"

drop if v2 == ""

drop if v1 == ""

rename v2 IVR_Age_Sex

rename v4 STEPS_Age_Sex

rename v3 IVR_Age_Sex_CI

rename v5 STEPS_Age_Sex_CI

gen id = _n

save Table3_Weighted_Age_Sex_W.dta, replace

restore

preserve

use Table3_Unweighted.dta, replace

merge 1:1 v1 using Table3_Weighted_IPW, nogen

merge 1:1 v1 using Table3_Weighted_Age_Sex_W.dta, nogen

merge 1:1 v1 using Table3_n.dta, nogen

sort id

drop id

label var v1 "Indicator"

drop STEPS_IPW

gen indicator = _n

gen pop_group = 16

save Table3_16_Prevalence_GMAK_20Jul2022.dta, replace

restore

********************************************************************************

preserve

keep if sex_n == 1

keep if age_cat == 1

keep if school_n == 1

tabout current_tobacco_any current_smoke_n current_smokeless_n ///

daily_tobacco_any daily_smoke_n daily_smokeless_n ///

smoke_only smokeless_only both_smoke_smokeless ///

alcohol_current no_fruit_veg no_fruit no_veg ///

atelessthan5 salt_eat_n salt_pack_n ///

htn_known htn_med file1 ///

using Table3_n.xls, ///

c(freq col) f(1c 1p) replace

import delimited using Table3_n.xls, clear

drop if v1 == "No"

drop if v1 == "Yes"

replace v1 = v1[_n - 1] if v1 == "Total"

drop v3 v5 v6 v7

drop if v1 == ""

drop if v2 == ""

destring v2 v4, ignore(",") replace

rename v2 IVR_n

rename v4 STEPS_n

gen id = _n

save Table3_n.dta, replace

restore

preserve

keep if sex_n == 1

keep if age_cat == 1

keep if school_n == 1

gen wt = 1

svyset [pweight= wt]

tabout current_tobacco_any current_smoke_n current_smokeless_n ///

daily_tobacco_any daily_smoke_n daily_smokeless_n ///

smoke_only smokeless_only both_smoke_smokeless ///

alcohol_current no_fruit_veg no_fruit no_veg ///

atelessthan5 salt_eat_n salt_pack_n ///

htn_known htn_med file1 ///

using Table3_Unweight.xls, ///

c(col ci) f(1 1) percent svy replace

import delimited using Table3_Unweight.xls, clear

drop v6 v7

drop if v1 == "No" | v1 == "Total"

replace v1 = v1[_n - 1] if v1 == "Yes"

drop if v2 == ""

drop if v1 == ""

rename v2 IVR_Unweight

rename v4 STEPS_Unweight

rename v3 IVR_Unweight_CI

rename v5 STEPS_Unweight_CI

gen id = _n

save Table3_Unweighted.dta, replace

restore

********

preserve

keep if sex_n == 1

keep if age_cat == 1

keep if school_n == 1

svyset [pweight= weight_ipw]

tabout current_tobacco_any current_smoke_n current_smokeless_n ///

daily_tobacco_any daily_smoke_n daily_smokeless_n ///

smoke_only smokeless_only both_smoke_smokeless ///

alcohol_current no_fruit_veg no_fruit no_veg ///

atelessthan5 salt_eat_n salt_pack_n ///

htn_known htn_med file1 ///

using Table3_Weight_IPW.xls, ///

c(col ci) f(1 1) percent svy replace

import delimited using Table3_Weight_IPW.xls, clear

drop v6 v7

drop if v1 == "No" | v1 == "Total"

replace v1 = v1[_n - 1] if v1 == "Yes"

drop if v2 == ""

drop if v1 == ""

rename v2 IVR_IPW

rename v4 STEPS_IPW

rename v3 IVR_IPW_CI

rename v5 STEPS_IPW_CI

gen id = _n

save Table3_Weighted_IPW.dta, replace

restore

********

preserve

keep if sex_n == 1

keep if age_cat == 1

keep if school_n == 1

svyset [pweight= age_sex_w]

tabout current_tobacco_any current_smoke_n current_smokeless_n ///

daily_tobacco_any daily_smoke_n daily_smokeless_n ///

smoke_only smokeless_only both_smoke_smokeless ///

alcohol_current no_fruit_veg no_fruit no_veg ///

atelessthan5 salt_eat_n salt_pack_n ///

htn_known htn_med file1 ///

using Table3_Weight_Age_Sex_W.xls, ///

c(col ci) f(1 1) percent svy replace

import delimited using Table3_Weight_Age_Sex_W.xls, clear

drop v6 v7

drop if v1 == "No" | v1 == "Total"

replace v1 = v1[_n - 1] if v1 == "Yes"

drop if v2 == ""

drop if v1 == ""

rename v2 IVR_Age_Sex

rename v4 STEPS_Age_Sex

rename v3 IVR_Age_Sex_CI

rename v5 STEPS_Age_Sex_CI

gen id = _n

save Table3_Weighted_Age_Sex_W.dta, replace

restore

preserve

use Table3_Unweighted.dta, replace

merge 1:1 v1 using Table3_Weighted_IPW, nogen

merge 1:1 v1 using Table3_Weighted_Age_Sex_W.dta, nogen

merge 1:1 v1 using Table3_n.dta, nogen

sort id

drop id

label var v1 "Indicator"

drop STEPS_IPW

gen indicator = _n

gen pop_group = 17

save Table3_17_Prevalence_GMAK_20Jul2022.dta, replace

restore

********************************************************************************

preserve

keep if sex_n == 1

keep if age_cat == 1

keep if school_n == 2

tabout current_tobacco_any current_smoke_n current_smokeless_n ///

daily_tobacco_any daily_smoke_n daily_smokeless_n ///

smoke_only smokeless_only both_smoke_smokeless ///

alcohol_current no_fruit_veg no_fruit no_veg ///

atelessthan5 salt_eat_n salt_pack_n ///

htn_known htn_med file1 ///

using Table3_n.xls, ///

c(freq col) f(1c 1p) replace

import delimited using Table3_n.xls, clear

drop if v1 == "No"

drop if v1 == "Yes"

replace v1 = v1[_n - 1] if v1 == "Total"

drop v3 v5 v6 v7

drop if v1 == ""

drop if v2 == ""

destring v2 v4, ignore(",") replace

rename v2 IVR_n

rename v4 STEPS_n

gen id = _n

save Table3_n.dta, replace

restore

preserve

keep if sex_n == 1

keep if age_cat == 1

keep if school_n == 2

gen wt = 1

svyset [pweight= wt]

tabout current_tobacco_any current_smoke_n current_smokeless_n ///

daily_tobacco_any daily_smoke_n daily_smokeless_n ///

smoke_only smokeless_only both_smoke_smokeless ///

alcohol_current no_fruit_veg no_fruit no_veg ///

atelessthan5 salt_eat_n salt_pack_n ///

htn_known htn_med file1 ///

using Table3_Unweight.xls, ///

c(col ci) f(1 1) percent svy replace

import delimited using Table3_Unweight.xls, clear

drop v6 v7

drop if v1 == "No" | v1 == "Total"

replace v1 = v1[_n - 1] if v1 == "Yes"

drop if v2 == ""

drop if v1 == ""

rename v2 IVR_Unweight

rename v4 STEPS_Unweight

rename v3 IVR_Unweight_CI

rename v5 STEPS_Unweight_CI

gen id = _n

save Table3_Unweighted.dta, replace

restore

********

preserve

keep if sex_n == 1

keep if age_cat == 1

keep if school_n == 2

svyset [pweight= weight_ipw]

tabout current_tobacco_any current_smoke_n current_smokeless_n ///

daily_tobacco_any daily_smoke_n daily_smokeless_n ///

smoke_only smokeless_only both_smoke_smokeless ///

alcohol_current no_fruit_veg no_fruit no_veg ///

atelessthan5 salt_eat_n salt_pack_n ///

htn_known htn_med file1 ///

using Table3_Weight_IPW.xls, ///

c(col ci) f(1 1) percent svy replace

import delimited using Table3_Weight_IPW.xls, clear

drop v6 v7

drop if v1 == "No" | v1 == "Total"

replace v1 = v1[_n - 1] if v1 == "Yes"

drop if v2 == ""

drop if v1 == ""

rename v2 IVR_IPW

rename v4 STEPS_IPW

rename v3 IVR_IPW_CI

rename v5 STEPS_IPW_CI

gen id = _n

save Table3_Weighted_IPW.dta, replace

restore

********

preserve

keep if sex_n == 1

keep if age_cat == 1

keep if school_n == 2

svyset [pweight= age_sex_w]

tabout current_tobacco_any current_smoke_n current_smokeless_n ///

daily_tobacco_any daily_smoke_n daily_smokeless_n ///

smoke_only smokeless_only both_smoke_smokeless ///

alcohol_current no_fruit_veg no_fruit no_veg ///

atelessthan5 salt_eat_n salt_pack_n ///

htn_known htn_med file1 ///

using Table3_Weight_Age_Sex_W.xls, ///

c(col ci) f(1 1) percent svy replace

import delimited using Table3_Weight_Age_Sex_W.xls, clear

drop v6 v7

drop if v1 == "No" | v1 == "Total"

replace v1 = v1[_n - 1] if v1 == "Yes"

drop if v2 == ""

drop if v1 == ""

rename v2 IVR_Age_Sex

rename v4 STEPS_Age_Sex

rename v3 IVR_Age_Sex_CI

rename v5 STEPS_Age_Sex_CI

gen id = _n

save Table3_Weighted_Age_Sex_W.dta, replace

restore

preserve

use Table3_Unweighted.dta, replace

merge 1:1 v1 using Table3_Weighted_IPW, nogen

merge 1:1 v1 using Table3_Weighted_Age_Sex_W.dta, nogen

merge 1:1 v1 using Table3_n.dta, nogen

sort id

drop id

label var v1 "Indicator"

drop STEPS_IPW

gen indicator = _n

gen pop_group = 18

save Table3_18_Prevalence_GMAK_20Jul2022.dta, replace

restore

********************************************************************************

preserve

keep if sex_n == 1

keep if age_cat == 2

keep if school_n == 0

tabout current_tobacco_any current_smoke_n current_smokeless_n ///

daily_tobacco_any daily_smoke_n daily_smokeless_n ///

smoke_only smokeless_only both_smoke_smokeless ///

alcohol_current no_fruit_veg no_fruit no_veg ///

atelessthan5 salt_eat_n salt_pack_n ///

htn_known htn_med file1 ///

using Table3_n.xls, ///

c(freq col) f(1c 1p) replace

import delimited using Table3_n.xls, clear

drop if v1 == "No"

drop if v1 == "Yes"

replace v1 = v1[_n - 1] if v1 == "Total"

drop v3 v5 v6 v7

drop if v1 == ""

drop if v2 == ""

destring v2 v4, ignore(",") replace

rename v2 IVR_n

rename v4 STEPS_n

gen id = _n

save Table3_n.dta, replace

restore

preserve

keep if sex_n == 1

keep if age_cat == 2

keep if school_n == 0

gen wt = 1

svyset [pweight= wt]

tab no_fruit_veg file1, col

tabout current_tobacco_any current_smoke_n current_smokeless_n ///

daily_tobacco_any daily_smoke_n daily_smokeless_n ///

smoke_only smokeless_only both_smoke_smokeless ///

alcohol_current no_fruit_veg no_fruit no_veg ///

atelessthan5 salt_eat_n salt_pack_n ///

htn_known htn_med file1 ///

using Table3_Unweight.xls, ///

c(col ci) f(1 1) percent svy replace

import delimited using Table3_Unweight.xls, clear

drop v6 v7

drop if v1 == "No" | v1 == "Total"

replace v1 = v1[_n - 1] if v1 == "Yes"

drop if v2 == ""

drop if v1 == ""

rename v2 IVR_Unweight

rename v4 STEPS_Unweight

rename v3 IVR_Unweight_CI

rename v5 STEPS_Unweight_CI

gen id = _n

save Table3_Unweighted.dta, replace

restore

********

preserve

keep if sex_n == 1

keep if age_cat == 2

keep if school_n == 0

svyset [pweight= weight_ipw]

svy: tab no_fruit_veg file1, col

tabout current_tobacco_any current_smoke_n current_smokeless_n ///

daily_tobacco_any daily_smoke_n daily_smokeless_n ///

smoke_only smokeless_only both_smoke_smokeless ///

alcohol_current no_fruit_veg no_fruit no_veg ///

atelessthan5 salt_eat_n salt_pack_n ///

htn_known htn_med file1 ///

using Table3_Weight_IPW.xls, ///

c(col ci) f(1 1) percent svy replace

import delimited using Table3_Weight_IPW.xls, clear

drop v6 v7

drop if v1 == "No" | v1 == "Total"

replace v1 = v1[_n - 1] if v1 == "Yes"

drop if v2 == ""

drop if v1 == ""

rename v2 IVR_IPW

rename v4 STEPS_IPW

rename v3 IVR_IPW_CI

rename v5 STEPS_IPW_CI

gen id = _n

save Table3_Weighted_IPW.dta, replace

restore

********

preserve

keep if sex_n == 1

keep if age_cat == 2

keep if school_n == 0

svyset [pweight= age_sex_w]

svy: tab no_fruit_veg file1, col

tabout current_tobacco_any current_smoke_n current_smokeless_n ///

daily_tobacco_any daily_smoke_n daily_smokeless_n ///

smoke_only smokeless_only both_smoke_smokeless ///

alcohol_current no_fruit_veg no_fruit no_veg ///

atelessthan5 salt_eat_n salt_pack_n ///

htn_known htn_med file1 ///

using Table3_Weight_Age_Sex_W.xls, ///

c(col ci) f(1 1) percent svy replace

import delimited using Table3_Weight_Age_Sex_W.xls, clear

drop v6 v7

drop if v1 == "No" | v1 == "Total"

replace v1 = v1[_n - 1] if v1 == "Yes"

drop if v2 == ""

drop if v1 == ""

rename v2 IVR_Age_Sex

rename v4 STEPS_Age_Sex

rename v3 IVR_Age_Sex_CI

rename v5 STEPS_Age_Sex_CI

gen id = _n

save Table3_Weighted_Age_Sex_W.dta, replace

restore

preserve

use Table3_Unweighted.dta, replace

merge 1:1 v1 using Table3_Weighted_IPW, nogen

merge 1:1 v1 using Table3_Weighted_Age_Sex_W.dta, nogen

merge 1:1 v1 using Table3_n.dta, nogen

sort id

drop id

label var v1 "Indicator"

drop STEPS_IPW

gen indicator = _n

gen pop_group = 19

save Table3_19_Prevalence_GMAK_20Jul2022.dta, replace

restore

*******************************************************************************

preserve

keep if sex_n == 1

keep if age_cat == 2

keep if school_n == 1

tabout current_tobacco_any current_smoke_n current_smokeless_n ///

daily_tobacco_any daily_smoke_n daily_smokeless_n ///

smoke_only smokeless_only both_smoke_smokeless ///

alcohol_current no_fruit_veg no_fruit no_veg ///

atelessthan5 salt_eat_n salt_pack_n ///

htn_known htn_med file1 ///

using Table3_n.xls, ///

c(freq col) f(1c 1p) replace

import delimited using Table3_n.xls, clear

drop if v1 == "No"

drop if v1 == "Yes"

replace v1 = v1[_n - 1] if v1 == "Total"

drop v3 v5 v6 v7

drop if v1 == ""

drop if v2 == ""

destring v2 v4, ignore(",") replace

rename v2 IVR_n

rename v4 STEPS_n

gen id = _n

save Table3_n.dta, replace

restore

preserve

keep if sex_n == 1

keep if age_cat == 2

keep if school_n == 1

gen wt = 1

svyset [pweight= wt]

svy: tab no_fruit_veg file1, col

tabout current_tobacco_any current_smoke_n current_smokeless_n ///

daily_tobacco_any daily_smoke_n daily_smokeless_n ///

smoke_only smokeless_only both_smoke_smokeless ///

alcohol_current no_fruit_veg no_fruit no_veg ///

atelessthan5 salt_eat_n salt_pack_n ///

htn_known htn_med file1 ///

using Table3_Unweight.xls, ///

c(col ci) f(1 1) percent svy replace

import delimited using Table3_Unweight.xls, clear

drop v6 v7

drop if v1 == "No" | v1 == "Total"

replace v1 = v1[_n - 1] if v1 == "Yes"

drop if v2 == ""

drop if v1 == ""

rename v2 IVR_Unweight

rename v4 STEPS_Unweight

rename v3 IVR_Unweight_CI

rename v5 STEPS_Unweight_CI

gen id = _n

save Table3_Unweighted.dta, replace

restore

********

preserve

keep if sex_n == 1

keep if age_cat == 2

keep if school_n == 1

svyset [pweight= weight_ipw]

svy: tab no_fruit_veg file1, col

tabout current_tobacco_any current_smoke_n current_smokeless_n ///

daily_tobacco_any daily_smoke_n daily_smokeless_n ///

smoke_only smokeless_only both_smoke_smokeless ///

alcohol_current no_fruit_veg no_fruit no_veg ///

atelessthan5 salt_eat_n salt_pack_n ///

htn_known htn_med file1 ///

using Table3_Weight_IPW.xls, ///

c(col ci) f(1 1) percent svy replace

import delimited using Table3_Weight_IPW.xls, clear

drop v6 v7

drop if v1 == "No" | v1 == "Total"

replace v1 = v1[_n - 1] if v1 == "Yes"

drop if v2 == ""

drop if v1 == ""

rename v2 IVR_IPW

rename v4 STEPS_IPW

rename v3 IVR_IPW_CI

rename v5 STEPS_IPW_CI

gen id = _n

save Table3_Weighted_IPW.dta, replace

restore

********

preserve

keep if sex_n == 1

keep if age_cat == 2

keep if school_n == 1

svyset [pweight= age_sex_w]

svy: tab no_fruit_veg file1, col

tabout current_tobacco_any current_smoke_n current_smokeless_n ///

daily_tobacco_any daily_smoke_n daily_smokeless_n ///

smoke_only smokeless_only both_smoke_smokeless ///

alcohol_current no_fruit_veg no_fruit no_veg ///

atelessthan5 salt_eat_n salt_pack_n ///

htn_known htn_med file1 ///

using Table3_Weight_Age_Sex_W.xls, ///

c(col ci) f(1 1) percent svy replace

import delimited using Table3_Weight_Age_Sex_W.xls, clear

drop v6 v7

drop if v1 == "No" | v1 == "Total"

replace v1 = v1[_n - 1] if v1 == "Yes"

drop if v2 == ""

drop if v1 == ""

rename v2 IVR_Age_Sex

rename v4 STEPS_Age_Sex

rename v3 IVR_Age_Sex_CI

rename v5 STEPS_Age_Sex_CI

gen id = _n

save Table3_Weighted_Age_Sex_W.dta, replace

restore

preserve

use Table3_Unweighted.dta, replace

merge 1:1 v1 using Table3_Weighted_IPW, nogen

merge 1:1 v1 using Table3_Weighted_Age_Sex_W.dta, nogen

merge 1:1 v1 using Table3_n.dta, nogen

sort id

drop id

label var v1 "Indicator"

drop STEPS_IPW

gen indicator = _n

gen pop_group = 20

save Table3_20_Prevalence_GMAK_20Jul2022.dta, replace

restore

cap erase Table3_Unweighted.dta

cap erase Table3_Weighted_IPW.dta

cap erase Table3_Weighted_Age_Sex_W.dta

cap erase Table3_Weight_Age_Sex_W.xls

cap erase Table3_Weight_IPW.xls

cap erase Table3_Unweight.xls

*******************************************************************************

preserve

keep if sex_n == 1

keep if age_cat == 2

keep if school_n == 2

tabout current_tobacco_any current_smoke_n current_smokeless_n ///

daily_tobacco_any daily_smoke_n daily_smokeless_n ///

smoke_only smokeless_only both_smoke_smokeless ///

alcohol_current no_fruit_veg no_fruit no_veg ///

atelessthan5 salt_eat_n salt_pack_n ///

htn_known htn_med file1 ///

using Table3_n.xls, ///

c(freq col) f(1c 1p) replace

import delimited using Table3_n.xls, clear

drop if v1 == "No"

drop if v1 == "Yes"

replace v1 = v1[_n - 1] if v1 == "Total"

drop v3 v5 v6 v7

drop if v1 == ""

drop if v2 == ""

destring v2 v4, ignore(",") replace

rename v2 IVR_n

rename v4 STEPS_n

gen id = _n

save Table3_n.dta, replace

restore

preserve

keep if sex_n == 1

keep if age_cat == 2

keep if school_n == 2

gen wt = 1

svyset [pweight= wt]

tabout current_tobacco_any current_smoke_n current_smokeless_n ///

daily_tobacco_any daily_smoke_n daily_smokeless_n ///

smoke_only smokeless_only both_smoke_smokeless ///

alcohol_current no_fruit_veg no_fruit no_veg ///

atelessthan5 salt_eat_n salt_pack_n ///

htn_known htn_med file1 ///

using Table3_Unweight.xls, ///

c(col ci) f(1 1) percent svy replace

import delimited using Table3_Unweight.xls, clear

drop v6 v7

drop if v1 == "No" | v1 == "Total"

replace v1 = v1[_n - 1] if v1 == "Yes"

drop if v2 == ""

drop if v1 == ""

rename v2 IVR_Unweight

rename v4 STEPS_Unweight

rename v3 IVR_Unweight_CI

rename v5 STEPS_Unweight_CI

gen id = _n

save Table3_Unweighted.dta, replace

restore

********

preserve

keep if sex_n == 1

keep if age_cat == 2

keep if school_n == 2

svyset [pweight= weight_ipw]

tabout current_tobacco_any current_smoke_n current_smokeless_n ///

daily_tobacco_any daily_smoke_n daily_smokeless_n ///

smoke_only smokeless_only both_smoke_smokeless ///

alcohol_current no_fruit_veg no_fruit no_veg ///

atelessthan5 salt_eat_n salt_pack_n ///

htn_known htn_med file1 ///

using Table3_Weight_IPW.xls, ///

c(col ci) f(1 1) percent svy replace

import delimited using Table3_Weight_IPW.xls, clear

drop v6 v7

drop if v1 == "No" | v1 == "Total"

replace v1 = v1[_n - 1] if v1 == "Yes"

drop if v2 == ""

drop if v1 == ""

rename v2 IVR_IPW

rename v4 STEPS_IPW

rename v3 IVR_IPW_CI

rename v5 STEPS_IPW_CI

gen id = _n

save Table3_Weighted_IPW.dta, replace

restore

********

preserve

keep if sex_n == 1

keep if age_cat == 2

keep if school_n == 2

svyset [pweight= age_sex_w]

tabout current_tobacco_any current_smoke_n current_smokeless_n ///

daily_tobacco_any daily_smoke_n daily_smokeless_n ///

smoke_only smokeless_only both_smoke_smokeless ///

alcohol_current no_fruit_veg no_fruit no_veg ///

atelessthan5 salt_eat_n salt_pack_n ///

htn_known htn_med file1 ///

using Table3_Weight_Age_Sex_W.xls, ///

c(col ci) f(1 1) percent svy replace

import delimited using Table3_Weight_Age_Sex_W.xls, clear

drop v6 v7

drop if v1 == "No" | v1 == "Total"

replace v1 = v1[_n - 1] if v1 == "Yes"

drop if v2 == ""

drop if v1 == ""

rename v2 IVR_Age_Sex

rename v4 STEPS_Age_Sex

rename v3 IVR_Age_Sex_CI

rename v5 STEPS_Age_Sex_CI

gen id = _n

save Table3_Weighted_Age_Sex_W.dta, replace

restore

preserve

use Table3_Unweighted.dta, replace

merge 1:1 v1 using Table3_Weighted_IPW, nogen

merge 1:1 v1 using Table3_Weighted_Age_Sex_W.dta, nogen

merge 1:1 v1 using Table3_n.dta, nogen

sort id

drop id

label var v1 "Indicator"

drop STEPS_IPW

gen indicator = _n

gen pop_group = 21

save Table3_21_Prevalence_GMAK_20Jul2022.dta, replace

restore

*******************************************************************************

preserve

keep if sex_n == 1

keep if school_n == 0

tabout current_tobacco_any current_smoke_n current_smokeless_n ///

daily_tobacco_any daily_smoke_n daily_smokeless_n ///

smoke_only smokeless_only both_smoke_smokeless ///

alcohol_current no_fruit_veg no_fruit no_veg ///

atelessthan5 salt_eat_n salt_pack_n ///

htn_known htn_med file1 ///

using Table3_n.xls, ///

c(freq col) f(1c 1p) replace

import delimited using Table3_n.xls, clear

drop if v1 == "No"

drop if v1 == "Yes"

replace v1 = v1[_n - 1] if v1 == "Total"

drop v3 v5 v6 v7

drop if v1 == ""

drop if v2 == ""

destring v2 v4, ignore(",") replace

rename v2 IVR_n

rename v4 STEPS_n

gen id = _n

save Table3_n.dta, replace

restore

preserve

keep if sex_n == 1

keep if school_n == 0

gen wt = 1

svyset [pweight= wt]

tabout current_tobacco_any current_smoke_n current_smokeless_n ///

daily_tobacco_any daily_smoke_n daily_smokeless_n ///

smoke_only smokeless_only both_smoke_smokeless ///

alcohol_current no_fruit_veg no_fruit no_veg ///

atelessthan5 salt_eat_n salt_pack_n ///

htn_known htn_med file1 ///

using Table3_Unweight.xls, ///

c(col ci) f(1 1) percent svy replace

import delimited using Table3_Unweight.xls, clear

drop v6 v7

drop if v1 == "No" | v1 == "Total"

replace v1 = v1[_n - 1] if v1 == "Yes"

drop if v2 == ""

drop if v1 == ""

rename v2 IVR_Unweight

rename v4 STEPS_Unweight

rename v3 IVR_Unweight_CI

rename v5 STEPS_Unweight_CI

gen id = _n

save Table3_Unweighted.dta, replace

restore

********

preserve

keep if sex_n == 1

keep if school_n == 0

svyset [pweight= weight_ipw]

tabout current_tobacco_any current_smoke_n current_smokeless_n ///

daily_tobacco_any daily_smoke_n daily_smokeless_n ///

smoke_only smokeless_only both_smoke_smokeless ///

alcohol_current no_fruit_veg no_fruit no_veg ///

atelessthan5 salt_eat_n salt_pack_n ///

htn_known htn_med file1 ///

using Table3_Weight_IPW.xls, ///

c(col ci) f(1 1) percent svy replace

import delimited using Table3_Weight_IPW.xls, clear

drop v6 v7

drop if v1 == "No" | v1 == "Total"

replace v1 = v1[_n - 1] if v1 == "Yes"

drop if v2 == ""

drop if v1 == ""

rename v2 IVR_IPW

rename v4 STEPS_IPW

rename v3 IVR_IPW_CI

rename v5 STEPS_IPW_CI

gen id = _n

save Table3_Weighted_IPW.dta, replace

restore

********

preserve

keep if sex_n == 1

keep if school_n == 0

svyset [pweight= age_sex_w]

tabout current_tobacco_any current_smoke_n current_smokeless_n ///

daily_tobacco_any daily_smoke_n daily_smokeless_n ///

smoke_only smokeless_only both_smoke_smokeless ///

alcohol_current no_fruit_veg no_fruit no_veg ///

atelessthan5 salt_eat_n salt_pack_n ///

htn_known htn_med file1 ///

using Table3_Weight_Age_Sex_W.xls, ///

c(col ci) f(1 1) percent svy replace

import delimited using Table3_Weight_Age_Sex_W.xls, clear

drop v6 v7

drop if v1 == "No" | v1 == "Total"

replace v1 = v1[_n - 1] if v1 == "Yes"

drop if v2 == ""

drop if v1 == ""

rename v2 IVR_Age_Sex

rename v4 STEPS_Age_Sex

rename v3 IVR_Age_Sex_CI

rename v5 STEPS_Age_Sex_CI

gen id = _n

save Table3_Weighted_Age_Sex_W.dta, replace

restore

preserve

use Table3_Unweighted.dta, replace

merge 1:1 v1 using Table3_Weighted_IPW, nogen

merge 1:1 v1 using Table3_Weighted_Age_Sex_W.dta, nogen

merge 1:1 v1 using Table3_n.dta, nogen

sort id

drop id

label var v1 "Indicator"

drop STEPS_IPW

gen indicator = _n

gen pop_group = 22

save Table3_22_Prevalence_GMAK_20Jul2022.dta, replace

restore

cap erase Table3_Unweighted.dta

cap erase Table3_Weighted_IPW.dta

cap erase Table3_Weighted_Age_Sex_W.dta

cap erase Table3_Weight_Age_Sex_W.xls

cap erase Table3_Weight_IPW.xls

cap erase Table3_Unweight.xls

*******************************************************************************

preserve

keep if sex_n == 1

keep if school_n == 1

tabout current_tobacco_any current_smoke_n current_smokeless_n ///

daily_tobacco_any daily_smoke_n daily_smokeless_n ///

smoke_only smokeless_only both_smoke_smokeless ///

alcohol_current no_fruit_veg no_fruit no_veg ///

atelessthan5 salt_eat_n salt_pack_n ///

htn_known htn_med file1 ///

using Table3_n.xls, ///

c(freq col) f(1c 1p) replace

import delimited using Table3_n.xls, clear

drop if v1 == "No"

drop if v1 == "Yes"

replace v1 = v1[_n - 1] if v1 == "Total"

drop v3 v5 v6 v7

drop if v1 == ""

drop if v2 == ""

destring v2 v4, ignore(",") replace

rename v2 IVR_n

rename v4 STEPS_n

gen id = _n

save Table3_n.dta, replace

restore

preserve

keep if sex_n == 1

keep if school_n == 1

gen wt = 1

svyset [pweight= wt]

tabout current_tobacco_any current_smoke_n current_smokeless_n ///

daily_tobacco_any daily_smoke_n daily_smokeless_n ///

smoke_only smokeless_only both_smoke_smokeless ///

alcohol_current no_fruit_veg no_fruit no_veg ///

atelessthan5 salt_eat_n salt_pack_n ///

htn_known htn_med file1 ///

using Table3_Unweight.xls, ///

c(col ci) f(1 1) percent svy replace

import delimited using Table3_Unweight.xls, clear

drop v6 v7

drop if v1 == "No" | v1 == "Total"

replace v1 = v1[_n - 1] if v1 == "Yes"

drop if v2 == ""

drop if v1 == ""

rename v2 IVR_Unweight

rename v4 STEPS_Unweight

rename v3 IVR_Unweight_CI

rename v5 STEPS_Unweight_CI

gen id = _n

save Table3_Unweighted.dta, replace

restore

********

preserve

keep if sex_n == 1

keep if school_n == 1

svyset [pweight= weight_ipw]

tabout current_tobacco_any current_smoke_n current_smokeless_n ///

daily_tobacco_any daily_smoke_n daily_smokeless_n ///

smoke_only smokeless_only both_smoke_smokeless ///

alcohol_current no_fruit_veg no_fruit no_veg ///

atelessthan5 salt_eat_n salt_pack_n ///

htn_known htn_med file1 ///

using Table3_Weight_IPW.xls, ///

c(col ci) f(1 1) percent svy replace

import delimited using Table3_Weight_IPW.xls, clear

drop v6 v7

drop if v1 == "No" | v1 == "Total"

replace v1 = v1[_n - 1] if v1 == "Yes"

drop if v2 == ""

drop if v1 == ""

rename v2 IVR_IPW

rename v4 STEPS_IPW

rename v3 IVR_IPW_CI

rename v5 STEPS_IPW_CI

gen id = _n

save Table3_Weighted_IPW.dta, replace

restore

********

preserve

keep if sex_n == 1

keep if school_n == 1

svyset [pweight= age_sex_w]

tabout current_tobacco_any current_smoke_n current_smokeless_n ///

daily_tobacco_any daily_smoke_n daily_smokeless_n ///

smoke_only smokeless_only both_smoke_smokeless ///

alcohol_current no_fruit_veg no_fruit no_veg ///

atelessthan5 salt_eat_n salt_pack_n ///

htn_known htn_med file1 ///

using Table3_Weight_Age_Sex_W.xls, ///

c(col ci) f(1 1) percent svy replace

import delimited using Table3_Weight_Age_Sex_W.xls, clear

drop v6 v7

drop if v1 == "No" | v1 == "Total"

replace v1 = v1[_n - 1] if v1 == "Yes"

drop if v2 == ""

drop if v1 == ""

rename v2 IVR_Age_Sex

rename v4 STEPS_Age_Sex

rename v3 IVR_Age_Sex_CI

rename v5 STEPS_Age_Sex_CI

gen id = _n

save Table3_Weighted_Age_Sex_W.dta, replace

restore

preserve

use Table3_Unweighted.dta, replace

merge 1:1 v1 using Table3_Weighted_IPW, nogen

merge 1:1 v1 using Table3_Weighted_Age_Sex_W.dta, nogen

merge 1:1 v1 using Table3_n.dta, nogen

sort id

drop id

label var v1 "Indicator"

drop STEPS_IPW

gen indicator = _n

gen pop_group = 23

save Table3_23_Prevalence_GMAK_20Jul2022.dta, replace

restore

cap erase Table3_Unweighted.dta

cap erase Table3_Weighted_IPW.dta

cap erase Table3_Weighted_Age_Sex_W.dta

cap erase Table3_Weight_Age_Sex_W.xls

cap erase Table3_Weight_IPW.xls

cap erase Table3_Unweight.xls

*******************************************************************************

preserve

keep if sex_n == 1

keep if school_n == 2

tabout current_tobacco_any current_smoke_n current_smokeless_n ///

daily_tobacco_any daily_smoke_n daily_smokeless_n ///

smoke_only smokeless_only both_smoke_smokeless ///

alcohol_current no_fruit_veg no_fruit no_veg ///

atelessthan5 salt_eat_n salt_pack_n ///

htn_known htn_med file1 ///

using Table3_n.xls, ///

c(freq col) f(1c 1p) replace

import delimited using Table3_n.xls, clear

drop if v1 == "No"

drop if v1 == "Yes"

replace v1 = v1[_n - 1] if v1 == "Total"

drop v3 v5 v6 v7

drop if v1 == ""

drop if v2 == ""

destring v2 v4, ignore(",") replace

rename v2 IVR_n

rename v4 STEPS_n

gen id = _n

save Table3_n.dta, replace

restore

preserve

keep if sex_n == 1

keep if school_n == 2

gen wt = 1

svyset [pweight= wt]

tabout current_tobacco_any current_smoke_n current_smokeless_n ///

daily_tobacco_any daily_smoke_n daily_smokeless_n ///

smoke_only smokeless_only both_smoke_smokeless ///

alcohol_current no_fruit_veg no_fruit no_veg ///

atelessthan5 salt_eat_n salt_pack_n ///

htn_known htn_med file1 ///

using Table3_Unweight.xls, ///

c(col ci) f(1 1) percent svy replace

import delimited using Table3_Unweight.xls, clear

drop v6 v7

drop if v1 == "No" | v1 == "Total"

replace v1 = v1[_n - 1] if v1 == "Yes"

drop if v2 == ""

drop if v1 == ""

rename v2 IVR_Unweight

rename v4 STEPS_Unweight

rename v3 IVR_Unweight_CI

rename v5 STEPS_Unweight_CI

gen id = _n

save Table3_Unweighted.dta, replace

restore

********

preserve

keep if sex_n == 1

keep if school_n == 2

svyset [pweight= weight_ipw]

tabout current_tobacco_any current_smoke_n current_smokeless_n ///

daily_tobacco_any daily_smoke_n daily_smokeless_n ///

smoke_only smokeless_only both_smoke_smokeless ///

alcohol_current no_fruit_veg no_fruit no_veg ///

atelessthan5 salt_eat_n salt_pack_n ///

htn_known htn_med file1 ///

using Table3_Weight_IPW.xls, ///

c(col ci) f(1 1) percent svy replace

import delimited using Table3_Weight_IPW.xls, clear

drop v6 v7

drop if v1 == "No" | v1 == "Total"

replace v1 = v1[_n - 1] if v1 == "Yes"

drop if v2 == ""

drop if v1 == ""

rename v2 IVR_IPW

rename v4 STEPS_IPW

rename v3 IVR_IPW_CI

rename v5 STEPS_IPW_CI

gen id = _n

save Table3_Weighted_IPW.dta, replace

restore

********

preserve

keep if sex_n == 1

keep if school_n == 2

svyset [pweight= age_sex_w]

tabout current_tobacco_any current_smoke_n current_smokeless_n ///

daily_tobacco_any daily_smoke_n daily_smokeless_n ///

smoke_only smokeless_only both_smoke_smokeless ///

alcohol_current no_fruit_veg no_fruit no_veg ///

atelessthan5 salt_eat_n salt_pack_n ///

htn_known htn_med file1 ///

using Table3_Weight_Age_Sex_W.xls, ///

c(col ci) f(1 1) percent svy replace

import delimited using Table3_Weight_Age_Sex_W.xls, clear

drop v6 v7

drop if v1 == "No" | v1 == "Total"

replace v1 = v1[_n - 1] if v1 == "Yes"

drop if v2 == ""

drop if v1 == ""

rename v2 IVR_Age_Sex

rename v4 STEPS_Age_Sex

rename v3 IVR_Age_Sex_CI

rename v5 STEPS_Age_Sex_CI

gen id = _n

save Table3_Weighted_Age_Sex_W.dta, replace

restore

preserve

use Table3_Unweighted.dta, replace

merge 1:1 v1 using Table3_Weighted_IPW, nogen

merge 1:1 v1 using Table3_Weighted_Age_Sex_W.dta, nogen

merge 1:1 v1 using Table3_n.dta, nogen

sort id

drop id

label var v1 "Indicator"

drop STEPS_IPW

gen indicator = _n

gen pop_group = 24

save Table3_24_Prevalence_GMAK_20Jul2022.dta, replace

restore

cap erase Table3_Unweighted.dta

cap erase Table3_Weighted_IPW.dta

cap erase Table3_Weighted_Age_Sex_W.dta

cap erase Table3_Weight_Age_Sex_W.xls

cap erase Table3_Weight_IPW.xls

cap erase Table3_Unweight.xls

*******************************************************************************

preserve

keep if sex_n == 2

keep if age_cat == 0

keep if school_n == 0

tabout current_tobacco_any current_smoke_n current_smokeless_n ///

daily_tobacco_any daily_smoke_n daily_smokeless_n ///

smoke_only smokeless_only both_smoke_smokeless ///

alcohol_current no_fruit_veg no_fruit no_veg ///

atelessthan5 salt_eat_n salt_pack_n ///

htn_known htn_med file1 ///

using Table3_n.xls, ///

c(freq col) f(1c 1p) replace

import delimited using Table3_n.xls, clear

drop if v1 == "No"

drop if v1 == "Yes"

replace v1 = v1[_n - 1] if v1 == "Total"

drop v3 v5 v6 v7

drop if v1 == ""

drop if v2 == ""

destring v2 v4, ignore(",") replace

rename v2 IVR_n

rename v4 STEPS_n

gen id = _n

save Table3_n.dta, replace

restore

preserve

keep if sex_n == 2

keep if age_cat == 0

keep if school_n == 0

gen wt = 1

svyset [pweight= wt]

tabout current_tobacco_any current_smoke_n current_smokeless_n ///

daily_tobacco_any daily_smoke_n daily_smokeless_n ///

smoke_only smokeless_only both_smoke_smokeless ///

alcohol_current no_fruit_veg no_fruit no_veg ///

atelessthan5 salt_eat_n salt_pack_n ///

htn_known htn_med file1 ///

using Table3_Unweight.xls, ///

c(col ci) f(1 1) percent svy replace

import delimited using Table3_Unweight.xls, clear

drop v6 v7

drop if v1 == "No" | v1 == "Total"

replace v1 = v1[_n - 1] if v1 == "Yes"

drop if v2 == ""

drop if v1 == ""

rename v2 IVR_Unweight

rename v4 STEPS_Unweight

rename v3 IVR_Unweight_CI

rename v5 STEPS_Unweight_CI

gen id = _n

save Table3_Unweighted.dta, replace

restore

********

preserve

keep if sex_n == 2

keep if age_cat == 0

keep if school_n == 0

svyset [pweight= weight_ipw]

tabout current_tobacco_any current_smoke_n current_smokeless_n ///

daily_tobacco_any daily_smoke_n daily_smokeless_n ///

smoke_only smokeless_only both_smoke_smokeless ///

alcohol_current no_fruit_veg no_fruit no_veg ///

atelessthan5 salt_eat_n salt_pack_n ///

htn_known htn_med file1 ///

using Table3_Weight_IPW.xls, ///

c(col ci) f(1 1) percent svy replace

import delimited using Table3_Weight_IPW.xls, clear

drop v6 v7

drop if v1 == "No" | v1 == "Total"

replace v1 = v1[_n - 1] if v1 == "Yes"

drop if v2 == ""

drop if v1 == ""

rename v2 IVR_IPW

rename v4 STEPS_IPW

rename v3 IVR_IPW_CI

rename v5 STEPS_IPW_CI

gen id = _n

save Table3_Weighted_IPW.dta, replace

restore

********

preserve

keep if sex_n == 2

keep if age_cat == 0

keep if school_n == 0

svyset [pweight= age_sex_w]

tabout current_tobacco_any current_smoke_n current_smokeless_n ///

daily_tobacco_any daily_smoke_n daily_smokeless_n ///

smoke_only smokeless_only both_smoke_smokeless ///

alcohol_current no_fruit_veg no_fruit no_veg ///

atelessthan5 salt_eat_n salt_pack_n ///

htn_known htn_med file1 ///

using Table3_Weight_Age_Sex_W.xls, ///

c(col ci) f(1 1) percent svy replace

import delimited using Table3_Weight_Age_Sex_W.xls, clear

drop v6 v7

drop if v1 == "No" | v1 == "Total"

replace v1 = v1[_n - 1] if v1 == "Yes"

drop if v2 == ""

drop if v1 == ""

rename v2 IVR_Age_Sex

rename v4 STEPS_Age_Sex

rename v3 IVR_Age_Sex_CI

rename v5 STEPS_Age_Sex_CI

gen id = _n

save Table3_Weighted_Age_Sex_W.dta, replace

restore

preserve

use Table3_Unweighted.dta, replace

merge 1:1 v1 using Table3_Weighted_IPW, nogen

merge 1:1 v1 using Table3_Weighted_Age_Sex_W.dta, nogen

merge 1:1 v1 using Table3_n.dta, nogen

sort id

drop id

label var v1 "Indicator"

drop STEPS_IPW

gen indicator = _n

gen pop_group = 25

save Table3_25_Prevalence_GMAK_20Jul2022.dta, replace

restore

cap erase Table3_Unweighted.dta

cap erase Table3_Weighted_IPW.dta

cap erase Table3_Weighted_Age_Sex_W.dta

cap erase Table3_Weight_Age_Sex_W.xls

cap erase Table3_Weight_IPW.xls

cap erase Table3_Unweight.xls

*******************************************************************************

preserve

keep if sex_n == 2

keep if age_cat == 0

keep if school_n == 1

tabout current_tobacco_any current_smoke_n current_smokeless_n ///

daily_tobacco_any daily_smoke_n daily_smokeless_n ///

smoke_only smokeless_only both_smoke_smokeless ///

alcohol_current no_fruit_veg no_fruit no_veg ///

atelessthan5 salt_eat_n salt_pack_n ///

htn_known htn_med file1 ///

using Table3_n.xls, ///

c(freq col) f(1c 1p) replace

import delimited using Table3_n.xls, clear

drop if v1 == "No"

drop if v1 == "Yes"

replace v1 = v1[_n - 1] if v1 == "Total"

drop v3 v5 v6 v7

drop if v1 == ""

drop if v2 == ""

destring v2 v4, ignore(",") replace

rename v2 IVR_n

rename v4 STEPS_n

gen id = _n

save Table3_n.dta, replace

restore

preserve

keep if sex_n == 2

keep if age_cat == 0

keep if school_n == 1

gen wt = 1

svyset [pweight= wt]

for var current_tobacco_any current_smoke_n current_smokeless_n ///

daily_tobacco_any daily_smoke_n daily_smokeless_n ///

smoke_only smokeless_only both_smoke_smokeless ///

alcohol_current no_fruit_veg no_fruit no_veg ///

atelessthan5 salt_eat_n salt_pack_n ///

htn_known htn_med: tab X file1

tabout current_tobacco_any current_smoke_n current_smokeless_n ///

daily_tobacco_any daily_smoke_n daily_smokeless_n ///

smoke_only smokeless_only ///

no_fruit_veg no_fruit no_veg ///

atelessthan5 salt_eat_n salt_pack_n ///

htn_known htn_med file1 ///

using Table3_Unweight.xls, ///

c(col ci) f(1 1) percent svy replace

import delimited using Table3_Unweight.xls, clear

drop v6 v7

drop if v1 == "No" | v1 == "Total"

replace v1 = v1[_n - 1] if v1 == "Yes"

drop if v2 == ""

drop if v1 == ""

rename v2 IVR_Unweight

rename v4 STEPS_Unweight

rename v3 IVR_Unweight_CI

rename v5 STEPS_Unweight_CI

gen id = _n

save Table3_Unweighted.dta, replace

restore

********

preserve

keep if sex_n == 2

keep if age_cat == 0

keep if school_n == 1

svyset [pweight= weight_ipw]

tabout current_tobacco_any current_smoke_n current_smokeless_n ///

daily_tobacco_any daily_smoke_n daily_smokeless_n ///

smoke_only smokeless_only ///

no_fruit_veg no_fruit no_veg ///

atelessthan5 salt_eat_n salt_pack_n ///

htn_known htn_med file1 ///

using Table3_Weight_IPW.xls, ///

c(col ci) f(1 1) percent svy replace

import delimited using Table3_Weight_IPW.xls, clear

drop v6 v7

drop if v1 == "No" | v1 == "Total"

replace v1 = v1[_n - 1] if v1 == "Yes"

drop if v2 == ""

drop if v1 == ""

rename v2 IVR_IPW

rename v4 STEPS_IPW

rename v3 IVR_IPW_CI

rename v5 STEPS_IPW_CI

gen id = _n

save Table3_Weighted_IPW.dta, replace

restore

********

preserve

keep if sex_n == 2

keep if age_cat == 0

keep if school_n == 1

svyset [pweight= age_sex_w]

tabout current_tobacco_any current_smoke_n current_smokeless_n ///

daily_tobacco_any daily_smoke_n daily_smokeless_n ///

smoke_only smokeless_only ///

no_fruit_veg no_fruit no_veg ///

atelessthan5 salt_eat_n salt_pack_n ///

htn_known htn_med file1 ///

using Table3_Weight_Age_Sex_W.xls, ///

c(col ci) f(1 1) percent svy replace

import delimited using Table3_Weight_Age_Sex_W.xls, clear

drop v6 v7

drop if v1 == "No" | v1 == "Total"

replace v1 = v1[_n - 1] if v1 == "Yes"

drop if v2 == ""

drop if v1 == ""

rename v2 IVR_Age_Sex

rename v4 STEPS_Age_Sex

rename v3 IVR_Age_Sex_CI

rename v5 STEPS_Age_Sex_CI

gen id = _n

save Table3_Weighted_Age_Sex_W.dta, replace

restore

preserve

use Table3_Unweighted.dta, replace

merge 1:1 v1 using Table3_Weighted_IPW, nogen

merge 1:1 v1 using Table3_Weighted_Age_Sex_W.dta, nogen

merge 1:1 v1 using Table3_n.dta, nogen

sort id

drop id

label var v1 "Indicator"

drop STEPS_IPW

gen indicator = _n

gen pop_group = 26

save Table3_26_Prevalence_GMAK_20Jul2022.dta, replace

restore

cap erase Table3_Unweighted.dta

cap erase Table3_Weighted_IPW.dta

cap erase Table3_Weighted_Age_Sex_W.dta

cap erase Table3_Weight_Age_Sex_W.xls

cap erase Table3_Weight_IPW.xls

cap erase Table3_Unweight.xls

*******************************************************************************

preserve

keep if sex_n == 2

keep if age_cat == 0

keep if school_n == 2

tabout current_tobacco_any current_smoke_n current_smokeless_n ///

daily_tobacco_any daily_smoke_n daily_smokeless_n ///

smoke_only smokeless_only both_smoke_smokeless ///

alcohol_current no_fruit_veg no_fruit no_veg ///

atelessthan5 salt_eat_n salt_pack_n ///

htn_known htn_med file1 ///

using Table3_n.xls, ///

c(freq col) f(1c 1p) replace

import delimited using Table3_n.xls, clear

drop if v1 == "No"

drop if v1 == "Yes"

replace v1 = v1[_n - 1] if v1 == "Total"

drop v3 v5 v6 v7

drop if v1 == ""

drop if v2 == ""

destring v2 v4, ignore(",") replace

rename v2 IVR_n

rename v4 STEPS_n

gen id = _n

save Table3_n.dta, replace

restore

preserve

keep if sex_n == 2

keep if age_cat == 0

keep if school_n == 2

gen wt = 1

svyset [pweight= wt]

for var current_tobacco_any current_smoke_n current_smokeless_n ///

daily_tobacco_any daily_smoke_n daily_smokeless_n ///

smoke_only smokeless_only both_smoke_smokeless ///

alcohol_current no_fruit_veg no_fruit no_veg ///

atelessthan5 salt_eat_n salt_pack_n ///

htn_known htn_med: tab X file1

tabout current_tobacco_any current_smoke_n current_smokeless_n ///

daily_tobacco_any daily_smokeless_n ///

smoke_only smokeless_only no_fruit_veg no_fruit no_veg ///

atelessthan5 salt_eat_n salt_pack_n ///

htn_known htn_med file1 ///

using Table3_Unweight.xls, ///

c(col ci) f(1 1) percent svy replace

import delimited using Table3_Unweight.xls, clear

drop v6 v7

drop if v1 == "No" | v1 == "Total"

replace v1 = v1[_n - 1] if v1 == "Yes"

drop if v2 == ""

drop if v1 == ""

rename v2 IVR_Unweight

rename v4 STEPS_Unweight

rename v3 IVR_Unweight_CI

rename v5 STEPS_Unweight_CI

gen id = _n

save Table3_Unweighted.dta, replace

restore

********

preserve

keep if sex_n == 2

keep if age_cat == 0

keep if school_n == 2

svyset [pweight= weight_ipw]

tabout current_tobacco_any current_smoke_n current_smokeless_n ///

daily_tobacco_any daily_smokeless_n ///

smoke_only smokeless_only no_fruit_veg no_fruit no_veg ///

atelessthan5 salt_eat_n salt_pack_n ///

htn_known htn_med file1 ///

using Table3_Weight_IPW.xls, ///

c(col ci) f(1 1) percent svy replace

import delimited using Table3_Weight_IPW.xls, clear

drop v6 v7

drop if v1 == "No" | v1 == "Total"

replace v1 = v1[_n - 1] if v1 == "Yes"

drop if v2 == ""

drop if v1 == ""

rename v2 IVR_IPW

rename v4 STEPS_IPW

rename v3 IVR_IPW_CI

rename v5 STEPS_IPW_CI

gen id = _n

save Table3_Weighted_IPW.dta, replace

restore

********

preserve

keep if sex_n == 2

keep if age_cat == 0

keep if school_n == 2

svyset [pweight= age_sex_w]

tabout current_tobacco_any current_smoke_n current_smokeless_n ///

daily_tobacco_any daily_smokeless_n ///

smoke_only smokeless_only no_fruit_veg no_fruit no_veg ///

atelessthan5 salt_eat_n salt_pack_n ///

htn_known htn_med file1 ///

using Table3_Weight_Age_Sex_W.xls, ///

c(col ci) f(1 1) percent svy replace

import delimited using Table3_Weight_Age_Sex_W.xls, clear

drop v6 v7

drop if v1 == "No" | v1 == "Total"

replace v1 = v1[_n - 1] if v1 == "Yes"

drop if v2 == ""

drop if v1 == ""

rename v2 IVR_Age_Sex

rename v4 STEPS_Age_Sex

rename v3 IVR_Age_Sex_CI

rename v5 STEPS_Age_Sex_CI

gen id = _n

save Table3_Weighted_Age_Sex_W.dta, replace

restore

preserve

use Table3_Unweighted.dta, replace

merge 1:1 v1 using Table3_Weighted_IPW, nogen

merge 1:1 v1 using Table3_Weighted_Age_Sex_W.dta, nogen

merge 1:1 v1 using Table3_n.dta, nogen

sort id

drop id

label var v1 "Indicator"

drop STEPS_IPW

gen indicator = _n

gen pop_group = 27

save Table3_27_Prevalence_GMAK_20Jul2022.dta, replace

restore

cap erase Table3_Unweighted.dta

cap erase Table3_Weighted_IPW.dta

cap erase Table3_Weighted_Age_Sex_W.dta

cap erase Table3_Weight_Age_Sex_W.xls

cap erase Table3_Weight_IPW.xls

cap erase Table3_Unweight.xls

*******************************************************************************

preserve

keep if sex_n == 2

keep if age_cat == 1

keep if school_n == 0

tabout current_tobacco_any current_smoke_n current_smokeless_n ///

daily_tobacco_any daily_smoke_n daily_smokeless_n ///

smoke_only smokeless_only both_smoke_smokeless ///

alcohol_current no_fruit_veg no_fruit no_veg ///

atelessthan5 salt_eat_n salt_pack_n ///

htn_known htn_med file1 ///

using Table3_n.xls, ///

c(freq col) f(1c 1p) replace

import delimited using Table3_n.xls, clear

drop if v1 == "No"

drop if v1 == "Yes"

replace v1 = v1[_n - 1] if v1 == "Total"

drop v3 v5 v6 v7

drop if v1 == ""

drop if v2 == ""

destring v2 v4, ignore(",") replace

rename v2 IVR_n

rename v4 STEPS_n

gen id = _n

save Table3_n.dta, replace

restore

preserve

keep if sex_n == 2

keep if age_cat == 1

keep if school_n == 0

gen wt = 1

svyset [pweight= wt]

for var current_tobacco_any current_smoke_n current_smokeless_n ///

daily_tobacco_any daily_smoke_n daily_smokeless_n ///

smoke_only smokeless_only both_smoke_smokeless ///

alcohol_current no_fruit_veg no_fruit no_veg ///

atelessthan5 salt_eat_n salt_pack_n ///

htn_known htn_med: tab X file1

tabout current_tobacco_any current_smoke_n current_smokeless_n ///

daily_tobacco_any daily_smoke_n daily_smokeless_n ///

smokeless_only both_smoke_smokeless ///

alcohol_current no_fruit_veg no_fruit no_veg ///

atelessthan5 salt_eat_n salt_pack_n ///

htn_known htn_med file1 ///

using Table3_Unweight.xls, ///

c(col ci) f(1 1) percent svy replace

import delimited using Table3_Unweight.xls, clear

drop v6 v7

drop if v1 == "No" | v1 == "Total"

replace v1 = v1[_n - 1] if v1 == "Yes"

drop if v2 == ""

drop if v1 == ""

rename v2 IVR_Unweight

rename v4 STEPS_Unweight

rename v3 IVR_Unweight_CI

rename v5 STEPS_Unweight_CI

gen id = _n

save Table3_Unweighted.dta, replace

restore

********

preserve

keep if sex_n == 2

keep if age_cat == 1

keep if school_n == 0

svyset [pweight= weight_ipw]

tabout current_tobacco_any current_smoke_n current_smokeless_n ///

daily_tobacco_any daily_smoke_n daily_smokeless_n ///

smokeless_only both_smoke_smokeless ///

alcohol_current no_fruit_veg no_fruit no_veg ///

atelessthan5 salt_eat_n salt_pack_n ///

htn_known htn_med file1 ///

using Table3_Weight_IPW.xls, ///

c(col ci) f(1 1) percent svy replace

import delimited using Table3_Weight_IPW.xls, clear

drop v6 v7

drop if v1 == "No" | v1 == "Total"

replace v1 = v1[_n - 1] if v1 == "Yes"

drop if v2 == ""

drop if v1 == ""

rename v2 IVR_IPW

rename v4 STEPS_IPW

rename v3 IVR_IPW_CI

rename v5 STEPS_IPW_CI

gen id = _n

save Table3_Weighted_IPW.dta, replace

restore

********

preserve

keep if sex_n == 2

keep if age_cat == 1

keep if school_n == 0

svyset [pweight= age_sex_w]

tabout current_tobacco_any current_smoke_n current_smokeless_n ///

daily_tobacco_any daily_smoke_n daily_smokeless_n ///

smokeless_only both_smoke_smokeless ///

alcohol_current no_fruit_veg no_fruit no_veg ///

atelessthan5 salt_eat_n salt_pack_n ///

htn_known htn_med file1 ///

using Table3_Weight_Age_Sex_W.xls, ///

c(col ci) f(1 1) percent svy replace

import delimited using Table3_Weight_Age_Sex_W.xls, clear

drop v6 v7

drop if v1 == "No" | v1 == "Total"

replace v1 = v1[_n - 1] if v1 == "Yes"

drop if v2 == ""

drop if v1 == ""

rename v2 IVR_Age_Sex

rename v4 STEPS_Age_Sex

rename v3 IVR_Age_Sex_CI

rename v5 STEPS_Age_Sex_CI

gen id = _n

save Table3_Weighted_Age_Sex_W.dta, replace

restore

preserve

use Table3_Unweighted.dta, replace

merge 1:1 v1 using Table3_Weighted_IPW, nogen

merge 1:1 v1 using Table3_Weighted_Age_Sex_W.dta, nogen

merge 1:1 v1 using Table3_n.dta, nogen

sort id

drop id

label var v1 "Indicator"

drop STEPS_IPW

gen indicator = _n

gen pop_group = 28

save Table3_28_Prevalence_GMAK_20Jul2022.dta, replace

restore

cap erase Table3_Unweighted.dta

cap erase Table3_Weighted_IPW.dta

cap erase Table3_Weighted_Age_Sex_W.dta

cap erase Table3_Weight_Age_Sex_W.xls

cap erase Table3_Weight_IPW.xls

cap erase Table3_Unweight.xls

*******************************************************************************

preserve

keep if sex_n == 2

keep if age_cat == 1

keep if school_n == 1

tabout current_tobacco_any current_smoke_n current_smokeless_n ///

daily_tobacco_any daily_smoke_n daily_smokeless_n ///

smoke_only smokeless_only both_smoke_smokeless ///

alcohol_current no_fruit_veg no_fruit no_veg ///

atelessthan5 salt_eat_n salt_pack_n ///

htn_known htn_med file1 ///

using Table3_n.xls, ///

c(freq col) f(1c 1p) replace

import delimited using Table3_n.xls, clear

drop if v1 == "No"

drop if v1 == "Yes"

replace v1 = v1[_n - 1] if v1 == "Total"

drop v3 v5 v6 v7

drop if v1 == ""

drop if v2 == ""

destring v2 v4, ignore(",") replace

rename v2 IVR_n

rename v4 STEPS_n

gen id = _n

save Table3_n.dta, replace

restore

preserve

keep if sex_n == 2

keep if age_cat == 1

keep if school_n == 1

gen wt = 1

svyset [pweight= wt]

for var current_tobacco_any current_smoke_n current_smokeless_n ///

daily_tobacco_any daily_smoke_n daily_smokeless_n ///

smoke_only smokeless_only both_smoke_smokeless ///

alcohol_current no_fruit_veg no_fruit no_veg ///

atelessthan5 salt_eat_n salt_pack_n ///

htn_known htn_med: tab X file1

tabout current_tobacco_any current_smoke_n current_smokeless_n ///

daily_tobacco_any daily_smoke_n daily_smokeless_n ///

smoke_only smokeless_only both_smoke_smokeless no_fruit ///

atelessthan5 salt_eat_n salt_pack_n ///

htn_known htn_med file1 ///

using Table3_Unweight.xls, ///

c(col ci) f(1 1) percent svy replace

import delimited using Table3_Unweight.xls, clear

drop v6 v7

drop if v1 == "No" | v1 == "Total"

replace v1 = v1[_n - 1] if v1 == "Yes"

drop if v2 == ""

drop if v1 == ""

rename v2 IVR_Unweight

rename v4 STEPS_Unweight

rename v3 IVR_Unweight_CI

rename v5 STEPS_Unweight_CI

gen id = _n

save Table3_Unweighted.dta, replace

restore

********

preserve

keep if sex_n == 2

keep if age_cat == 1

keep if school_n == 1

svyset [pweight= weight_ipw]

tabout current_tobacco_any current_smoke_n current_smokeless_n ///

daily_tobacco_any daily_smoke_n daily_smokeless_n ///

smoke_only smokeless_only both_smoke_smokeless no_fruit ///

atelessthan5 salt_eat_n salt_pack_n ///

htn_known htn_med file1 ///

using Table3_Weight_IPW.xls, ///

c(col ci) f(1 1) percent svy replace

import delimited using Table3_Weight_IPW.xls, clear

drop v6 v7

drop if v1 == "No" | v1 == "Total"

replace v1 = v1[_n - 1] if v1 == "Yes"

drop if v2 == ""

drop if v1 == ""

rename v2 IVR_IPW

rename v4 STEPS_IPW

rename v3 IVR_IPW_CI

rename v5 STEPS_IPW_CI

gen id = _n

save Table3_Weighted_IPW.dta, replace

restore

********

preserve

keep if sex_n == 2

keep if age_cat == 1

keep if school_n == 1

svyset [pweight= age_sex_w]

tabout current_tobacco_any current_smoke_n current_smokeless_n ///

daily_tobacco_any daily_smoke_n daily_smokeless_n ///

smoke_only smokeless_only both_smoke_smokeless no_fruit ///

atelessthan5 salt_eat_n salt_pack_n ///

htn_known htn_med file1 ///

using Table3_Weight_Age_Sex_W.xls, ///

c(col ci) f(1 1) percent svy replace

import delimited using Table3_Weight_Age_Sex_W.xls, clear

drop v6 v7

drop if v1 == "No" | v1 == "Total"

replace v1 = v1[_n - 1] if v1 == "Yes"

drop if v2 == ""

drop if v1 == ""

rename v2 IVR_Age_Sex

rename v4 STEPS_Age_Sex

rename v3 IVR_Age_Sex_CI

rename v5 STEPS_Age_Sex_CI

gen id = _n

save Table3_Weighted_Age_Sex_W.dta, replace

restore

preserve

use Table3_Unweighted.dta, replace

merge 1:1 v1 using Table3_Weighted_IPW, nogen

merge 1:1 v1 using Table3_Weighted_Age_Sex_W.dta, nogen

merge 1:1 v1 using Table3_n.dta, nogen

sort id

drop id

label var v1 "Indicator"

drop STEPS_IPW

gen indicator = _n

gen pop_group = 29

save Table3_29_Prevalence_GMAK_20Jul2022.dta, replace

restore

cap erase Table3_Unweighted.dta

cap erase Table3_Weighted_IPW.dta

cap erase Table3_Weighted_Age_Sex_W.dta

cap erase Table3_Weight_Age_Sex_W.xls

cap erase Table3_Weight_IPW.xls

cap erase Table3_Unweight.xls

*******************************************************************************

preserve

keep if sex_n == 2

keep if age_cat == 1

keep if school_n == 2

tabout current_tobacco_any current_smoke_n current_smokeless_n ///

daily_tobacco_any daily_smoke_n daily_smokeless_n ///

smoke_only smokeless_only both_smoke_smokeless ///

alcohol_current no_fruit_veg no_fruit no_veg ///

atelessthan5 salt_eat_n salt_pack_n ///

htn_known htn_med file1 ///

using Table3_n.xls, ///

c(freq col) f(1c 1p) replace

import delimited using Table3_n.xls, clear

drop if v1 == "No"

drop if v1 == "Yes"

replace v1 = v1[_n - 1] if v1 == "Total"

drop v3 v5 v6 v7

drop if v1 == ""

drop if v2 == ""

destring v2 v4, ignore(",") replace

rename v2 IVR_n

rename v4 STEPS_n

gen id = _n

save Table3_n.dta, replace

restore

preserve

keep if sex_n == 2

keep if age_cat == 1

keep if school_n == 2

gen wt = 1

svyset [pweight= wt]

for var current_tobacco_any current_smoke_n current_smokeless_n ///

daily_tobacco_any daily_smoke_n daily_smokeless_n ///

smoke_only smokeless_only both_smoke_smokeless ///

alcohol_current no_fruit_veg no_fruit no_veg ///

atelessthan5 salt_eat_n salt_pack_n ///

htn_known htn_med: tab X file1

tabout current_tobacco_any current_smoke_n current_smokeless_n ///

daily_tobacco_any daily_smoke_n daily_smokeless_n ///

smoke_only smokeless_only ///

no_fruit no_veg ///

atelessthan5 salt_eat_n salt_pack_n ///

htn_known htn_med file1 ///

using Table3_Unweight.xls, ///

c(col ci) f(1 1) percent svy replace

import delimited using Table3_Unweight.xls, clear

drop v6 v7

drop if v1 == "No" | v1 == "Total"

replace v1 = v1[_n - 1] if v1 == "Yes"

drop if v2 == ""

drop if v1 == ""

rename v2 IVR_Unweight

rename v4 STEPS_Unweight

rename v3 IVR_Unweight_CI

rename v5 STEPS_Unweight_CI

gen id = _n

save Table3_Unweighted.dta, replace

restore

********

preserve

keep if sex_n == 2

keep if age_cat == 1

keep if school_n == 2

svyset [pweight= weight_ipw]

tabout current_tobacco_any current_smoke_n current_smokeless_n ///

daily_tobacco_any daily_smoke_n daily_smokeless_n ///

smoke_only smokeless_only ///

no_fruit no_veg ///

atelessthan5 salt_eat_n salt_pack_n ///

htn_known htn_med file1 ///

using Table3_Weight_IPW.xls, ///

c(col ci) f(1 1) percent svy replace

import delimited using Table3_Weight_IPW.xls, clear

drop v6 v7

drop if v1 == "No" | v1 == "Total"

replace v1 = v1[_n - 1] if v1 == "Yes"

drop if v2 == ""

drop if v1 == ""

rename v2 IVR_IPW

rename v4 STEPS_IPW

rename v3 IVR_IPW_CI

rename v5 STEPS_IPW_CI

gen id = _n

save Table3_Weighted_IPW.dta, replace

restore

********

preserve

keep if sex_n == 2

keep if age_cat == 1

keep if school_n == 2

svyset [pweight= age_sex_w]

tabout current_tobacco_any current_smoke_n current_smokeless_n ///

daily_tobacco_any daily_smoke_n daily_smokeless_n ///

smoke_only smokeless_only ///

no_fruit no_veg ///

atelessthan5 salt_eat_n salt_pack_n ///

htn_known htn_med file1 ///

using Table3_Weight_Age_Sex_W.xls, ///

c(col ci) f(1 1) percent svy replace

import delimited using Table3_Weight_Age_Sex_W.xls, clear

drop v6 v7

drop if v1 == "No" | v1 == "Total"

replace v1 = v1[_n - 1] if v1 == "Yes"

drop if v2 == ""

drop if v1 == ""

rename v2 IVR_Age_Sex

rename v4 STEPS_Age_Sex

rename v3 IVR_Age_Sex_CI

rename v5 STEPS_Age_Sex_CI

gen id = _n

save Table3_Weighted_Age_Sex_W.dta, replace

restore

preserve

use Table3_Unweighted.dta, replace

merge 1:1 v1 using Table3_Weighted_IPW, nogen

merge 1:1 v1 using Table3_Weighted_Age_Sex_W.dta, nogen

merge 1:1 v1 using Table3_n.dta, nogen

sort id

drop id

label var v1 "Indicator"

drop STEPS_IPW

gen indicator = _n

gen pop_group = 30

save Table3_30_Prevalence_GMAK_20Jul2022.dta, replace

restore

cap erase Table3_Unweighted.dta

cap erase Table3_Weighted_IPW.dta

cap erase Table3_Weighted_Age_Sex_W.dta

cap erase Table3_Weight_Age_Sex_W.xls

cap erase Table3_Weight_IPW.xls

cap erase Table3_Unweight.xls

*******************************************************************************

preserve

keep if sex_n == 2

keep if age_cat == 2

keep if school_n == 0

tabout current_tobacco_any current_smoke_n current_smokeless_n ///

daily_tobacco_any daily_smoke_n daily_smokeless_n ///

smoke_only smokeless_only both_smoke_smokeless ///

alcohol_current no_fruit_veg no_fruit no_veg ///

atelessthan5 salt_eat_n salt_pack_n ///

htn_known htn_med file1 ///

using Table3_n.xls, ///

c(freq col) f(1c 1p) replace

import delimited using Table3_n.xls, clear

drop if v1 == "No"

drop if v1 == "Yes"

replace v1 = v1[_n - 1] if v1 == "Total"

drop v3 v5 v6 v7

drop if v1 == ""

drop if v2 == ""

destring v2 v4, ignore(",") replace

rename v2 IVR_n

rename v4 STEPS_n

gen id = _n

save Table3_n.dta, replace

restore

preserve

keep if sex_n == 2

keep if age_cat == 2

keep if school_n == 0

gen wt = 1

svyset [pweight= wt]

tabout current_tobacco_any current_smoke_n current_smokeless_n ///

daily_tobacco_any daily_smoke_n daily_smokeless_n ///

smoke_only smokeless_only both_smoke_smokeless ///

alcohol_current no_fruit_veg no_fruit no_veg ///

atelessthan5 salt_eat_n salt_pack_n ///

htn_known htn_med file1 ///

using Table3_Unweight.xls, ///

c(col ci) f(1 1) percent svy replace

import delimited using Table3_Unweight.xls, clear

drop v6 v7

drop if v1 == "No" | v1 == "Total"

replace v1 = v1[_n - 1] if v1 == "Yes"

drop if v2 == ""

drop if v1 == ""

rename v2 IVR_Unweight

rename v4 STEPS_Unweight

rename v3 IVR_Unweight_CI

rename v5 STEPS_Unweight_CI

gen id = _n

save Table3_Unweighted.dta, replace

restore

********

preserve

keep if sex_n == 2

keep if age_cat == 2

keep if school_n == 0

svyset [pweight= weight_ipw]

tabout current_tobacco_any current_smoke_n current_smokeless_n ///

daily_tobacco_any daily_smoke_n daily_smokeless_n ///

smoke_only smokeless_only both_smoke_smokeless ///

alcohol_current no_fruit_veg no_fruit no_veg ///

atelessthan5 salt_eat_n salt_pack_n ///

htn_known htn_med file1 ///

using Table3_Weight_IPW.xls, ///

c(col ci) f(1 1) percent svy replace

import delimited using Table3_Weight_IPW.xls, clear

drop v6 v7

drop if v1 == "No" | v1 == "Total"

replace v1 = v1[_n - 1] if v1 == "Yes"

drop if v2 == ""

drop if v1 == ""

rename v2 IVR_IPW

rename v4 STEPS_IPW

rename v3 IVR_IPW_CI

rename v5 STEPS_IPW_CI

gen id = _n

save Table3_Weighted_IPW.dta, replace

restore

********

preserve

keep if sex_n == 2

keep if age_cat == 2

keep if school_n == 0

svyset [pweight= age_sex_w]

tabout current_tobacco_any current_smoke_n current_smokeless_n ///

daily_tobacco_any daily_smoke_n daily_smokeless_n ///

smoke_only smokeless_only both_smoke_smokeless ///

alcohol_current no_fruit_veg no_fruit no_veg ///

atelessthan5 salt_eat_n salt_pack_n ///

htn_known htn_med file1 ///

using Table3_Weight_Age_Sex_W.xls, ///

c(col ci) f(1 1) percent svy replace

import delimited using Table3_Weight_Age_Sex_W.xls, clear

drop v6 v7

drop if v1 == "No" | v1 == "Total"

replace v1 = v1[_n - 1] if v1 == "Yes"

drop if v2 == ""

drop if v1 == ""

rename v2 IVR_Age_Sex

rename v4 STEPS_Age_Sex

rename v3 IVR_Age_Sex_CI

rename v5 STEPS_Age_Sex_CI

gen id = _n

save Table3_Weighted_Age_Sex_W.dta, replace

restore

preserve

use Table3_Unweighted.dta, replace

merge 1:1 v1 using Table3_Weighted_IPW, nogen

merge 1:1 v1 using Table3_Weighted_Age_Sex_W.dta, nogen

merge 1:1 v1 using Table3_n.dta, nogen

sort id

drop id

label var v1 "Indicator"

drop STEPS_IPW

gen indicator = _n

gen pop_group = 31

save Table3_31_Prevalence_GMAK_20Jul2022.dta, replace

restore

cap erase Table3_Unweighted.dta

cap erase Table3_Weighted_IPW.dta

cap erase Table3_Weighted_Age_Sex_W.dta

cap erase Table3_Weight_Age_Sex_W.xls

cap erase Table3_Weight_IPW.xls

cap erase Table3_Unweight.xls

*******************************************************************************

preserve

keep if sex_n == 2

keep if age_cat == 2

keep if school_n == 1

tabout current_tobacco_any current_smoke_n current_smokeless_n ///

daily_tobacco_any daily_smoke_n daily_smokeless_n ///

smoke_only smokeless_only both_smoke_smokeless ///

alcohol_current no_fruit_veg no_fruit no_veg ///

atelessthan5 salt_eat_n salt_pack_n ///

htn_known htn_med file1 ///

using Table3_n.xls, ///

c(freq col) f(1c 1p) replace

import delimited using Table3_n.xls, clear

drop if v1 == "No"

drop if v1 == "Yes"

replace v1 = v1[_n - 1] if v1 == "Total"

drop v3 v5 v6 v7

drop if v1 == ""

drop if v2 == ""

destring v2 v4, ignore(",") replace

rename v2 IVR_n

rename v4 STEPS_n

gen id = _n

save Table3_n.dta, replace

restore

preserve

keep if sex_n == 2

keep if age_cat == 2

keep if school_n == 1

gen wt = 1

svyset [pweight= wt]

for var current_tobacco_any current_smoke_n current_smokeless_n ///

daily_tobacco_any daily_smoke_n daily_smokeless_n ///

smoke_only smokeless_only both_smoke_smokeless ///

alcohol_current no_fruit_veg no_fruit no_veg ///

atelessthan5 salt_eat_n salt_pack_n ///

htn_known htn_med: tab X file1

tabout current_tobacco_any current_smokeless_n ///

daily_tobacco_any daily_smokeless_n ///

smokeless_only no_fruit ///

atelessthan5 salt_eat_n salt_pack_n ///

htn_known htn_med file1 ///

using Table3_Unweight.xls, ///

c(col ci) f(1 1) percent svy replace

import delimited using Table3_Unweight.xls, clear

drop v6 v7

drop if v1 == "No" | v1 == "Total"

replace v1 = v1[_n - 1] if v1 == "Yes"

drop if v2 == ""

drop if v1 == ""

rename v2 IVR_Unweight

rename v4 STEPS_Unweight

rename v3 IVR_Unweight_CI

rename v5 STEPS_Unweight_CI

gen id = _n

save Table3_Unweighted.dta, replace

restore

********

preserve

keep if sex_n == 2

keep if age_cat == 2

keep if school_n == 1

svyset [pweight= weight_ipw]

tabout current_tobacco_any current_smokeless_n ///

daily_tobacco_any daily_smokeless_n ///

smokeless_only no_fruit ///

atelessthan5 salt_eat_n salt_pack_n ///

htn_known htn_med file1 ///

using Table3_Weight_IPW.xls, ///

c(col ci) f(1 1) percent svy replace

import delimited using Table3_Weight_IPW.xls, clear

drop v6 v7

drop if v1 == "No" | v1 == "Total"

replace v1 = v1[_n - 1] if v1 == "Yes"

drop if v2 == ""

drop if v1 == ""

rename v2 IVR_IPW

rename v4 STEPS_IPW

rename v3 IVR_IPW_CI

rename v5 STEPS_IPW_CI

gen id = _n

save Table3_Weighted_IPW.dta, replace

restore

********

preserve

keep if sex_n == 2

keep if age_cat == 2

keep if school_n == 1

svyset [pweight= age_sex_w]

tabout current_tobacco_any current_smokeless_n ///

daily_tobacco_any daily_smokeless_n ///

smokeless_only no_fruit ///

atelessthan5 salt_eat_n salt_pack_n ///

htn_known htn_med file1 ///

using Table3_Weight_Age_Sex_W.xls, ///

c(col ci) f(1 1) percent svy replace

import delimited using Table3_Weight_Age_Sex_W.xls, clear

drop v6 v7

drop if v1 == "No" | v1 == "Total"

replace v1 = v1[_n - 1] if v1 == "Yes"

drop if v2 == ""

drop if v1 == ""

rename v2 IVR_Age_Sex

rename v4 STEPS_Age_Sex

rename v3 IVR_Age_Sex_CI

rename v5 STEPS_Age_Sex_CI

gen id = _n

save Table3_Weighted_Age_Sex_W.dta, replace

restore

preserve

use Table3_Unweighted.dta, replace

merge 1:1 v1 using Table3_Weighted_IPW, nogen

merge 1:1 v1 using Table3_Weighted_Age_Sex_W.dta, nogen

merge 1:1 v1 using Table3_n.dta, nogen

sort id

drop id

label var v1 "Indicator"

drop STEPS_IPW

gen indicator = _n

gen pop_group = 32

save Table3_32_Prevalence_GMAK_20Jul2022.dta, replace

restore

cap erase Table3_Unweighted.dta

cap erase Table3_Weighted_IPW.dta

cap erase Table3_Weighted_Age_Sex_W.dta

cap erase Table3_Weight_Age_Sex_W.xls

cap erase Table3_Weight_IPW.xls

cap erase Table3_Unweight.xls

*******************************************************************************

preserve

keep if sex_n == 2

keep if age_cat == 2

keep if school_n == 2

tabout current_tobacco_any current_smoke_n current_smokeless_n ///

daily_tobacco_any daily_smoke_n daily_smokeless_n ///

smoke_only smokeless_only both_smoke_smokeless ///

alcohol_current no_fruit_veg no_fruit no_veg ///

atelessthan5 salt_eat_n salt_pack_n ///

htn_known htn_med file1 ///

using Table3_n.xls, ///

c(freq col) f(1c 1p) replace

import delimited using Table3_n.xls, clear

drop if v1 == "No"

drop if v1 == "Yes"

replace v1 = v1[_n - 1] if v1 == "Total"

drop v3 v5 v6 v7

drop if v1 == ""

drop if v2 == ""

destring v2 v4, ignore(",") replace

rename v2 IVR_n

rename v4 STEPS_n

gen id = _n

save Table3_n.dta, replace

restore

preserve

keep if sex_n == 2

keep if age_cat == 2

keep if school_n == 2

gen wt = 1

svyset [pweight= wt]

for var current_tobacco_any current_smoke_n current_smokeless_n ///

daily_tobacco_any daily_smoke_n daily_smokeless_n ///

smoke_only smokeless_only both_smoke_smokeless ///

alcohol_current no_fruit_veg no_fruit no_veg ///

atelessthan5 salt_eat_n salt_pack_n ///

htn_known htn_med: tab X file1

tabout current_tobacco_any current_smokeless_n ///

daily_tobacco_any daily_smokeless_n ///

smokeless_only ///

alcohol_current no_fruit no_veg ///

atelessthan5 salt_eat_n salt_pack_n ///

htn_known htn_med file1 ///

using Table3_Unweight.xls, ///

c(col ci) f(1 1) percent svy replace

import delimited using Table3_Unweight.xls, clear

drop v6 v7

drop if v1 == "No" | v1 == "Total"

replace v1 = v1[_n - 1] if v1 == "Yes"

drop if v2 == ""

drop if v1 == ""

rename v2 IVR_Unweight

rename v4 STEPS_Unweight

rename v3 IVR_Unweight_CI

rename v5 STEPS_Unweight_CI

gen id = _n

save Table3_Unweighted.dta, replace

restore

********

preserve

keep if sex_n == 2

keep if age_cat == 2

keep if school_n == 2

svyset [pweight= weight_ipw]

tabout current_tobacco_any current_smokeless_n ///

daily_tobacco_any daily_smokeless_n ///

smokeless_only ///

alcohol_current no_fruit no_veg ///

atelessthan5 salt_eat_n salt_pack_n ///

htn_known htn_med file1 ///

using Table3_Weight_IPW.xls, ///

c(col ci) f(1 1) percent svy replace

import delimited using Table3_Weight_IPW.xls, clear

drop v6 v7

drop if v1 == "No" | v1 == "Total"

replace v1 = v1[_n - 1] if v1 == "Yes"

drop if v2 == ""

drop if v1 == ""

rename v2 IVR_IPW

rename v4 STEPS_IPW

rename v3 IVR_IPW_CI

rename v5 STEPS_IPW_CI

gen id = _n

save Table3_Weighted_IPW.dta, replace

restore

********

preserve

keep if sex_n == 2

keep if age_cat == 2

keep if school_n == 2

svyset [pweight= age_sex_w]

tabout current_tobacco_any current_smokeless_n ///

daily_tobacco_any daily_smokeless_n ///

smokeless_only ///

alcohol_current no_fruit no_veg ///

atelessthan5 salt_eat_n salt_pack_n ///

htn_known htn_med file1 ///

using Table3_Weight_Age_Sex_W.xls, ///

c(col ci) f(1 1) percent svy replace

import delimited using Table3_Weight_Age_Sex_W.xls, clear

drop v6 v7

drop if v1 == "No" | v1 == "Total"

replace v1 = v1[_n - 1] if v1 == "Yes"

drop if v2 == ""

drop if v1 == ""

rename v2 IVR_Age_Sex

rename v4 STEPS_Age_Sex

rename v3 IVR_Age_Sex_CI

rename v5 STEPS_Age_Sex_CI

gen id = _n

save Table3_Weighted_Age_Sex_W.dta, replace

restore

preserve

use Table3_Unweighted.dta, replace

merge 1:1 v1 using Table3_Weighted_IPW, nogen

merge 1:1 v1 using Table3_Weighted_Age_Sex_W.dta, nogen

merge 1:1 v1 using Table3_n.dta, nogen

sort id

drop id

label var v1 "Indicator"

drop STEPS_IPW

gen indicator = _n

gen pop_group = 33

save Table3_33_Prevalence_GMAK_20Jul2022.dta, replace

restore

cap erase Table3_Unweighted.dta

cap erase Table3_Weighted_IPW.dta

cap erase Table3_Weighted_Age_Sex_W.dta

cap erase Table3_Weight_Age_Sex_W.xls

cap erase Table3_Weight_IPW.xls

cap erase Table3_Unweight.xls

*******************************************************************************

preserve

keep if sex_n == 2

keep if school_n == 0

tabout current_tobacco_any current_smoke_n current_smokeless_n ///

daily_tobacco_any daily_smoke_n daily_smokeless_n ///

smoke_only smokeless_only both_smoke_smokeless ///

alcohol_current no_fruit_veg no_fruit no_veg ///

atelessthan5 salt_eat_n salt_pack_n ///

htn_known htn_med file1 ///

using Table3_n.xls, ///

c(freq col) f(1c 1p) replace

import delimited using Table3_n.xls, clear

drop if v1 == "No"

drop if v1 == "Yes"

replace v1 = v1[_n - 1] if v1 == "Total"

drop v3 v5 v6 v7

drop if v1 == ""

drop if v2 == ""

destring v2 v4, ignore(",") replace

rename v2 IVR_n

rename v4 STEPS_n

gen id = _n

save Table3_n.dta, replace

restore

preserve

keep if sex_n == 2

keep if school_n == 0

gen wt = 1

svyset [pweight= wt]

tabout current_tobacco_any current_smoke_n current_smokeless_n ///

daily_tobacco_any daily_smoke_n daily_smokeless_n ///

smoke_only smokeless_only both_smoke_smokeless ///

alcohol_current no_fruit_veg no_fruit no_veg ///

atelessthan5 salt_eat_n salt_pack_n ///

htn_known htn_med file1 ///

using Table3_Unweight.xls, ///

c(col ci) f(1 1) percent svy replace

import delimited using Table3_Unweight.xls, clear

drop v6 v7

drop if v1 == "No" | v1 == "Total"

replace v1 = v1[_n - 1] if v1 == "Yes"

drop if v2 == ""

drop if v1 == ""

rename v2 IVR_Unweight

rename v4 STEPS_Unweight

rename v3 IVR_Unweight_CI

rename v5 STEPS_Unweight_CI

gen id = _n

save Table3_Unweighted.dta, replace

restore

********

preserve

keep if sex_n == 2

keep if school_n == 0

svyset [pweight= weight_ipw]

tabout current_tobacco_any current_smoke_n current_smokeless_n ///

daily_tobacco_any daily_smoke_n daily_smokeless_n ///

smoke_only smokeless_only both_smoke_smokeless ///

alcohol_current no_fruit_veg no_fruit no_veg ///

atelessthan5 salt_eat_n salt_pack_n ///

htn_known htn_med file1 ///

using Table3_Weight_IPW.xls, ///

c(col ci) f(1 1) percent svy replace

import delimited using Table3_Weight_IPW.xls, clear

drop v6 v7

drop if v1 == "No" | v1 == "Total"

replace v1 = v1[_n - 1] if v1 == "Yes"

drop if v2 == ""

drop if v1 == ""

rename v2 IVR_IPW

rename v4 STEPS_IPW

rename v3 IVR_IPW_CI

rename v5 STEPS_IPW_CI

gen id = _n

save Table3_Weighted_IPW.dta, replace

restore

********

preserve

keep if sex_n == 2

keep if school_n == 0

svyset [pweight= age_sex_w]

tabout current_tobacco_any current_smoke_n current_smokeless_n ///

daily_tobacco_any daily_smoke_n daily_smokeless_n ///

smoke_only smokeless_only both_smoke_smokeless ///

alcohol_current no_fruit_veg no_fruit no_veg ///

atelessthan5 salt_eat_n salt_pack_n ///

htn_known htn_med file1 ///

using Table3_Weight_Age_Sex_W.xls, ///

c(col ci) f(1 1) percent svy replace

import delimited using Table3_Weight_Age_Sex_W.xls, clear

drop v6 v7

drop if v1 == "No" | v1 == "Total"

replace v1 = v1[_n - 1] if v1 == "Yes"

drop if v2 == ""

drop if v1 == ""

rename v2 IVR_Age_Sex

rename v4 STEPS_Age_Sex

rename v3 IVR_Age_Sex_CI

rename v5 STEPS_Age_Sex_CI

gen id = _n

save Table3_Weighted_Age_Sex_W.dta, replace

restore

preserve

use Table3_Unweighted.dta, replace

merge 1:1 v1 using Table3_Weighted_IPW, nogen

merge 1:1 v1 using Table3_Weighted_Age_Sex_W.dta, nogen

merge 1:1 v1 using Table3_n.dta, nogen

sort id

drop id

label var v1 "Indicator"

drop STEPS_IPW

gen indicator = _n

gen pop_group = 34

save Table3_34_Prevalence_GMAK_20Jul2022.dta, replace

restore

cap erase Table3_Unweighted.dta

cap erase Table3_Weighted_IPW.dta

cap erase Table3_Weighted_Age_Sex_W.dta

cap erase Table3_Weight_Age_Sex_W.xls

cap erase Table3_Weight_IPW.xls

cap erase Table3_Unweight.xls

*******************************************************************************

preserve

keep if sex_n == 2

keep if school_n == 1

tabout current_tobacco_any current_smoke_n current_smokeless_n ///

daily_tobacco_any daily_smoke_n daily_smokeless_n ///

smoke_only smokeless_only both_smoke_smokeless ///

alcohol_current no_fruit_veg no_fruit no_veg ///

atelessthan5 salt_eat_n salt_pack_n ///

htn_known htn_med file1 ///

using Table3_n.xls, ///

c(freq col) f(1c 1p) replace

import delimited using Table3_n.xls, clear

drop if v1 == "No"

drop if v1 == "Yes"

replace v1 = v1[_n - 1] if v1 == "Total"

drop v3 v5 v6 v7

drop if v1 == ""

drop if v2 == ""

destring v2 v4, ignore(",") replace

rename v2 IVR_n

rename v4 STEPS_n

gen id = _n

save Table3_n.dta, replace

restore

preserve

keep if sex_n == 2

keep if school_n == 1

gen wt = 1

svyset [pweight= wt]

for var current_tobacco_any current_smoke_n current_smokeless_n ///

daily_tobacco_any daily_smoke_n daily_smokeless_n ///

smoke_only smokeless_only both_smoke_smokeless ///

alcohol_current no_fruit_veg no_fruit no_veg ///

atelessthan5 salt_eat_n salt_pack_n ///

htn_known htn_med: tab X file1

tabout current_tobacco_any current_smoke_n current_smokeless_n ///

daily_tobacco_any daily_smoke_n daily_smokeless_n ///

smoke_only smokeless_only both_smoke_smokeless ///

no_fruit_veg no_fruit no_veg ///

atelessthan5 salt_eat_n salt_pack_n ///

htn_known htn_med file1 ///

using Table3_Unweight.xls, ///

c(col ci) f(1 1) percent svy replace

import delimited using Table3_Unweight.xls, clear

drop v6 v7

drop if v1 == "No" | v1 == "Total"

replace v1 = v1[_n - 1] if v1 == "Yes"

drop if v2 == ""

drop if v1 == ""

rename v2 IVR_Unweight

rename v4 STEPS_Unweight

rename v3 IVR_Unweight_CI

rename v5 STEPS_Unweight_CI

gen id = _n

save Table3_Unweighted.dta, replace

restore

********

preserve

keep if sex_n == 2

keep if school_n == 1

svyset [pweight= weight_ipw]

tabout current_tobacco_any current_smoke_n current_smokeless_n ///

daily_tobacco_any daily_smoke_n daily_smokeless_n ///

smoke_only smokeless_only both_smoke_smokeless ///

no_fruit_veg no_fruit no_veg ///

atelessthan5 salt_eat_n salt_pack_n ///

htn_known htn_med file1 ///

using Table3_Weight_IPW.xls, ///

c(col ci) f(1 1) percent svy replace

import delimited using Table3_Weight_IPW.xls, clear

drop v6 v7

drop if v1 == "No" | v1 == "Total"

replace v1 = v1[_n - 1] if v1 == "Yes"

drop if v2 == ""

drop if v1 == ""

rename v2 IVR_IPW

rename v4 STEPS_IPW

rename v3 IVR_IPW_CI

rename v5 STEPS_IPW_CI

gen id = _n

save Table3_Weighted_IPW.dta, replace

restore

********

preserve

keep if sex_n == 2

keep if school_n == 1

svyset [pweight= age_sex_w]

tabout current_tobacco_any current_smoke_n current_smokeless_n ///

daily_tobacco_any daily_smoke_n daily_smokeless_n ///

smoke_only smokeless_only both_smoke_smokeless ///

no_fruit_veg no_fruit no_veg ///

atelessthan5 salt_eat_n salt_pack_n ///

htn_known htn_med file1 ///

using Table3_Weight_Age_Sex_W.xls, ///

c(col ci) f(1 1) percent svy replace

import delimited using Table3_Weight_Age_Sex_W.xls, clear

drop v6 v7

drop if v1 == "No" | v1 == "Total"

replace v1 = v1[_n - 1] if v1 == "Yes"

drop if v2 == ""

drop if v1 == ""

rename v2 IVR_Age_Sex

rename v4 STEPS_Age_Sex

rename v3 IVR_Age_Sex_CI

rename v5 STEPS_Age_Sex_CI

gen id = _n

save Table3_Weighted_Age_Sex_W.dta, replace

restore

preserve

use Table3_Unweighted.dta, replace

merge 1:1 v1 using Table3_Weighted_IPW, nogen

merge 1:1 v1 using Table3_Weighted_Age_Sex_W.dta, nogen

merge 1:1 v1 using Table3_n.dta, nogen

sort id

drop id

label var v1 "Indicator"

drop STEPS_IPW

gen indicator = _n

gen pop_group = 35

save Table3_35_Prevalence_GMAK_20Jul2022.dta, replace

restore

*******************************************************************************

preserve

keep if sex_n == 2

keep if school_n == 2

tabout current_tobacco_any current_smoke_n current_smokeless_n ///

daily_tobacco_any daily_smoke_n daily_smokeless_n ///

smoke_only smokeless_only both_smoke_smokeless ///

alcohol_current no_fruit_veg no_fruit no_veg ///

atelessthan5 salt_eat_n salt_pack_n ///

htn_known htn_med file1 ///

using Table3_n.xls, ///

c(freq col) f(1c 1p) replace

import delimited using Table3_n.xls, clear

drop if v1 == "No"

drop if v1 == "Yes"

replace v1 = v1[_n - 1] if v1 == "Total"

drop v3 v5 v6 v7

drop if v1 == ""

drop if v2 == ""

destring v2 v4, ignore(",") replace

rename v2 IVR_n

rename v4 STEPS_n

gen id = _n

save Table3_n.dta, replace

restore

preserve

keep if sex_n == 2

keep if school_n == 2

gen wt = 1

svyset [pweight= wt]

for var current_tobacco_any current_smoke_n current_smokeless_n ///

daily_tobacco_any daily_smoke_n daily_smokeless_n ///

smoke_only smokeless_only both_smoke_smokeless ///

alcohol_current no_fruit_veg no_fruit no_veg ///

atelessthan5 salt_eat_n salt_pack_n ///

htn_known htn_med: tab X file1

tabout current_tobacco_any current_smoke_n current_smokeless_n ///

daily_tobacco_any daily_smoke_n daily_smokeless_n ///

smoke_only smokeless_only ///

alcohol_current no_fruit_veg no_fruit no_veg ///

atelessthan5 salt_eat_n salt_pack_n ///

htn_known htn_med file1 ///

using Table3_Unweight.xls, ///

c(col ci) f(1 1) percent svy replace

import delimited using Table3_Unweight.xls, clear

drop v6 v7

drop if v1 == "No" | v1 == "Total"

replace v1 = v1[_n - 1] if v1 == "Yes"

drop if v2 == ""

drop if v1 == ""

rename v2 IVR_Unweight

rename v4 STEPS_Unweight

rename v3 IVR_Unweight_CI

rename v5 STEPS_Unweight_CI

gen id = _n

save Table3_Unweighted.dta, replace

restore

********

preserve

keep if sex_n == 2

keep if school_n == 2

svyset [pweight= weight_ipw]

tabout current_tobacco_any current_smoke_n current_smokeless_n ///

daily_tobacco_any daily_smoke_n daily_smokeless_n ///

smoke_only smokeless_only ///

alcohol_current no_fruit_veg no_fruit no_veg ///

atelessthan5 salt_eat_n salt_pack_n ///

htn_known htn_med file1 ///

using Table3_Weight_IPW.xls, ///

c(col ci) f(1 1) percent svy replace

import delimited using Table3_Weight_IPW.xls, clear

drop v6 v7

drop if v1 == "No" | v1 == "Total"

replace v1 = v1[_n - 1] if v1 == "Yes"

drop if v2 == ""

drop if v1 == ""

rename v2 IVR_IPW

rename v4 STEPS_IPW

rename v3 IVR_IPW_CI

rename v5 STEPS_IPW_CI

gen id = _n

save Table3_Weighted_IPW.dta, replace

restore

********

preserve

keep if sex_n == 2

keep if school_n == 2

svyset [pweight= age_sex_w]

tabout current_tobacco_any current_smoke_n current_smokeless_n ///

daily_tobacco_any daily_smoke_n daily_smokeless_n ///

smoke_only smokeless_only ///

alcohol_current no_fruit_veg no_fruit no_veg ///

atelessthan5 salt_eat_n salt_pack_n ///

htn_known htn_med file1 ///

using Table3_Weight_Age_Sex_W.xls, ///

c(col ci) f(1 1) percent svy replace

import delimited using Table3_Weight_Age_Sex_W.xls, clear

drop v6 v7

drop if v1 == "No" | v1 == "Total"

replace v1 = v1[_n - 1] if v1 == "Yes"

drop if v2 == ""

drop if v1 == ""

rename v2 IVR_Age_Sex

rename v4 STEPS_Age_Sex

rename v3 IVR_Age_Sex_CI

rename v5 STEPS_Age_Sex_CI

gen id = _n

save Table3_Weighted_Age_Sex_W.dta, replace

restore

preserve

use Table3_Unweighted.dta, replace

merge 1:1 v1 using Table3_Weighted_IPW, nogen

merge 1:1 v1 using Table3_Weighted_Age_Sex_W.dta,

merge 1:1 v1 using Table3_n.dta, nogen

sort id

drop id

label var v1 "Indicator"

drop STEPS_IPW

gen indicator = _n

gen pop_group = 36

save Table3_36_Prevalence_GMAK_20Jul2022.dta, replace

restore

******************************************************************************

preserve

keep if age_cat == 0

keep if school_n == 0

tabout current_tobacco_any current_smoke_n current_smokeless_n ///

daily_tobacco_any daily_smoke_n daily_smokeless_n ///

smoke_only smokeless_only both_smoke_smokeless ///

alcohol_current no_fruit_veg no_fruit no_veg ///

atelessthan5 salt_eat_n salt_pack_n ///

htn_known htn_med file1 ///

using Table3_n.xls, ///

c(freq col) f(1c 1p) replace

import delimited using Table3_n.xls, clear

drop if v1 == "No"

drop if v1 == "Yes"

replace v1 = v1[_n - 1] if v1 == "Total"

drop v3 v5 v6 v7

drop if v1 == ""

drop if v2 == ""

destring v2 v4, ignore(",") replace

rename v2 IVR_n

rename v4 STEPS_n

gen id = _n

save Table3_n.dta, replace

restore

preserve

keep if age_cat == 0

keep if school_n == 0

gen wt = 1

svyset [pweight= wt]

tabout current_tobacco_any current_smoke_n current_smokeless_n ///

daily_tobacco_any daily_smoke_n daily_smokeless_n ///

smoke_only smokeless_only both_smoke_smokeless ///

alcohol_current no_fruit_veg no_fruit no_veg ///

atelessthan5 salt_eat_n salt_pack_n ///

htn_known htn_med file1 ///

using Table3_Unweight.xls, ///

c(col ci) f(1 1) percent svy replace

import delimited using Table3_Unweight.xls, clear

drop v6 v7

drop if v1 == "No" | v1 == "Total"

replace v1 = v1[_n - 1] if v1 == "Yes"

drop if v2 == ""

drop if v1 == ""

rename v2 IVR_Unweight

rename v4 STEPS_Unweight

rename v3 IVR_Unweight_CI

rename v5 STEPS_Unweight_CI

gen id = _n

save Table3_Unweighted.dta, replace

restore

********

preserve

keep if age_cat == 0

keep if school_n == 0

svyset [pweight= weight_ipw]

tabout current_tobacco_any current_smoke_n current_smokeless_n ///

daily_tobacco_any daily_smoke_n daily_smokeless_n ///

smoke_only smokeless_only both_smoke_smokeless ///

alcohol_current no_fruit_veg no_fruit no_veg ///

atelessthan5 salt_eat_n salt_pack_n ///

htn_known htn_med file1 ///

using Table3_Weight_IPW.xls, ///

c(col ci) f(1 1) percent svy replace

import delimited using Table3_Weight_IPW.xls, clear

drop v6 v7

drop if v1 == "No" | v1 == "Total"

replace v1 = v1[_n - 1] if v1 == "Yes"

drop if v2 == ""

drop if v1 == ""

rename v2 IVR_IPW

rename v4 STEPS_IPW

rename v3 IVR_IPW_CI

rename v5 STEPS_IPW_CI

gen id = _n

save Table3_Weighted_IPW.dta, replace

restore

********

preserve

keep if age_cat == 0

keep if school_n == 0

svyset [pweight= age_sex_w]

tabout current_tobacco_any current_smoke_n current_smokeless_n ///

daily_tobacco_any daily_smoke_n daily_smokeless_n ///

smoke_only smokeless_only both_smoke_smokeless ///

alcohol_current no_fruit_veg no_fruit no_veg ///

atelessthan5 salt_eat_n salt_pack_n ///

htn_known htn_med file1 ///

using Table3_Weight_Age_Sex_W.xls, ///

c(col ci) f(1 1) percent svy replace

import delimited using Table3_Weight_Age_Sex_W.xls, clear

drop v6 v7

drop if v1 == "No" | v1 == "Total"

replace v1 = v1[_n - 1] if v1 == "Yes"

drop if v2 == ""

drop if v1 == ""

rename v2 IVR_Age_Sex

rename v4 STEPS_Age_Sex

rename v3 IVR_Age_Sex_CI

rename v5 STEPS_Age_Sex_CI

gen id = _n

save Table3_Weighted_Age_Sex_W.dta, replace

restore

preserve

use Table3_Unweighted.dta, replace

merge 1:1 v1 using Table3_Weighted_IPW, nogen

merge 1:1 v1 using Table3_Weighted_Age_Sex_W.dta, nogen

merge 1:1 v1 using Table3_n.dta, nogen

sort id

drop id

label var v1 "Indicator"

drop STEPS_IPW

gen indicator = _n

gen pop_group = 37

save Table3_37_Prevalence_GMAK_20Jul2022.dta, replace

restore

******************************************************************************

preserve

keep if age_cat == 0

keep if school_n == 1

tabout current_tobacco_any current_smoke_n current_smokeless_n ///

daily_tobacco_any daily_smoke_n daily_smokeless_n ///

smoke_only smokeless_only both_smoke_smokeless ///

alcohol_current no_fruit_veg no_fruit no_veg ///

atelessthan5 salt_eat_n salt_pack_n ///

htn_known htn_med file1 ///

using Table3_n.xls, ///

c(freq col) f(1c 1p) replace

import delimited using Table3_n.xls, clear

drop if v1 == "No"

drop if v1 == "Yes"

replace v1 = v1[_n - 1] if v1 == "Total"

drop v3 v5 v6 v7

drop if v1 == ""

drop if v2 == ""

destring v2 v4, ignore(",") replace

rename v2 IVR_n

rename v4 STEPS_n

gen id = _n

save Table3_n.dta, replace

restore

preserve

keep if age_cat == 0

keep if school_n == 1

gen wt = 1

svyset [pweight= wt]

tabout current_tobacco_any current_smoke_n current_smokeless_n ///

daily_tobacco_any daily_smoke_n daily_smokeless_n ///

smoke_only smokeless_only both_smoke_smokeless ///

alcohol_current no_fruit_veg no_fruit no_veg ///

atelessthan5 salt_eat_n salt_pack_n ///

htn_known htn_med file1 ///

using Table3_Unweight.xls, ///

c(col ci) f(1 1) percent svy replace

import delimited using Table3_Unweight.xls, clear

drop v6 v7

drop if v1 == "No" | v1 == "Total"

replace v1 = v1[_n - 1] if v1 == "Yes"

drop if v2 == ""

drop if v1 == ""

rename v2 IVR_Unweight

rename v4 STEPS_Unweight

rename v3 IVR_Unweight_CI

rename v5 STEPS_Unweight_CI

gen id = _n

save Table3_Unweighted.dta, replace

restore

********

preserve

keep if age_cat == 0

keep if school_n == 1

svyset [pweight= weight_ipw]

tabout current_tobacco_any current_smoke_n current_smokeless_n ///

daily_tobacco_any daily_smoke_n daily_smokeless_n ///

smoke_only smokeless_only both_smoke_smokeless ///

alcohol_current no_fruit_veg no_fruit no_veg ///

atelessthan5 salt_eat_n salt_pack_n ///

htn_known htn_med file1 ///

using Table3_Weight_IPW.xls, ///

c(col ci) f(1 1) percent svy replace

import delimited using Table3_Weight_IPW.xls, clear

drop v6 v7

drop if v1 == "No" | v1 == "Total"

replace v1 = v1[_n - 1] if v1 == "Yes"

drop if v2 == ""

drop if v1 == ""

rename v2 IVR_IPW

rename v4 STEPS_IPW

rename v3 IVR_IPW_CI

rename v5 STEPS_IPW_CI

gen id = _n

save Table3_Weighted_IPW.dta, replace

restore

********

preserve

keep if age_cat == 0

keep if school_n == 1

svyset [pweight= age_sex_w]

tabout current_tobacco_any current_smoke_n current_smokeless_n ///

daily_tobacco_any daily_smoke_n daily_smokeless_n ///

smoke_only smokeless_only both_smoke_smokeless ///

alcohol_current no_fruit_veg no_fruit no_veg ///

atelessthan5 salt_eat_n salt_pack_n ///

htn_known htn_med file1 ///

using Table3_Weight_Age_Sex_W.xls, ///

c(col ci) f(1 1) percent svy replace

import delimited using Table3_Weight_Age_Sex_W.xls, clear

drop v6 v7

drop if v1 == "No" | v1 == "Total"

replace v1 = v1[_n - 1] if v1 == "Yes"

drop if v2 == ""

drop if v1 == ""

rename v2 IVR_Age_Sex

rename v4 STEPS_Age_Sex

rename v3 IVR_Age_Sex_CI

rename v5 STEPS_Age_Sex_CI

gen id = _n

save Table3_Weighted_Age_Sex_W.dta, replace

restore

preserve

use Table3_Unweighted.dta, replace

merge 1:1 v1 using Table3_Weighted_IPW, nogen

merge 1:1 v1 using Table3_Weighted_Age_Sex_W.dta, nogen

merge 1:1 v1 using Table3_n.dta, nogen

sort id

drop id

label var v1 "Indicator"

drop STEPS_IPW

gen indicator = _n

gen pop_group = 38

save Table3_38_Prevalence_GMAK_20Jul2022.dta, replace

restore

cap erase Table3_Unweighted.dta

cap erase Table3_Weighted_IPW.dta

cap erase Table3_Weighted_Age_Sex_W.dta

cap erase Table3_Weight_Age_Sex_W.xls

cap erase Table3_Weight_IPW.xls

cap erase Table3_Unweight.xls

******************************************************************************

preserve

keep if age_cat == 0

keep if school_n == 2

tabout current_tobacco_any current_smoke_n current_smokeless_n ///

daily_tobacco_any daily_smoke_n daily_smokeless_n ///

smoke_only smokeless_only both_smoke_smokeless ///

alcohol_current no_fruit_veg no_fruit no_veg ///

atelessthan5 salt_eat_n salt_pack_n ///

htn_known htn_med file1 ///

using Table3_n.xls, ///

c(freq col) f(1c 1p) replace

import delimited using Table3_n.xls, clear

drop if v1 == "No"

drop if v1 == "Yes"

replace v1 = v1[_n - 1] if v1 == "Total"

drop v3 v5 v6 v7

drop if v1 == ""

drop if v2 == ""

destring v2 v4, ignore(",") replace

rename v2 IVR_n

rename v4 STEPS_n

gen id = _n

save Table3_n.dta, replace

restore

preserve

keep if age_cat == 0

keep if school_n == 2

gen wt = 1

svyset [pweight= wt]

for var current_tobacco_any current_smoke_n current_smokeless_n ///

daily_tobacco_any daily_smoke_n daily_smokeless_n ///

smoke_only smokeless_only both_smoke_smokeless ///

alcohol_current no_fruit_veg no_fruit no_veg ///

atelessthan5 salt_eat_n salt_pack_n ///

htn_known htn_med: tab X file1

tabout current_tobacco_any current_smoke_n current_smokeless_n ///

daily_tobacco_any daily_smoke_n daily_smokeless_n ///

smoke_only smokeless_only no_fruit_veg no_fruit no_veg ///

atelessthan5 salt_eat_n salt_pack_n ///

htn_known htn_med file1 ///

using Table3_Unweight.xls, ///

c(col ci) f(1 1) percent svy replace

import delimited using Table3_Unweight.xls, clear

drop v6 v7

drop if v1 == "No" | v1 == "Total"

replace v1 = v1[_n - 1] if v1 == "Yes"

drop if v2 == ""

drop if v1 == ""

rename v2 IVR_Unweight

rename v4 STEPS_Unweight

rename v3 IVR_Unweight_CI

rename v5 STEPS_Unweight_CI

gen id = _n

save Table3_Unweighted.dta, replace

restore

********

preserve

keep if age_cat == 0

keep if school_n == 2

svyset [pweight= weight_ipw]

tabout current_tobacco_any current_smoke_n current_smokeless_n ///

daily_tobacco_any daily_smoke_n daily_smokeless_n ///

smoke_only smokeless_only no_fruit_veg no_fruit no_veg ///

atelessthan5 salt_eat_n salt_pack_n ///

htn_known htn_med file1 ///

using Table3_Weight_IPW.xls, ///

c(col ci) f(1 1) percent svy replace

import delimited using Table3_Weight_IPW.xls, clear

drop v6 v7

drop if v1 == "No" | v1 == "Total"

replace v1 = v1[_n - 1] if v1 == "Yes"

drop if v2 == ""

drop if v1 == ""

rename v2 IVR_IPW

rename v4 STEPS_IPW

rename v3 IVR_IPW_CI

rename v5 STEPS_IPW_CI

gen id = _n

save Table3_Weighted_IPW.dta, replace

restore

********

preserve

keep if age_cat == 0

keep if school_n == 2

svyset [pweight= age_sex_w]

tabout current_tobacco_any current_smoke_n current_smokeless_n ///

daily_tobacco_any daily_smoke_n daily_smokeless_n ///

smoke_only smokeless_only no_fruit_veg no_fruit no_veg ///

atelessthan5 salt_eat_n salt_pack_n ///

htn_known htn_med file1 ///

using Table3_Weight_Age_Sex_W.xls, ///

c(col ci) f(1 1) percent svy replace

import delimited using Table3_Weight_Age_Sex_W.xls, clear

drop v6 v7

drop if v1 == "No" | v1 == "Total"

replace v1 = v1[_n - 1] if v1 == "Yes"

drop if v2 == ""

drop if v1 == ""

rename v2 IVR_Age_Sex

rename v4 STEPS_Age_Sex

rename v3 IVR_Age_Sex_CI

rename v5 STEPS_Age_Sex_CI

gen id = _n

save Table3_Weighted_Age_Sex_W.dta, replace

restore

preserve

use Table3_Unweighted.dta, replace

merge 1:1 v1 using Table3_Weighted_IPW, nogen

merge 1:1 v1 using Table3_Weighted_Age_Sex_W.dta, nogen

merge 1:1 v1 using Table3_n.dta, nogen

sort id

drop id

label var v1 "Indicator"

drop STEPS_IPW

gen indicator = _n

gen pop_group = 39

save Table3_39_Prevalence_GMAK_20Jul2022.dta, replace

restore

cap erase Table3_Unweighted.dta

cap erase Table3_Weighted_IPW.dta

cap erase Table3_Weighted_Age_Sex_W.dta

cap erase Table3_Weight_Age_Sex_W.xls

cap erase Table3_Weight_IPW.xls

cap erase Table3_Unweight.xls

******************************************************************************

preserve

keep if age_cat == 1

keep if school_n == 0

tabout current_tobacco_any current_smoke_n current_smokeless_n ///

daily_tobacco_any daily_smoke_n daily_smokeless_n ///

smoke_only smokeless_only both_smoke_smokeless ///

alcohol_current no_fruit_veg no_fruit no_veg ///

atelessthan5 salt_eat_n salt_pack_n ///

htn_known htn_med file1 ///

using Table3_n.xls, ///

c(freq col) f(1c 1p) replace

import delimited using Table3_n.xls, clear

drop if v1 == "No"

drop if v1 == "Yes"

replace v1 = v1[_n - 1] if v1 == "Total"

drop v3 v5 v6 v7

drop if v1 == ""

drop if v2 == ""

destring v2 v4, ignore(",") replace

rename v2 IVR_n

rename v4 STEPS_n

gen id = _n

save Table3_n.dta, replace

restore

preserve

keep if age_cat == 1

keep if school_n == 0

gen wt = 1

svyset [pweight= wt]

tabout current_tobacco_any current_smoke_n current_smokeless_n ///

daily_tobacco_any daily_smoke_n daily_smokeless_n ///

smoke_only smokeless_only both_smoke_smokeless ///

alcohol_current no_fruit_veg no_fruit no_veg ///

atelessthan5 salt_eat_n salt_pack_n ///

htn_known htn_med file1 ///

using Table3_Unweight.xls, ///

c(col ci) f(1 1) percent svy replace

import delimited using Table3_Unweight.xls, clear

drop v6 v7

drop if v1 == "No" | v1 == "Total"

replace v1 = v1[_n - 1] if v1 == "Yes"

drop if v2 == ""

drop if v1 == ""

rename v2 IVR_Unweight

rename v4 STEPS_Unweight

rename v3 IVR_Unweight_CI

rename v5 STEPS_Unweight_CI

gen id = _n

save Table3_Unweighted.dta, replace

restore

********

preserve

keep if age_cat == 1

keep if school_n == 0

svyset [pweight= weight_ipw]

tabout current_tobacco_any current_smoke_n current_smokeless_n ///

daily_tobacco_any daily_smoke_n daily_smokeless_n ///

smoke_only smokeless_only both_smoke_smokeless ///

alcohol_current no_fruit_veg no_fruit no_veg ///

atelessthan5 salt_eat_n salt_pack_n ///

htn_known htn_med file1 ///

using Table3_Weight_IPW.xls, ///

c(col ci) f(1 1) percent svy replace

import delimited using Table3_Weight_IPW.xls, clear

drop v6 v7

drop if v1 == "No" | v1 == "Total"

replace v1 = v1[_n - 1] if v1 == "Yes"

drop if v2 == ""

drop if v1 == ""

rename v2 IVR_IPW

rename v4 STEPS_IPW

rename v3 IVR_IPW_CI

rename v5 STEPS_IPW_CI

gen id = _n

save Table3_Weighted_IPW.dta, replace

restore

********

preserve

keep if age_cat == 1

keep if school_n == 0

svyset [pweight= age_sex_w]

tabout current_tobacco_any current_smoke_n current_smokeless_n ///

daily_tobacco_any daily_smoke_n daily_smokeless_n ///

smoke_only smokeless_only both_smoke_smokeless ///

alcohol_current no_fruit_veg no_fruit no_veg ///

atelessthan5 salt_eat_n salt_pack_n ///

htn_known htn_med file1 ///

using Table3_Weight_Age_Sex_W.xls, ///

c(col ci) f(1 1) percent svy replace

import delimited using Table3_Weight_Age_Sex_W.xls, clear

drop v6 v7

drop if v1 == "No" | v1 == "Total"

replace v1 = v1[_n - 1] if v1 == "Yes"

drop if v2 == ""

drop if v1 == ""

rename v2 IVR_Age_Sex

rename v4 STEPS_Age_Sex

rename v3 IVR_Age_Sex_CI

rename v5 STEPS_Age_Sex_CI

gen id = _n

save Table3_Weighted_Age_Sex_W.dta, replace

restore

preserve

use Table3_Unweighted.dta, replace

merge 1:1 v1 using Table3_Weighted_IPW, nogen

merge 1:1 v1 using Table3_Weighted_Age_Sex_W.dta, nogen

merge 1:1 v1 using Table3_n.dta, nogen

sort id

drop id

label var v1 "Indicator"

drop STEPS_IPW

gen indicator = _n

gen pop_group = 40

save Table3_40_Prevalence_GMAK_20Jul2022.dta, replace

restore

******************************************************************************

preserve

keep if age_cat == 1

keep if school_n == 1

tabout current_tobacco_any current_smoke_n current_smokeless_n ///

daily_tobacco_any daily_smoke_n daily_smokeless_n ///

smoke_only smokeless_only both_smoke_smokeless ///

alcohol_current no_fruit_veg no_fruit no_veg ///

atelessthan5 salt_eat_n salt_pack_n ///

htn_known htn_med file1 ///

using Table3_n.xls, ///

c(freq col) f(1c 1p) replace

import delimited using Table3_n.xls, clear

drop if v1 == "No"

drop if v1 == "Yes"

replace v1 = v1[_n - 1] if v1 == "Total"

drop v3 v5 v6 v7

drop if v1 == ""

drop if v2 == ""

destring v2 v4, ignore(",") replace

rename v2 IVR_n

rename v4 STEPS_n

gen id = _n

save Table3_n.dta, replace

restore

preserve

keep if age_cat == 1

keep if school_n == 1

gen wt = 1

svyset [pweight= wt]

tabout current_tobacco_any current_smoke_n current_smokeless_n ///

daily_tobacco_any daily_smoke_n daily_smokeless_n ///

smoke_only smokeless_only both_smoke_smokeless ///

alcohol_current no_fruit_veg no_fruit no_veg ///

atelessthan5 salt_eat_n salt_pack_n ///

htn_known htn_med file1 ///

using Table3_Unweight.xls, ///

c(col ci) f(1 1) percent svy replace

import delimited using Table3_Unweight.xls, clear

drop v6 v7

drop if v1 == "No" | v1 == "Total"

replace v1 = v1[_n - 1] if v1 == "Yes"

drop if v2 == ""

drop if v1 == ""

rename v2 IVR_Unweight

rename v4 STEPS_Unweight

rename v3 IVR_Unweight_CI

rename v5 STEPS_Unweight_CI

gen id = _n

save Table3_Unweighted.dta, replace

restore

********

preserve

keep if age_cat == 1

keep if school_n == 1

svyset [pweight= weight_ipw]

tabout current_tobacco_any current_smoke_n current_smokeless_n ///

daily_tobacco_any daily_smoke_n daily_smokeless_n ///

smoke_only smokeless_only both_smoke_smokeless ///

alcohol_current no_fruit_veg no_fruit no_veg ///

atelessthan5 salt_eat_n salt_pack_n ///

htn_known htn_med file1 ///

using Table3_Weight_IPW.xls, ///

c(col ci) f(1 1) percent svy replace

import delimited using Table3_Weight_IPW.xls, clear

drop v6 v7

drop if v1 == "No" | v1 == "Total"

replace v1 = v1[_n - 1] if v1 == "Yes"

drop if v2 == ""

drop if v1 == ""

rename v2 IVR_IPW

rename v4 STEPS_IPW

rename v3 IVR_IPW_CI

rename v5 STEPS_IPW_CI

gen id = _n

save Table3_Weighted_IPW.dta, replace

restore

********

preserve

keep if age_cat == 1

keep if school_n == 1

svyset [pweight= age_sex_w]

tabout current_tobacco_any current_smoke_n current_smokeless_n ///

daily_tobacco_any daily_smoke_n daily_smokeless_n ///

smoke_only smokeless_only both_smoke_smokeless ///

alcohol_current no_fruit_veg no_fruit no_veg ///

atelessthan5 salt_eat_n salt_pack_n ///

htn_known htn_med file1 ///

using Table3_Weight_Age_Sex_W.xls, ///

c(col ci) f(1 1) percent svy replace

import delimited using Table3_Weight_Age_Sex_W.xls, clear

drop v6 v7

drop if v1 == "No" | v1 == "Total"

replace v1 = v1[_n - 1] if v1 == "Yes"

drop if v2 == ""

drop if v1 == ""

rename v2 IVR_Age_Sex

rename v4 STEPS_Age_Sex

rename v3 IVR_Age_Sex_CI

rename v5 STEPS_Age_Sex_CI

gen id = _n

save Table3_Weighted_Age_Sex_W.dta, replace

restore

preserve

use Table3_Unweighted.dta, replace

merge 1:1 v1 using Table3_Weighted_IPW, nogen

merge 1:1 v1 using Table3_Weighted_Age_Sex_W.dta, nogen

merge 1:1 v1 using Table3_n.dta, nogen

sort id

drop id

label var v1 "Indicator"

drop STEPS_IPW

gen indicator = _n

gen pop_group = 41

save Table3_41_Prevalence_GMAK_20Jul2022.dta, replace

restore

******************************************************************************

preserve

keep if age_cat == 1

keep if school_n == 2

tabout current_tobacco_any current_smoke_n current_smokeless_n ///

daily_tobacco_any daily_smoke_n daily_smokeless_n ///

smoke_only smokeless_only both_smoke_smokeless ///

alcohol_current no_fruit_veg no_fruit no_veg ///

atelessthan5 salt_eat_n salt_pack_n ///

htn_known htn_med file1 ///

using Table3_n.xls, ///

c(freq col) f(1c 1p) replace

import delimited using Table3_n.xls, clear

drop if v1 == "No"

drop if v1 == "Yes"

replace v1 = v1[_n - 1] if v1 == "Total"

drop v3 v5 v6 v7

drop if v1 == ""

drop if v2 == ""

destring v2 v4, ignore(",") replace

rename v2 IVR_n

rename v4 STEPS_n

gen id = _n

save Table3_n.dta, replace

restore

preserve

keep if age_cat == 1

keep if school_n == 2

gen wt = 1

svyset [pweight= wt]

tabout current_tobacco_any current_smoke_n current_smokeless_n ///

daily_tobacco_any daily_smoke_n daily_smokeless_n ///

smoke_only smokeless_only both_smoke_smokeless ///

alcohol_current no_fruit_veg no_fruit no_veg ///

atelessthan5 salt_eat_n salt_pack_n ///

htn_known htn_med file1 ///

using Table3_Unweight.xls, ///

c(col ci) f(1 1) percent svy replace

import delimited using Table3_Unweight.xls, clear

drop v6 v7

drop if v1 == "No" | v1 == "Total"

replace v1 = v1[_n - 1] if v1 == "Yes"

drop if v2 == ""

drop if v1 == ""

rename v2 IVR_Unweight

rename v4 STEPS_Unweight

rename v3 IVR_Unweight_CI

rename v5 STEPS_Unweight_CI

gen id = _n

save Table3_Unweighted.dta, replace

restore

********

preserve

keep if age_cat == 1

keep if school_n == 2

svyset [pweight= weight_ipw]

tabout current_tobacco_any current_smoke_n current_smokeless_n ///

daily_tobacco_any daily_smoke_n daily_smokeless_n ///

smoke_only smokeless_only both_smoke_smokeless ///

alcohol_current no_fruit_veg no_fruit no_veg ///

atelessthan5 salt_eat_n salt_pack_n ///

htn_known htn_med file1 ///

using Table3_Weight_IPW.xls, ///

c(col ci) f(1 1) percent svy replace

import delimited using Table3_Weight_IPW.xls, clear

drop v6 v7

drop if v1 == "No" | v1 == "Total"

replace v1 = v1[_n - 1] if v1 == "Yes"

drop if v2 == ""

drop if v1 == ""

rename v2 IVR_IPW

rename v4 STEPS_IPW

rename v3 IVR_IPW_CI

rename v5 STEPS_IPW_CI

gen id = _n

save Table3_Weighted_IPW.dta, replace

restore

********

preserve

keep if age_cat == 1

keep if school_n == 2

svyset [pweight= age_sex_w]

tabout current_tobacco_any current_smoke_n current_smokeless_n ///

daily_tobacco_any daily_smoke_n daily_smokeless_n ///

smoke_only smokeless_only both_smoke_smokeless ///

alcohol_current no_fruit_veg no_fruit no_veg ///

atelessthan5 salt_eat_n salt_pack_n ///

htn_known htn_med file1 ///

using Table3_Weight_Age_Sex_W.xls, ///

c(col ci) f(1 1) percent svy replace

import delimited using Table3_Weight_Age_Sex_W.xls, clear

drop v6 v7

drop if v1 == "No" | v1 == "Total"

replace v1 = v1[_n - 1] if v1 == "Yes"

drop if v2 == ""

drop if v1 == ""

rename v2 IVR_Age_Sex

rename v4 STEPS_Age_Sex

rename v3 IVR_Age_Sex_CI

rename v5 STEPS_Age_Sex_CI

gen id = _n

save Table3_Weighted_Age_Sex_W.dta, replace

restore

preserve

use Table3_Unweighted.dta, replace

merge 1:1 v1 using Table3_Weighted_IPW, nogen

merge 1:1 v1 using Table3_Weighted_Age_Sex_W.dta, nogen

merge 1:1 v1 using Table3_n.dta, nogen

sort id

drop id

label var v1 "Indicator"

drop STEPS_IPW

gen indicator = _n

gen pop_group = 42

save Table3_42_Prevalence_GMAK_20Jul2022.dta, replace

restore

******************************************************************************

preserve

keep if age_cat == 2

keep if school_n == 0

tabout current_tobacco_any current_smoke_n current_smokeless_n ///

daily_tobacco_any daily_smoke_n daily_smokeless_n ///

smoke_only smokeless_only both_smoke_smokeless ///

alcohol_current no_fruit_veg no_fruit no_veg ///

atelessthan5 salt_eat_n salt_pack_n ///

htn_known htn_med file1 ///

using Table3_n.xls, ///

c(freq col) f(1c 1p) replace

import delimited using Table3_n.xls, clear

drop if v1 == "No"

drop if v1 == "Yes"

replace v1 = v1[_n - 1] if v1 == "Total"

drop v3 v5 v6 v7

drop if v1 == ""

drop if v2 == ""

destring v2 v4, ignore(",") replace

rename v2 IVR_n

rename v4 STEPS_n

gen id = _n

save Table3_n.dta, replace

restore

preserve

keep if age_cat == 2

keep if school_n == 0

gen wt = 1

svyset [pweight= wt]

tabout current_tobacco_any current_smoke_n current_smokeless_n ///

daily_tobacco_any daily_smoke_n daily_smokeless_n ///

smoke_only smokeless_only both_smoke_smokeless ///

alcohol_current no_fruit_veg no_fruit no_veg ///

atelessthan5 salt_eat_n salt_pack_n ///

htn_known htn_med file1 ///

using Table3_Unweight.xls, ///

c(col ci) f(1 1) percent svy replace

import delimited using Table3_Unweight.xls, clear

drop v6 v7

drop if v1 == "No" | v1 == "Total"

replace v1 = v1[_n - 1] if v1 == "Yes"

drop if v2 == ""

drop if v1 == ""

rename v2 IVR_Unweight

rename v4 STEPS_Unweight

rename v3 IVR_Unweight_CI

rename v5 STEPS_Unweight_CI

gen id = _n

save Table3_Unweighted.dta, replace

restore

********

preserve

keep if age_cat == 2

keep if school_n == 0

svyset [pweight= weight_ipw]

tabout current_tobacco_any current_smoke_n current_smokeless_n ///

daily_tobacco_any daily_smoke_n daily_smokeless_n ///

smoke_only smokeless_only both_smoke_smokeless ///

alcohol_current no_fruit_veg no_fruit no_veg ///

atelessthan5 salt_eat_n salt_pack_n ///

htn_known htn_med file1 ///

using Table3_Weight_IPW.xls, ///

c(col ci) f(1 1) percent svy replace

import delimited using Table3_Weight_IPW.xls, clear

drop v6 v7

drop if v1 == "No" | v1 == "Total"

replace v1 = v1[_n - 1] if v1 == "Yes"

drop if v2 == ""

drop if v1 == ""

rename v2 IVR_IPW

rename v4 STEPS_IPW

rename v3 IVR_IPW_CI

rename v5 STEPS_IPW_CI

gen id = _n

save Table3_Weighted_IPW.dta, replace

restore

********

preserve

keep if age_cat == 2

keep if school_n == 0

svyset [pweight= age_sex_w]

tabout current_tobacco_any current_smoke_n current_smokeless_n ///

daily_tobacco_any daily_smoke_n daily_smokeless_n ///

smoke_only smokeless_only both_smoke_smokeless ///

alcohol_current no_fruit_veg no_fruit no_veg ///

atelessthan5 salt_eat_n salt_pack_n ///

htn_known htn_med file1 ///

using Table3_Weight_Age_Sex_W.xls, ///

c(col ci) f(1 1) percent svy replace

import delimited using Table3_Weight_Age_Sex_W.xls, clear

drop v6 v7

drop if v1 == "No" | v1 == "Total"

replace v1 = v1[_n - 1] if v1 == "Yes"

drop if v2 == ""

drop if v1 == ""

rename v2 IVR_Age_Sex

rename v4 STEPS_Age_Sex

rename v3 IVR_Age_Sex_CI

rename v5 STEPS_Age_Sex_CI

gen id = _n

save Table3_Weighted_Age_Sex_W.dta, replace

restore

preserve

use Table3_Unweighted.dta, replace

merge 1:1 v1 using Table3_Weighted_IPW, nogen

merge 1:1 v1 using Table3_Weighted_Age_Sex_W.dta, nogen

merge 1:1 v1 using Table3_n.dta, nogen

sort id

drop id

label var v1 "Indicator"

drop STEPS_IPW

gen indicator = _n

gen pop_group = 43

save Table3_43_Prevalence_GMAK_20Jul2022.dta, replace

restore

******************************************************************************

preserve

keep if age_cat == 2

keep if school_n == 1

tabout current_tobacco_any current_smoke_n current_smokeless_n ///

daily_tobacco_any daily_smoke_n daily_smokeless_n ///

smoke_only smokeless_only both_smoke_smokeless ///

alcohol_current no_fruit_veg no_fruit no_veg ///

atelessthan5 salt_eat_n salt_pack_n ///

htn_known htn_med file1 ///

using Table3_n.xls, ///

c(freq col) f(1c 1p) replace

import delimited using Table3_n.xls, clear

drop if v1 == "No"

drop if v1 == "Yes"

replace v1 = v1[_n - 1] if v1 == "Total"

drop v3 v5 v6 v7

drop if v1 == ""

drop if v2 == ""

destring v2 v4, ignore(",") replace

rename v2 IVR_n

rename v4 STEPS_n

gen id = _n

save Table3_n.dta, replace

restore

preserve

keep if age_cat == 2

keep if school_n == 1

gen wt = 1

svyset [pweight= wt]

tabout current_tobacco_any current_smoke_n current_smokeless_n ///

daily_tobacco_any daily_smoke_n daily_smokeless_n ///

smoke_only smokeless_only both_smoke_smokeless ///

alcohol_current no_fruit_veg no_fruit no_veg ///

atelessthan5 salt_eat_n salt_pack_n ///

htn_known htn_med file1 ///

using Table3_Unweight.xls, ///

c(col ci) f(1 1) percent svy replace

import delimited using Table3_Unweight.xls, clear

drop v6 v7

drop if v1 == "No" | v1 == "Total"

replace v1 = v1[_n - 1] if v1 == "Yes"

drop if v2 == ""

drop if v1 == ""

rename v2 IVR_Unweight

rename v4 STEPS_Unweight

rename v3 IVR_Unweight_CI

rename v5 STEPS_Unweight_CI

gen id = _n

save Table3_Unweighted.dta, replace

restore

********

preserve

keep if age_cat == 2

keep if school_n == 1

svyset [pweight= weight_ipw]

tabout current_tobacco_any current_smoke_n current_smokeless_n ///

daily_tobacco_any daily_smoke_n daily_smokeless_n ///

smoke_only smokeless_only both_smoke_smokeless ///

alcohol_current no_fruit_veg no_fruit no_veg ///

atelessthan5 salt_eat_n salt_pack_n ///

htn_known htn_med file1 ///

using Table3_Weight_IPW.xls, ///

c(col ci) f(1 1) percent svy replace

import delimited using Table3_Weight_IPW.xls, clear

drop v6 v7

drop if v1 == "No" | v1 == "Total"

replace v1 = v1[_n - 1] if v1 == "Yes"

drop if v2 == ""

drop if v1 == ""

rename v2 IVR_IPW

rename v4 STEPS_IPW

rename v3 IVR_IPW_CI

rename v5 STEPS_IPW_CI

gen id = _n

save Table3_Weighted_IPW.dta, replace

restore

********

preserve

keep if age_cat == 2

keep if school_n == 1

svyset [pweight= age_sex_w]

tabout current_tobacco_any current_smoke_n current_smokeless_n ///

daily_tobacco_any daily_smoke_n daily_smokeless_n ///

smoke_only smokeless_only both_smoke_smokeless ///

alcohol_current no_fruit_veg no_fruit no_veg ///

atelessthan5 salt_eat_n salt_pack_n ///

htn_known htn_med file1 ///

using Table3_Weight_Age_Sex_W.xls, ///

c(col ci) f(1 1) percent svy replace

import delimited using Table3_Weight_Age_Sex_W.xls, clear

drop v6 v7

drop if v1 == "No" | v1 == "Total"

replace v1 = v1[_n - 1] if v1 == "Yes"

drop if v2 == ""

drop if v1 == ""

rename v2 IVR_Age_Sex

rename v4 STEPS_Age_Sex

rename v3 IVR_Age_Sex_CI

rename v5 STEPS_Age_Sex_CI

gen id = _n

save Table3_Weighted_Age_Sex_W.dta, replace

restore

preserve

use Table3_Unweighted.dta, replace

merge 1:1 v1 using Table3_Weighted_IPW, nogen

merge 1:1 v1 using Table3_Weighted_Age_Sex_W.dta, nogen

merge 1:1 v1 using Table3_n.dta, nogen

sort id

drop id

label var v1 "Indicator"

drop STEPS_IPW

gen indicator = _n

gen pop_group = 44

save Table3_44_Prevalence_GMAK_20Jul2022.dta, replace

restore

cap erase Table3_Unweighted.dta

cap erase Table3_Weighted_IPW.dta

cap erase Table3_Weighted_Age_Sex_W.dta

cap erase Table3_Weight_Age_Sex_W.xls

cap erase Table3_Weight_IPW.xls

cap erase Table3_Unweight.xls

******************************************************************************

preserve

keep if age_cat == 2

keep if school_n == 2

tabout current_tobacco_any current_smoke_n current_smokeless_n ///

daily_tobacco_any daily_smoke_n daily_smokeless_n ///

smoke_only smokeless_only both_smoke_smokeless ///

alcohol_current no_fruit_veg no_fruit no_veg ///

atelessthan5 salt_eat_n salt_pack_n ///

htn_known htn_med file1 ///

using Table3_n.xls, ///

c(freq col) f(1c 1p) replace

import delimited using Table3_n.xls, clear

drop if v1 == "No"

drop if v1 == "Yes"

replace v1 = v1[_n - 1] if v1 == "Total"

drop v3 v5 v6 v7

drop if v1 == ""

drop if v2 == ""

destring v2 v4, ignore(",") replace

rename v2 IVR_n

rename v4 STEPS_n

gen id = _n

save Table3_n.dta, replace

restore

preserve

keep if age_cat == 2

keep if school_n == 2

gen wt = 1

svyset [pweight= wt]

tabout current_tobacco_any current_smoke_n current_smokeless_n ///

daily_tobacco_any daily_smoke_n daily_smokeless_n ///

smoke_only smokeless_only both_smoke_smokeless ///

alcohol_current no_fruit_veg no_fruit no_veg ///

atelessthan5 salt_eat_n salt_pack_n ///

htn_known htn_med file1 ///

using Table3_Unweight.xls, ///

c(col ci) f(1 1) percent svy replace

import delimited using Table3_Unweight.xls, clear

drop v6 v7

drop if v1 == "No" | v1 == "Total"

replace v1 = v1[_n - 1] if v1 == "Yes"

drop if v2 == ""

drop if v1 == ""

rename v2 IVR_Unweight

rename v4 STEPS_Unweight

rename v3 IVR_Unweight_CI

rename v5 STEPS_Unweight_CI

gen id = _n

save Table3_Unweighted.dta, replace

restore

********

preserve

keep if age_cat == 2

keep if school_n == 2

svyset [pweight= weight_ipw]

tabout current_tobacco_any current_smoke_n current_smokeless_n ///

daily_tobacco_any daily_smoke_n daily_smokeless_n ///

smoke_only smokeless_only both_smoke_smokeless ///

alcohol_current no_fruit_veg no_fruit no_veg ///

atelessthan5 salt_eat_n salt_pack_n ///

htn_known htn_med file1 ///

using Table3_Weight_IPW.xls, ///

c(col ci) f(1 1) percent svy replace

import delimited using Table3_Weight_IPW.xls, clear

drop v6 v7

drop if v1 == "No" | v1 == "Total"

replace v1 = v1[_n - 1] if v1 == "Yes"

drop if v2 == ""

drop if v1 == ""

rename v2 IVR_IPW

rename v4 STEPS_IPW

rename v3 IVR_IPW_CI

rename v5 STEPS_IPW_CI

gen id = _n

save Table3_Weighted_IPW.dta, replace

restore

********

preserve

keep if age_cat == 2

keep if school_n == 2

svyset [pweight= age_sex_w]

tabout current_tobacco_any current_smoke_n current_smokeless_n ///

daily_tobacco_any daily_smoke_n daily_smokeless_n ///

smoke_only smokeless_only both_smoke_smokeless ///

alcohol_current no_fruit_veg no_fruit no_veg ///

atelessthan5 salt_eat_n salt_pack_n ///

htn_known htn_med file1 ///

using Table3_Weight_Age_Sex_W.xls, ///

c(col ci) f(1 1) percent svy replace

import delimited using Table3_Weight_Age_Sex_W.xls, clear

drop v6 v7

drop if v1 == "No" | v1 == "Total"

replace v1 = v1[_n - 1] if v1 == "Yes"

drop if v2 == ""

drop if v1 == ""

rename v2 IVR_Age_Sex

rename v4 STEPS_Age_Sex

rename v3 IVR_Age_Sex_CI

rename v5 STEPS_Age_Sex_CI

gen id = _n

save Table3_Weighted_Age_Sex_W.dta, replace

restore

preserve

use Table3_Unweighted.dta, replace

merge 1:1 v1 using Table3_Weighted_IPW, nogen

merge 1:1 v1 using Table3_Weighted_Age_Sex_W.dta, nogen

merge 1:1 v1 using Table3_n.dta, nogen

sort id

drop id

label var v1 "Indicator"

drop STEPS_IPW

gen indicator = _n

gen pop_group = 45

save Table3_45_Prevalence_GMAK_20Jul2022.dta, replace

restore

******************************************************************************

preserve

keep if school_n == 0

tabout current_tobacco_any current_smoke_n current_smokeless_n ///

daily_tobacco_any daily_smoke_n daily_smokeless_n ///

smoke_only smokeless_only both_smoke_smokeless ///

alcohol_current no_fruit_veg no_fruit no_veg ///

atelessthan5 salt_eat_n salt_pack_n ///

htn_known htn_med file1 ///

using Table3_n.xls, ///

c(freq col) f(1c 1p) replace

import delimited using Table3_n.xls, clear

drop if v1 == "No"

drop if v1 == "Yes"

replace v1 = v1[_n - 1] if v1 == "Total"

drop v3 v5 v6 v7

drop if v1 == ""

drop if v2 == ""

destring v2 v4, ignore(",") replace

rename v2 IVR_n

rename v4 STEPS_n

gen id = _n

save Table3_n.dta, replace

restore

preserve

keep if school_n == 0

gen wt = 1

svyset [pweight= wt]

tabout current_tobacco_any current_smoke_n current_smokeless_n ///

daily_tobacco_any daily_smoke_n daily_smokeless_n ///

smoke_only smokeless_only both_smoke_smokeless ///

alcohol_current no_fruit_veg no_fruit no_veg ///

atelessthan5 salt_eat_n salt_pack_n ///

htn_known htn_med file1 ///

using Table3_Unweight.xls, ///

c(col ci) f(1 1) percent svy replace

import delimited using Table3_Unweight.xls, clear

drop v6 v7

drop if v1 == "No" | v1 == "Total"

replace v1 = v1[_n - 1] if v1 == "Yes"

drop if v2 == ""

drop if v1 == ""

rename v2 IVR_Unweight

rename v4 STEPS_Unweight

rename v3 IVR_Unweight_CI

rename v5 STEPS_Unweight_CI

gen id = _n

save Table3_Unweighted.dta, replace

restore

********

preserve

keep if school_n == 0

svyset [pweight= weight_ipw]

tabout current_tobacco_any current_smoke_n current_smokeless_n ///

daily_tobacco_any daily_smoke_n daily_smokeless_n ///

smoke_only smokeless_only both_smoke_smokeless ///

alcohol_current no_fruit_veg no_fruit no_veg ///

atelessthan5 salt_eat_n salt_pack_n ///

htn_known htn_med file1 ///

using Table3_Weight_IPW.xls, ///

c(col ci) f(1 1) percent svy replace

import delimited using Table3_Weight_IPW.xls, clear

drop v6 v7

drop if v1 == "No" | v1 == "Total"

replace v1 = v1[_n - 1] if v1 == "Yes"

drop if v2 == ""

drop if v1 == ""

rename v2 IVR_IPW

rename v4 STEPS_IPW

rename v3 IVR_IPW_CI

rename v5 STEPS_IPW_CI

gen id = _n

save Table3_Weighted_IPW.dta, replace

restore

********

preserve

keep if school_n == 0

svyset [pweight= age_sex_w]

tabout current_tobacco_any current_smoke_n current_smokeless_n ///

daily_tobacco_any daily_smoke_n daily_smokeless_n ///

smoke_only smokeless_only both_smoke_smokeless ///

alcohol_current no_fruit_veg no_fruit no_veg ///

atelessthan5 salt_eat_n salt_pack_n ///

htn_known htn_med file1 ///

using Table3_Weight_Age_Sex_W.xls, ///

c(col ci) f(1 1) percent svy replace

import delimited using Table3_Weight_Age_Sex_W.xls, clear

drop v6 v7

drop if v1 == "No" | v1 == "Total"

replace v1 = v1[_n - 1] if v1 == "Yes"

drop if v2 == ""

drop if v1 == ""

rename v2 IVR_Age_Sex

rename v4 STEPS_Age_Sex

rename v3 IVR_Age_Sex_CI

rename v5 STEPS_Age_Sex_CI

gen id = _n

save Table3_Weighted_Age_Sex_W.dta, replace

restore

preserve

use Table3_Unweighted.dta, replace

merge 1:1 v1 using Table3_Weighted_IPW, nogen

merge 1:1 v1 using Table3_Weighted_Age_Sex_W.dta, nogen

merge 1:1 v1 using Table3_n.dta, nogen

sort id

drop id

label var v1 "Indicator"

drop STEPS_IPW

gen indicator = _n

gen pop_group = 46

save Table3_46_Prevalence_GMAK_20Jul2022.dta, replace

restore

******************************************************************************

preserve

keep if school_n == 1

tabout current_tobacco_any current_smoke_n current_smokeless_n ///

daily_tobacco_any daily_smoke_n daily_smokeless_n ///

smoke_only smokeless_only both_smoke_smokeless ///

alcohol_current no_fruit_veg no_fruit no_veg ///

atelessthan5 salt_eat_n salt_pack_n ///

htn_known htn_med file1 ///

using Table3_n.xls, ///

c(freq col) f(1c 1p) replace

import delimited using Table3_n.xls, clear

drop if v1 == "No"

drop if v1 == "Yes"

replace v1 = v1[_n - 1] if v1 == "Total"

drop v3 v5 v6 v7

drop if v1 == ""

drop if v2 == ""

destring v2 v4, ignore(",") replace

rename v2 IVR_n

rename v4 STEPS_n

gen id = _n

save Table3_n.dta, replace

restore

preserve

keep if school_n == 1

gen wt = 1

svyset [pweight= wt]

tabout current_tobacco_any current_smoke_n current_smokeless_n ///

daily_tobacco_any daily_smoke_n daily_smokeless_n ///

smoke_only smokeless_only both_smoke_smokeless ///

alcohol_current no_fruit_veg no_fruit no_veg ///

atelessthan5 salt_eat_n salt_pack_n ///

htn_known htn_med file1 ///

using Table3_Unweight.xls, ///

c(col ci) f(1 1) percent svy replace

import delimited using Table3_Unweight.xls, clear

drop v6 v7

drop if v1 == "No" | v1 == "Total"

replace v1 = v1[_n - 1] if v1 == "Yes"

drop if v2 == ""

drop if v1 == ""

rename v2 IVR_Unweight

rename v4 STEPS_Unweight

rename v3 IVR_Unweight_CI

rename v5 STEPS_Unweight_CI

gen id = _n

save Table3_Unweighted.dta, replace

restore

********

preserve

keep if school_n == 1

svyset [pweight= weight_ipw]

tabout current_tobacco_any current_smoke_n current_smokeless_n ///

daily_tobacco_any daily_smoke_n daily_smokeless_n ///

smoke_only smokeless_only both_smoke_smokeless ///

alcohol_current no_fruit_veg no_fruit no_veg ///

atelessthan5 salt_eat_n salt_pack_n ///

htn_known htn_med file1 ///

using Table3_Weight_IPW.xls, ///

c(col ci) f(1 1) percent svy replace

import delimited using Table3_Weight_IPW.xls, clear

drop v6 v7

drop if v1 == "No" | v1 == "Total"

replace v1 = v1[_n - 1] if v1 == "Yes"

drop if v2 == ""

drop if v1 == ""

rename v2 IVR_IPW

rename v4 STEPS_IPW

rename v3 IVR_IPW_CI

rename v5 STEPS_IPW_CI

gen id = _n

save Table3_Weighted_IPW.dta, replace

restore

********

preserve

keep if school_n == 1

svyset [pweight= age_sex_w]

tabout current_tobacco_any current_smoke_n current_smokeless_n ///

daily_tobacco_any daily_smoke_n daily_smokeless_n ///

smoke_only smokeless_only both_smoke_smokeless ///

alcohol_current no_fruit_veg no_fruit no_veg ///

atelessthan5 salt_eat_n salt_pack_n ///

htn_known htn_med file1 ///

using Table3_Weight_Age_Sex_W.xls, ///

c(col ci) f(1 1) percent svy replace

import delimited using Table3_Weight_Age_Sex_W.xls, clear

drop v6 v7

drop if v1 == "No" | v1 == "Total"

replace v1 = v1[_n - 1] if v1 == "Yes"

drop if v2 == ""

drop if v1 == ""

rename v2 IVR_Age_Sex

rename v4 STEPS_Age_Sex

rename v3 IVR_Age_Sex_CI

rename v5 STEPS_Age_Sex_CI

gen id = _n

save Table3_Weighted_Age_Sex_W.dta, replace

restore

preserve

use Table3_Unweighted.dta, replace

merge 1:1 v1 using Table3_Weighted_IPW, nogen

merge 1:1 v1 using Table3_Weighted_Age_Sex_W.dta, nogen

merge 1:1 v1 using Table3_n.dta, nogen

sort id

drop id

label var v1 "Indicator"

drop STEPS_IPW

gen indicator = _n

gen pop_group = 47

save Table3_47_Prevalence_GMAK_20Jul2022.dta, replace

restore

******************************************************************************

preserve

keep if school_n == 2

tabout current_tobacco_any current_smoke_n current_smokeless_n ///

daily_tobacco_any daily_smoke_n daily_smokeless_n ///

smoke_only smokeless_only both_smoke_smokeless ///

alcohol_current no_fruit_veg no_fruit no_veg ///

atelessthan5 salt_eat_n salt_pack_n ///

htn_known htn_med file1 ///

using Table3_n.xls, ///

c(freq col) f(1c 1p) replace

import delimited using Table3_n.xls, clear

drop if v1 == "No"

drop if v1 == "Yes"

replace v1 = v1[_n - 1] if v1 == "Total"

drop v3 v5 v6 v7

drop if v1 == ""

drop if v2 == ""

destring v2 v4, ignore(",") replace

rename v2 IVR_n

rename v4 STEPS_n

gen id = _n

save Table3_n.dta, replace

restore

preserve

keep if school_n == 2

gen wt = 1

svyset [pweight= wt]

tabout current_tobacco_any current_smoke_n current_smokeless_n ///

daily_tobacco_any daily_smoke_n daily_smokeless_n ///

smoke_only smokeless_only both_smoke_smokeless ///

alcohol_current no_fruit_veg no_fruit no_veg ///

atelessthan5 salt_eat_n salt_pack_n ///

htn_known htn_med file1 ///

using Table3_Unweight.xls, ///

c(col ci) f(1 1) percent svy replace

import delimited using Table3_Unweight.xls, clear

drop v6 v7

drop if v1 == "No" | v1 == "Total"

replace v1 = v1[_n - 1] if v1 == "Yes"

drop if v2 == ""

drop if v1 == ""

rename v2 IVR_Unweight

rename v4 STEPS_Unweight

rename v3 IVR_Unweight_CI

rename v5 STEPS_Unweight_CI

gen id = _n

save Table3_Unweighted.dta, replace

restore

********

preserve

keep if school_n == 2

svyset [pweight= weight_ipw]

tabout current_tobacco_any current_smoke_n current_smokeless_n ///

daily_tobacco_any daily_smoke_n daily_smokeless_n ///

smoke_only smokeless_only both_smoke_smokeless ///

alcohol_current no_fruit_veg no_fruit no_veg ///

atelessthan5 salt_eat_n salt_pack_n ///

htn_known htn_med file1 ///

using Table3_Weight_IPW.xls, ///

c(col ci) f(1 1) percent svy replace

import delimited using Table3_Weight_IPW.xls, clear

drop v6 v7

drop if v1 == "No" | v1 == "Total"

replace v1 = v1[_n - 1] if v1 == "Yes"

drop if v2 == ""

drop if v1 == ""

rename v2 IVR_IPW

rename v4 STEPS_IPW

rename v3 IVR_IPW_CI

rename v5 STEPS_IPW_CI

gen id = _n

save Table3_Weighted_IPW.dta, replace

restore

********

preserve

keep if school_n == 2

svyset [pweight= age_sex_w]

tabout current_tobacco_any current_smoke_n current_smokeless_n ///

daily_tobacco_any daily_smoke_n daily_smokeless_n ///

smoke_only smokeless_only both_smoke_smokeless ///

alcohol_current no_fruit_veg no_fruit no_veg ///

atelessthan5 salt_eat_n salt_pack_n ///

htn_known htn_med file1 ///

using Table3_Weight_Age_Sex_W.xls, ///

c(col ci) f(1 1) percent svy replace

import delimited using Table3_Weight_Age_Sex_W.xls, clear

drop v6 v7

drop if v1 == "No" | v1 == "Total"

replace v1 = v1[_n - 1] if v1 == "Yes"

drop if v2 == ""

drop if v1 == ""

rename v2 IVR_Age_Sex

rename v4 STEPS_Age_Sex

rename v3 IVR_Age_Sex_CI

rename v5 STEPS_Age_Sex_CI

gen id = _n

save Table3_Weighted_Age_Sex_W.dta, replace

restore

preserve

use Table3_Unweighted.dta, replace

merge 1:1 v1 using Table3_Weighted_IPW, nogen

merge 1:1 v1 using Table3_Weighted_Age_Sex_W.dta, nogen

merge 1:1 v1 using Table3_n.dta, nogen

sort id

drop id

label var v1 "Indicator"

drop STEPS_IPW

gen indicator = _n

gen pop_group = 48

save Table3_48_Prevalence_GMAK_20Jul2022.dta, replace

restore

cap erase Table3_Unweighted.dta

cap erase Table3_Weighted_IPW.dta

cap erase Table3_Weighted_Age_Sex_W.dta

cap erase Table3_Weight_Age_Sex_W.xls

cap erase Table3_Weight_IPW.xls

cap erase Table3_Unweight.xls

********************************************************************************

preserve

recode file1 (1 = 0) (0 = 1)

keep if age_cat == 0

svyset [pweight= weight_ipw]

gen file2 = file1

gen file3 = file1

gen file4 = file1

gen file5 = file1

gen file6 = file1

gen file7 = file1

gen file8 = file1

gen file9 = file1

gen file10 = file1

gen file11 = file1

gen file12 = file1

gen file13 = file1

gen file14 = file1

gen file15 = file1

gen file16 = file1

gen file17 = file1

gen file18 = file1

svy: glm current_tobacco_any file1, fam(bin) link(identity)

estimates store I1

svy: glm current_smoke_n file2, fam(bin) link(identity)

estimates store I2

svy: glm current_smokeless_n file3, fam(bin) link(identity)

estimates store I3

svy: glm daily_tobacco_any file4, fam(bin) link(identity)

estimates store I4

svy: glm daily_smoke_n file5, fam(bin) link(identity)

estimates store I5

svy: glm daily_smokeless_n file6, fam(bin) link(identity)

estimates store I6

svy: glm smoke_only file7, fam(bin) link(identity)

estimates store I7

svy: glm smokeless_only file8, fam(bin) link(identity)

estimates store I8

svy: glm both_smoke_smokeless file9, fam(bin) link(identity)

estimates store I9

svy: glm alcohol_current file10, fam(bin) link(identity)

estimates store I10

svy: glm no_fruit_veg file11, fam(bin) link(identity)

estimates store I11

svy: glm no_fruit file12, fam(bin) link(identity)

estimates store I12

svy: glm no_veg file13, fam(bin) link(identity)

estimates store I13

svy: glm atelessthan5 file14, fam(bin) link(identity)

estimates store I14

svy: glm salt_eat_n file15, fam(bin) link(identity)

estimates store I15

svy: glm salt_pack_n file16, fam(bin) link(identity)

estimates store I16

svy: glm htn_known file17, fam(bin) link(identity)

estimates store I17

svy: glm htn_med file18, fam(bin) link(identity)

estimates store I18

estout I1 I2 I3 I4 I5 I6 I7 I8 I9 I10 I11 I12 I13 I14 I15 I16 I17 I18 using Table_RD.xls, ///

cells ("b(star fmt(1)) ci(par fmt(1) label(95% CI)) p (fmt(4)) ") replace transform(@*100)

import delimited "Table_RD.xls", clear

replace v2 = v5 if v2 == ""

replace v2 = v8 if v2 == ""

replace v2 = v11 if v2 == ""

replace v2 = v14 if v2 == ""

replace v2 = v17 if v2 == ""

replace v2 = v20 if v2 == ""

replace v2 = v23 if v2 == ""

replace v2 = v26 if v2 == ""

replace v2 = v29 if v2 == ""

replace v2 = v32 if v2 == ""

replace v2 = v35 if v2 == ""

replace v2 = v38 if v2 == ""

replace v2 = v41 if v2 == ""

replace v2 = v44 if v2 == ""

replace v2 = v47 if v2 == ""

replace v2 = v50 if v2 == ""

replace v2 = v53 if v2 == ""

replace v3 = v6 if v3 == ""

replace v3 = v9 if v3 == ""

replace v3 = v12 if v3 == ""

replace v3 = v15 if v3 == ""

replace v3 = v18 if v3 == ""

replace v3 = v21 if v3 == ""

replace v3 = v24 if v3 == ""

replace v3 = v27 if v3 == ""

replace v3 = v30 if v3 == ""

replace v3 = v33 if v3 == ""

replace v3 = v36 if v3 == ""

replace v3 = v39 if v3 == ""

replace v3 = v42 if v3 == ""

replace v3 = v45 if v3 == ""

replace v3 = v48 if v3 == ""

replace v3 = v51 if v3 == ""

replace v3 = v54 if v3 == ""

replace v4 = v7 if v4 == ""

replace v4 = v10 if v4 == ""

replace v4 = v13 if v4 == ""

replace v4 = v16 if v4 == ""

replace v4 = v19 if v4 == ""

replace v4 = v22 if v4 == ""

replace v4 = v25 if v4 == ""

replace v4 = v28 if v4 == ""

replace v4 = v31 if v4 == ""

replace v4 = v34 if v4 == ""

replace v4 = v37 if v4 == ""

replace v4 = v40 if v4 == ""

replace v4 = v43 if v4 == ""

replace v4 = v46 if v4 == ""

replace v4 = v49 if v4 == ""

replace v4 = v52 if v4 == ""

replace v4 = v55 if v4 == ""

keep v1 v2 v3 v4

drop if v1 == ""

drop if v1 == "main"

drop if v1 == "_cons"

destring v1, replace ignore(file)

rename v1 indicator

rename v2 Diff_IVR_IPW

rename v3 Diff_IVR_IPW_CI

rename v4 Diff_IVR_IPW_P

gen pop_group = 1

save File_Diff_IVR_IPW_1, replace

restore

********

preserve

recode file1 (1 = 0) (0 = 1)

keep if age_cat == 1

svyset [pweight= weight_ipw]

gen file2 = file1

gen file3 = file1

gen file4 = file1

gen file5 = file1

gen file6 = file1

gen file7 = file1

gen file8 = file1

gen file9 = file1

gen file10 = file1

gen file11 = file1

gen file12 = file1

gen file13 = file1

gen file14 = file1

gen file15 = file1

gen file16 = file1

gen file17 = file1

gen file18 = file1

svy: glm current_tobacco_any file1, fam(bin) link(identity)

estimates store I1

svy: glm current_smoke_n file2, fam(bin) link(identity)

estimates store I2

svy: glm current_smokeless_n file3, fam(bin) link(identity)

estimates store I3

svy: glm daily_tobacco_any file4, fam(bin) link(identity)

estimates store I4

svy: glm daily_smoke_n file5, fam(bin) link(identity)

estimates store I5

svy: glm daily_smokeless_n file6, fam(bin) link(identity)

estimates store I6

svy: glm smoke_only file7, fam(bin) link(identity)

estimates store I7

svy: glm smokeless_only file8, fam(bin) link(identity)

estimates store I8

svy: glm both_smoke_smokeless file9, fam(bin) link(identity)

estimates store I9

svy: glm alcohol_current file10, fam(bin) link(identity)

estimates store I10

svy: glm no_fruit_veg file11, fam(bin) link(identity)

estimates store I11

svy: glm no_fruit file12, fam(bin) link(identity)

estimates store I12

svy: glm no_veg file13, fam(bin) link(identity)

estimates store I13

svy: glm atelessthan5 file14, fam(bin) link(identity)

estimates store I14

svy: glm salt_eat_n file15, fam(bin) link(identity)

estimates store I15

svy: glm salt_pack_n file16, fam(bin) link(identity)

estimates store I16

svy: glm htn_known file17, fam(bin) link(identity)

estimates store I17

svy: glm htn_med file18, fam(bin) link(identity)

estimates store I18

estout I1 I2 I3 I4 I5 I6 I7 I8 I9 I10 I11 I12 I13 I14 I15 I16 I17 I18 using Table_RD.xls, ///

cells ("b(star fmt(1)) ci(par fmt(1) label(95% CI)) p (fmt(4)) ") replace transform(@*100)

import delimited "Table_RD.xls", clear

replace v2 = v5 if v2 == ""

replace v2 = v8 if v2 == ""

replace v2 = v11 if v2 == ""

replace v2 = v14 if v2 == ""

replace v2 = v17 if v2 == ""

replace v2 = v20 if v2 == ""

replace v2 = v23 if v2 == ""

replace v2 = v26 if v2 == ""

replace v2 = v29 if v2 == ""

replace v2 = v32 if v2 == ""

replace v2 = v35 if v2 == ""

replace v2 = v38 if v2 == ""

replace v2 = v41 if v2 == ""

replace v2 = v44 if v2 == ""

replace v2 = v47 if v2 == ""

replace v2 = v50 if v2 == ""

replace v2 = v53 if v2 == ""

replace v3 = v6 if v3 == ""

replace v3 = v9 if v3 == ""

replace v3 = v12 if v3 == ""

replace v3 = v15 if v3 == ""

replace v3 = v18 if v3 == ""

replace v3 = v21 if v3 == ""

replace v3 = v24 if v3 == ""

replace v3 = v27 if v3 == ""

replace v3 = v30 if v3 == ""

replace v3 = v33 if v3 == ""

replace v3 = v36 if v3 == ""

replace v3 = v39 if v3 == ""

replace v3 = v42 if v3 == ""

replace v3 = v45 if v3 == ""

replace v3 = v48 if v3 == ""

replace v3 = v51 if v3 == ""

replace v3 = v54 if v3 == ""

replace v4 = v7 if v4 == ""

replace v4 = v10 if v4 == ""

replace v4 = v13 if v4 == ""

replace v4 = v16 if v4 == ""

replace v4 = v19 if v4 == ""

replace v4 = v22 if v4 == ""

replace v4 = v25 if v4 == ""

replace v4 = v28 if v4 == ""

replace v4 = v31 if v4 == ""

replace v4 = v34 if v4 == ""

replace v4 = v37 if v4 == ""

replace v4 = v40 if v4 == ""

replace v4 = v43 if v4 == ""

replace v4 = v46 if v4 == ""

replace v4 = v49 if v4 == ""

replace v4 = v52 if v4 == ""

replace v4 = v55 if v4 == ""

keep v1 v2 v3 v4

drop if v1 == ""

drop if v1 == "main"

drop if v1 == "_cons"

destring v1, replace ignore(file)

rename v1 indicator

rename v2 Diff_IVR_IPW

rename v3 Diff_IVR_IPW_CI

rename v4 Diff_IVR_IPW_P

gen pop_group = 2

save File_Diff_IVR_IPW_2, replace

restore

********

preserve

recode file1 (1 = 0) (0 = 1)

keep if age_cat == 2

svyset [pweight= weight_ipw]

gen file2 = file1

gen file3 = file1

gen file4 = file1

gen file5 = file1

gen file6 = file1

gen file7 = file1

gen file8 = file1

gen file9 = file1

gen file10 = file1

gen file11 = file1

gen file12 = file1

gen file13 = file1

gen file14 = file1

gen file15 = file1

gen file16 = file1

gen file17 = file1

gen file18 = file1

svy: glm current_tobacco_any file1, fam(bin) link(identity)

estimates store I1

svy: glm current_smoke_n file2, fam(bin) link(identity)

estimates store I2

svy: glm current_smokeless_n file3, fam(bin) link(identity)

estimates store I3

svy: glm daily_tobacco_any file4, fam(bin) link(identity)

estimates store I4

svy: glm daily_smoke_n file5, fam(bin) link(identity)

estimates store I5

svy: glm daily_smokeless_n file6, fam(bin) link(identity)

estimates store I6

svy: glm smoke_only file7, fam(bin) link(identity)

estimates store I7

svy: glm smokeless_only file8, fam(bin) link(identity)

estimates store I8

svy: glm both_smoke_smokeless file9, fam(bin) link(identity)

estimates store I9

svy: glm alcohol_current file10, fam(bin) link(identity)

estimates store I10

svy: glm no_fruit_veg file11, fam(bin) link(identity)

estimates store I11

svy: glm no_fruit file12, fam(bin) link(identity)

estimates store I12

svy: glm no_veg file13, fam(bin) link(identity)

estimates store I13

svy: glm atelessthan5 file14, fam(bin) link(identity)

estimates store I14

svy: glm salt_eat_n file15, fam(bin) link(identity)

estimates store I15

svy: glm salt_pack_n file16, fam(bin) link(identity)

estimates store I16

svy: glm htn_known file17, fam(bin) link(identity)

estimates store I17

svy: glm htn_med file18, fam(bin) link(identity)

estimates store I18

estout I1 I2 I3 I4 I5 I6 I7 I8 I9 I10 I11 I12 I13 I14 I15 I16 I17 I18 using Table_RD.xls, ///

cells ("b(star fmt(1)) ci(par fmt(1) label(95% CI)) p (fmt(4)) ") replace transform(@*100)

import delimited "Table_RD.xls", clear

replace v2 = v5 if v2 == ""

replace v2 = v8 if v2 == ""

replace v2 = v11 if v2 == ""

replace v2 = v14 if v2 == ""

replace v2 = v17 if v2 == ""

replace v2 = v20 if v2 == ""

replace v2 = v23 if v2 == ""

replace v2 = v26 if v2 == ""

replace v2 = v29 if v2 == ""

replace v2 = v32 if v2 == ""

replace v2 = v35 if v2 == ""

replace v2 = v38 if v2 == ""

replace v2 = v41 if v2 == ""

replace v2 = v44 if v2 == ""

replace v2 = v47 if v2 == ""

replace v2 = v50 if v2 == ""

replace v2 = v53 if v2 == ""

replace v3 = v6 if v3 == ""

replace v3 = v9 if v3 == ""

replace v3 = v12 if v3 == ""

replace v3 = v15 if v3 == ""

replace v3 = v18 if v3 == ""

replace v3 = v21 if v3 == ""

replace v3 = v24 if v3 == ""

replace v3 = v27 if v3 == ""

replace v3 = v30 if v3 == ""

replace v3 = v33 if v3 == ""

replace v3 = v36 if v3 == ""

replace v3 = v39 if v3 == ""

replace v3 = v42 if v3 == ""

replace v3 = v45 if v3 == ""

replace v3 = v48 if v3 == ""

replace v3 = v51 if v3 == ""

replace v3 = v54 if v3 == ""

replace v4 = v7 if v4 == ""

replace v4 = v10 if v4 == ""

replace v4 = v13 if v4 == ""

replace v4 = v16 if v4 == ""

replace v4 = v19 if v4 == ""

replace v4 = v22 if v4 == ""

replace v4 = v25 if v4 == ""

replace v4 = v28 if v4 == ""

replace v4 = v31 if v4 == ""

replace v4 = v34 if v4 == ""

replace v4 = v37 if v4 == ""

replace v4 = v40 if v4 == ""

replace v4 = v43 if v4 == ""

replace v4 = v46 if v4 == ""

replace v4 = v49 if v4 == ""

replace v4 = v52 if v4 == ""

replace v4 = v55 if v4 == ""

keep v1 v2 v3 v4

drop if v1 == ""

drop if v1 == "main"

drop if v1 == "_cons"

destring v1, replace ignore(file)

rename v1 indicator

rename v2 Diff_IVR_IPW

rename v3 Diff_IVR_IPW_CI

rename v4 Diff_IVR_IPW_P

gen pop_group = 3

save File_Diff_IVR_IPW_3, replace

restore

*******

preserve

recode file1 (1 = 0) (0 = 1)

svyset [pweight= weight_ipw]

gen file2 = file1

gen file3 = file1

gen file4 = file1

gen file5 = file1

gen file6 = file1

gen file7 = file1

gen file8 = file1

gen file9 = file1

gen file10 = file1

gen file11 = file1

gen file12 = file1

gen file13 = file1

gen file14 = file1

gen file15 = file1

gen file16 = file1

gen file17 = file1

gen file18 = file1

svy: glm current_tobacco_any file1, fam(bin) link(identity)

estimates store I1

svy: glm current_smoke_n file2, fam(bin) link(identity)

estimates store I2

svy: glm current_smokeless_n file3, fam(bin) link(identity)

estimates store I3

svy: glm daily_tobacco_any file4, fam(bin) link(identity)

estimates store I4

svy: glm daily_smoke_n file5, fam(bin) link(identity)

estimates store I5

svy: glm daily_smokeless_n file6, fam(bin) link(identity)

estimates store I6

svy: glm smoke_only file7, fam(bin) link(identity)

estimates store I7

svy: glm smokeless_only file8, fam(bin) link(identity)

estimates store I8

svy: glm both_smoke_smokeless file9, fam(bin) link(identity)

estimates store I9

svy: glm alcohol_current file10, fam(bin) link(identity)

estimates store I10

svy: glm no_fruit_veg file11, fam(bin) link(identity)

estimates store I11

svy: glm no_fruit file12, fam(bin) link(identity)

estimates store I12

svy: glm no_veg file13, fam(bin) link(identity)

estimates store I13

svy: glm atelessthan5 file14, fam(bin) link(identity)

estimates store I14

svy: glm salt_eat_n file15, fam(bin) link(identity)

estimates store I15

svy: glm salt_pack_n file16, fam(bin) link(identity)

estimates store I16

svy: glm htn_known file17, fam(bin) link(identity)

estimates store I17

svy: glm htn_med file18, fam(bin) link(identity)

estimates store I18

estout I1 I2 I3 I4 I5 I6 I7 I8 I9 I10 I11 I12 I13 I14 I15 I16 I17 I18 using Table_RD.xls, ///

cells ("b(star fmt(1)) ci(par fmt(1) label(95% CI)) p (fmt(4)) ") replace transform(@*100)

import delimited "Table_RD.xls", clear

replace v2 = v5 if v2 == ""

replace v2 = v8 if v2 == ""

replace v2 = v11 if v2 == ""

replace v2 = v14 if v2 == ""

replace v2 = v17 if v2 == ""

replace v2 = v20 if v2 == ""

replace v2 = v23 if v2 == ""

replace v2 = v26 if v2 == ""

replace v2 = v29 if v2 == ""

replace v2 = v32 if v2 == ""

replace v2 = v35 if v2 == ""

replace v2 = v38 if v2 == ""

replace v2 = v41 if v2 == ""

replace v2 = v44 if v2 == ""

replace v2 = v47 if v2 == ""

replace v2 = v50 if v2 == ""

replace v2 = v53 if v2 == ""

replace v3 = v6 if v3 == ""

replace v3 = v9 if v3 == ""

replace v3 = v12 if v3 == ""

replace v3 = v15 if v3 == ""

replace v3 = v18 if v3 == ""

replace v3 = v21 if v3 == ""

replace v3 = v24 if v3 == ""

replace v3 = v27 if v3 == ""

replace v3 = v30 if v3 == ""

replace v3 = v33 if v3 == ""

replace v3 = v36 if v3 == ""

replace v3 = v39 if v3 == ""

replace v3 = v42 if v3 == ""

replace v3 = v45 if v3 == ""

replace v3 = v48 if v3 == ""

replace v3 = v51 if v3 == ""

replace v3 = v54 if v3 == ""

replace v4 = v7 if v4 == ""

replace v4 = v10 if v4 == ""

replace v4 = v13 if v4 == ""

replace v4 = v16 if v4 == ""

replace v4 = v19 if v4 == ""

replace v4 = v22 if v4 == ""

replace v4 = v25 if v4 == ""

replace v4 = v28 if v4 == ""

replace v4 = v31 if v4 == ""

replace v4 = v34 if v4 == ""

replace v4 = v37 if v4 == ""

replace v4 = v40 if v4 == ""

replace v4 = v43 if v4 == ""

replace v4 = v46 if v4 == ""

replace v4 = v49 if v4 == ""

replace v4 = v52 if v4 == ""

replace v4 = v55 if v4 == ""

keep v1 v2 v3 v4

drop if v1 == ""

drop if v1 == "main"

drop if v1 == "_cons"

destring v1, replace ignore(file)

rename v1 indicator

rename v2 Diff_IVR_IPW

rename v3 Diff_IVR_IPW_CI

rename v4 Diff_IVR_IPW_P

gen pop_group = 4

save File_Diff_IVR_IPW_4, replace

restore

*******

preserve

recode file1 (1 = 0) (0 = 1)

keep if age_cat == 0

keep if sex == 1

svyset [pweight= weight_ipw]

gen file2 = file1

gen file3 = file1

gen file4 = file1

gen file5 = file1

gen file6 = file1

gen file7 = file1

gen file8 = file1

gen file9 = file1

gen file10 = file1

gen file11 = file1

gen file12 = file1

gen file13 = file1

gen file14 = file1

gen file15 = file1

gen file16 = file1

gen file17 = file1

gen file18 = file1

svy: glm current_tobacco_any file1, fam(bin) link(identity)

estimates store I1

svy: glm current_smoke_n file2, fam(bin) link(identity)

estimates store I2

svy: glm current_smokeless_n file3, fam(bin) link(identity)

estimates store I3

svy: glm daily_tobacco_any file4, fam(bin) link(identity)

estimates store I4

svy: glm daily_smoke_n file5, fam(bin) link(identity)

estimates store I5

svy: glm daily_smokeless_n file6, fam(bin) link(identity)

estimates store I6

svy: glm smoke_only file7, fam(bin) link(identity)

estimates store I7

svy: glm smokeless_only file8, fam(bin) link(identity)

estimates store I8

svy: glm both_smoke_smokeless file9, fam(bin) link(identity)

estimates store I9

svy: glm alcohol_current file10, fam(bin) link(identity)

estimates store I10

svy: glm no_fruit_veg file11, fam(bin) link(identity)

estimates store I11

svy: glm no_fruit file12, fam(bin) link(identity)

estimates store I12

svy: glm no_veg file13, fam(bin) link(identity)

estimates store I13

svy: glm atelessthan5 file14, fam(bin) link(identity)

estimates store I14

svy: glm salt_eat_n file15, fam(bin) link(identity)

estimates store I15

svy: glm salt_pack_n file16, fam(bin) link(identity)

estimates store I16

svy: glm htn_known file17, fam(bin) link(identity)

estimates store I17

svy: glm htn_med file18, fam(bin) link(identity)

estimates store I18

estout I1 I2 I3 I4 I5 I6 I7 I8 I9 I10 I11 I12 I13 I14 I15 I16 I17 I18 using Table_RD.xls, ///

cells ("b(star fmt(1)) ci(par fmt(1) label(95% CI)) p (fmt(4)) ") replace transform(@*100)

import delimited "Table_RD.xls", clear

replace v2 = v5 if v2 == ""

replace v2 = v8 if v2 == ""

replace v2 = v11 if v2 == ""

replace v2 = v14 if v2 == ""

replace v2 = v17 if v2 == ""

replace v2 = v20 if v2 == ""

replace v2 = v23 if v2 == ""

replace v2 = v26 if v2 == ""

replace v2 = v29 if v2 == ""

replace v2 = v32 if v2 == ""

replace v2 = v35 if v2 == ""

replace v2 = v38 if v2 == ""

replace v2 = v41 if v2 == ""

replace v2 = v44 if v2 == ""

replace v2 = v47 if v2 == ""

replace v2 = v50 if v2 == ""

replace v2 = v53 if v2 == ""

replace v3 = v6 if v3 == ""

replace v3 = v9 if v3 == ""

replace v3 = v12 if v3 == ""

replace v3 = v15 if v3 == ""

replace v3 = v18 if v3 == ""

replace v3 = v21 if v3 == ""

replace v3 = v24 if v3 == ""

replace v3 = v27 if v3 == ""

replace v3 = v30 if v3 == ""

replace v3 = v33 if v3 == ""

replace v3 = v36 if v3 == ""

replace v3 = v39 if v3 == ""

replace v3 = v42 if v3 == ""

replace v3 = v45 if v3 == ""

replace v3 = v48 if v3 == ""

replace v3 = v51 if v3 == ""

replace v3 = v54 if v3 == ""

replace v4 = v7 if v4 == ""

replace v4 = v10 if v4 == ""

replace v4 = v13 if v4 == ""

replace v4 = v16 if v4 == ""

replace v4 = v19 if v4 == ""

replace v4 = v22 if v4 == ""

replace v4 = v25 if v4 == ""

replace v4 = v28 if v4 == ""

replace v4 = v31 if v4 == ""

replace v4 = v34 if v4 == ""

replace v4 = v37 if v4 == ""

replace v4 = v40 if v4 == ""

replace v4 = v43 if v4 == ""

replace v4 = v46 if v4 == ""

replace v4 = v49 if v4 == ""

replace v4 = v52 if v4 == ""

replace v4 = v55 if v4 == ""

keep v1 v2 v3 v4

drop if v1 == ""

drop if v1 == "main"

drop if v1 == "_cons"

destring v1, replace ignore(file)

rename v1 indicator

rename v2 Diff_IVR_IPW

rename v3 Diff_IVR_IPW_CI

rename v4 Diff_IVR_IPW_P

gen pop_group = 5

save File_Diff_IVR_IPW_5, replace

restore

******

preserve

recode file1 (1 = 0) (0 = 1)

keep if age_cat == 1

keep if sex == 1

svyset [pweight= weight_ipw]

gen file2 = file1

gen file3 = file1

gen file4 = file1

gen file5 = file1

gen file6 = file1

gen file7 = file1

gen file8 = file1

gen file9 = file1

gen file10 = file1

gen file11 = file1

gen file12 = file1

gen file13 = file1

gen file14 = file1

gen file15 = file1

gen file16 = file1

gen file17 = file1

gen file18 = file1

svy: glm current_tobacco_any file1, fam(bin) link(identity)

estimates store I1

svy: glm current_smoke_n file2, fam(bin) link(identity)

estimates store I2

svy: glm current_smokeless_n file3, fam(bin) link(identity)

estimates store I3

svy: glm daily_tobacco_any file4, fam(bin) link(identity)

estimates store I4

svy: glm daily_smoke_n file5, fam(bin) link(identity)

estimates store I5

svy: glm daily_smokeless_n file6, fam(bin) link(identity)

estimates store I6

svy: glm smoke_only file7, fam(bin) link(identity)

estimates store I7

svy: glm smokeless_only file8, fam(bin) link(identity)

estimates store I8

svy: glm both_smoke_smokeless file9, fam(bin) link(identity)

estimates store I9

svy: glm alcohol_current file10, fam(bin) link(identity)

estimates store I10

svy: glm no_fruit_veg file11, fam(bin) link(identity)

estimates store I11

svy: glm no_fruit file12, fam(bin) link(identity)

estimates store I12

svy: glm no_veg file13, fam(bin) link(identity)

estimates store I13

svy: glm atelessthan5 file14, fam(bin) link(identity)

estimates store I14

svy: glm salt_eat_n file15, fam(bin) link(identity)

estimates store I15

svy: glm salt_pack_n file16, fam(bin) link(identity)

estimates store I16

svy: glm htn_known file17, fam(bin) link(identity)

estimates store I17

svy: glm htn_med file18, fam(bin) link(identity)

estimates store I18

estout I1 I2 I3 I4 I5 I6 I7 I8 I9 I10 I11 I12 I13 I14 I15 I16 I17 I18 using Table_RD.xls, ///

cells ("b(star fmt(1)) ci(par fmt(1) label(95% CI)) p (fmt(4)) ") replace transform(@*100)

import delimited "Table_RD.xls", clear

replace v2 = v5 if v2 == ""

replace v2 = v8 if v2 == ""

replace v2 = v11 if v2 == ""

replace v2 = v14 if v2 == ""

replace v2 = v17 if v2 == ""

replace v2 = v20 if v2 == ""

replace v2 = v23 if v2 == ""

replace v2 = v26 if v2 == ""

replace v2 = v29 if v2 == ""

replace v2 = v32 if v2 == ""

replace v2 = v35 if v2 == ""

replace v2 = v38 if v2 == ""

replace v2 = v41 if v2 == ""

replace v2 = v44 if v2 == ""

replace v2 = v47 if v2 == ""

replace v2 = v50 if v2 == ""

replace v2 = v53 if v2 == ""

replace v3 = v6 if v3 == ""

replace v3 = v9 if v3 == ""

replace v3 = v12 if v3 == ""

replace v3 = v15 if v3 == ""

replace v3 = v18 if v3 == ""

replace v3 = v21 if v3 == ""

replace v3 = v24 if v3 == ""

replace v3 = v27 if v3 == ""

replace v3 = v30 if v3 == ""

replace v3 = v33 if v3 == ""

replace v3 = v36 if v3 == ""

replace v3 = v39 if v3 == ""

replace v3 = v42 if v3 == ""

replace v3 = v45 if v3 == ""

replace v3 = v48 if v3 == ""

replace v3 = v51 if v3 == ""

replace v3 = v54 if v3 == ""

replace v4 = v7 if v4 == ""

replace v4 = v10 if v4 == ""

replace v4 = v13 if v4 == ""

replace v4 = v16 if v4 == ""

replace v4 = v19 if v4 == ""

replace v4 = v22 if v4 == ""

replace v4 = v25 if v4 == ""

replace v4 = v28 if v4 == ""

replace v4 = v31 if v4 == ""

replace v4 = v34 if v4 == ""

replace v4 = v37 if v4 == ""

replace v4 = v40 if v4 == ""

replace v4 = v43 if v4 == ""

replace v4 = v46 if v4 == ""

replace v4 = v49 if v4 == ""

replace v4 = v52 if v4 == ""

replace v4 = v55 if v4 == ""

keep v1 v2 v3 v4

drop if v1 == ""

drop if v1 == "main"

drop if v1 == "_cons"

destring v1, replace ignore(file)

rename v1 indicator

rename v2 Diff_IVR_IPW

rename v3 Diff_IVR_IPW_CI

rename v4 Diff_IVR_IPW_P

gen pop_group = 6

save File_Diff_IVR_IPW_6, replace

restore

******

preserve

recode file1 (1 = 0) (0 = 1)

keep if age_cat == 2

keep if sex == 1

svyset [pweight= weight_ipw]

gen file2 = file1

gen file3 = file1

gen file4 = file1

gen file5 = file1

gen file6 = file1

gen file7 = file1

gen file8 = file1

gen file9 = file1

gen file10 = file1

gen file11 = file1

gen file12 = file1

gen file13 = file1

gen file14 = file1

gen file15 = file1

gen file16 = file1

gen file17 = file1

gen file18 = file1

svy: glm current_tobacco_any file1, fam(bin) link(identity)

estimates store I1

svy: glm current_smoke_n file2, fam(bin) link(identity)

estimates store I2

svy: glm current_smokeless_n file3, fam(bin) link(identity)

estimates store I3

svy: glm daily_tobacco_any file4, fam(bin) link(identity)

estimates store I4

svy: glm daily_smoke_n file5, fam(bin) link(identity)

estimates store I5

svy: glm daily_smokeless_n file6, fam(bin) link(identity)

estimates store I6

svy: glm smoke_only file7, fam(bin) link(identity)

estimates store I7

svy: glm smokeless_only file8, fam(bin) link(identity)

estimates store I8

svy: glm both_smoke_smokeless file9, fam(bin) link(identity)

estimates store I9

svy: glm alcohol_current file10, fam(bin) link(identity)

estimates store I10

svy: glm no_fruit_veg file11, fam(bin) link(identity)

estimates store I11

svy: glm no_fruit file12, fam(bin) link(identity)

estimates store I12

svy: glm no_veg file13, fam(bin) link(identity)

estimates store I13

svy: glm atelessthan5 file14, fam(bin) link(identity)

estimates store I14

svy: glm salt_eat_n file15, fam(bin) link(identity)

estimates store I15

svy: glm salt_pack_n file16, fam(bin) link(identity)

estimates store I16

svy: glm htn_known file17, fam(bin) link(identity)

estimates store I17

svy: glm htn_med file18, fam(bin) link(identity)

estimates store I18

estout I1 I2 I3 I4 I5 I6 I7 I8 I9 I10 I11 I12 I13 I14 I15 I16 I17 I18 using Table_RD.xls, ///

cells ("b(star fmt(1)) ci(par fmt(1) label(95% CI)) p (fmt(4)) ") replace transform(@*100)

import delimited "Table_RD.xls", clear

replace v2 = v5 if v2 == ""

replace v2 = v8 if v2 == ""

replace v2 = v11 if v2 == ""

replace v2 = v14 if v2 == ""

replace v2 = v17 if v2 == ""

replace v2 = v20 if v2 == ""

replace v2 = v23 if v2 == ""

replace v2 = v26 if v2 == ""

replace v2 = v29 if v2 == ""

replace v2 = v32 if v2 == ""

replace v2 = v35 if v2 == ""

replace v2 = v38 if v2 == ""

replace v2 = v41 if v2 == ""

replace v2 = v44 if v2 == ""

replace v2 = v47 if v2 == ""

replace v2 = v50 if v2 == ""

replace v2 = v53 if v2 == ""

replace v3 = v6 if v3 == ""

replace v3 = v9 if v3 == ""

replace v3 = v12 if v3 == ""

replace v3 = v15 if v3 == ""

replace v3 = v18 if v3 == ""

replace v3 = v21 if v3 == ""

replace v3 = v24 if v3 == ""

replace v3 = v27 if v3 == ""

replace v3 = v30 if v3 == ""

replace v3 = v33 if v3 == ""

replace v3 = v36 if v3 == ""

replace v3 = v39 if v3 == ""

replace v3 = v42 if v3 == ""

replace v3 = v45 if v3 == ""

replace v3 = v48 if v3 == ""

replace v3 = v51 if v3 == ""

replace v3 = v54 if v3 == ""

replace v4 = v7 if v4 == ""

replace v4 = v10 if v4 == ""

replace v4 = v13 if v4 == ""

replace v4 = v16 if v4 == ""

replace v4 = v19 if v4 == ""

replace v4 = v22 if v4 == ""

replace v4 = v25 if v4 == ""

replace v4 = v28 if v4 == ""

replace v4 = v31 if v4 == ""

replace v4 = v34 if v4 == ""

replace v4 = v37 if v4 == ""

replace v4 = v40 if v4 == ""

replace v4 = v43 if v4 == ""

replace v4 = v46 if v4 == ""

replace v4 = v49 if v4 == ""

replace v4 = v52 if v4 == ""

replace v4 = v55 if v4 == ""

keep v1 v2 v3 v4

drop if v1 == ""

drop if v1 == "main"

drop if v1 == "_cons"

destring v1, replace ignore(file)

rename v1 indicator

rename v2 Diff_IVR_IPW

rename v3 Diff_IVR_IPW_CI

rename v4 Diff_IVR_IPW_P

gen pop_group = 7

save File_Diff_IVR_IPW_7, replace

restore

******

preserve

recode file1 (1 = 0) (0 = 1)

keep if sex == 1

svyset [pweight= weight_ipw]

gen file2 = file1

gen file3 = file1

gen file4 = file1

gen file5 = file1

gen file6 = file1

gen file7 = file1

gen file8 = file1

gen file9 = file1

gen file10 = file1

gen file11 = file1

gen file12 = file1

gen file13 = file1

gen file14 = file1

gen file15 = file1

gen file16 = file1

gen file17 = file1

gen file18 = file1

svy: glm current_tobacco_any file1, fam(bin) link(identity)

estimates store I1

svy: glm current_smoke_n file2, fam(bin) link(identity)

estimates store I2

svy: glm current_smokeless_n file3, fam(bin) link(identity)

estimates store I3

svy: glm daily_tobacco_any file4, fam(bin) link(identity)

estimates store I4

svy: glm daily_smoke_n file5, fam(bin) link(identity)

estimates store I5

svy: glm daily_smokeless_n file6, fam(bin) link(identity)

estimates store I6

svy: glm smoke_only file7, fam(bin) link(identity)

estimates store I7

svy: glm smokeless_only file8, fam(bin) link(identity)

estimates store I8

svy: glm both_smoke_smokeless file9, fam(bin) link(identity)

estimates store I9

svy: glm alcohol_current file10, fam(bin) link(identity)

estimates store I10

svy: glm no_fruit_veg file11, fam(bin) link(identity)

estimates store I11

svy: glm no_fruit file12, fam(bin) link(identity)

estimates store I12

svy: glm no_veg file13, fam(bin) link(identity)

estimates store I13

svy: glm atelessthan5 file14, fam(bin) link(identity)

estimates store I14

svy: glm salt_eat_n file15, fam(bin) link(identity)

estimates store I15

svy: glm salt_pack_n file16, fam(bin) link(identity)

estimates store I16

svy: glm htn_known file17, fam(bin) link(identity)

estimates store I17

svy: glm htn_med file18, fam(bin) link(identity)

estimates store I18

estout I1 I2 I3 I4 I5 I6 I7 I8 I9 I10 I11 I12 I13 I14 I15 I16 I17 I18 using Table_RD.xls, ///

cells ("b(star fmt(1)) ci(par fmt(1) label(95% CI)) p (fmt(4)) ") replace transform(@*100)

import delimited "Table_RD.xls", clear

replace v2 = v5 if v2 == ""

replace v2 = v8 if v2 == ""

replace v2 = v11 if v2 == ""

replace v2 = v14 if v2 == ""

replace v2 = v17 if v2 == ""

replace v2 = v20 if v2 == ""

replace v2 = v23 if v2 == ""

replace v2 = v26 if v2 == ""

replace v2 = v29 if v2 == ""

replace v2 = v32 if v2 == ""

replace v2 = v35 if v2 == ""

replace v2 = v38 if v2 == ""

replace v2 = v41 if v2 == ""

replace v2 = v44 if v2 == ""

replace v2 = v47 if v2 == ""

replace v2 = v50 if v2 == ""

replace v2 = v53 if v2 == ""

replace v3 = v6 if v3 == ""

replace v3 = v9 if v3 == ""

replace v3 = v12 if v3 == ""

replace v3 = v15 if v3 == ""

replace v3 = v18 if v3 == ""

replace v3 = v21 if v3 == ""

replace v3 = v24 if v3 == ""

replace v3 = v27 if v3 == ""

replace v3 = v30 if v3 == ""

replace v3 = v33 if v3 == ""

replace v3 = v36 if v3 == ""

replace v3 = v39 if v3 == ""

replace v3 = v42 if v3 == ""

replace v3 = v45 if v3 == ""

replace v3 = v48 if v3 == ""

replace v3 = v51 if v3 == ""

replace v3 = v54 if v3 == ""

replace v4 = v7 if v4 == ""

replace v4 = v10 if v4 == ""

replace v4 = v13 if v4 == ""

replace v4 = v16 if v4 == ""

replace v4 = v19 if v4 == ""

replace v4 = v22 if v4 == ""

replace v4 = v25 if v4 == ""

replace v4 = v28 if v4 == ""

replace v4 = v31 if v4 == ""

replace v4 = v34 if v4 == ""

replace v4 = v37 if v4 == ""

replace v4 = v40 if v4 == ""

replace v4 = v43 if v4 == ""

replace v4 = v46 if v4 == ""

replace v4 = v49 if v4 == ""

replace v4 = v52 if v4 == ""

replace v4 = v55 if v4 == ""

keep v1 v2 v3 v4

drop if v1 == ""

drop if v1 == "main"

drop if v1 == "_cons"

destring v1, replace ignore(file)

rename v1 indicator

rename v2 Diff_IVR_IPW

rename v3 Diff_IVR_IPW_CI

rename v4 Diff_IVR_IPW_P

gen pop_group = 8

save File_Diff_IVR_IPW_8, replace

restore

******

preserve

recode file1 (1 = 0) (0 = 1)

keep if age_cat == 0

keep if sex == 2

svyset [pweight= weight_ipw]

gen file2 = file1

gen file3 = file1

gen file4 = file1

gen file5 = file1

gen file6 = file1

gen file7 = file1

gen file8 = file1

gen file9 = file1

gen file10 = file1

gen file11 = file1

gen file12 = file1

gen file13 = file1

gen file14 = file1

gen file15 = file1

gen file16 = file1

gen file17 = file1

gen file18 = file1

svy: glm current_tobacco_any file1, fam(bin) link(identity)

estimates store I1

svy: glm current_smoke_n file2, fam(bin) link(identity)

estimates store I2

svy: glm current_smokeless_n file3, fam(bin) link(identity)

estimates store I3

svy: glm daily_tobacco_any file4, fam(bin) link(identity)

estimates store I4

svy: glm daily_smoke_n file5, fam(bin) link(identity)

estimates store I5

svy: glm daily_smokeless_n file6, fam(bin) link(identity)

estimates store I6

svy: glm smoke_only file7, fam(bin) link(identity)

estimates store I7

svy: glm smokeless_only file8, fam(bin) link(identity)

estimates store I8

svy: glm both_smoke_smokeless file9, fam(bin) link(identity)

estimates store I9

svy: glm alcohol_current file10, fam(bin) link(identity)

estimates store I10

svy: glm no_fruit_veg file11, fam(bin) link(identity)

estimates store I11

svy: glm no_fruit file12, fam(bin) link(identity)

estimates store I12

svy: glm no_veg file13, fam(bin) link(identity)

estimates store I13

svy: glm atelessthan5 file14, fam(bin) link(identity)

estimates store I14

svy: glm salt_eat_n file15, fam(bin) link(identity)

estimates store I15

svy: glm salt_pack_n file16, fam(bin) link(identity)

estimates store I16

svy: glm htn_known file17, fam(bin) link(identity)

estimates store I17

svy: glm htn_med file18, fam(bin) link(identity)

estimates store I18

estout I1 I2 I3 I4 I5 I6 I7 I8 I9 I10 I11 I12 I13 I14 I15 I16 I17 I18 using Table_RD.xls, ///

cells ("b(star fmt(1)) ci(par fmt(1) label(95% CI)) p (fmt(4)) ") replace transform(@*100)

import delimited "Table_RD.xls", clear

replace v2 = v5 if v2 == ""

replace v2 = v8 if v2 == ""

replace v2 = v11 if v2 == ""

replace v2 = v14 if v2 == ""

replace v2 = v17 if v2 == ""

replace v2 = v20 if v2 == ""

replace v2 = v23 if v2 == ""

replace v2 = v26 if v2 == ""

replace v2 = v29 if v2 == ""

replace v2 = v32 if v2 == ""

replace v2 = v35 if v2 == ""

replace v2 = v38 if v2 == ""

replace v2 = v41 if v2 == ""

replace v2 = v44 if v2 == ""

replace v2 = v47 if v2 == ""

replace v2 = v50 if v2 == ""

replace v2 = v53 if v2 == ""

replace v3 = v6 if v3 == ""

replace v3 = v9 if v3 == ""

replace v3 = v12 if v3 == ""

replace v3 = v15 if v3 == ""

replace v3 = v18 if v3 == ""

replace v3 = v21 if v3 == ""

replace v3 = v24 if v3 == ""

replace v3 = v27 if v3 == ""

replace v3 = v30 if v3 == ""

replace v3 = v33 if v3 == ""

replace v3 = v36 if v3 == ""

replace v3 = v39 if v3 == ""

replace v3 = v42 if v3 == ""

replace v3 = v45 if v3 == ""

replace v3 = v48 if v3 == ""

replace v3 = v51 if v3 == ""

replace v3 = v54 if v3 == ""

replace v4 = v7 if v4 == ""

replace v4 = v10 if v4 == ""

replace v4 = v13 if v4 == ""

replace v4 = v16 if v4 == ""

replace v4 = v19 if v4 == ""

replace v4 = v22 if v4 == ""

replace v4 = v25 if v4 == ""

replace v4 = v28 if v4 == ""

replace v4 = v31 if v4 == ""

replace v4 = v34 if v4 == ""

replace v4 = v37 if v4 == ""

replace v4 = v40 if v4 == ""

replace v4 = v43 if v4 == ""

replace v4 = v46 if v4 == ""

replace v4 = v49 if v4 == ""

replace v4 = v52 if v4 == ""

replace v4 = v55 if v4 == ""

keep v1 v2 v3 v4

drop if v1 == ""

drop if v1 == "main"

drop if v1 == "_cons"

destring v1, replace ignore(file)

rename v1 indicator

rename v2 Diff_IVR_IPW

rename v3 Diff_IVR_IPW_CI

rename v4 Diff_IVR_IPW_P

gen pop_group = 9

save File_Diff_IVR_IPW_9, replace

restore

*******

******

preserve

recode file1 (1 = 0) (0 = 1)

keep if age_cat == 1

keep if sex == 2

svyset [pweight= weight_ipw]

gen file2 = file1

gen file3 = file1

gen file4 = file1

gen file5 = file1

gen file6 = file1

gen file7 = file1

gen file8 = file1

gen file9 = file1

gen file10 = file1

gen file11 = file1

gen file12 = file1

gen file13 = file1

gen file14 = file1

gen file15 = file1

gen file16 = file1

gen file17 = file1

gen file18 = file1

svy: glm current_tobacco_any file1, fam(bin) link(identity)

estimates store I1

svy: glm current_smoke_n file2, fam(bin) link(identity)

estimates store I2

svy: glm current_smokeless_n file3, fam(bin) link(identity)

estimates store I3

svy: glm daily_tobacco_any file4, fam(bin) link(identity)

estimates store I4

svy: glm daily_smoke_n file5, fam(bin) link(identity)

estimates store I5

svy: glm daily_smokeless_n file6, fam(bin) link(identity)

estimates store I6

svy: glm smoke_only file7, fam(bin) link(identity)

estimates store I7

svy: glm smokeless_only file8, fam(bin) link(identity)

estimates store I8

svy: glm both_smoke_smokeless file9, fam(bin) link(identity)

estimates store I9

svy: glm alcohol_current file10, fam(bin) link(identity)

estimates store I10

svy: glm no_fruit_veg file11, fam(bin) link(identity)

estimates store I11

svy: glm no_fruit file12, fam(bin) link(identity)

estimates store I12

svy: glm no_veg file13, fam(bin) link(identity)

estimates store I13

svy: glm atelessthan5 file14, fam(bin) link(identity)

estimates store I14

svy: glm salt_eat_n file15, fam(bin) link(identity)

estimates store I15

svy: glm salt_pack_n file16, fam(bin) link(identity)

estimates store I16

svy: glm htn_known file17, fam(bin) link(identity)

estimates store I17

svy: glm htn_med file18, fam(bin) link(identity)

estimates store I18

estout I1 I2 I3 I4 I5 I6 I7 I8 I9 I10 I11 I12 I13 I14 I15 I16 I17 I18 using Table_RD.xls, ///

cells ("b(star fmt(1)) ci(par fmt(1) label(95% CI)) p (fmt(4)) ") replace transform(@*100)

import delimited "Table_RD.xls", clear

replace v2 = v5 if v2 == ""

replace v2 = v8 if v2 == ""

replace v2 = v11 if v2 == ""

replace v2 = v14 if v2 == ""

replace v2 = v17 if v2 == ""

replace v2 = v20 if v2 == ""

replace v2 = v23 if v2 == ""

replace v2 = v26 if v2 == ""

replace v2 = v29 if v2 == ""

replace v2 = v32 if v2 == ""

replace v2 = v35 if v2 == ""

replace v2 = v38 if v2 == ""

replace v2 = v41 if v2 == ""

replace v2 = v44 if v2 == ""

replace v2 = v47 if v2 == ""

replace v2 = v50 if v2 == ""

replace v2 = v53 if v2 == ""

replace v3 = v6 if v3 == ""

replace v3 = v9 if v3 == ""

replace v3 = v12 if v3 == ""

replace v3 = v15 if v3 == ""

replace v3 = v18 if v3 == ""

replace v3 = v21 if v3 == ""

replace v3 = v24 if v3 == ""

replace v3 = v27 if v3 == ""

replace v3 = v30 if v3 == ""

replace v3 = v33 if v3 == ""

replace v3 = v36 if v3 == ""

replace v3 = v39 if v3 == ""

replace v3 = v42 if v3 == ""

replace v3 = v45 if v3 == ""

replace v3 = v48 if v3 == ""

replace v3 = v51 if v3 == ""

replace v3 = v54 if v3 == ""

replace v4 = v7 if v4 == ""

replace v4 = v10 if v4 == ""

replace v4 = v13 if v4 == ""

replace v4 = v16 if v4 == ""

replace v4 = v19 if v4 == ""

replace v4 = v22 if v4 == ""

replace v4 = v25 if v4 == ""

replace v4 = v28 if v4 == ""

replace v4 = v31 if v4 == ""

replace v4 = v34 if v4 == ""

replace v4 = v37 if v4 == ""

replace v4 = v40 if v4 == ""

replace v4 = v43 if v4 == ""

replace v4 = v46 if v4 == ""

replace v4 = v49 if v4 == ""

replace v4 = v52 if v4 == ""

replace v4 = v55 if v4 == ""

keep v1 v2 v3 v4

drop if v1 == ""

drop if v1 == "main"

drop if v1 == "_cons"

destring v1, replace ignore(file)

rename v1 indicator

rename v2 Diff_IVR_IPW

rename v3 Diff_IVR_IPW_CI

rename v4 Diff_IVR_IPW_P

gen pop_group = 10

save File_Diff_IVR_IPW_10, replace

restore

*******

******

preserve

recode file1 (1 = 0) (0 = 1)

keep if age_cat == 2

keep if sex == 2

svyset [pweight= weight_ipw]

gen file2 = file1

gen file3 = file1

gen file4 = file1

gen file5 = file1

gen file6 = file1

gen file7 = file1

gen file8 = file1

gen file9 = file1

gen file10 = file1

gen file11 = file1

gen file12 = file1

gen file13 = file1

gen file14 = file1

gen file15 = file1

gen file16 = file1

gen file17 = file1

gen file18 = file1

svy: glm current_tobacco_any file1, fam(bin) link(identity)

estimates store I1

svy: glm current_smoke_n file2, fam(bin) link(identity)

estimates store I2

svy: glm current_smokeless_n file3, fam(bin) link(identity)

estimates store I3

svy: glm daily_tobacco_any file4, fam(bin) link(identity)

estimates store I4

svy: glm daily_smoke_n file5, fam(bin) link(identity)

estimates store I5

svy: glm daily_smokeless_n file6, fam(bin) link(identity)

estimates store I6

svy: glm smoke_only file7, fam(bin) link(identity)

estimates store I7

svy: glm smokeless_only file8, fam(bin) link(identity)

estimates store I8

svy: glm both_smoke_smokeless file9, fam(bin) link(identity)

estimates store I9

svy: glm alcohol_current file10, fam(bin) link(identity)

estimates store I10

svy: glm no_fruit_veg file11, fam(bin) link(identity)

estimates store I11

svy: glm no_fruit file12, fam(bin) link(identity)

estimates store I12

svy: glm no_veg file13, fam(bin) link(identity)

estimates store I13

svy: glm atelessthan5 file14, fam(bin) link(identity)

estimates store I14

svy: glm salt_eat_n file15, fam(bin) link(identity)

estimates store I15

svy: glm salt_pack_n file16, fam(bin) link(identity)

estimates store I16

svy: glm htn_known file17, fam(bin) link(identity)

estimates store I17

svy: glm htn_med file18, fam(bin) link(identity)

estimates store I18

estout I1 I2 I3 I4 I5 I6 I7 I8 I9 I10 I11 I12 I13 I14 I15 I16 I17 I18 using Table_RD.xls, ///

cells ("b(star fmt(1)) ci(par fmt(1) label(95% CI)) p (fmt(4)) ") replace transform(@*100)

import delimited "Table_RD.xls", clear

replace v2 = v5 if v2 == ""

replace v2 = v8 if v2 == ""

replace v2 = v11 if v2 == ""

replace v2 = v14 if v2 == ""

replace v2 = v17 if v2 == ""

replace v2 = v20 if v2 == ""

replace v2 = v23 if v2 == ""

replace v2 = v26 if v2 == ""

replace v2 = v29 if v2 == ""

replace v2 = v32 if v2 == ""

replace v2 = v35 if v2 == ""

replace v2 = v38 if v2 == ""

replace v2 = v41 if v2 == ""

replace v2 = v44 if v2 == ""

replace v2 = v47 if v2 == ""

replace v2 = v50 if v2 == ""

replace v2 = v53 if v2 == ""

replace v3 = v6 if v3 == ""

replace v3 = v9 if v3 == ""

replace v3 = v12 if v3 == ""

replace v3 = v15 if v3 == ""

replace v3 = v18 if v3 == ""

replace v3 = v21 if v3 == ""

replace v3 = v24 if v3 == ""

replace v3 = v27 if v3 == ""

replace v3 = v30 if v3 == ""

replace v3 = v33 if v3 == ""

replace v3 = v36 if v3 == ""

replace v3 = v39 if v3 == ""

replace v3 = v42 if v3 == ""

replace v3 = v45 if v3 == ""

replace v3 = v48 if v3 == ""

replace v3 = v51 if v3 == ""

replace v3 = v54 if v3 == ""

replace v4 = v7 if v4 == ""

replace v4 = v10 if v4 == ""

replace v4 = v13 if v4 == ""

replace v4 = v16 if v4 == ""

replace v4 = v19 if v4 == ""

replace v4 = v22 if v4 == ""

replace v4 = v25 if v4 == ""

replace v4 = v28 if v4 == ""

replace v4 = v31 if v4 == ""

replace v4 = v34 if v4 == ""

replace v4 = v37 if v4 == ""

replace v4 = v40 if v4 == ""

replace v4 = v43 if v4 == ""

replace v4 = v46 if v4 == ""

replace v4 = v49 if v4 == ""

replace v4 = v52 if v4 == ""

replace v4 = v55 if v4 == ""

keep v1 v2 v3 v4

drop if v1 == ""

drop if v1 == "main"

drop if v1 == "_cons"

destring v1, replace ignore(file)

rename v1 indicator

rename v2 Diff_IVR_IPW

rename v3 Diff_IVR_IPW_CI

rename v4 Diff_IVR_IPW_P

gen pop_group = 11

save File_Diff_IVR_IPW_11, replace

restore

******

preserve

recode file1 (1 = 0) (0 = 1)

keep if sex == 2

svyset [pweight= weight_ipw]

gen file2 = file1

gen file3 = file1

gen file4 = file1

gen file5 = file1

gen file6 = file1

gen file7 = file1

gen file8 = file1

gen file9 = file1

gen file10 = file1

gen file11 = file1

gen file12 = file1

gen file13 = file1

gen file14 = file1

gen file15 = file1

gen file16 = file1

gen file17 = file1

gen file18 = file1

svy: glm current_tobacco_any file1, fam(bin) link(identity)

estimates store I1

svy: glm current_smoke_n file2, fam(bin) link(identity)

estimates store I2

svy: glm current_smokeless_n file3, fam(bin) link(identity)

estimates store I3

svy: glm daily_tobacco_any file4, fam(bin) link(identity)

estimates store I4

svy: glm daily_smoke_n file5, fam(bin) link(identity)

estimates store I5

svy: glm daily_smokeless_n file6, fam(bin) link(identity)

estimates store I6

svy: glm smoke_only file7, fam(bin) link(identity)

estimates store I7

svy: glm smokeless_only file8, fam(bin) link(identity)

estimates store I8

svy: glm both_smoke_smokeless file9, fam(bin) link(identity)

estimates store I9

svy: glm alcohol_current file10, fam(bin) link(identity)

estimates store I10

svy: glm no_fruit_veg file11, fam(bin) link(identity)

estimates store I11

svy: glm no_fruit file12, fam(bin) link(identity)

estimates store I12

svy: glm no_veg file13, fam(bin) link(identity)

estimates store I13

svy: glm atelessthan5 file14, fam(bin) link(identity)

estimates store I14

svy: glm salt_eat_n file15, fam(bin) link(identity)

estimates store I15

svy: glm salt_pack_n file16, fam(bin) link(identity)

estimates store I16

svy: glm htn_known file17, fam(bin) link(identity)

estimates store I17

svy: glm htn_med file18, fam(bin) link(identity)

estimates store I18

estout I1 I2 I3 I4 I5 I6 I7 I8 I9 I10 I11 I12 I13 I14 I15 I16 I17 I18 using Table_RD.xls, ///

cells ("b(star fmt(1)) ci(par fmt(1) label(95% CI)) p (fmt(4)) ") replace transform(@*100)

import delimited "Table_RD.xls", clear

replace v2 = v5 if v2 == ""

replace v2 = v8 if v2 == ""

replace v2 = v11 if v2 == ""

replace v2 = v14 if v2 == ""

replace v2 = v17 if v2 == ""

replace v2 = v20 if v2 == ""

replace v2 = v23 if v2 == ""

replace v2 = v26 if v2 == ""

replace v2 = v29 if v2 == ""

replace v2 = v32 if v2 == ""

replace v2 = v35 if v2 == ""

replace v2 = v38 if v2 == ""

replace v2 = v41 if v2 == ""

replace v2 = v44 if v2 == ""

replace v2 = v47 if v2 == ""

replace v2 = v50 if v2 == ""

replace v2 = v53 if v2 == ""

replace v3 = v6 if v3 == ""

replace v3 = v9 if v3 == ""

replace v3 = v12 if v3 == ""

replace v3 = v15 if v3 == ""

replace v3 = v18 if v3 == ""

replace v3 = v21 if v3 == ""

replace v3 = v24 if v3 == ""

replace v3 = v27 if v3 == ""

replace v3 = v30 if v3 == ""

replace v3 = v33 if v3 == ""

replace v3 = v36 if v3 == ""

replace v3 = v39 if v3 == ""

replace v3 = v42 if v3 == ""

replace v3 = v45 if v3 == ""

replace v3 = v48 if v3 == ""

replace v3 = v51 if v3 == ""

replace v3 = v54 if v3 == ""

replace v4 = v7 if v4 == ""

replace v4 = v10 if v4 == ""

replace v4 = v13 if v4 == ""

replace v4 = v16 if v4 == ""
[truncated: 331,164 more chars]
