# Supplementary material for: Developing digital tools for health surveys in low- and middle-income countries: Comparing findings of two mobile phone surveys with a nationally representative in-person survey in Bangladesh
Source: PLOS Glob Public Health. 2023 Jul 27;3(7):e0002053. doi: 10.1371/journal.pgph.0002053 (PMC10374008; doi:10.1371/journal.pgph.0002053)
Supplement: S1 Table — (DOCX) [file pgph.0002053.s004.docx]

S1 Table: Prevalence (95% CI) of the studied indicators according to survey mode

among people with ‘up to primary’ education level

| Indicators | CATI | IVR | STEPS |
| --- | --- | --- | --- |
| Current smoker | 23.6 [21.4,26.0] | 19.7 [15.8,24.3] | 25.5 [23.8,27.1] |
| Current smokeless tobacco user | 23.9 [21.7,26.3] | 15.2 [11.2,20.4] | 33.1 [31.3,34.8] |
| Daily smoker | 19.0 [17.0,21.2] | 17.2 [13.6,21.7] | 24.4 [22.8,26.1] |
| Daily smokeless tobacco user | 16.0 [14.2,18.0] | 7.9 [5.2,11.7] | 29.0 [27.4,30.7] |
| Alcohol past month | 1.8 [1.2,2.7] | 2.6 [1.4,4.8] | 1.5 [1.1,2.2] |
| <5 servings of fruits-veg in a day | 33.7 [31.1,36.4] | 47.2 [41.2,53.3] | 90.1 [88.9,91.1] |
| Add salt to food while eating | 36.9 [34.2,39.6] | 29.4 [24.2,35.1] | 51.7 [49.8,53.7] |
| Processed food high in salt | 11.1 [9.5,13.0] | 24.6 [19.8,30.0] | 12.2 [10.9,13.5] |
| Known raised BP/HTN | 14.5 [12.7,16.5] | 16.6 [12.3,22.1] | 13.5 [12.3,14.8] |
| Take medication for BP/HTN | 55.0 [47.9,61.8] | 56.4 [40.6,71.0] | 78.4 [74.3,81.9] |

Abbreviations: CATI: Computer Assisted Telephone Interview; CI: Confidence interval; IVR: Interactive Voice Response
