# Supplementary material for: Developing digital tools for health surveys in low- and middle-income countries: Comparing findings of two mobile phone surveys with a nationally representative in-person survey in Bangladesh
Source: PLOS Glob Public Health. 2023 Jul 27;3(7):e0002053. doi: 10.1371/journal.pgph.0002053 (PMC10374008; doi:10.1371/journal.pgph.0002053)
Supplement: S2 Table — (DOCX) [file pgph.0002053.s005.docx]

S2 Table: Prevalence (95% CI) of the studied indicators according to survey mode

among people with ‘more than primary’ to ‘up to secondary’ education level

| Indicators | CATI | IVR | STEPS |
| --- | --- | --- | --- |
| Current smoker | 13.3 [10.3,17.0] | 15.4 [12.3,19.0] | 18.6 [14.9,22.9] |
| Current smokeless tobacco user | 11.2 [8.3,14.9] | 8.2 [5.7,11.7] | 11.4 [8.6,15.0] |
| Daily smoker | 7.7 [5.5,10.7] | 13.0 [10.2,16.5] | 17.6 [14.0,22.0] |
| Daily smokeless tobacco user | 5.1 [3.4,7.7] | 5.1 [3.0,8.5] | 8.3 [6.0,11.4] |
| Alcohol past month | 1.1 [0.4,2.9] | 1.1 [0.5,2.3] | 1.4 [0.5,3.6] |
| <5 servings of fruits-veg in a day | 33.7 [28.8,38.9] | 49.7 [44.4,54.9] | 88.9 [85.5,91.5] |
| Add salt to food while eating | 28.4 [23.8,33.6] | 29.5 [24.9,34.6] | 45.7 [40.3,51.1] |
| Processed food high in salt | 15.9 [12.4,20.2] | 18.5 [14.6,23.1] | 20.1 [16.0,24.8] |
| Known raised BP/HTN | 12.2 [9.4,15.5] | 11.8 [8.6,16.0] | 11.5 [8.8,14.9] |
| Take medication for BP/HTN | 57.6 [44.3,69.9] | 64.8 [48.6,78.2] | 75.4 [62.4,85.0] |

Abbreviations: CATI: Computer Assisted Telephone Interview; CI: Confidence interval; IVR: Interactive Voice Response
