# Supplementary material for: Developing digital tools for health surveys in low- and middle-income countries: Comparing findings of two mobile phone surveys with a nationally representative in-person survey in Bangladesh
Source: PLOS Glob Public Health. 2023 Jul 27;3(7):e0002053. doi: 10.1371/journal.pgph.0002053 (PMC10374008; doi:10.1371/journal.pgph.0002053)
Supplement: S3 Table — (DOCX) [file pgph.0002053.s006.docx]

S3 Table: Prevalence (95% CI) of the studied indicators according to survey mode

among people with ‘more than secondary’ education level

| Indicators | CATI | IVR | STEPS |
| --- | --- | --- | --- |
| Current smoker | 16.0 [13.3,19.1] | 9.7 [7.9,11.8] | 16.8 [13.3,21.0] |
| Current smokeless tobacco user | 8.6 [6.7,11.1] | 4.4 [3.0,6.5] | 9.9 [7.6,12.8] |
| Daily smoker | 12.0 [9.7,14.8] | 7.1 [5.6,8.9] | 14.7 [11.5,18.7] |
| Daily smokeless tobacco user | 3.1 [2.1,4.6] | 2.1 [1.1,3.9] | 8.2 [6.0,10.9] |
| Alcohol past month | 0.7 [0.3,1.6] | 1.1 [0.6,1.8] | 1.1 [0.5,2.6] |
| <5 servings of fruits-veg in a day | 28.2 [24.7,32.1] | 56.6 [52.7,60.4] | 88.1 [84.3,91.1] |
| Add salt to food while eating | 21.8 [18.6,25.5] | 19.2 [16.2,22.5] | 30.2 [25.6,35.3] |
| Processed food high in salt | 12.8 [10.2,15.8] | 9.3 [7.2,11.9] | 14.7 [11.5,18.6] |
| Known raised BP/HTN | 14.0 [11.7,16.8] | 20.5 [17.5,24.0] | 16.8 [13.7,20.6] |
| Take medication for BP/HTN | 66.6 [57.1,74.9] | 53.1 [44.1,61.9] | 73.3 [62.1,82.1] |

Abbreviations: CATI: Computer Assisted Telephone Interview; CI: Confidence interval; IVR: Interactive Voice Response
